# Images of the unseen: extrapolating visual representations for abstract and concrete words in a data-driven computational model

**DOI:** 10.1007/s00426-020-01429-7

**Published:** 2020-11-12

**Authors:** Fritz Günther, Marco Alessandro Petilli, Alessandra Vergallito, Marco Marelli

**Affiliations:** 1 University of Tübingen, Tübingen, Germany; 2 University of Milano–Bicocca, Milan, Italy; 3 NeuroMI, Milan Center for Neuroscience, Milan, Italy

**Keywords:** Grounded Cognition, Deep Learning, Distributional Semantics, Abstract concepts, Abstract words, Concreteness

## Abstract

Theories of grounded cognition assume that conceptual representations are grounded in sensorimotor experience. However, abstract concepts such as *jealousy* or *childhood* have no directly associated referents with which such sensorimotor experience can be made; therefore, the grounding of abstract concepts has long been a topic of debate. Here, we propose (a) that systematic relations exist between semantic representations learned from language on the one hand and perceptual experience on the other hand, (b) that these relations can be learned in a bottom-up fashion, and (c) that it is possible to extrapolate from this learning experience to predict expected perceptual representations for words even where direct experience is missing. To test this, we implement a data-driven computational model that is trained to map language-based representations (obtained from text corpora, representing language experience) onto vision-based representations (obtained from an image database, representing perceptual experience), and apply its mapping function onto language-based representations for abstract and concrete words outside the training set. In three experiments, we present participants with these words, accompanied by two images: the image predicted by the model and a random control image. Results show that participants’ judgements were in line with model predictions even for the most abstract words. This preference was stronger for more concrete items and decreased for the more abstract ones. Taken together, our findings have substantial implications in support of the grounding of abstract words, suggesting that we can tap into our previous experience to create possible visual representation we don’t have.

**Electronic supplementary material:**

The online version of this article (10.1007/s00426-020-01429-7) contains supplementary material, which is available to authorized users.

## Introduction

One of the most debated topics of cognitive science concerns the way conceptual representation are acquired and organized. This issue became even more central over the last two decades due to the influence of the grounded cognition framework, which claims that concepts are represented at a sensorimotor level (Barsalou, 1999; Fischer, 2012; Glenberg, 2015; Zwaan & Madden, 2005). According to this view, our semantic memory cannot be a self-contained system in which all the representations are abstract, amodal symbols that are defined exclusively by their relations to one another (see for example Collins & Quillian, 1969; Kintsch, 1988). The best-known argument against this conceptualization is provided by Harnad’s (1990) adaptation of Searle’s (1980) Chinese room argument: If a monolingual English speaker suddenly finds herself in China, only equipped with a monolingual Chinese-Chinese dictionary, she will never be able to understand anything. In this case, whenever she looks up any symbol, it is only ever linked to other symbols that have no meaning for her. At some point, she needs the symbols to be grounded in a format she is able to understand—in this concrete example, this could be English words or pictures. This argument directly translates to our semantic memory: At some point, semantic representations need to be grounded in a primary format of our cognitive system. This is the core assumption of theories of grounded cognition (e.g. Barsalou, 1999; Glenberg & Kaschak, 2002; Glenberg & Robertson, 2000; Zwaan & Madden, 2005), which postulate that perceptual and motor systems take on this vital grounding role. Simply speaking, in order to understand a word such as *horse*, the cognitive system re-activates sensorimotor experience with the word referent, such as visual experience with a horse standing in a stable or running on a field. This sensorimotor experience can be linked to linguistic experience by systematic patterns of co-occurrence (for example, by hearing the word *horse* while seeing a horse on a field; Zwaan & Madden, 2005), with several studies suggesting this connection to be established as the result of Hebbian learning at the brain level (see also Pulvermüller 2005; Hoenig et al., 2011; Kiefer et al., 2007; Trumpp and Kiefer, 2018).

Such a co-occurrence-based grounding mechanism appears to be straightforward for concrete words, which refer to clearly identifiable objects that can be perceived with our senses. However, it is far less obvious how grounding would be achieved when this is not the case (Barsalou 2016; Borghi et al. 2017). One prime example are abstract words such as *libertarianism*, *jealousy*, or *childhood*, which by definition do not refer to a distinct class of physical objects (for an overview, see Borghi et al. 2017). However, it should be noted that these issues already arise for concrete words whose referents one has never experienced directly, such as *Atlantis* or *supernova* (Günther, Dudschig, & Kaup, 2018; Günther and Nguyen, et al., 2020). The question of how can we achieve grounding in the absence of any direct sensorimotor experience is of central importance for theories of grounded cognition (Borghi et al. 2017); if they can account for only a fraction of words that are directly experienced, the usefulness and adequacy of grounded cognition theories as a general-level cognitive theory stands in question.

Over the recent years many different proposals have been made addressing this issue (see Barsalou, Santos, Simmons, & Wilson, 2008; Borghi & Binkofski, 2014; Glenberg, Sato, & Cattaneo, 2008; Harpaintner, Trumpp, & Kiefer, 2018; Harpaintner, Sim, Trumpp, Ulrich, & Kiefer, 2020; Hoffman, McClelland, & Lambon Ralph, 2018; Kousta, Vigliocco, Vinson, Andrews, & Del Campo, 2011; Lakoff & Johnson, 2008; Wilson-Mendenhall, Simmons, Martin, & Barsalou, 2013, for different theoretical approaches). One possible mechanism of how grounding can be established in the absence of experience (referred to as *acquired embodiment* by Hoffman et al., 2018 and *indirect grounding* by Günther, Nguyen, et al., 2020) is best illustrated by an example: Assume a friend tells you, *“On my way here, I saw a little wibby chirping in a tree!”*. You have never heard the word *wibby* before, but it does not seem difficult to imagine how it would look like: A small animal with feathers, wings, and a beak. Thus, due to the way *wibby* was used in language—similar to *bird*, *robin*, or *sparrow*—its semantic representation is similar to these words for which visual experience is available (Landauer and Dumais 1997; Lenci 2008), and you can draw on this information to predict a likely visual representation. In other words, one can *map* a semantic representation formed through linguistic experience onto perceptual experience, by exploiting systematic language-to-vision relations learned before. Note that this is not restricted to simply substituting a word with an already grounded one and retrieving the associated experience: If your friend adds the sentence “They used to build these wibbies from white steel, but nowadays it’s just aluminium.”, the visual representation probably changed to some robotic bird—something you most likely have never seen before. Thus, from this purely linguistic input, one can extrapolate from available experience, draw inferences about what a *wibby* would most likely look like, and simulate the corresponding visual experience.

In the present study, we investigate whether such a mapping can be reliably achieved for word meanings learned from language alone (i.e. without any accompanying direct experience, be it sensorimotor or emotional)—for concrete words, and crucially also for abstract words (see Hoffman et al., 2018). More specifically, we test whether language-based representations in our semantic memory along with their relation to their associated vision-based representations provide the necessary structural information to reliably map *remaining* language-based representations (i.e., the ones for which no vision-based representations is available) onto the visual domain. This can be achieved by exploiting (a) systematic relations between language-based semantic representations and visual representations (for example, birds usually have wings), and (b) the structure of similarity among language-based representations themselves (in the example above, *wibby* is used in a similar way as words denoting birds). To test this, we implement a data-driven, computational model in which both language-based and vision-based representations are conceptualized in a high-dimensional vector format. In the following section, we will first describe the model in detail; we will then discuss the perspective it provides on the grounding problem for abstract words, before putting it to empirical test.

## The mapping model

In the model presented here, we employ a distributional semantics framework to model language-based semantic representations, a deep neural network computer-vision approach to model visual representations, and train a simple linear function to establish a mapping from the former to the latter.

### Language-based semantic representations

Language-based representations were obtained via the distributional semantics framework (Günther, Rinaldi, & Marelli, 2019; Landauer & Dumais, 1997; Turney & Pantel, 2010). These models are based on the distributional hypothesis that words with similar meanings are used in a similar manner (Wittgenstein 1953) and thus occur in similar (linguistic) contexts (Harris, 1954; Lenci, 2008). Consequently, distributional semantic models estimate a word meaning from its distribution over linguistic contexts in large corpora of natural language, resulting in a representation of word meanings as high-dimensional numerical vectors. Over the past decades, these models have received strong empirical (e.g. Baroni, Dinu, & Kruszewski, 2014; Jones, Kintsch, & Mewhort, 2006; Mandera, Keuleers, & Brysbaert, 2017;Pereira, Gershman, Ritter, & Botvinick, 2016) and theoretical support (Günther et al. 2019; Jones et al. 2015; Westbury 2016) as models of human semantic memory.

**Fig. 1:**
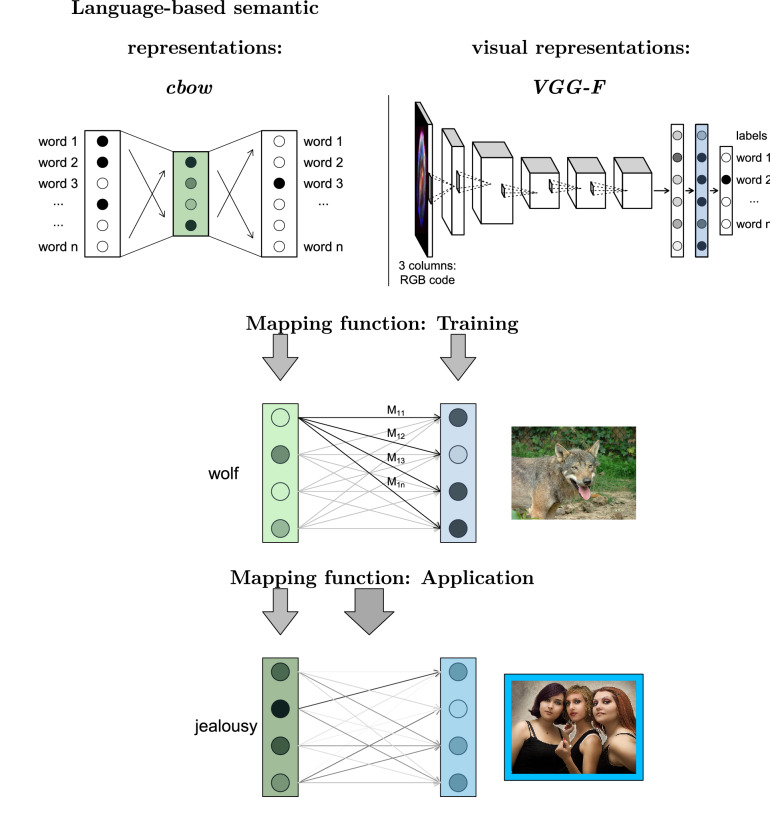
Graphical illustration of the workflow of the mapping model. A linear function is estimated from a training set that maps text-based vectors onto image-based vectors. The trained function is then applied to text-based vectors outside the training set to predict visual representations. The closest image to this visual representation (marked here with a blue frame) is then taken as the predicted image

There are many different possible parametrizations for distributional semantic models (Jones et al. 2015). In the present study, we employed the model with the overall best performance in a systematic evaluation by Baroni et al. (2014): a system trained using the *cbow* algorithm (with 400-dimensions vectors, negative sampling with $k = 10$, and subsampling with $t = 1e^{-5}$ as parameter settings) of the *word2vec* model (Mikolov et al. 2013, 2013). The *cbow* algorithm is aimed at predicting a target word from its context (here, the context of a word is defined as the 5 words to its left and to its right), using a neural network model with one hidden layer (see the top left illustration in Fig. 1). The vector representing a given word meaning is then estimated as the activation level of the units in the hidden layer once the system is fed this target word. Besides its good empirical performance, several studies have also identified the *cbow* model as a psychologically plausible learning model for the acquisition of semantic representations (Hollis, 2017; Mandera et al. 2017).

This model was trained on an English $\sim$ 2.8 billion word source corpus (a concatenation of the ukWaC corpus, Baroni, Bernardini, Ferraresi, & Zanchetta, 2009; an English Wikipedia dump; and the British National corpus, BNC Consortium, 2007), while considering the 300,000 most frequent words in the corpus as target and context words.

### Vision-based representations

Vision-based representations were obtained via a state-of-the-art computer vision model (Krizhevsky, Sutskever, & Hinton, 2012). This model employs a deep neural network that is trained to predict (human-generated) image labels from a vector representation encoding the pixel-based RGB values of the respective image (see the upper-right part of Fig. 1). Besides excelling at image labeling (Chatfield, Simonyan, Vedaldi, & Zisserman, 2014; Krizhevsky et al., 2012)—the task for which these neural-networks models are explicitly trained—they also provide high-quality measures of visual similarity (Petilli, Günther, Vergallito, Ciapparelli, & Marelli, 2019), which closely correspond to human intuitions (Bracci, Ritchie, Kalfas, & de Beeck, 2019; Lazaridou, Marelli, & Baroni, 2017; Phillips et al., 2018; Zhang, Isola, Efros, Shechtman, & Wang, 2018).

These models are trained on a large set of images that were annotated with noun labels by human raters, usually collected from the ImageNet database (Deng et al., 2009; see Krizhevsky et al., 2012). The pixel-based RGB values of the images serve as the input layer of an eight-layer deep convolutional neural network, and the image labels as the output to be predicted (see the top right illustration in Fig. 1). We extracted images for all 7801 labels for which (a) language-based representations were available (see above), and (b) at least 100 different images were available from the ImageNet database. For labels associated to a maximum of 200 of images in ImageNet, we extracted all the images available; if more images were available, 200 of them were randomly selected and extracted (the median original number of images per label was 1178; $Q_1 = 561$, $Q_3 = 1418$, max = 7908). In cases where a set of images was annotated with multiple labels in ImageNet, we only considered the label with the highest word frequency (the median original number of labels per set of images was 1; $Q_1 = 1$, $Q_3 = 2$, max =11; in cases where more than one label was used, these were mostly synonyms, such as *abbess—mother superior—prioress* or *albatross—mollymawk*).

We then employed the pre-trained VGG-F model (Chatfield et al. 2014), as implemented in the MatConvNet Matlab toolbox (Vedaldi and Lenc 2015), to obtain visual representations for these images. As established in previous studies, (e.g. Günther, Petilli, & Marelli, 2020; Lazaridou et al., 2017; Petilli et al., 2019), we extracted the activation values of the 4,096-dimensional second-to-last layer of the network as visual representations. In previous studies, the exact same model was already successfully employed to predict psychologically relevant phenomena, such as visual effects in priming (Petilli et al. 2019) and visual effects in conceptual combination (Günther et al. 2020). These pieces of evidence speak for the validity of the model, empirically showing it can be fruitfully applied to research in the cognitive science domain.

### Language-to-vision mapping

The final component of our model is a mapping function from the language-based semantic representations (in the form of distributional vectors) onto the vision-based representations associated to the respective label (see the middle and lower part of Fig. 1). Such mapping functions between distributional vectors and sensorimotor information have previously been successfully implemented for participant ratings on various sensorimotor features (Sommerauer and Fokkens 2018; Utsumi 2020) or emotional features (Martínez-Huertas, Jorge-Botana, Luzón, & Olmos, in press) associated to words.

In the model presented here, we implemented one of the simplest possible mapping functions: a linear function $m: \text {language} \rightarrow \text {vision}$, so that ${\hat{v}} = m(l) = M\cdot l$ for $l \in \text {language}$ and $v \in \text {vision}$ (${\hat{v}}$ being a *predicted* vision-based representation). *M* is estimated as a matrix where the cell entries $M_{ij}$ specify how much each element $l_i$ of the input vector influences each element ${\hat{v}}_j$ of the predicted output vector (with ${\hat{v}}_j = \sum _{i} M_{ij}\cdot l_i$). Note that such a linear function is equivalent to a linear regression with multiple predictors and dependent variables. With this approach, we follow up on similar previous developments in the field of natural language processing and computer vision (Lazaridou et al. 2015).

As a training set for this regression, we employed the complete set of 7801 words for which we had both language-based vectors and vision-based vectors available. The weights are then estimated so that, on average, the difference between the predicted visual representation ${\hat{v}}$ and the observed visual representation *v* is minimized, with respect to a least-squares criterion. We used the DISSECT toolkit to train the function (Dinu et al. 2013).

On an intuitive level, this training set can be thought of as the repeated presentation of a visual scene paired with a corresponding linguistic stimulus, in which both the visual representation of the scene and the language-based representation of the word meaning are activated and associated (Zwaan and Madden 2005). The training process then captures the learning of the systematic relation between the two, if it exists.

While the cbow model provides one single vector representing the word meaning for each word, the VGG-F model provides an individual vector for each single image. Thus, as a consequence of our selection procedure, we have between 100 and 200 VGG-F vectors for each label. We can hence set up two different frameworks to estimate the mapping function: A prototype-based approach and an exemplar-based approach (following a classical distinction in concept research; Smith & Medin, 1981). In the prototype-based approach, we averaged all the 100-to-200 vectors for images with the same label to obtain a single visual representation for each word (Günther et al. 2020; Petilli et al. 2019). The resulting 7801 pairs of cbow vectors and prototype VGG-F vectors were then used to train the mapping function *m*. In the exemplar-based approach, we instead randomly selected 20 images for each label^1^ and trained the mapping function *m* directly on these individual images. Thus, we had 20 training items per word, resulting in a training set of 156,020 pairs. The language part of the model was identical for both approaches (cbow vectors corresponding to the image labels).

Since the dimensionality of the vectors will directly influence the number of free parameters for our mapping function, we reduced the original dimensionality of the visual representations in both training sets from $d = 4096$ to $d' = 300$ using Singular Value Decomposition (SVD; Martin & Berry, 2007), as implemented in the DISSECT toolkit (Dinu et al. 2013). This was done separately for the prototype-based and the exemplar-based approach. As indicated by a pre-test on the similarity structure within the visual representations, this has a negligible effect on the informativity of these vectors (see Günther, Petilli, & Marelli, 2020). As a result, *m* is estimated as a 400$\times$300-dimensional matrix, instead of a 400$\times$4096-dimensional matrix.

Once the mapping function is estimated, it can take any cbow vector as input—including, and especially, those outside the training set—to predict a visual representation for the corresponding concept (see the bottom part of Fig. 1).

### Identifying predictors of model performance

In the previous section, we described the language-to-vision mapping system implemented for this study. Notably, “concreteness” is not explicitly encoded in this model and therefore is not implemented as an a-priori component: the model has no “concreteness feature” that is assigned to some concepts and not to others. The model only knows whether a word has any visual experience associated to itself (in technical terms, whether a word serves as a label for a set of images).

However, not all language-based vectors are the same: By definition, they have different dimensional values, and populate different neighborhoods of the induced semantic space (see Martínez-Huertas, Jorge-Botana, Luzón, & Olmos, in press, for a conceptually similar distinction between a *specific dimensionality hypothesis* and a *semantic neighborhood hypothesis* for the mapping between language and grounded information). Based on these properties, we can identify factors that potentially influence how well a vision-based representation can be predicted from a language-based representation. On the one hand, there could just be inherent, fundamental differences between language-based representations for concrete and abstract concepts which emerge naturally during the training of the language-based model. Initial evidence for this assumption is provided by Hollis and Westbury (2016), who demonstrate that the dimensions of language-based distributional vectors contain concreteness information that can be extracted using adequate mathematical methods.

On the other hand, language-based representations for different concepts could inhabit fundamentally different areas of the semantic system. For example, assume that a speaker has newly learned the words *stallion* and *jealousy* without accompanying visual experience. Due to the way these words are used, *stallion* will have a language-based representation that is very similar to *horse*, *steed*, and *pony*. For all these neighbors of *stallion*, direct visual experience is available, which makes estimations of how a *stallion* looks like very easy (similar to a horse). The language-based representation for *jealousy* on the other side will be similar to *envy*, *hatred* and *resentment*, and thus concepts for which no visual experience is available. Irrespective of the concreteness of the word itself, it might be easier to extrapolate a visual representations for a word if its linguistic neighborhood contains more other visually-grounded concepts that can provide “a bridge to visual experience” and an orientation on how the concept probably looks like. This difference between the *relative position of visual neighbors* (which we will from now on refer to simply as *visual neighbors* for brevity) might thus influence model performance.

In the following empirical studies, we test our model by deriving model-predicted images for words which are all outside the model training set (i.e., for which the model has no visual experience available). These model-predicted images are then paired with random control images. If our model matches human intuitions on which image better fits the word meaning (i.e., if participants systematically prefer the model prediction over the control image), this will demonstrate that our linguistic and perceptual experience provides the necessary information to establish a link between the two (and that our model provides one possible, simple account on how this can be achieved). The model will be tested in different conditions which we expect to influence model performance: On the one hand, we test the model on both concrete and abstract words; on the other hand, we test it on words that do or do not have training items (i.e., words for which visual experience is available) in their immediate neighborhood. Since these two variables (*concreteness* and *visual neighbors*) are normally highly correlated, we apply item selection procedures to disentangle them (see the Methods sections of Experiments 1, 2, and 3). This will allow us (a) to evaluate if the model generally succeeds in predicting visual representations from language-based representations, (b) to test which factors influence its ability to do so, and (c) to examine potential limits of our approach and identify conditions it is not able to handle.

## Experiment 1 and 2

We tested whether the model predictions generally matches human intuitions in two experiments: In Experiment 1, we employed the prototype-based mapping model, and in Experiment 2 we employed the exemplar-based mapping model.

### Method

#### Participants

In Experiment 1, we initially collected data from 57 participants with normal or corrected-to-normal vision. Data from one non-native English speaking participant were excluded, as was data from three participants who gave a correct answer in less than eight of ten catch trials (see Materials) and were thus suspected to not have performed the task properly. Of the 53 remaining participants ($M_\mathrm{Age} = 37.9$ years, $\mathrm{SD}_\mathrm{Age} = 12.3$ years), 45 identified as female, seven as male, and one as genderfluid (in an open answer format). Participants received £2 for their participation in the study.

In Experiment 2, we initially collected data from 57 participants who did not participate in Experiment 1. Data from two non-native English speaking participants was excluded, as well as data from one participant based on the catch-trial criterion. Of the 54 remaining participants ($M_\mathrm{Age} = 33.6$ years, $\mathrm{SD}_\mathrm{Age} = 10.8$ years), 36 identified as female and 18 as male. Participants received £1.50 for their participation in the study.

Participants were recruited via the *Prolific* crowdsourcing platform (Palan and Schitter 2018). We would like to note here that, for an online crowdsourcing study, we had to exclude very few participants who did not reliably perform the task as expected, which is in line with previous studies highlighting the reliability and quality of workers on this platform (Peer et al. 2017).

#### Materials

*Words* The item set for this study was constructed by systematically manipulating the two independent variables discussed above: Concreteness and visual neighbors. The potential word candidates were taken from a large database containing concreteness ratings for 39,954 English words (Brysbaert et al. 2014). Only nouns were selected as potential candidates, since the ImageNet labels and therefore our training set only consisted of nouns. Further, we selected only medium-frequent words (SUBTLEX frequencies larger than 100 and smaller than 12,000; van Heuven et al. 2014) so that participants would most likely know the word, but not have extreme amounts of experience with it. The remaining 4845 words were classified as concrete and abstract through a median split.^2^

As potential items, we only considered the 3080 words that did *not* have an initial corresponding visual representation, that is, only words outside the training set. For each of these words, we determined its (linguistic) semantic neighborhood as its 50 nearest neighbors according to the cbow model (i.e., the most similar words in terms of cosine similarity), using the *LSAfun* package (Günther et al. 2015) for R (R Core Team 2017). We then determined for each word how many of these neighbors had a visual representation in the VGG-F space. For both concrete and abstract words, we randomly selected 23 of the words that didn’t have a single visually-represented word in their neighborhood (which we will refer to as the *far* condition, since the closest visual neighbor is far from the target word). In addition, we selected for both concrete and abstract items the 23 words with the most visual neighbors. However, there were substantial differences between these concrete and abstract words with respect to this variable: While the concrete words on average had 15.0 of such visual neighbors, the abstract words only had on average 6.7 visual neighbors. Thus, in natural language, concreteness and the number of visual neighbors are severely confounded—which highlights the importance of manipulating them separately as independent variables in the present experiments. In order to control for this confounding, we created two different groups of concrete words: (1) a set of words that matched the number of visual neighbors found for the abstract words (on average 6.3 such neighbors, the *near* condition), and (2) a set of concrete words with the most of such neighbors (the *maximum* condition). Item examples for the five conditions are presented in Table 1, and a complete list of the 115 resulting items (5 conditions à 23 items) is provided in Supplementary Material A (Experiment 1) and Supplementary Material B (Experiment 2). The selected words can be expected to be very familiar to the vast majority of participants: In the large-scale study by Brysbaert et al. (2019) on vocabulary knowledge, involving more than 220,000 participants, all words included here were known to between 93.0% and 100.0% of participants ($M = 99.2\%$).

**Table 1:**
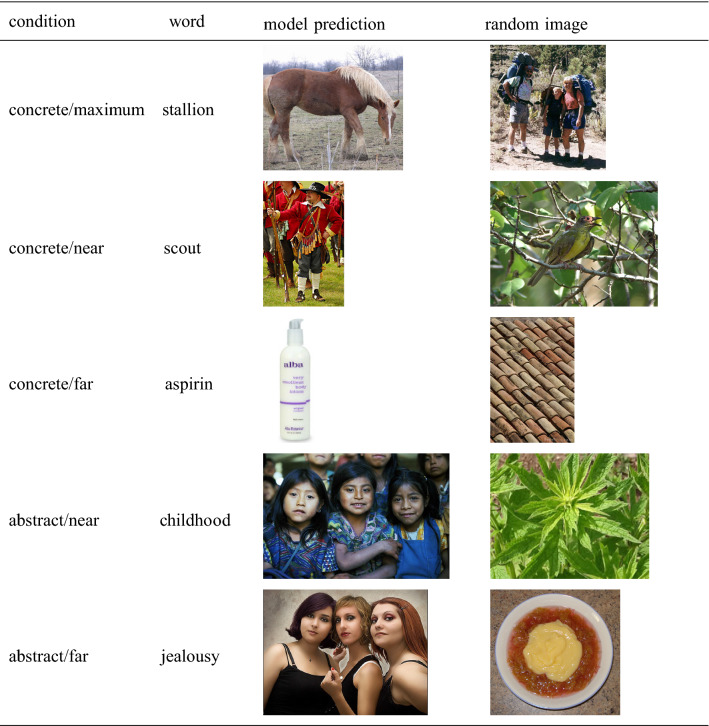
Examples for the triplets consisting of a word, the corresponding model prediction, and a random image (for the prototype-based model)

*Images* To generate visual predictions, we forwarded the cbow vectors for all the words in our item set to the trained mapping functions—both the prototype-based (Experiment 1) and the exemplar-based function (Experiment 2). Note that, in almost all cases, there is no specific image corresponding exactly to this predicted representation. In the exemplar-based approach (Experiment 2), we selected as predicted image the image whose VGG-F vector was most similar to the model prediction (in terms of cosine similarity). In the prototype-based approach (Experiment 1), we determined the prototype vector that was most similar to the model prediction and selected within the respective category the individual image that was most similar to the prototype vector.^3^

To set up an experimental baseline, we additionally selected for each word a random image that was not included in the image set. If participants systematically prefer the model prediction over the random image, we will conclude that the model predictions are in line with human intuition. Examples for the triplets consisting of a word, the corresponding model prediction, and a random image are displayed in Table 1. The complete item material for the experiments is provided in Supplementary Material A (Experiment 1) and Supplementary Material B (Experiment 2).

In addition, as catch trials to control that participants actually performed the task, we included 10 images for which visual representations were available, and whose referent is very clearly identifiable (such as *guitar* or *cow*). For these images, we manually selected one image that clearly represented the word referent and one that clearly did not.

#### Procedure

The study was administered as an online experiment, implemented using the *jsPsych* library (de Leeuw 2015; de Leeuw and Motz 2016). After giving informed consent to participate in the study, participants received their instructions. Each experimental trial consisted of the target word, printed in uppercase letters in the upper center of the screen. Below the target word, two images were presented next to one another: The model prediction and the random image. The position of these images (left and right) was randomized. Participants were instructed to select the picture that, according to their intuition, better represented the word meaning (by clicking on a button below the respective image).

The 125 items (115 experimental items and 10 catch trials) were presented to the participants in random order. After a response was given, the next item was presented, with no possibility of revising previous responses. We did not impose time limits, did not ask participants to answer as fast as possible, and encouraged them to look up words whose meaning they didn’t know in a dictionary. Most participants took between 5 and 10 minutes to complete the experiment.

### Results

We analyzed our data using a mixed-effect logistic regression (Jaeger 2008), using the packages *lme4* (Bates et al. 2015) and *lmerTest* (Kuznetsova et al. 2017) for R (R Core Team 2017). In each analysis, we fitted a model to predict whether or not participants chose the model-predicted image (we measure the *mapping performance* as proportion of participants who chose this model-predicted image over the random control image). As fixed-effect predictors in the mixed-effect model, we employed concreteness (concrete vs. abstract), visual neighbors (far vs. near vs. maximum), and their two-way interaction^4^. In addition, the model contained random intercepts for both participants and items. Although indicated by the experimental design (Barr et al. 2013), no by-participant random slopes for the fixed effects were included, since the model did not converge in that case (Bolker et al. 2009). To examine the significance of the fixed-effect terms, we then tested whether they could be removed from the model without a significant deterioration in model fit by employing likelihood-ratio tests.

**Fig. 2:**
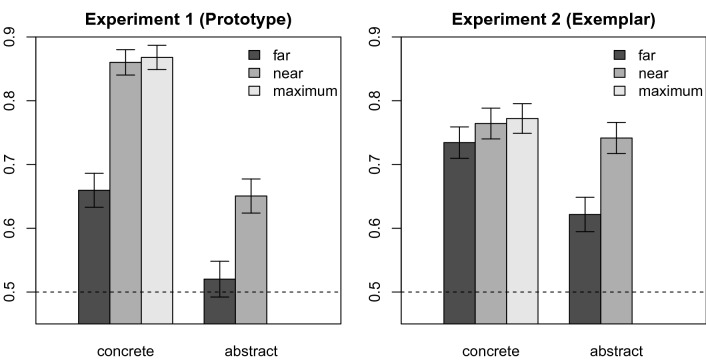
Mapping performance (means and 0.95-confidence intervals), defined as the proportion of trials where participants chose the model-predicted image, in Experiment 1 and Experiment 2, by concreteness and visual neighbors. The dashed line represents chance level

The mapping performance per condition is displayed in Fig. 2 (left panel: Experiment 1; right panel: Experiment 2). In Experiment 1 (based on the prototype model), a model without the two-way interaction term did not perform significantly worse than a model including this term ($X^2(1) = 1.17$, $p = 0.280$). However, from the resulting model, neither concreteness ($X^2(1) = 10.30$, $p < 0.001$) nor visual neighbors ($X^2(1) = 20.12$, $p < 0.001$) could be removed without significant deterioration of model fit. The parameters of the resulting two-main-effect-model are displayed in Table 2. As can be seen in Table 2, the intercept of this model is not significantly different from zero, which implies that performance in the condition serving as reference level—the abstract/far condition—is at chance level. However, all other conditions are significantly different from this condition, and thus by implication, performance in these conditions is above chance level.

**Table 2:** Final model parameters (after removal of all non-significant predictors) for the mixed effect logistic regression models predicting whether participants chose the mapping models’ predictions, for Experiment 1 (prototype model) and Experiment 2 (exemplar model). The abstract/far condition serves as the reference level, so all parameters have to be interpreted with respect to this condition (this includes the intercept, which codes this condition)

	Experiment 1			Experiment 2		
Parameter	$\beta$	*z*	*p*	$\beta$	*z*	*p*
Intercept	− 0.06	− 0.22	.825	1.38	9.54	$< 0.001$
Concreteness	1.09	3.29	0.001			
Neighborhood *near*	1.24	3.76	$< 0.001$			
Neighborhood *maximum*	1.73	3.86	$< 0.001$			
Conditional $R^2$ (complete model)	0.50			0.39		
marginal $R^2$ (fixed effects only)	0.15			*–*		

In Experiment 2 (based on the exemplar model), a model without the two-way interaction term did also not perform significantly worse than a model including this term ($X^2(1) = 0.41$, $p = 0.522$). However, also with respect to this two-main-effect-model, model fit did not deteriorate when concreteness was removed ($X^2(1) = 2.17$, $p = 0.140$), and also not when the parameter for visual neighbors was removed ($X^2(2) = 3.96$, $p = 0.138$). In fact, a model that included no fixed effect at all was neither improved significantly by including concreteness as the sole fixed-effect predictor ($X^2(1) = 3.54$, $p = 0.060$) nor by including visual neighbors ($X^2(2) = 5.33$, $p = 0.070$). The resulting final model thus only contained a significant intercept ($\beta = 1.38$, $z = 9.54$, $p < 0.001$; see Table 2). Importantly, the positive value of this intercept in the absence of any other effects indicates that mapping performance is well above chance level across *all* experimental conditions.

In Table 2, we report the model parameters for the final models after removal of all non-significant predictors, alongside their conditional and marginal $R^2$ (Bartoń 2018). A clear notable difference between the two Experiments, is that the model for Experiment 2 only contains an intercept which is significantly different from zero, indicating above-chance performance even the abstract/far condition. In Experiment 1, on the other hand, we observe above-chance performance in all conditions except for the abstract/far condition (see Fig. 2).

### Discussion

Results from both experiments show that the mapping model produces intuitively plausible predictions in most experimental conditions: Participants chose the model prediction over a random control image in the majority of cases. Critically, this is observed also for abstract words (the abstract/near condition in Experiment 1, and both abstract conditions in Experiment 2), and also for words without visual neighbors in their immediate semantic neighborhood (the concrete/far condition in Experiment 1 and both “far” conditions in Experiment 2). This implies (a) the language-based representations have to contain relevant information about the visual domain and (b) that our mapping model is able to capture this information and to successfully map it onto vision-based representations.

The only condition where the model predictions break down is for abstract words without nearby training neighbors (the abstract/far condition) in the prototype model. However, this does not imply that the relevant information is absent in the respective language-based vectors: In this case, also the exemplar model should break down in this condition, but this is not the case. Hence, since the language-based side of both models is the same, the breakdown in performance has to be attributed to the vision-based side of the model, with the prototypization procedure being the most likely candidate. We suspect that this is due to the loss of information in the averaging process, which at the same time results in a far smaller training set for the mapping function. Crucially however, from the performance of the exemplar model, we can conclude that mapping is possible across all conditions, given an appropriate parametrization of the model.

In addition, the results of Experiment 1 suggest that both the concreteness of words and the relative position of visual neighbors in their semantic neighborhood influence the model performance and thus the success of language-to-vision mapping. This is somewhat reflected (to a considerably smaller degree) also in Experiment 2, where model performance is at least numerically lower for abstract words without nearby training neighbors (see Fig. 2). These results have important implications. For our model architecture, there is no fundamental a priori difference between the different experimental conditions; all items equally lack visual experience, as there are no images available for them (rendering them virtually abstract from the model perspective). Nevertheless, we observe empirical differences between the conditions in the prototype model. We can therefore not simply equate “concreteness” with “availability of perceptual experience”—for some items, visual information can be more easily extrapolated from their language-based representations, which implies that there are fundamental differences between these representations.

However, at this point we need to point out a few issues with the material selection of Experiments 1 and 2, especially concerning the operationalization of the independent variables, that could have potentially influenced these results. These experiments were intended to allow a first assessment of our model architecture; accordingly, we opted for a simple experimental setup. First, we operationalized concreteness by dichotomizing the ratings by Brysbaert et al. (2014) through a median split. However, such an artificial dichotomization can distort the results (MacCallum et al. 2002): For example, the underlying continuous variable can still differ substantially between the conditions. And in fact, this was the case for our material: When considering concreteness as a continuous variable, the items in the “near” conditions had higher concreteness ratings than the “far” conditions (see Table 3). In addition to this point, our operationalization of the relative position of visual neighbors – whether or not the word has such visual neighbors amongst its fifty nearest neighbors—sets a quite arbitrary criterion which does not consider the complete neighborhood structure. We can think of a number of more nuanced and more comprehensive measures for the relative position of a word with respect to visually-grounded words (i.e., visual neighbors): The neighborhood rank of the nearest visual neighbor (i.e., when all words in the language-based semantic space are ordered by their cosine similarity to the target word, at which position does the first visual neighbor appears in this list) and its similarity to the target word, as well the mean neighborhood rank of all 7801 training items (i.e., visual neighbors) and their mean similarity to the target word. When considering all these variables, the concrete words consistently had better values than the abstract words (see Table 3). Thus, in Experiment 1 and 2, we were not as successful in operationalizing and in disentangling the two variables as it would be desired. This residual entanglement between concreteness and neighborhood could especially affect the performance of the prototype model due to its smaller training set. In order to address these issues, we set up a third experiment, implementing a new item selection procedure and also employing a considerably larger item set.

**Table 3:** Concreteness ratings and the relative position of visual neighbors by experimental condition

Condition	Concreteness	n. train. rank	n. train. sim.	all train. rank	all train. sim.
Abstract/far	1.79	275.2	0.340	162338.2	0.056
Abstract/near	2.69	6.8	0.480	161220.1	0.067
Concrete/far	3.99	135.2	0 .386	161260.2	0.068
Concrete/near	4.40	7.0	0.502	140618.7	0.085
Concrete/maximum	4.70	3.3	0.546	128624.6	.095

n., nearest; train., training items (i.e., visual neighbors); sim., similarity

## Experiment 3

While Experiment 1 and 2 demonstrated that language-to-vision mapping is possible across several (more or less favorable) conditions, the influence of concreteness and visual neighbors cannot be reliably determined from these experiments, due to the problems with disentangling wither variable from the other that we discussed in the previous section. Experiment 3 was designed to address these problems. First, we applied a more comprehensive measure for visual neighbors that considers the entire semantic space (in terms of the arrangement of *all* training items relative to the target word), instead of only the immediate neighborhood. For simplicity, we will still maintain the term *visual neighbors* (short for *relative position of visual neighbors*) to refer to this variable, although this “neighborhood” is now defined as the entire lexicon. Second, we considered both concreteness and visual neighbors as the continuous variables they are, instead of introducing an artificial dichotomization MacCallum et al. (2002).

Since the purpose of Experiment 3 was to thoroughly investigate the influence of concreteness and visual neighbors on model performance, we employed only the prototype model in Experiment 3, where the effect of both variables was more prominent as compared to the exemplar model with its relatively even performance across conditions.

### Method

#### Participants

We initially collected data from 164 participants with normal or corrected-to-normal vision. Data from one additional participant who did not provide information on their native language was excluded, as well as data from two participants who gave the correct answer in fewer than eight of the ten catch trials. Of these participants ($M_\mathrm{Age} = 34.7$ years, $\mathrm{SD}_\mathrm{Age} = 12.6$ years), 113 identified as female, 49 as male, one as non-binary, and one as two-spirited (in an open answer format). Participants received £1.5 for their participation in the study. None of the participants had taken part in Experiment 1 or Experiment 2.

#### Material

*Words* As in Experiments 1 and 2, we systematically manipulated the two independent variables: Concreteness and visual neighbors. The potential target words were the same 3080 words described in Experiments 1 and 2. We obtained the mean concreteness rating for each of these words from the Brysbaert et al. (2014) dataset.

Concerning the operationalization of neighborhood structure, of the four variables presented in Table 3, we opted to employ the mean similarity of the target word with all training words as the variable operationalizing the relative position of the visual neighbors, since (a) it considers the similarity between the target word and the entire training set (i.e., all words for which a visual representation is available), and (b) it has the overall highest correlations to the other three variables ($r = - 0.80$ with mean training item neighborhood rank, $r = .42$ with nearest training item similarity, and $r = - 0.17$ with nearest training item neighborhood rank). In formal terms, this measure is defined as $\frac{1}{n}\sum _{i=1}^{n}\cos {(w,t_i)}$, with *w* being the target word and $t_i$ being all $n = 7801$ training items.

**Fig. 3:**
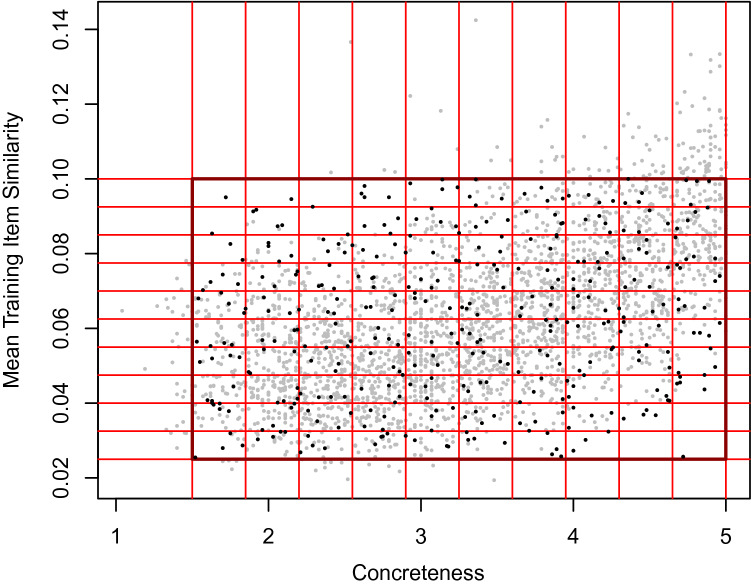
The relation between concreteness and visual neighbors, as measured by mean training item similarity. The red lines represent the bins considered for item selection; from each resulting cell within the boundaries of the black rectangle, we sampled four random items (if available), which are marked in black. From cells with less than four items, we selected all available items

However, as can be seen in Fig. 3, mean training item similarity and concreteness ratings are substantially correlated ($r = 0.53$). Note again—as mentioned for Experiment 1 and 2—that this is an interesting result in itself, as it demonstrates that concrete and abstract words inhabit very different regions of semantic memory, even when this latter is modeled on the basis of linguistic data only (i.e., without appeal to perception). Concrete words are, on average, much closer to words for which visual experience is available. In addition, this further highlights the importance of disentangling these variables.

In order to select items representing the range of both variables while also de-correlating them (see Günther and Marelli, 2020), we segmented the range of both of them into 10 bins, resulting in 100 cells (see Fig. 3). We then selected four random items from each cell. However, due to the high correlation between both variables, we cannot simultaneously cover the whole range of both values and select the full amount of items for each combination of variable values (see Fig. 3).We aimed to establish a compromise between these two objectives of the item selection procedure, thus selecting 371 items for which the variables were weakly correlated ($r = 0.16$). Again, the selected words can be expected to be very familiar to the vast majority of participants: In the study by Brysbaert et al. (2019), all words included here were known to between 86.3% and 100.0% of participants ($M = 99.1\%$; the only items with values under 90% being *tong* at 86.3% and *shaman* at 88.6%).

*Images* For each of the 371 words in our item material, we obtained images predicted by the prototype model as well as random images exactly as described in Experiment 1.^5^ To check whether participants actually performed the task, we further employed the same 10 “catch trials” described for Experiment 1. The complete item material for Experiment 3 is provided in Supplementary Material C.

#### Procedure

In order to keep the experiment short, we randomly split the 371 items into three different lists, two containing 124 items and one containing 123 items. The same 10 “catch trials” were included in each list, and each participant was only presented with exactly one list (54–56 participants per list). We thus collected the same number of data points per item as in Experiment 1 and 2.

Apart from this change due to the larger item set, the procedure of Experiment 3 was identical to Experiment 1.

### Results and discussion

As in Experiments 1 and 2, we again fitted a mixed-effects model predicting participants’ responses from the predictors concreteness and visual neighbors (as measured by mean training item similarity), as well as their two-way interaction, while the model also contained random intercepts for both participants and items to account for participant-specific variability and for item-specific idiosyncrasies (Baayen et al. 2008). In contrast to Experiment 1 and 2, concreteness and visual neighbors were entered as continuous instead of categorical variables. The minimum value of both variables was set to zero (by subtracting the original minimum value from all values), so that the model’s intercept can be interpreted as the predicted mapping performance for the item with the lowest concreteness value and the lowest mean training item similarity.

**Fig. 4:**
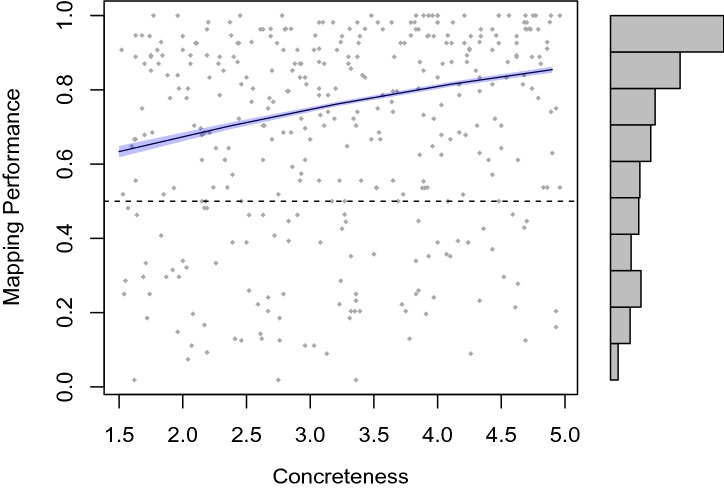
Mapping performance (i.e., proportion of trials where participants chose the mapping model-predicted image—predicted value with 0.95-confidence interval band) as a function of concreteness in Experiment 3

The interaction parameter could be removed from the model without a significant change in model fit ($\chi ^2(1) = 1.81$, $p = 0.179$), and in a following step, the same was the case for visual neighbors ($\chi ^2(1) = 3.28$, $p = 0.070$), but not for concreteness ($\chi ^2(1) = 16.17$, $p = < 0.001$). In the resulting model the fixed effect for concreteness was significant ($b = 0.36$, $z = 3.80$, $p < 0.001$). This concreteness effect with 0.95-confidence interval band Fox (2003) is displayed in Fig. 4; as can be seen, the mapping performance is consistently higher than chance level. This is confirmed by the significant intercept of the model ($b = 0.55$, $z = 2.92$, $p = 0.004$)^6^. For the final model including only the concreteness predictor, we obtained a conditional $R^2 = 0.48$ and a marginal $R^2 = 0.02$ (Bartoń 2018).

Experiment 3 yields two main results: first, and most importantly, we observe that the mapping performance is consistently well above chance level, even for the most abstract words in the prototype model. Second, we observe that the mapping performance increases for more concrete words, while no significant impact is found for the relative position of visual neighbors.

## General discussion

In the present article, we addressed the question of how words encountered in purely linguistic contexts can be grounded in perceptual experience, by investigating whether we can exploit our existing linguistic and visual experience to predict visual representations for words whose referents we have never seen before. To this end, we employed computational models of language-based and vision-based representations, and trained a mapping function that takes as input a language-based representation and predicts a corresponding vision-based representation. We then tested the model’s predictions against human judgments, in order to investigate (a) whether the model, in general, produces predictions that are in line with human intuition, and (b) which factors influence model performance. Our three experiments yielded the following results:

First, the model is able to predict visual representations that are in line with human conditions across the board, even for the most abstract words that are far from any items the model was trained on (i.e., words for which direct visual experience is available). This has substantial implications for theories on the grounding of abstract words, as it shows that we can extrapolate from our available experience to predict possible visual representations that we haven’t yet acquired. Critically, in this respect, the difference between concrete and abstract words is not a qualitative, but instead a graded one, with model performance being lower for abstract words, but not breaking down for those items.

This directly brings us to the second main result: As demonstrated by Experiment 3, the prototype model performance depends on word concreteness, not on the relative position of visual neighbors. Thus, the model does not work better just because a target word is close to other words with established links to visual experience. Instead, concreteness—an inherent property of the word itself that needs to be captured in its dimensional values—influences model performance.

Of course, there might be other predictors of model performance that we did not consider in out present study. For example, Harpaintner et al. (2018) identify different subgroups of abstract concepts, which are related to sensorimotor information (visual: *observation*, gustatory: *appetite*, interoceptive: *pain*, etc.), social constellations (*advice*), emotions/internal states (*emotion*), or verbal associations (*revolution*), with processing differences between these groups (Harpaintner et al. 2020). Since our model operates on visual information, it stands to reason that model performance might be higher for words related to sensorimotor information and especially visual information as compared to the other subgroups. This potential influence can be investigated in future studies.

### Differences between the experiments

In Experiment 1 (employing the prototype model), we observe an effect of concreteness and visual neighbors; however, more carefully operationalizing both variables, Experiment 3 (also employing the prototype model) only yields an effect of concreteness. Still, this poses a difference with respect to Experiment 2 (employing the exemplar model), where we observe no effect of these variables, and model performance is consistently good across all conditions.

The initial purpose of testing both the prototype and the exemplar model was to ensure the robustness of our model architecture against different possible parametrizations in the model, which was confirmed by the empirical results. We did not have a-priori hypotheses concerning potential performance differences between these models; therefore, any interpretation remains speculative.

One such interpretation relies on the structural difference between the two models: While the exemplar model is trained on multiple individual images per word, the prototype model is trained on an average visual representation. Therefore, in the prototype model information is averaged (and noise is reduced) at the output level, while in the exemplar model such averaging of information needs to take place within the linear function mapping one domain onto the other. Since this linear function has a very large number of parameters, the training process for the exemplar model might be able to pick up on more nuanced low-level information than the prototype model, while the prototype model might be more apt to pick up on high-level information shared between the individual images in a category. This might lead to a higher performance of the exemplar model in the “low-performance” categories (the abstract and far conditions), since it has the possibility to recover some idiosyncratic visual information from a few images, and to a higher performance of the prototype model in the “high-performance” categories (the concrete and near conditions), since it can recover very robust visual information based on general trends in the training data.

### Encoding perceptually related information in distributional vectors

Critically, as pointed out already, our language-based model gets no explicit information about concreteness whatsoever. And still, in order to explain our results, some form of perceptually-related information needs to be reflected in the language-based distributional vectors: The mapping model can only produce intuitively plausible predictions if its language-based input contains the necessary information to pattern with the actual perceptual data. However, the language-based model was never trained to perform perceptually-related tasks. Therefore, the presence of any “visual” information in the language-based model has to naturally emerge from linguistic distributions. How can this be the case?

First, the implication that concreteness information is encoded in distributional vectors is in line with results by Hollis and Westbury (2016), who were able to extract a dimension distinctly correlated to the Brysbaert et al. (2014) concreteness ratings by applying a principal component analysis to a *word2vec* model (Mikolov et al. 2013) similar to the one employed here. The possibility to extract this piece of information via mathematical operations implies that it is encoded in the system in the first place.

Second, a whole line of research by Louwerse and colleagues has repeatedly demonstrated that perceptually-related information is encoded in statistical patterns of language use: For example, Louwerse and Zwaan (2009) find that the similarities between distributional vectors corresponding to cities reflect their actual spatial arrangement in the physical world, and Louwerse and Connell (2011) demonstrate that modality-specific words can be categorized according to their modality based on their frequency of co-occurrence in language. This is because language is not at all independent from the physical world we live in, but instead often used to communicate about this very world (Louwerse 2011). This leads to statistical redundancies between the structure of the physical, directly-perceivable world on the one hand, and the structure of language on the other hand, so that relations between words tend to reflect the relations between their referents (Johns and Jones 2012; Louwerse 2011; Rinaldi and Marelli 2020)—which in turn influences the training of distributional semantic models and the dimensional values of the resulting distributional vectors. The possibility of encoding grounded information through language usage makes it possible for our model to work, by capturing this information and systematically linking it to information from the visual domain.

### On the mapping model

In our three experiments, the model predictions were non-attested vision-based representations; and in each experiment, we then selected as the most similar image to represent it (see the respective Methods sections). Thus, the experimental material of our present study was restricted to *existing* images. Consequently, one could argue that our model is not able to capture examples such as *zebra = horse with stripes*, which would correspond to *new* visual experience if we never encountered a zebra before. However, it is important to note that this shortcoming is a methodological rather than a fundamental one: The model predicts vision-based *representations*, not images. Thus, the model prediction could indeed be the visual representation corresponding to actual images of zebras drawn from sketch, if we forward them as new items to the VGG-F model.

We did not yet extend the model architecture to produce actual images that can be used as experimental material due to a uniqueness problem: Due to the excessive transformations and loss of information in the VGG-F model’s architecture, we cannot simply “reverse” the model to generate a new RGB pixel arrangement (i.e., a new image) from any vision-based representation. However, dedicated work in the field of computer vision has developed sophisticated methods to generate images from vector-based representations (e.g. van den Oord et al., 2016; Yan, Yang, Sohn, & Lee, 2016), especially with the arrival of Generative Adversarial Networks (GANs; Radford, Metz, & Chintala, 2015; Zhang et al., 2017). We leave the further exploration and adaption of such methods for language-to-vision mapping to future research.

However, a genuine argument concerning the model architecture is that, while both the language-based *cbow* model (Mikolov et al. 2013) and the vision-based VGG-F model (Chatfield et al. 2014) are reasonably sophisticated, the mapping part of the architecture is almost awkwardly simple: A standard linear regression (Lazaridou et al. 2015). We are certain that more sophisticated and optimized mapping functions, for example based on deep neural network architectures that can deal with non-linear relations, would lead to an improvement in model performance. However, already our extremely simple mapping function is able to detect the relevant dimensions and successfully translate them from the language-based to the vision-based space. The simplicity of the mapping approach, rather than a shortcoming of the model, thus represents one of its main strengths: it shows that sophisticated, overly optimized methods are not needed in order to capture the role of language in the grounding of abstract concepts. The extraction of immediate, linear statistical association in experience is enough to be in line with human intuitions.

### On theories on the grounding of abstract concepts

The present work is informative about the debate on abstract concepts currently characterizing the embodied cognition literature, and provides a unique computational perspective on the issue. In the present section, we first categorize our model using the terminology proposed in the comprehensive review by Borghi et al. (2017), before discussing its relations and compatibility with the other approaches reviewed by Borghi et al. (2017).

#### Classifying our model

Borghi et al. (2017) classify existing accounts concerning the grounding of abstract concepts by employing a detailed terminology. First, they specify if a theory assumes a fundamental, qualitative difference between abstract and concrete concepts (as in the Words as Social Tools (WAT) account, Borghi & Binkofski, 2014), or if they are seen as in principle the same (as in the motor theory; Glenberg, Sato, & Cattaneo, 2008; Glenberg, Sato, Cattaneo, Riggio, et al.,^2008^). Second, Borghi et al. (2017) distinguish between theories that allow the possibility of multiple representations for a single concept (linguistic and sensorimotor, but also emotional or social, as in the representational pluralism view, Dove, 2009, 2011; hybrid models of conceptual cognition, Kiefer & Pulvermüller, 2012; Kuhnke, Kiefer, & Hartwigsen, 2020; Patterson & Ralph, 2016; Popp, Trumpp, & Kiefer, 2019; or in the affective embodiment account; Kousta et al., 2011; Vigliocco et al., 2013) and theories that assume only a single type of representation (sensorimotor) for all concepts (as in the conceptual metaphor theory; Lakoff & Johnson, 1980, 2008). Along similar lines, they also distinguish between theories adopting a strong embodiment view (which assume that only the sensorimotor system is activated during conceptual processing, such as the motor theory or conceptual metaphor theory) and theories adopting a weak embodiment view (which assume that both sensorimotor and linguistic systems are activated, as in the language and situated simulation (LASS) theory; Barsalou et al., 2008). Finally, Borghi et al. (2017) employ the open-ended, qualitative category “role of acquisition” to describe how different theories account for the specific mechanisms of how abstract concepts are learned and grounded by speakers (with the majority of theories leaving this unspecified).

First, does our model assume a fundamental difference between abstract and concrete concepts? As described in the Introduction, on the surface level, it could be assumed that concrete concepts are those for which visual experience is available. However, as demonstrated in our experiments, the complete picture is more complex than that: It is more adequate to say that, in terms of our model, a word is more concrete the more successfully it can be mapped onto visual experience (representing sensorimotor experience in general), which does not necessarily require the availability of direct experience. Thus, our approach assumes the existence of a concreteness dimension, and characterizes concreteness as a graded variable rather than assuming a clear-cut difference between abstract and concrete concepts (compare, for example, Wiemer-Hastings and Xu 2005).

Second, the current version of the model presented here clearly assumes multiple representations for each concept; language-based representations are derived using the cbow model, while vision-based representations are derived using the VGG-F model, and the whole point of the model is to establish a mapping between these different representations. However, this representational pluralism is a working hypothesis of the current implementation of our model rather than a fundamental theoretical assumption: In principle, the model architecture can be extended to take multimodal representations which integrate language-based and vision-based information as input or output (see Andrews, Vigliocco, & Vinson, 2009; Bruni, Tran, & Baroni, 2014; Lazaridou et al., 2015, 2017). In the present study, we made clear distinction between the two representations in order to test our hypotheses as directly as possible (see Günther, Petilli, & Marelli, 2020, for a discussion on this methodological approach when evaluating multimodal models).

Concerning the question of strong versus weak embodiment, the model proposed here makes no specific assumption about which cognitive systems or even brain areas are engaged in processing; it is conceived as a learning-oriented model about how grounding can be achieved, and not as a processing model. However, the multiple-representation view appears to imply a weak embodiment perspective: According to Borghi et al. (2017), all multiple-representation approaches are classified as adapting a weak embodiment view.

Crucially however, due to its characterization of our model as a learning-oriented model, the present proposal excels in specifying the role of acquisition: We present a fully implemented and computational account of the grounding of abstract words, which is entirely rooted in (an approximation of) experience. Thus, while the idea that abstract words (or, more generally, words whose referents were not directly experienced) are mapped onto another domain for grounding is certainly not a new one, we provide a precise and fully data-driven model of *how* this can be achieved. The language-based model is trained on large quantities of natural text, which simulates learning from language experience; the vision-based model is trained to classify large numbers of images, representing visual experience; and the mapping model is trained to predict one from the other, based on experienced co-occurrences between linguistic stimuli and visual stimuli (see Zwaan & Madden, 2005). On a theoretical level, this design of the model is in line with Hebbian learning mechanisms, which neuropsychological studies have shown to play an important role for the mapping between language and the sensorimotor system (Hoenig et al., 2011; Kiefer et al., 2007; Pulvermüller 2005; Trumpp & Kiefer, 2018). Thus, acquisition is the core component of our model, and its role is explicitly and formally described.

#### Relation to other approaches

The core idea of our model is quite similar in spirit to the conceptual metaphor theory (Lakoff and Johnson 1980, 2008): we assume that one type of representation (concepts without visual experience) is mapped onto another type of representation (vision-based representations) in order to ground it and make it understandable. However, there are also notable differences between our model and the original metaphor approach: We do not assume a mapping that is strictly based on specific semantic domains, such as a structural mapping from concepts related to “time” onto concepts related to “space”; rather, the mapping takes place across the entire language-based and vision-based system, and if such structural mappings exist, they would naturally emerge due to the properties of these systems. Further, we do not need to rely on researcher intuition to hand-code specific types of mapping; rather, the mapping function learns these relations from experience in a purely data-driven way. However, this certainly does not prevent or speak against such specific structural mappings, which can manifest as special cases of a more general mapping system that is based on general-purpose statistical learning approaches.

Furthermore, our model is very much in line with the assumptions underlying the LASS theory (Barsalou et al. 2008; Wilson-Mendenhall et al. 2013) that both concrete and abstract concepts can activate a mixture of linguistic and sensorimotor representations, with different distributions among the two depending on the specific concept. We explicitly refer to *assumptions*, since the LASS theory itself is first and foremost a processing model concerned with the relative timing of this activation (Barsalou et al. 2008) which does not specify the role of acquisition (Borghi et al. 2017). On the other hand, while our model is focused on way concepts are represented and acquired, it is completely agnostic when it comes to processing. This difference in focus between the two approaches brings with it the appealing possibility of combining them into a unified account of the acquisition (our model) and processing (LASS) of abstract concepts.

**Fig. 5:**
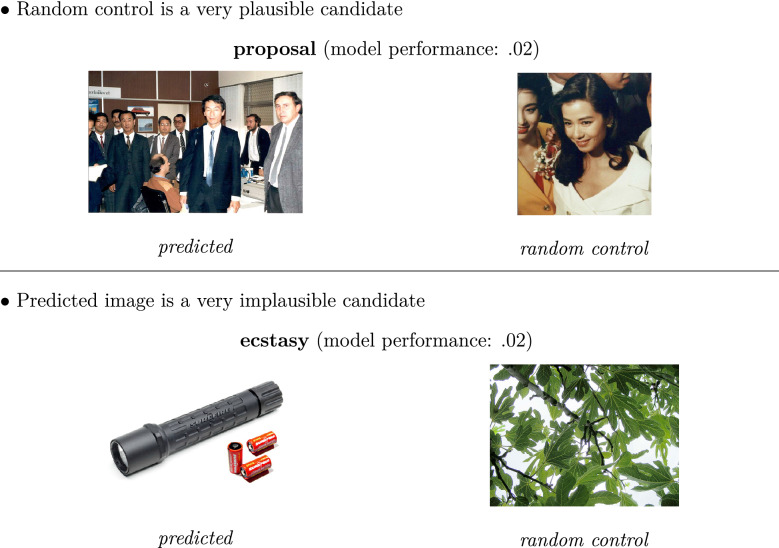
Items with low model performance in Experiment 3, with two intuitive explanations for the low performance

Determining whether our model is in line with a representational pluralism view, such as proposed by (Dove 2009, 2011), is not straightforward: Once it is implemented and trained, there surely are concepts for which the mapping apparently does not work (as suggested by the items below the dotted line in Fig. 4). Some of these low-performance cases might result from our experimental methodology of using random control images, which in principle can sometimes also be a good fit for the word by sheer chance (for example *proposal*, with a model performance of 0.02 in Experiment 3; see Fig. 5). However, in other cases the predicted image is just not a plausible candidate (for example *ecstasy*, with a model performance of .07 in Experiment 3; see Fig. 5). In the latter cases, we cannot assume that our model successfully links the representations of these concepts to sensorimotor experience. Therefore, in the absence of contrary evidence, it is plausible to assume in our framework that such concepts are represented in a purely language-based format. However, this all depends on the training and hence the experience available to the model, as well as the actual model architecture: If new experience is provided, by introducing new text to the language-based model or new images (maybe even with new labels) to the vision-based model, or if the model architecture is further optimized, the performance for items for which mapping previously failed could suddenly improve dramatically. Thus, the fairest assessment is that our model in its current implementation is *empirically*, but not necessarily by definition, in line with a representational pluralism view.

Interestingly, an unexpected finding of our study is that our model seems to align with the Words as Social Tools (WAT) theory (Borghi and Binkofski 2014)—also on an empirical level, rather than by definition. Upon inspecting the experimental material of our study, we noticed that the model shows a tendency to predict more and more images of humans, the more abstract the input words are (and, as indicated by our results, participants prefer to select these images over random controls). An example for this is already illustrated in Table 1.^7^ We want to emphasize that we did not expect this finding; this model behavior emerged naturally from the training data. However, it is surprisingly plausible in light of the WAT theory: Abstract words can only have a meaning in the presence of a conscious mind, which in our experience coincides with a human mind. Without a mind perceiving and interpreting the world, there is no such thing as *difference*, *value*, or *jealousy*. Therefore, it seems adequate for the model to predict that a word has something to do with humans or social interactions in cases missing information that can be mapped onto a clear perceptual referent.

To conclude this section, our model is agnostic towards the Affective Embodiment Account (Kousta et al. 2011; Vigliocco et al. 2013) or motor theories of grounding (Glenberg et al. 2008, 2008), since these approaches assume grounding in domains (emotion or action) that are not implemented in this version of the model. We also want to emphasize explicitly that we and our model do *not* claim that vision is the only relevant domain for grounding. Indeed, we acknowledge that recent works highlighted the role played by perceptual modality ratings across the five senses in predicting human performance (see for example Lynott et al. 2019). Instead, we focused the vision domain as *one instance* of sensorimotor experience (for which the necessary technical means to represent it are conveniently available at this point in time). However, with adequate data from other domains (for example, fMRI data representing cerebral activation, Mitchell et al. 2008; EMG data measuring peripheral motor activity, Vergallito et al. 2019; or affective representations collected from emoji usage in texts, Rotaru et al. 2019), our general model architecture could also be extended to other domains.

### Conclusion

How we can ground concepts in sensorimotor experience when this very experience is missing is one of the major challenges for theories of conceptual and semantic representation, and especially crucial for the tenability of grounded cognition accounts (Borghi et al. 2017). In the present article we demonstrate that, once we have available a sufficiently large amount of experience, we can use it as a scaffolding tool to draw inferences and “build bridges” from language-based to vision-based, grounded information. We can capture and learn structural (statistical) relations between the way we use language and the perceptual experience we have of the world (Johns & Jones, 2012; Louwerse, 2011; Zwaan & Madden, 2005), and then productively apply what we learned to extend our conceptual system beyond what we have already encountered (Günther et al. 2020).

## Electronic supplementary material

Below is the link to the electronic supplementary material.

Supplementary material 1 (pdf 27894 KB)

Supplementary material 2 (pdf 24305 KB)

Supplementary material 3 (pdf 79133 KB)

## Footnotes

1 We could not select the complete set of 100–200 images due to computational limitations in the subsequent dimensionality reduction step.

2 For Experiment 1 and 2, both predictors—concreteness and visual neighbors—were classified into categorical variables. Potential implications of this approach are discussed in the Discussion section, and in Experiment 3, no such categorization is applied.

3 Similarities between the prototype vectors and the selected images were very high (all between 0.76 and 0.94, $M = 0.86$), and did not differ between experimental conditions. When included in the data analysis reported below as a covariate, these similarities did not predict model performance ($t = 0.72$, $p = 0.47$).

4 Note that no abstract/maximum condition exists, so the respective coefficient was dropped from the model matrix. The results of all analyses reported here remain the same when excluding the *maximum* level from the analysis to achieve a balanced 2$\times$2-design.

5 Again, similarities between the prototype vectors and the selected images were very high (all between 0.75 and 0.94, $M = 0.86$). There was no correlation between these similarities and visual neighbors ($r = 0.00$), and a very small correlation with concreteness ($r = 0.09$). When included in the data analysis reported below as a covariate, these similarities had an additional positive effect on model performance ($t = 2.63$, $p = 0.01$), which did however not affect the pattern of results for any other variable.

6 A logit value of $b = 0.55$ corresponds to a probability of $P = 0.63$, see Fig. 4

7 The *jealousy* and *childhood* examples here are especially fitting, and while a number of these cases exist, there are also a several pictures of humans that, in our opinion, have no evident relation to the meaning of the word that predicted them.
