# Supplementary material for: Images of the unseen: extrapolating visual representations for abstract and concrete words in a data-driven computational model
Source: Psychol Res. 2020 Nov 12;86(8):2512–32. doi: 10.1007/s00426-020-01429-7 (PMC9674750; doi:10.1007/s00426-020-01429-7)
Supplement: Supplementary file 1 — Supplementary material 1 (pdf 27894 KB) [file 426_2020_1429_MOESM1_ESM.pdf]

# Experiment 1 (prototype model)

**freighter**

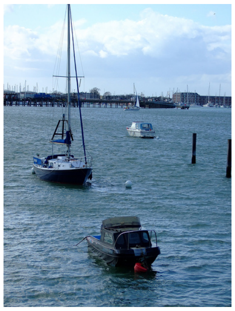

*predicted*

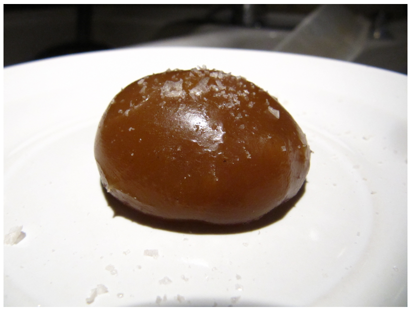

*random control*

**pocket**

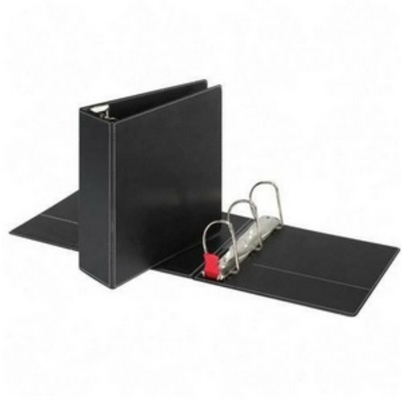

*predicted*

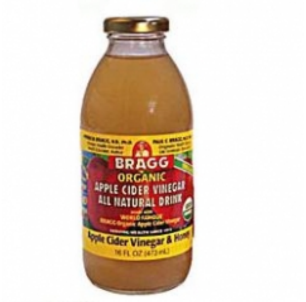


*random control*

**uncle**

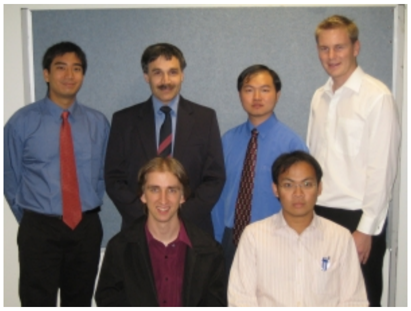

*predicted*

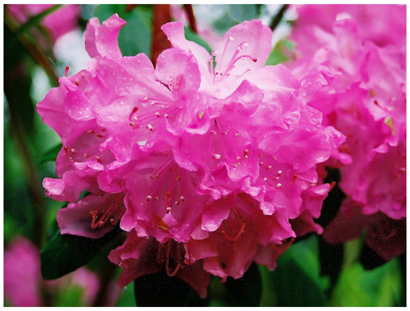

*random control*

**performer**

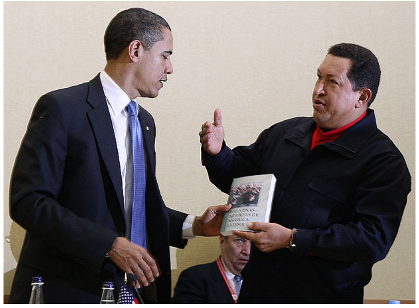

*predicted*

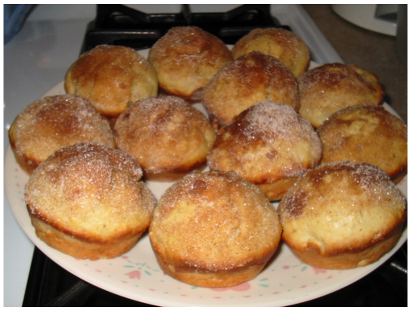

*random control*

**whiskey**

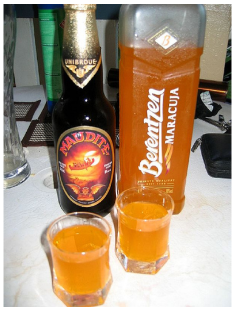


*predicted*

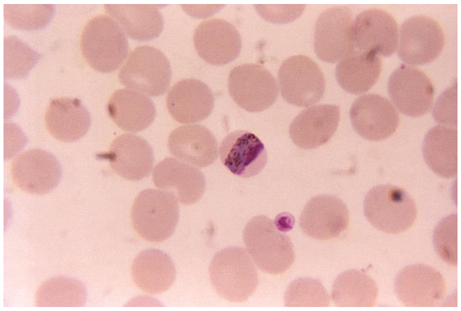

*random control*

**hamburger**

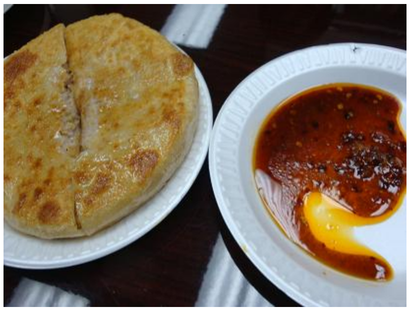

*predicted*

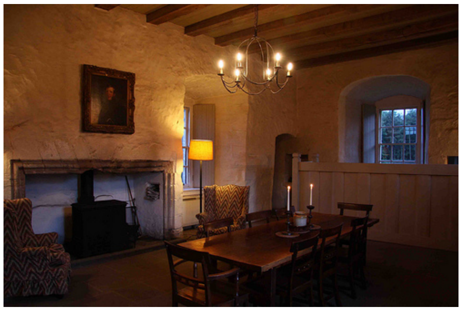

*random control*

**barb**

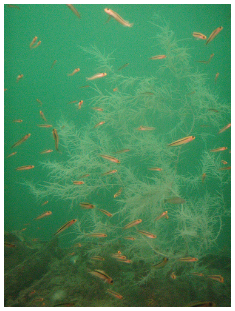

*predicted*

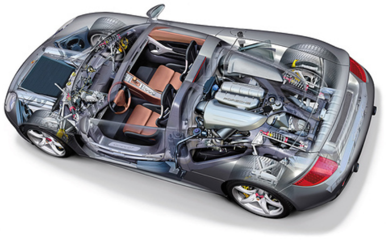

*random control*

**cushion**

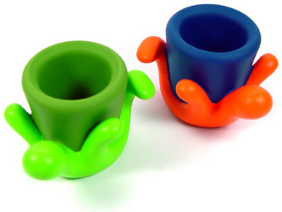

*predicted*

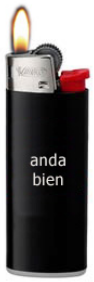


*random control*

**spear**

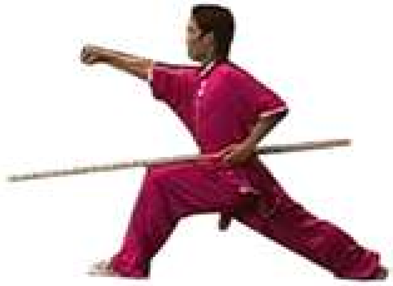

*predicted*

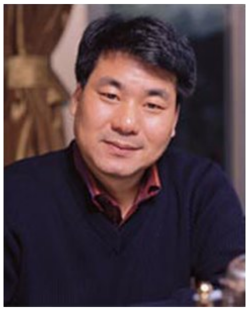

*random control*

## engineer

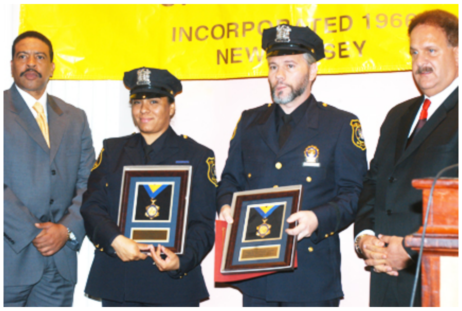

*predicted*

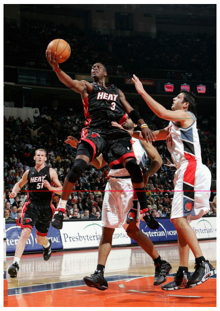

*random control*

## cheesecake

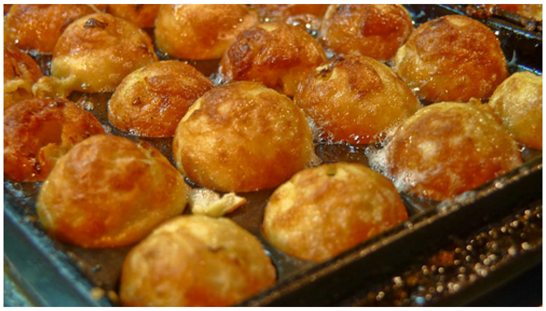

*predicted*

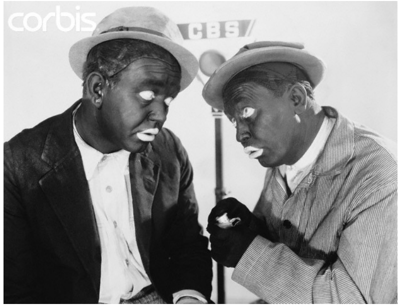


*random control*

## ribbon

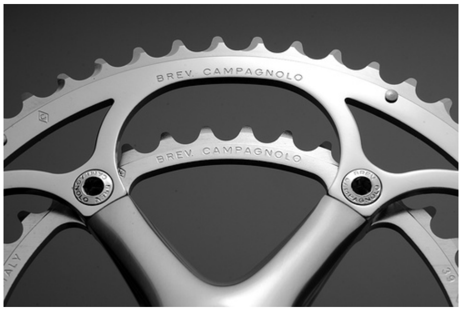


*predicted*

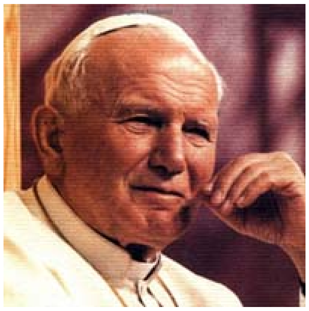

*random control*

**cockroach**

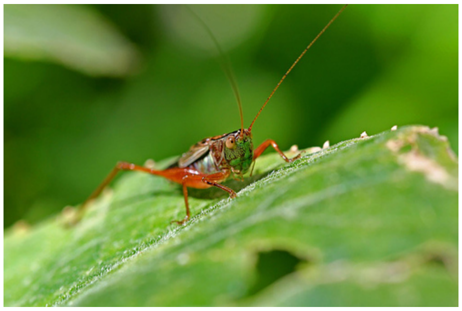

*predicted*

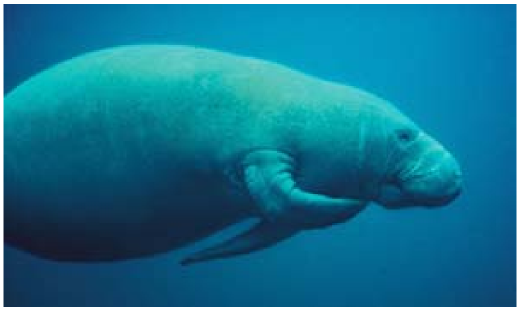

*random control*

**stallion**

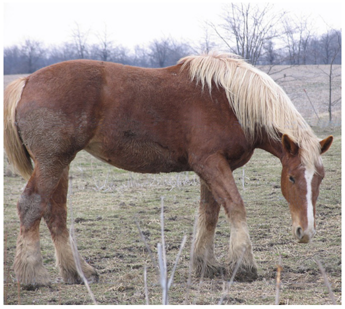

*predicted*

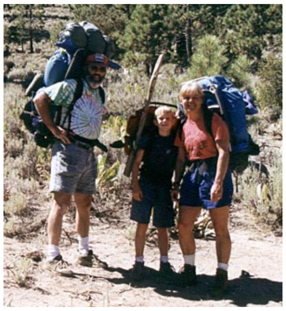

*random control*

**crank**

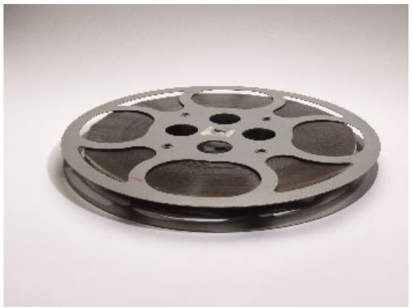

*predicted*

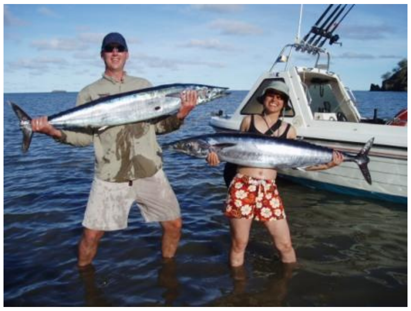

*random control*

**cloth**

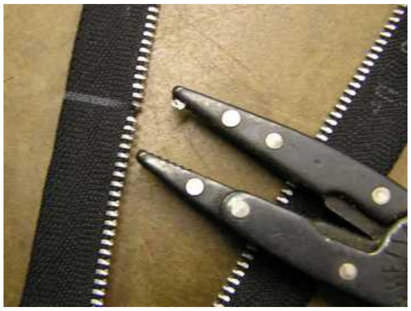

*predicted*

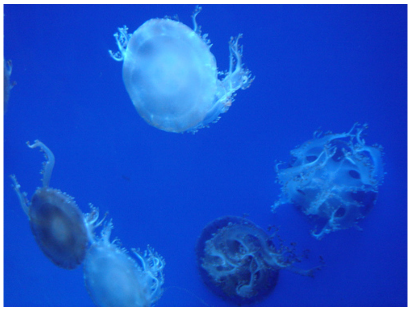

*random control*

**housewife**

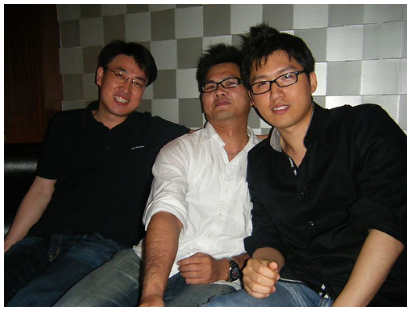

*predicted*

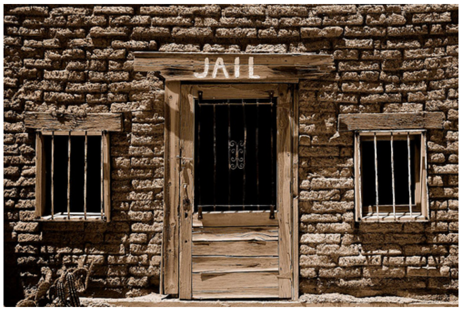


*random control*

**ivy**

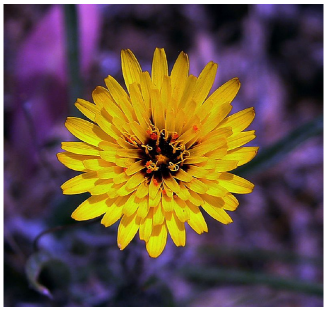

*predicted*

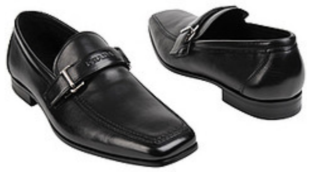

*random control*

**tomb**

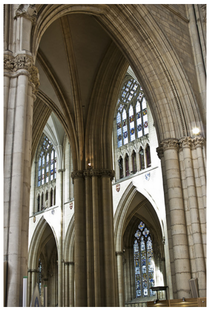

*predicted*

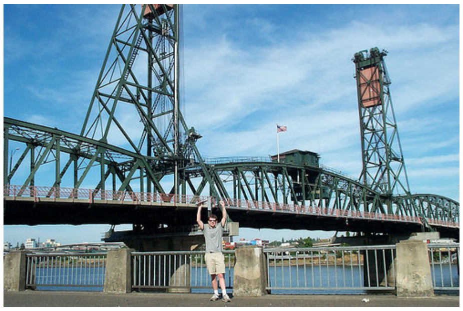

*random control*

**leash**

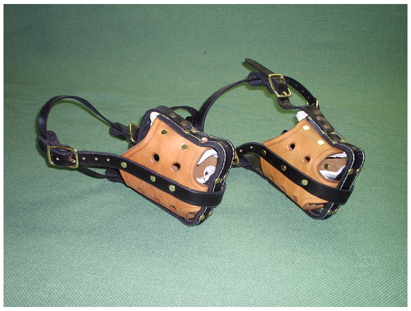

*predicted*

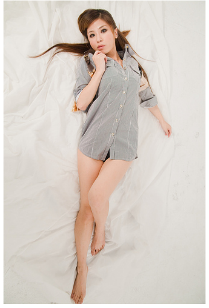

*random control*

**cactus**

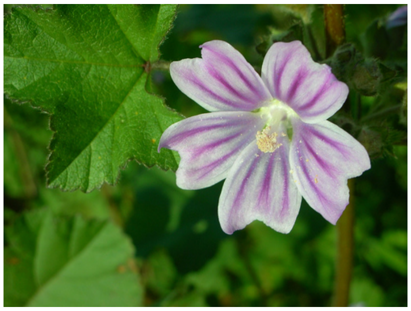

*predicted*

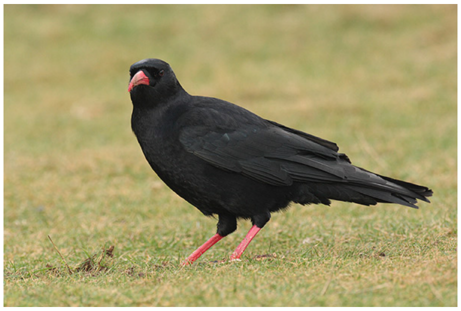

*random control*

**rug**

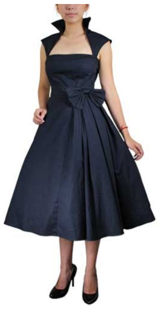

*predicted*

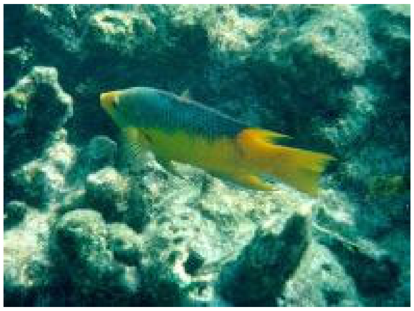

*random control*

**chimp**

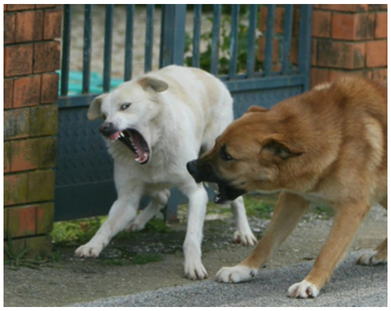

*predicted*

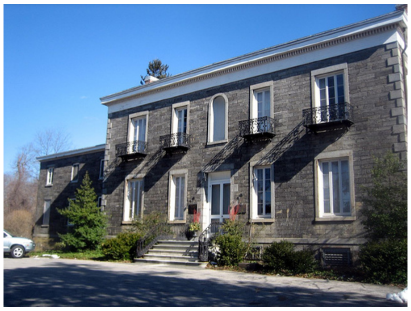

*random control*

**scenery**

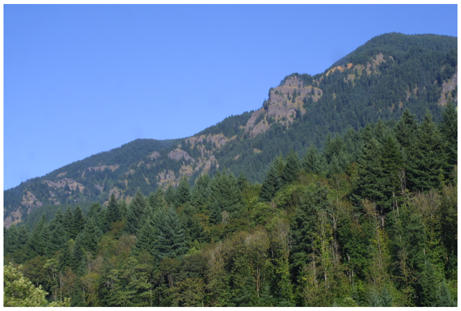

*predicted*

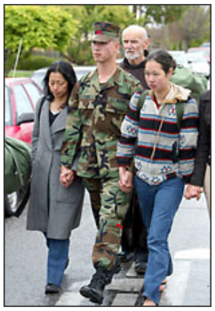

*random control*

**writing**

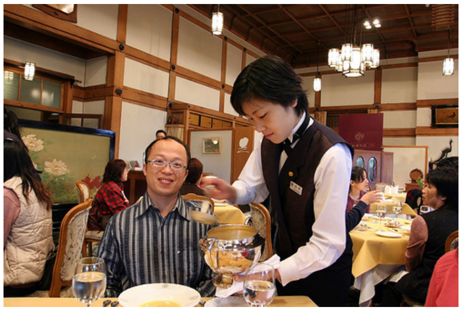

*predicted*

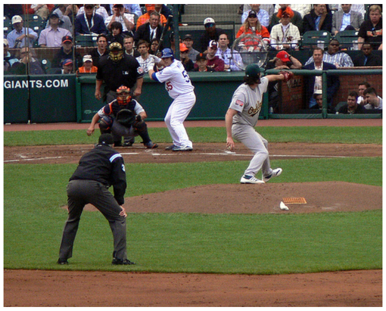


*random control*

**address**

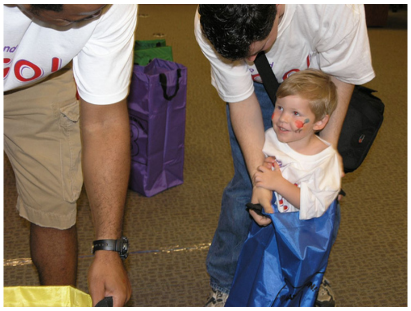

*predicted*

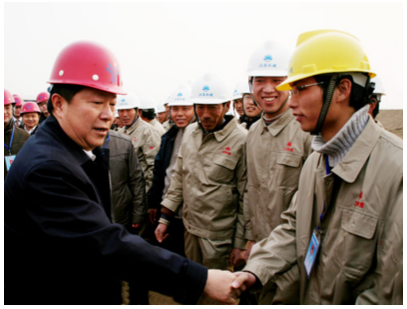

*random control*

**killing**

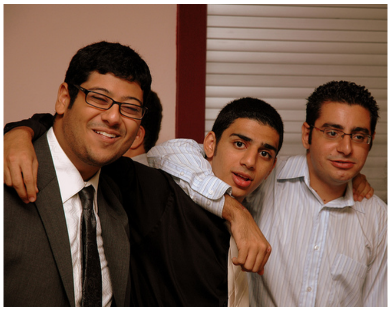

*predicted*

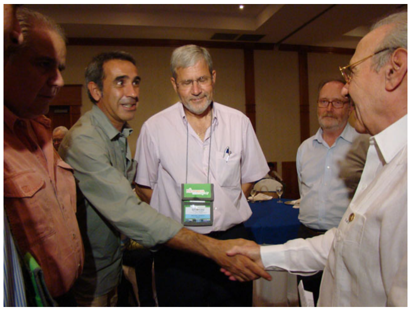

*random control*

**greeting**

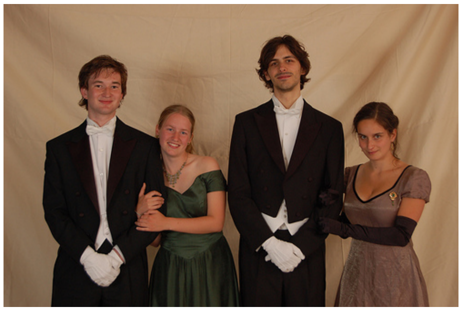

*predicted*

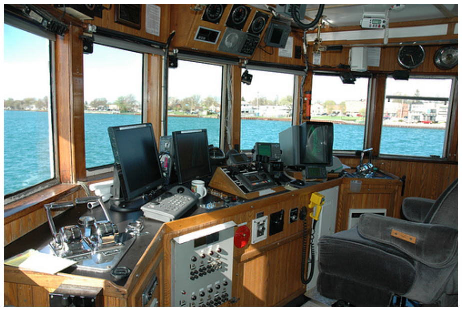

*random control*

**paragraph**

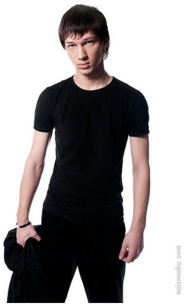

*predicted*

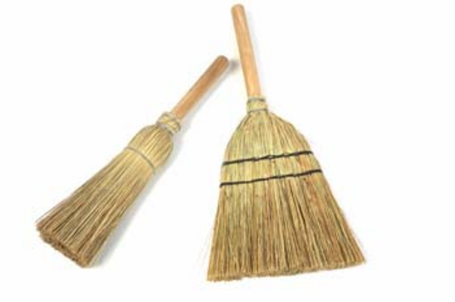

*random control*

**bent**

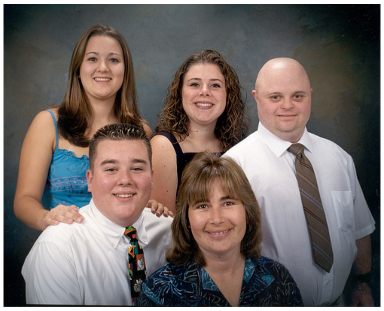

*predicted*

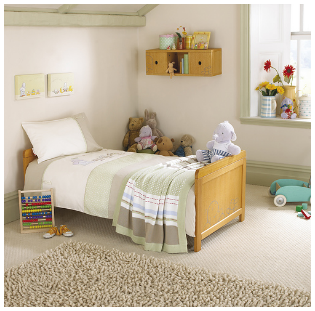

*random control*

**dime**

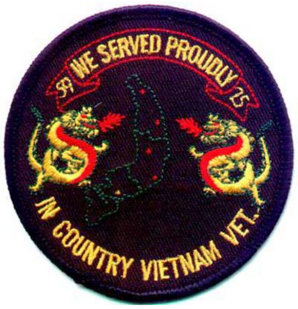


*predicted*

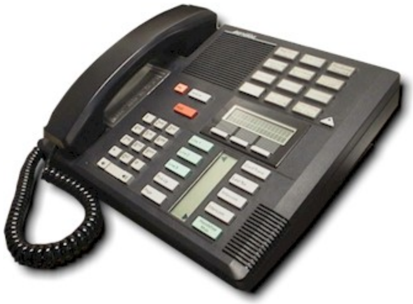

*random control*

**filth**

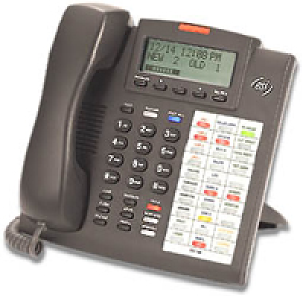

*predicted*

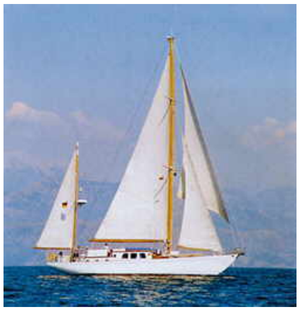

*random control*

**mob**

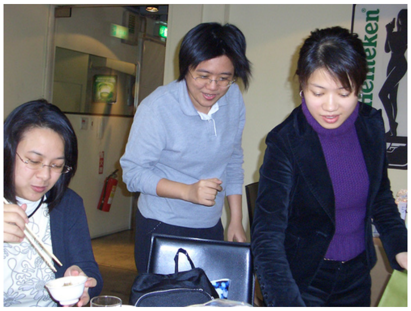

*predicted*

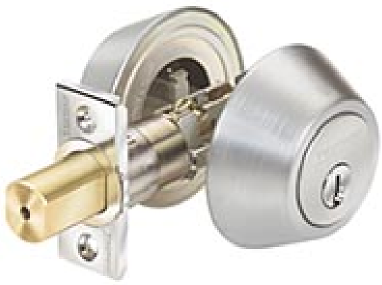

*random control*

**sedative**

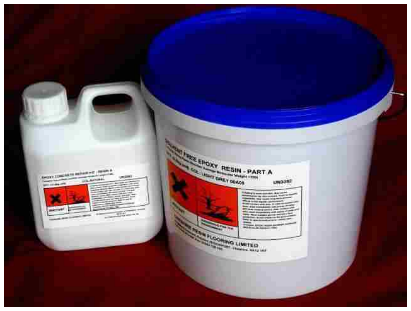

*predicted*

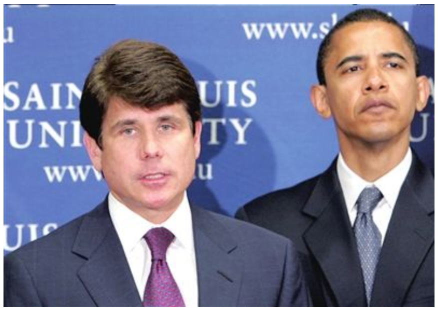

*random control*

**plague**

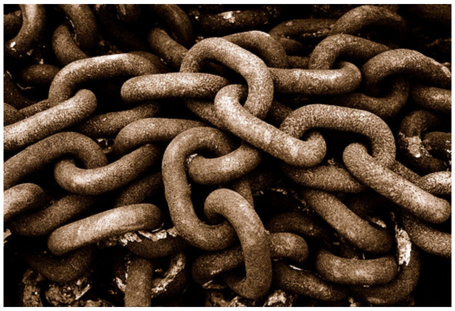

*predicted*

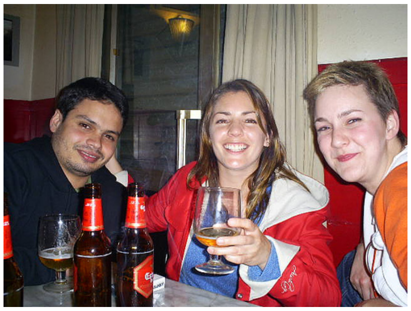

*random control*

**petition**

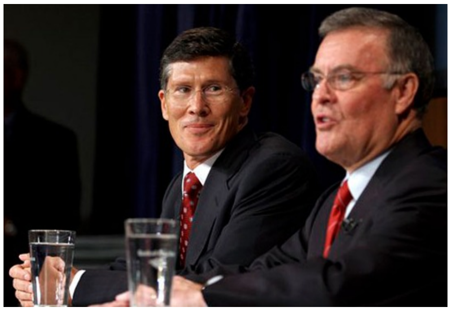

*predicted*

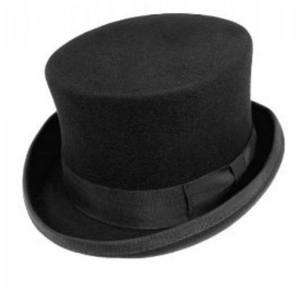

*random control*

**asthma**

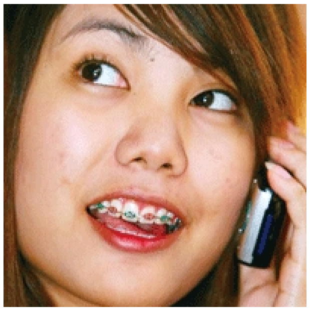

*predicted*

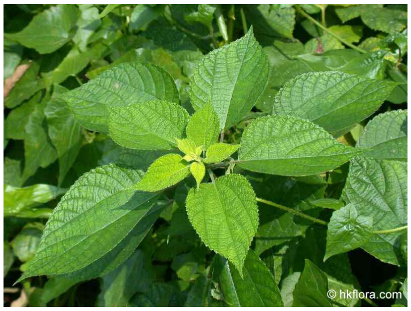


*random control*

**scrap**

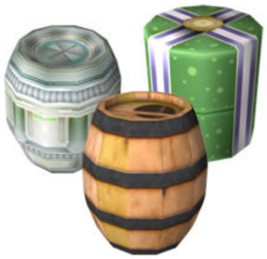

*predicted*

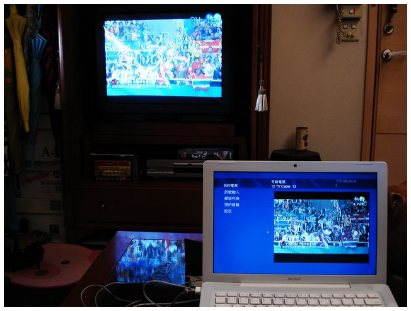

*random control*

**gram**

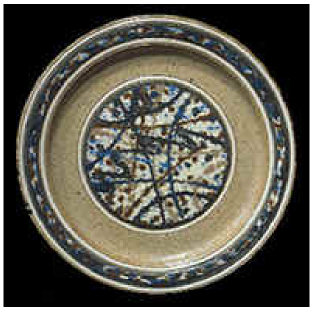

*predicted*

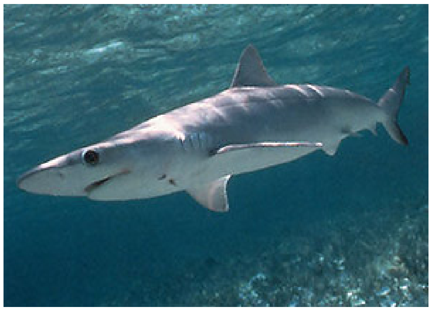

*random control*

## biology

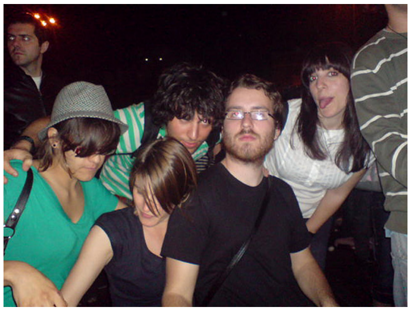

*predicted*

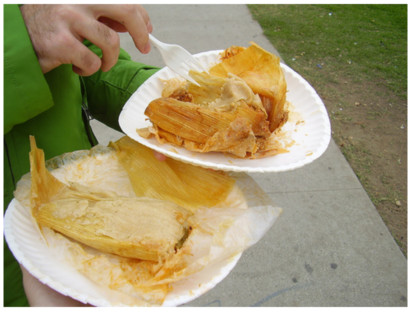

*random control*

## operation

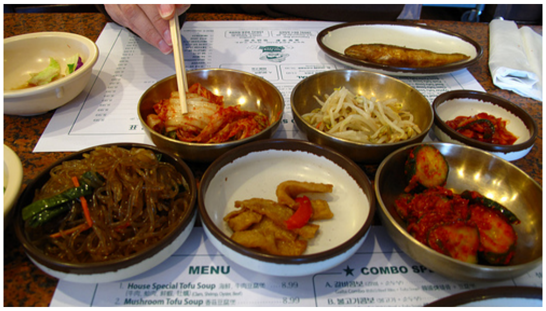


*predicted*

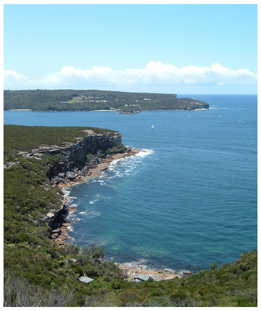

*random control*

## bruise

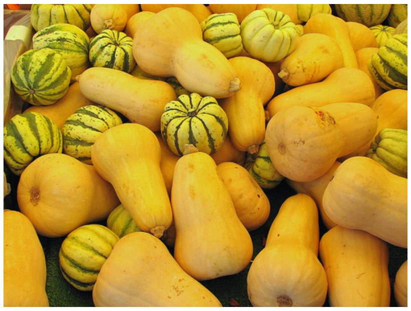

*predicted*

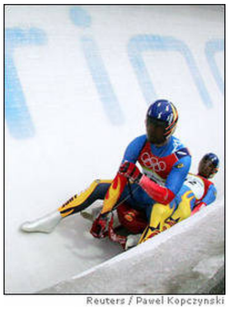


*random control*

**symphony**

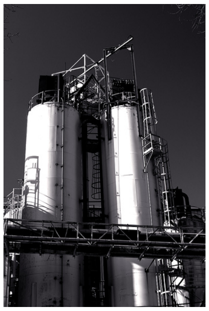

*predicted*

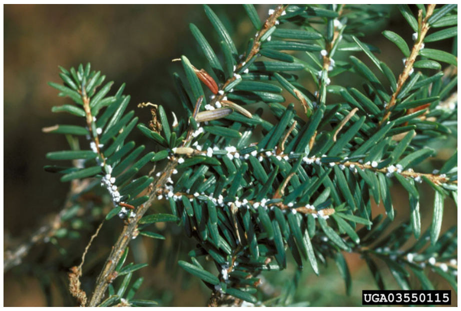


*random control*

**aspirin**

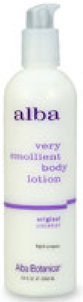


*predicted*

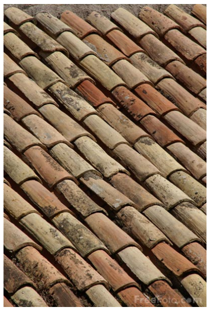

*random control*

**strike**

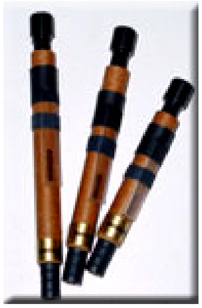

*predicted*

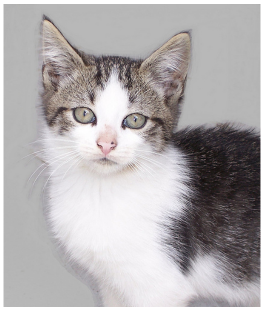

*random control*

**bible**

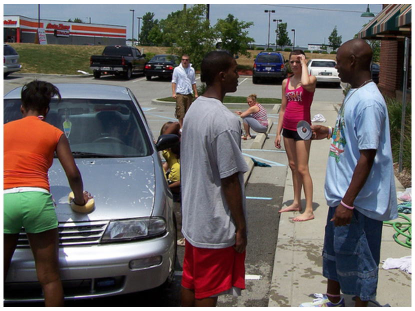

*predicted*

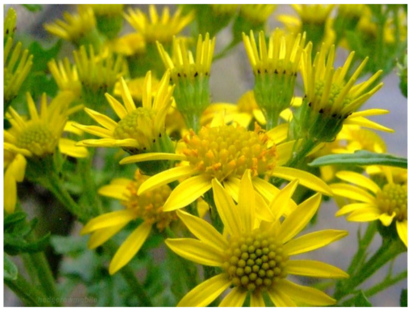

*random control*

**land**

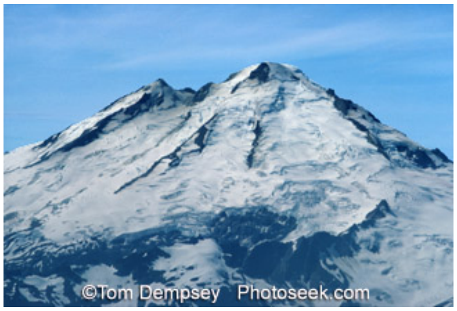


*predicted*

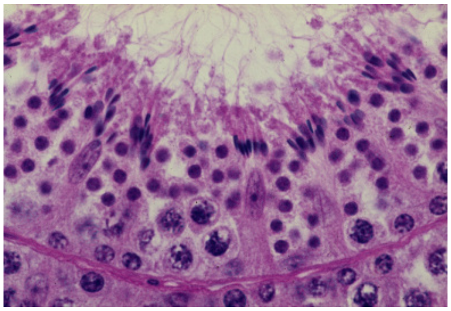

*random control*

**poison**

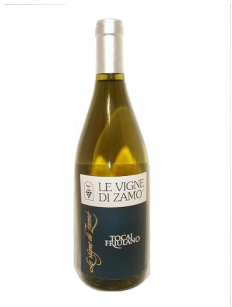


*predicted*

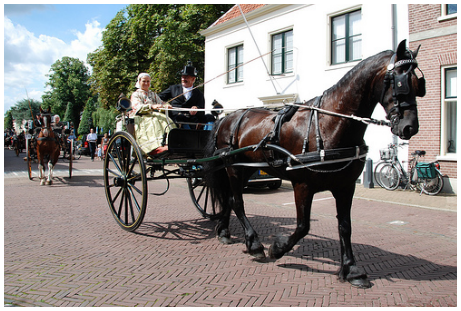

*random control*

**flame**

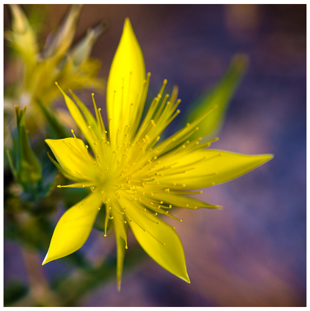

*predicted*

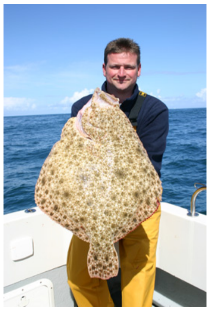

*random control*

**playboy**

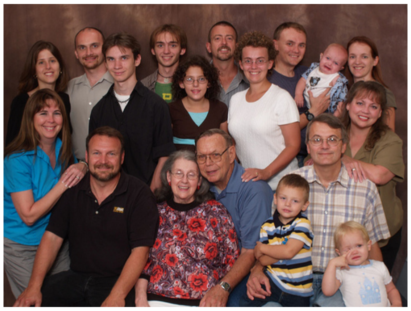

*predicted*

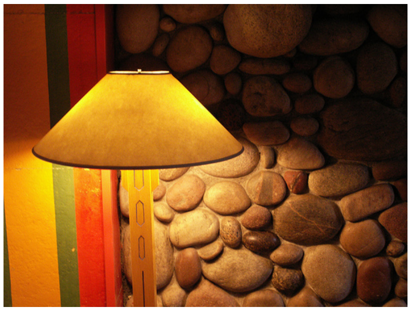

*random control*

**traps**

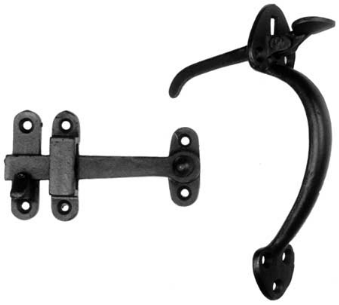

*predicted*

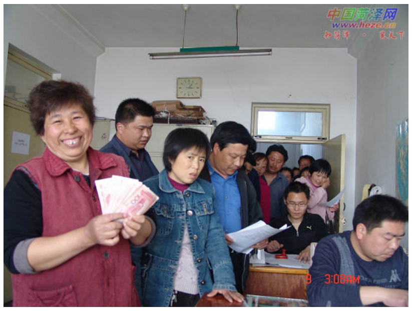

*random control*

**scout**

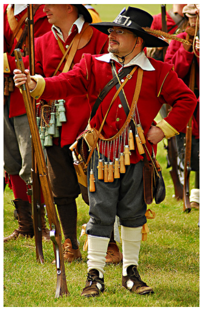

*predicted*

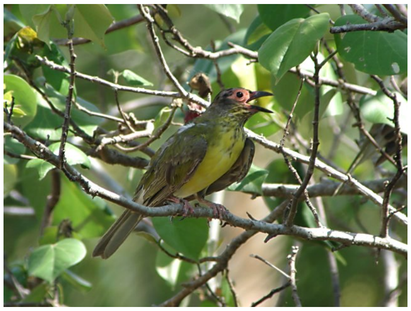

*random control*

**cardboard**

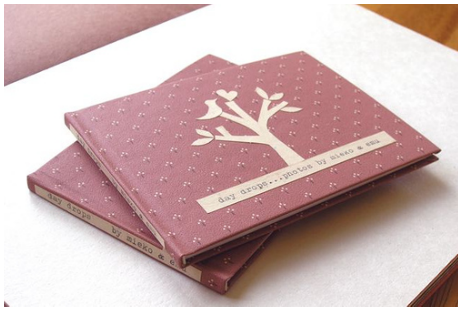

*predicted*

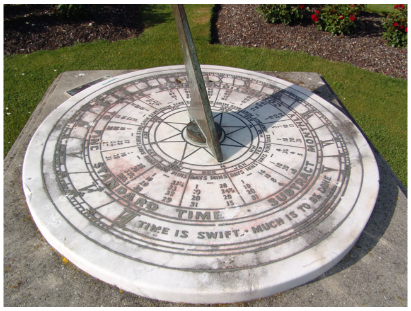


*random control*

**archives**

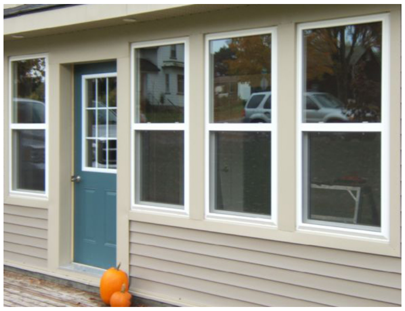

*predicted*

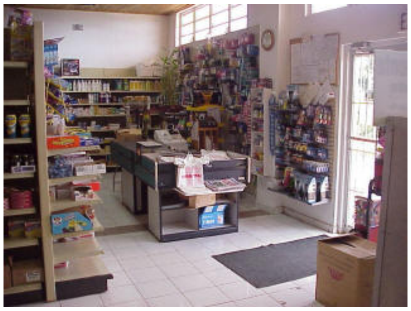

*random control*

**cutting**

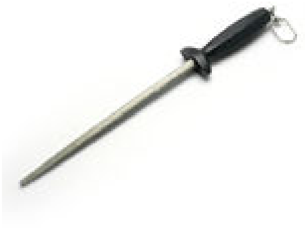

*predicted*

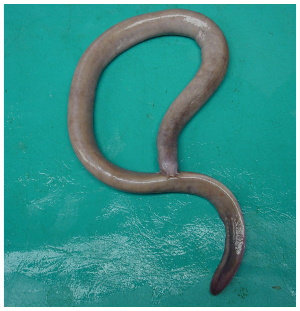

*random control*

**snack**

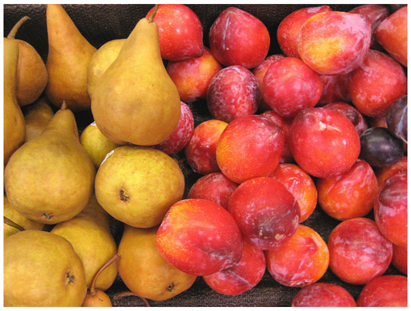

*predicted*

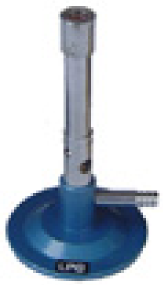

*random control*

**piglet**

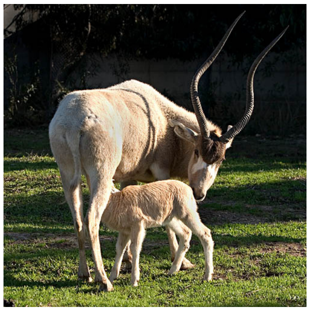

*predicted*

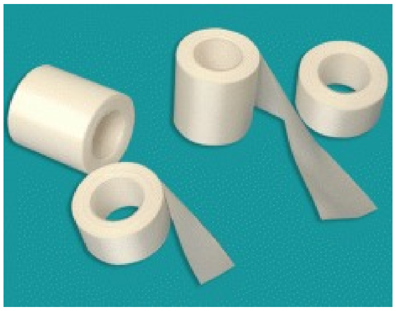

*random control*

**warden**

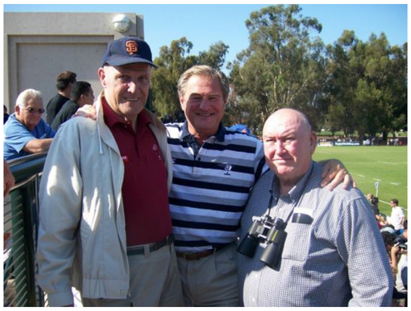

*predicted*

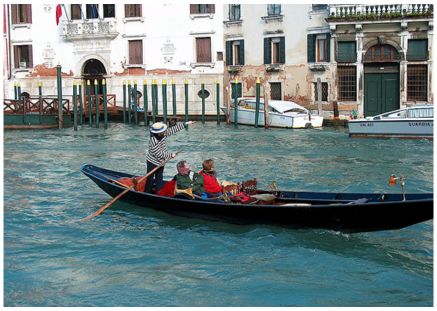

*random control*

**chop**

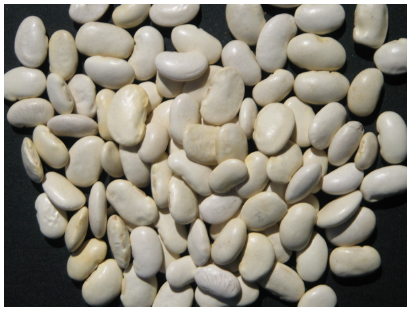

*predicted*

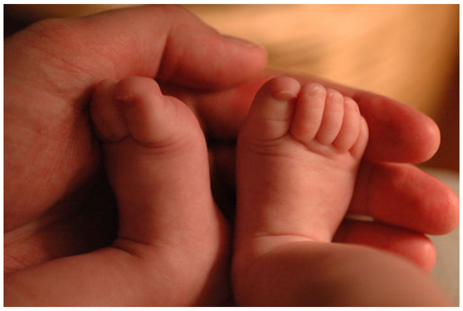

*random control*

**critic**

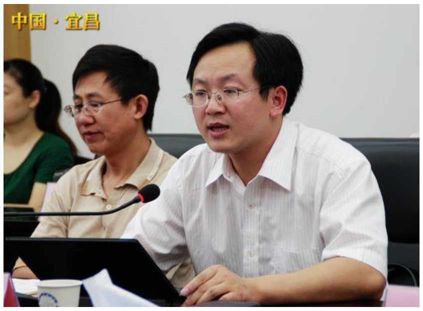


*predicted*

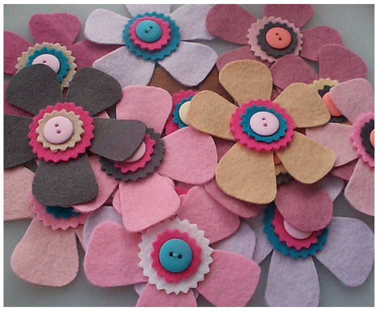

*random control*

**muffin**

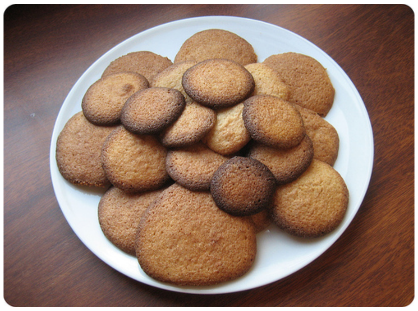

*predicted*

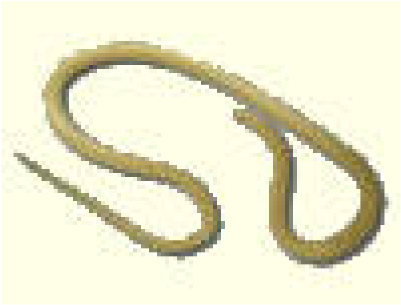

*random control*

**assistant**

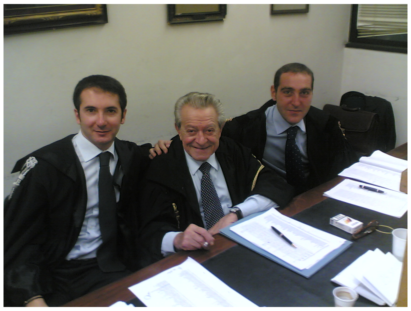

*predicted*

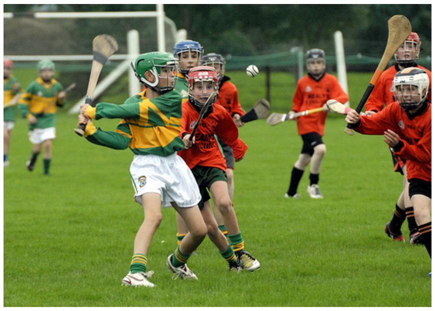

*random control*

**paramedic**

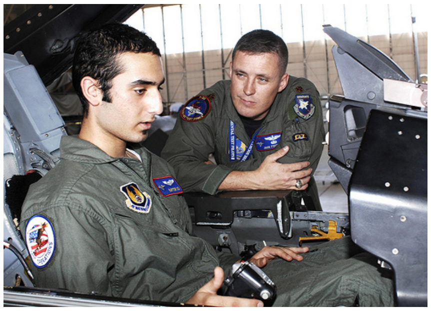

*predicted*

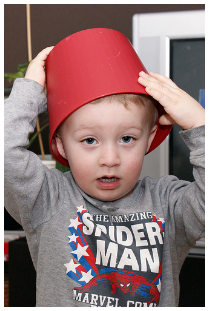


*random control*

**auntie**

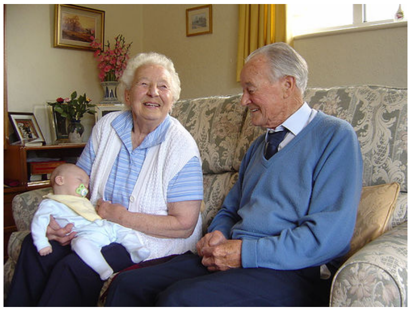

*predicted*

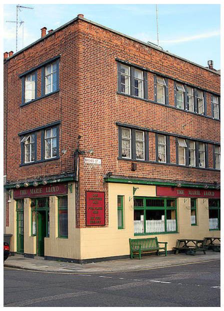

*random control*

**preacher**

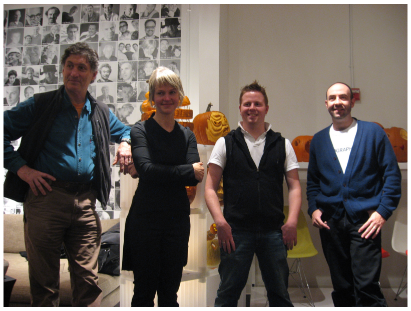

*predicted*

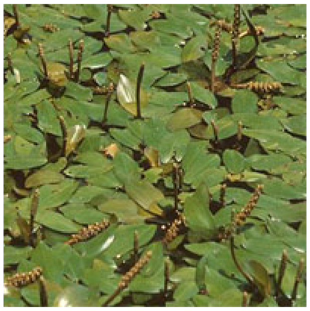

*random control*

**skeleton**

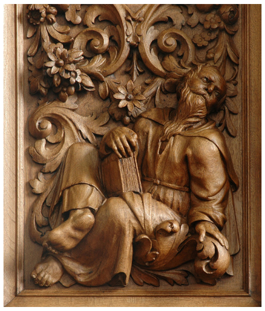

*predicted*

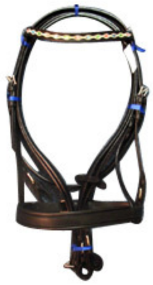

*random control*

**lily**

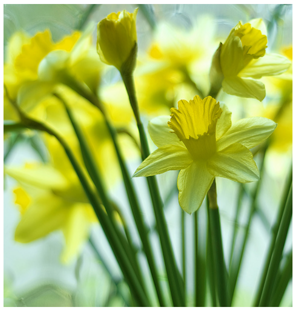

*predicted*

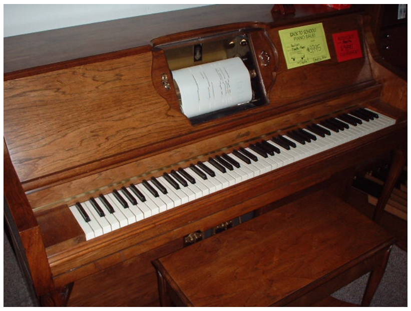

*random control*

**leather**

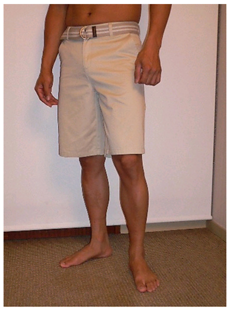

*predicted*

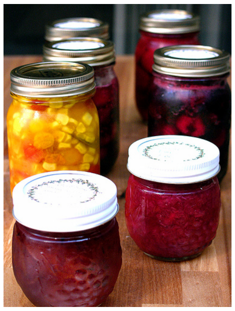

*random control*

**ally**

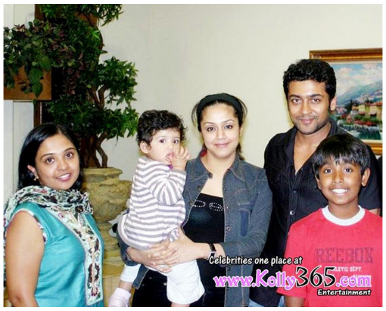


*predicted*

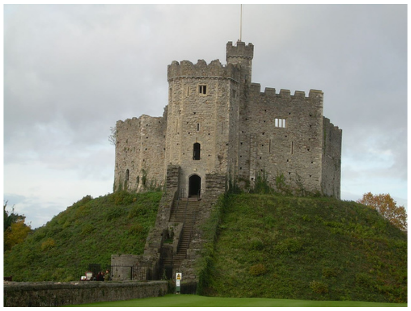

*random control*

**bout**

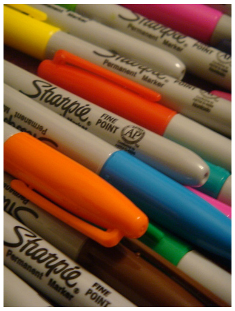


*predicted*

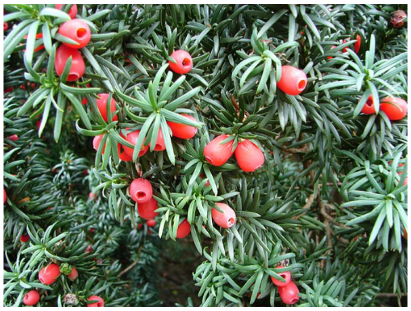

*random control*

**breed**

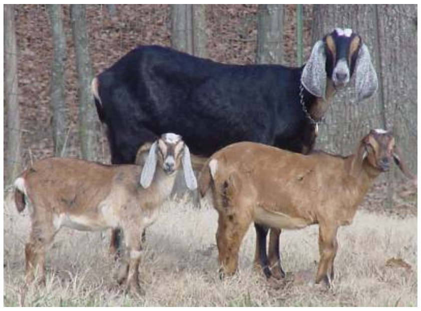

*predicted*

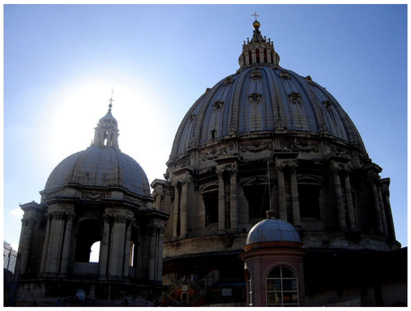

*random control*

**childhood**

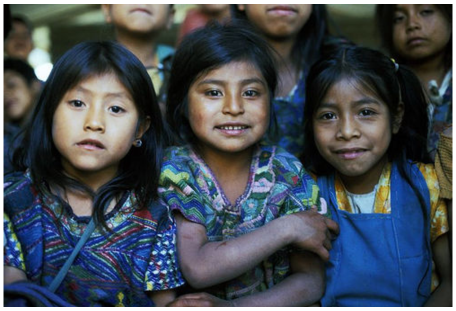

*predicted*

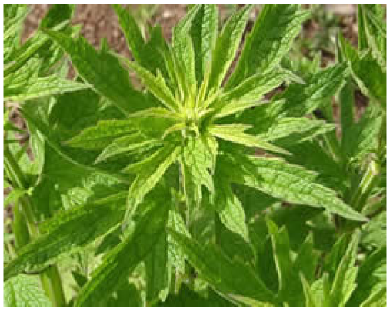

*random control*

**corporal**

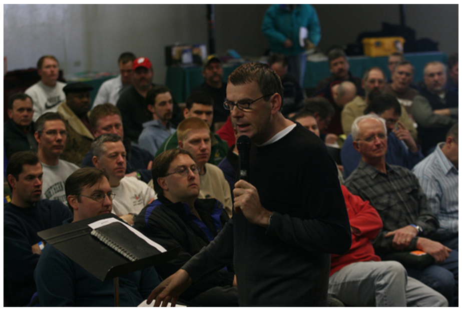

*predicted*

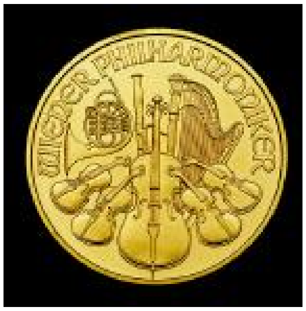


*random control*

**defensive**

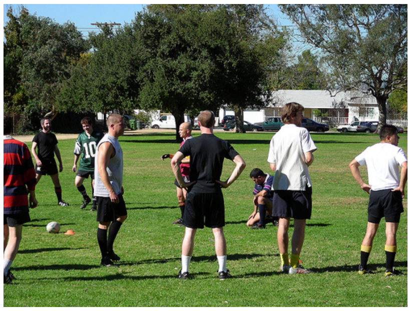

*predicted*

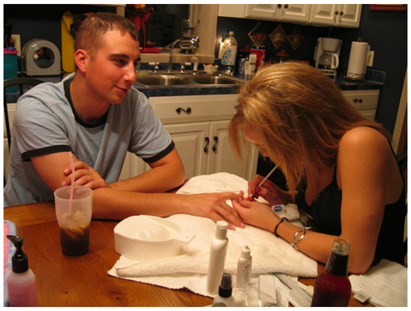

*random control*

**department**

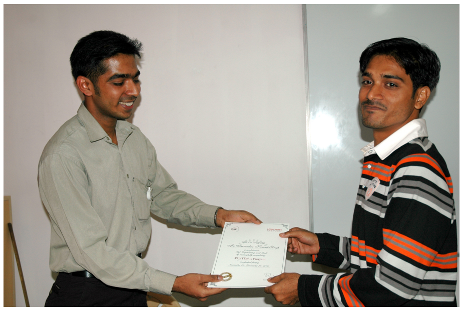

*predicted*

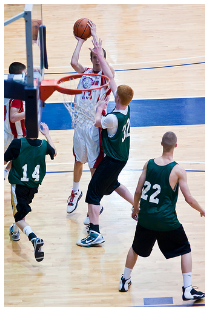

*random control*

**fit**

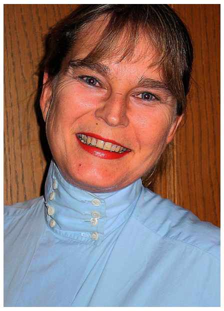

*predicted*

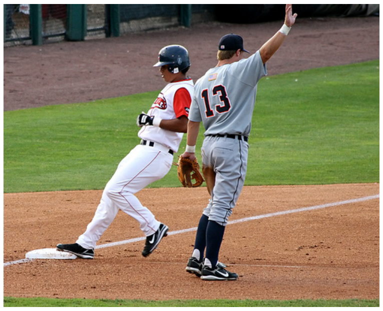


*random control*

**fore**

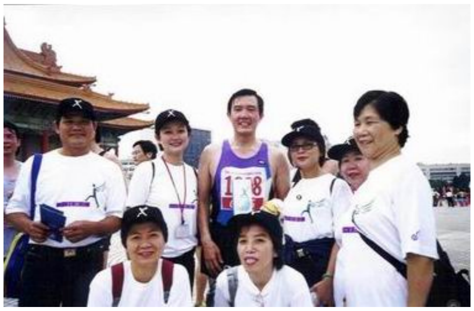

*predicted*

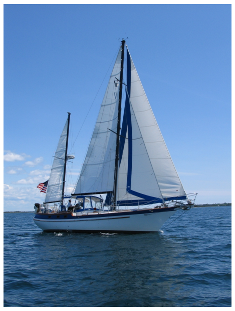

*random control*

**gall**

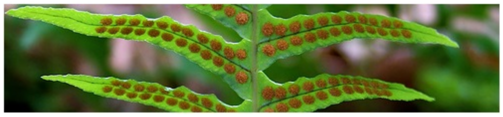

*predicted*

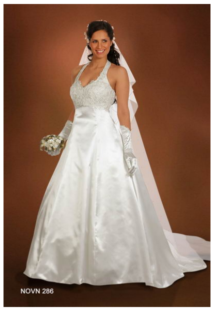


*random control*

**graduating**

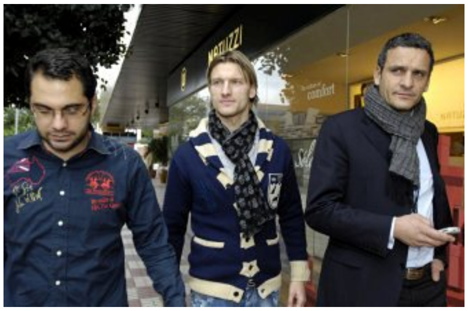

*predicted*

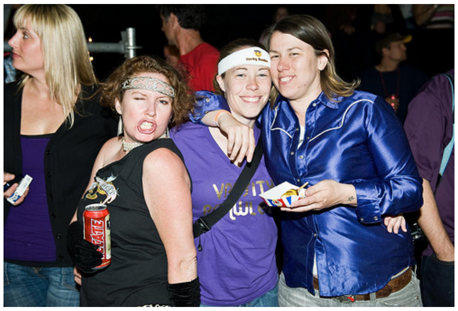

*random control*

**journalism**

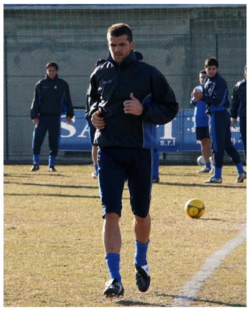

*predicted*

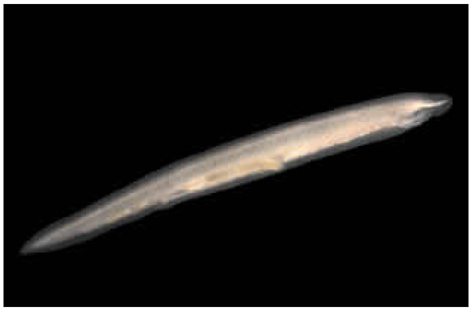

*random control*

**main**

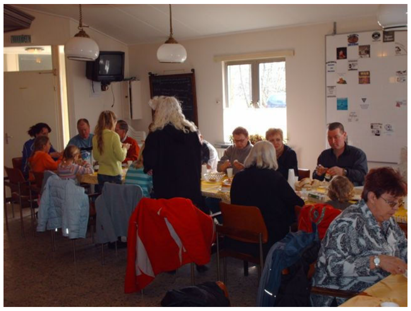

*predicted*

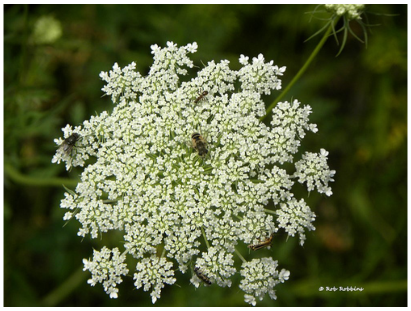

*random control*

**member**

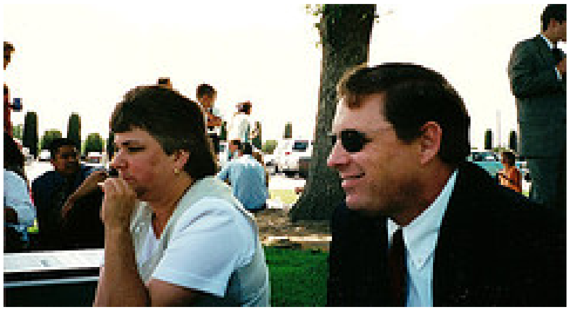

*predicted*

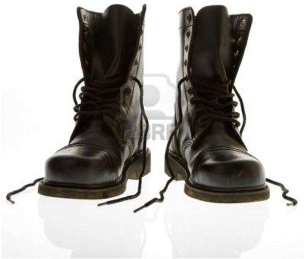

*random control*

**outsider**

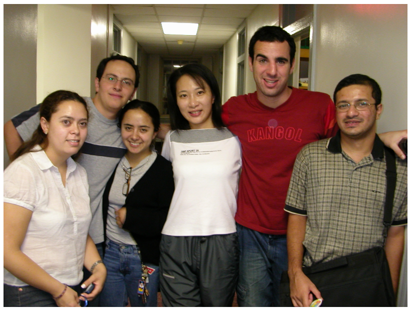

*predicted*

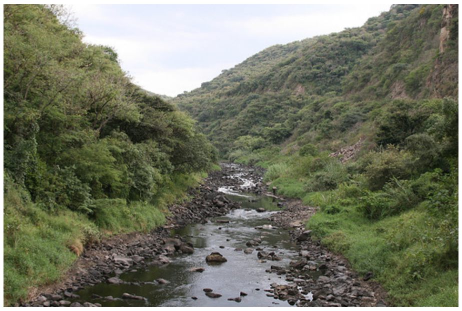

*random control*

**position**

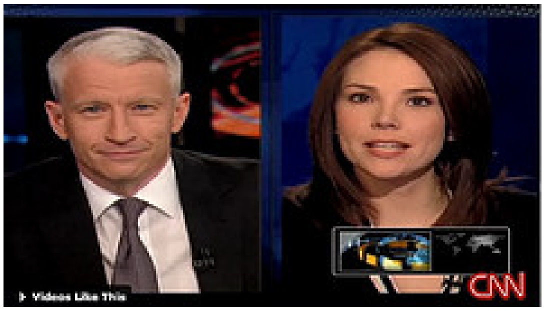


*predicted*

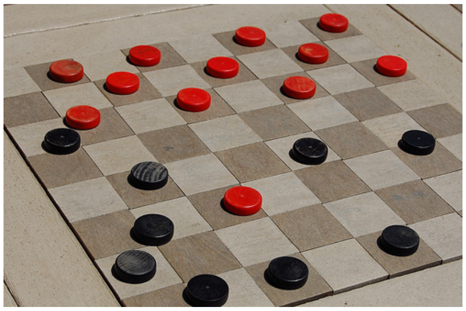

*random control*

**salvage**

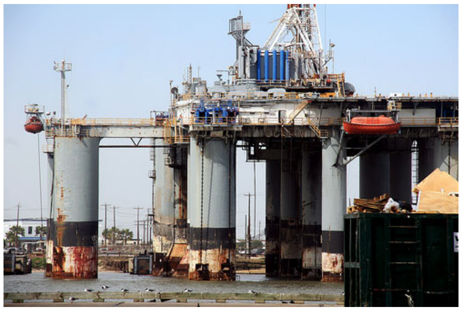

*predicted*

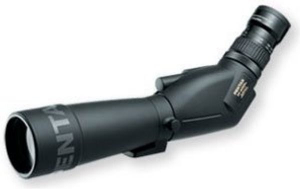

*random control*

**scoundrel**

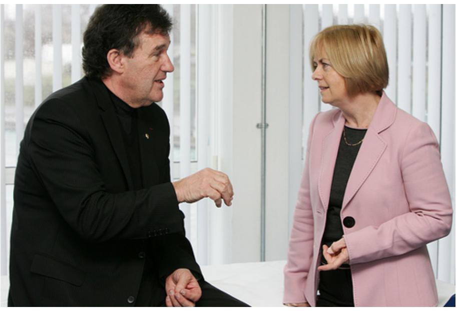

*predicted*

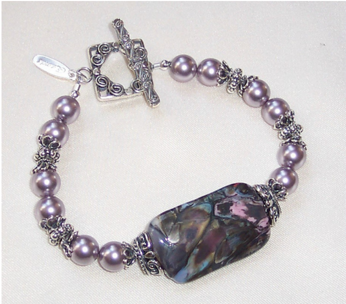

*random control*

**sight**

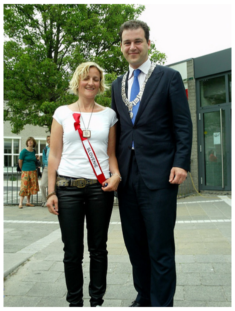

*predicted*

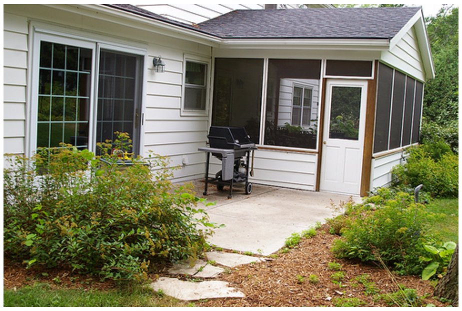

*random control*

**starboard**

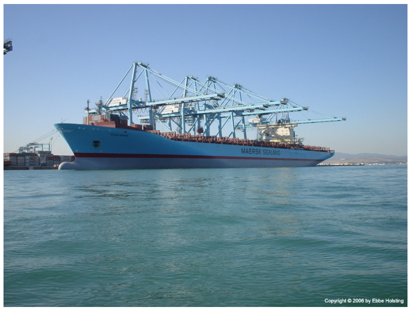

*predicted*

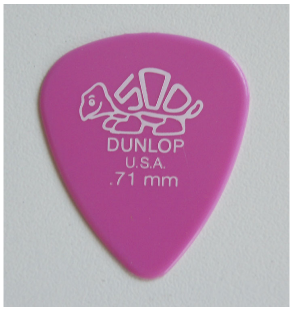


*random control*

**vice**

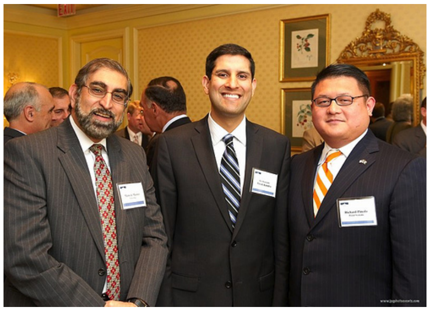

*predicted*

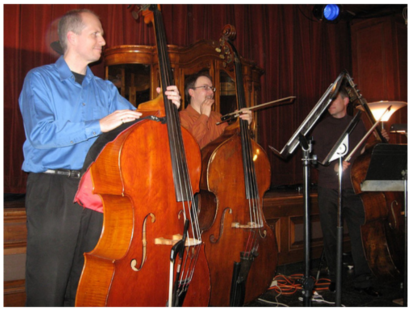

*random control*

**vogue**

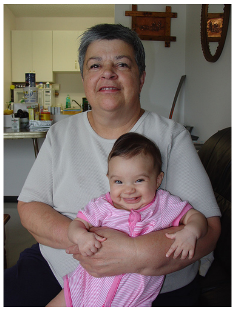

*predicted*

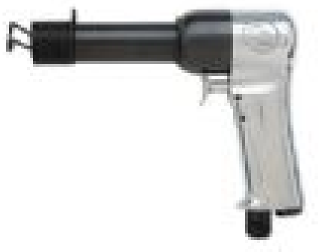

*random control*

**wild**

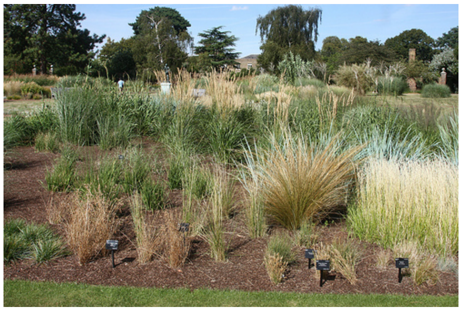

*predicted*

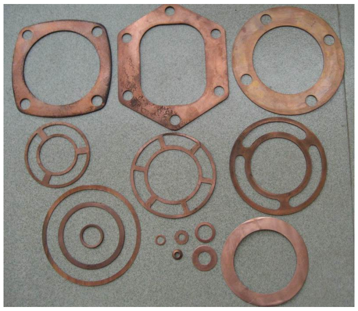

*random control*

**virtue**

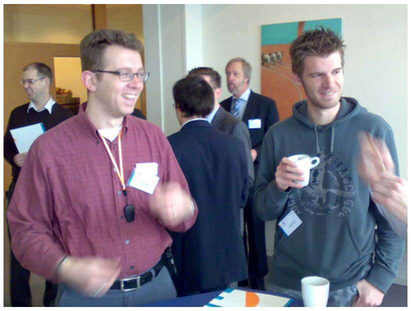

*predicted*

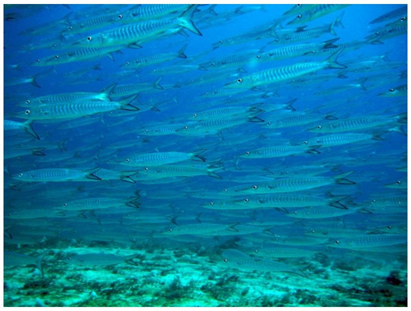

*random control*

**perfection**

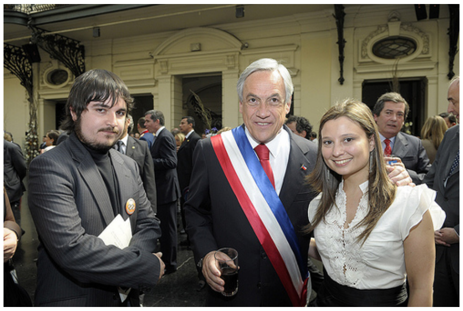

*predicted*

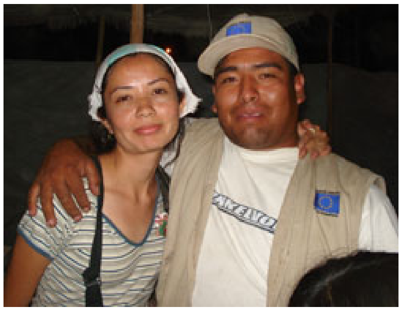

*random control*

**awareness**

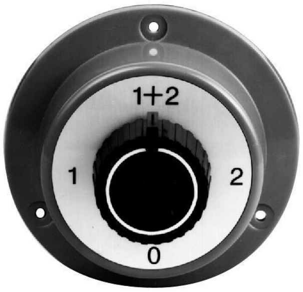


*predicted*

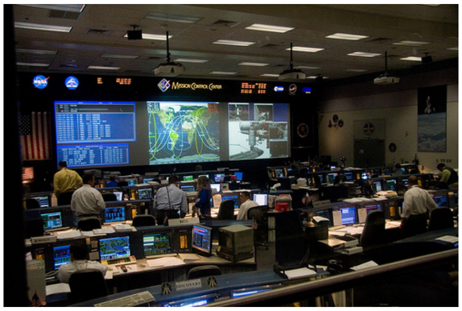

*random control*

**madness**

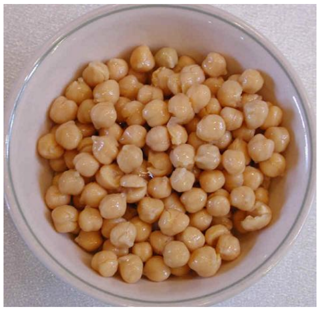

*predicted*

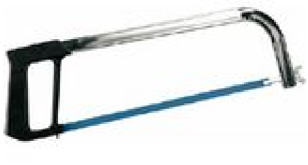

*random control*

**determination**

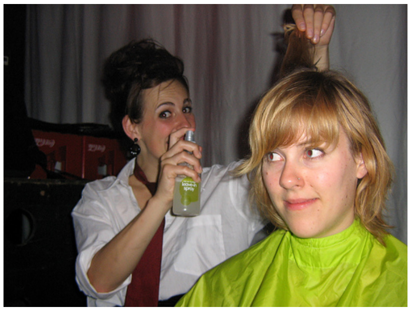

*predicted*

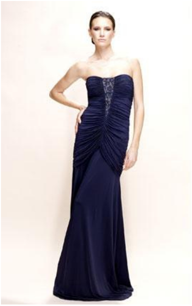

*random control*

**metaphor**

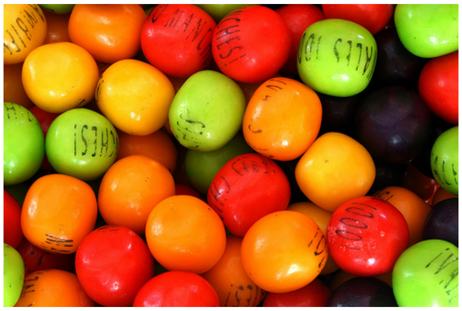

*predicted*

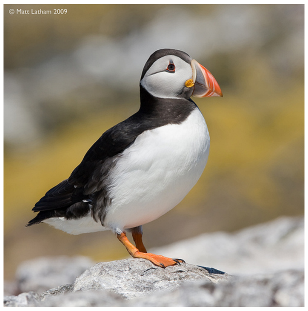

*random control*

**curiosity**

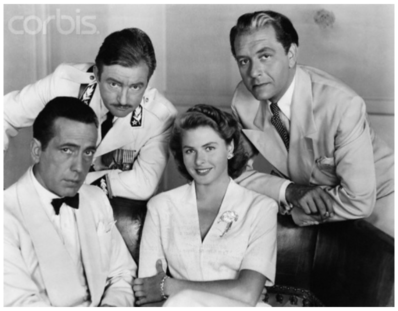


*predicted*

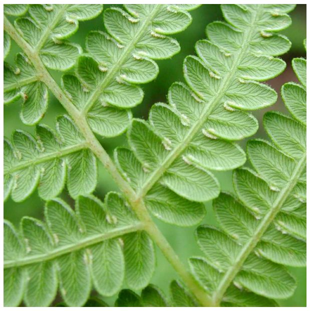

*random control*

**destiny**

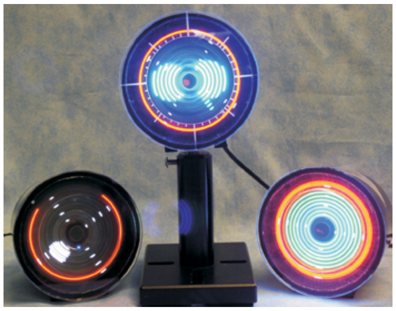

*predicted*

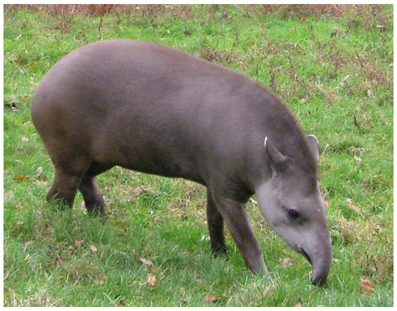

*random control*

**opportunity**

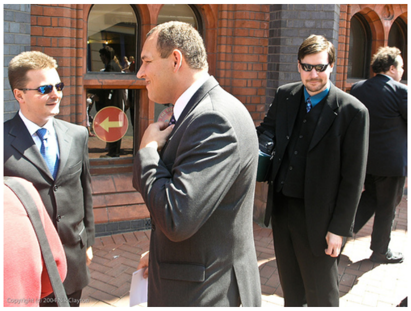
*predicted*

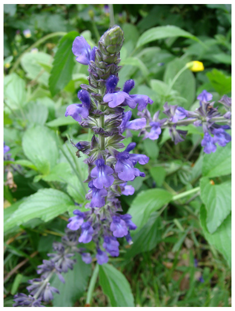
*random control*

**aspect**

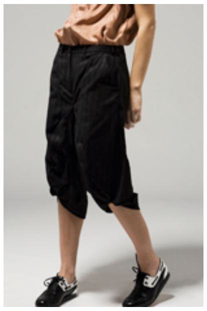
*predicted*

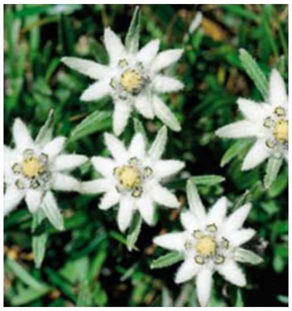
*random control*

**experience**

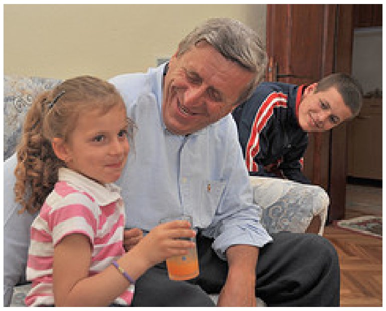
*predicted*

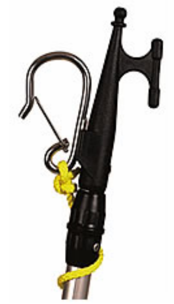
*random control*

**jealousy**

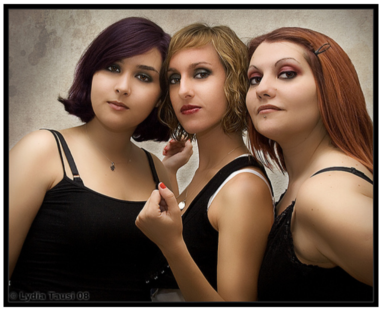


*predicted*

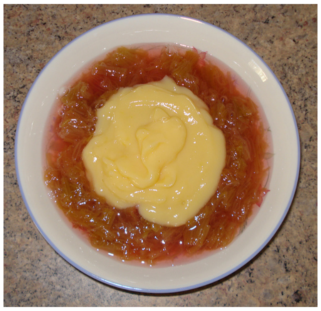

*random control*

**funk**

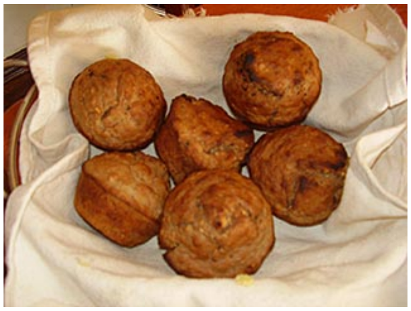

*predicted*

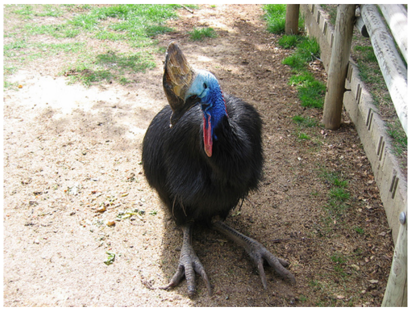

*random control*

**meaning**

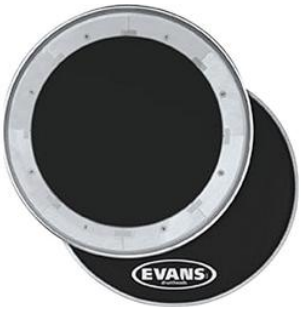


*predicted*

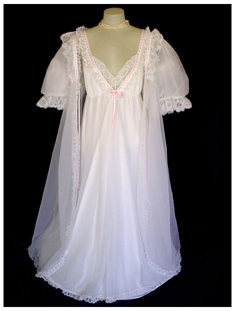

*random control*

**luck**

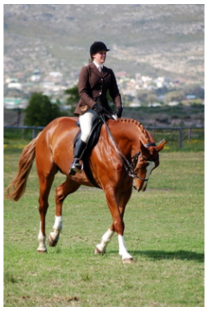

*predicted*

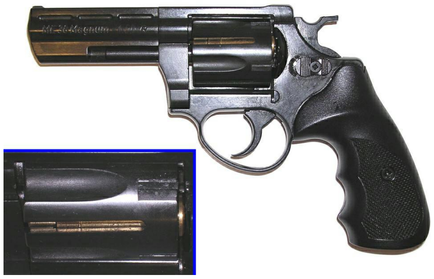

*random control*

**contrary**

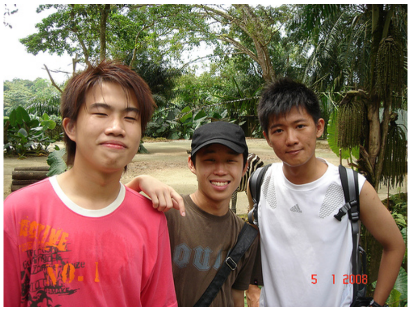


*predicted*

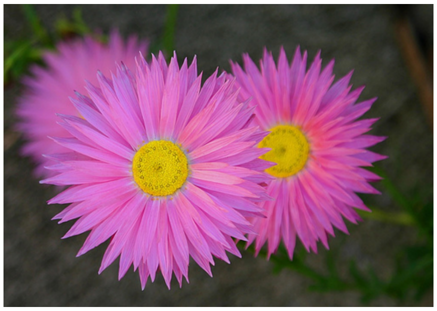

*random control*

**sadness**

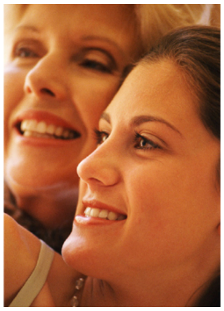

*predicted*

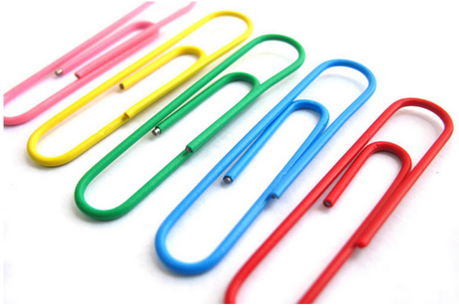

*random control*

**conscience**

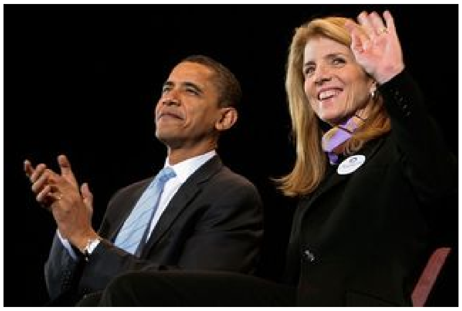

*predicted*

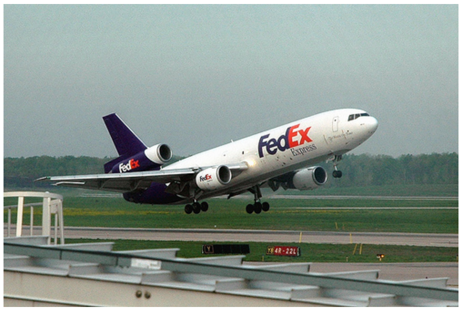


*random control*

**inconvenience**

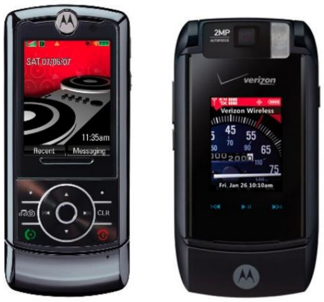


*predicted*

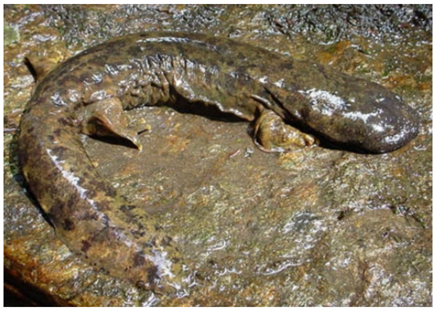

*random control*

**boredom**

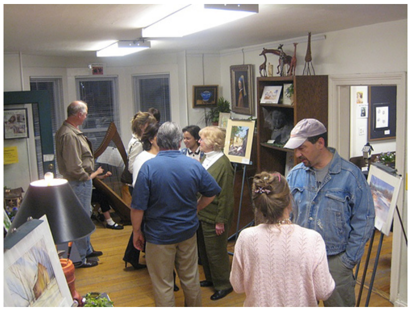

*predicted*

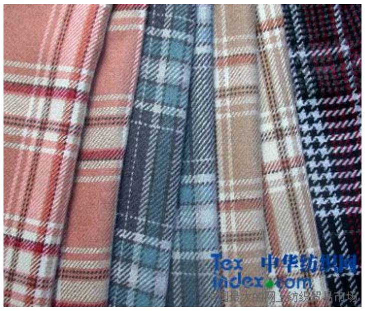

*random control*

**debut**

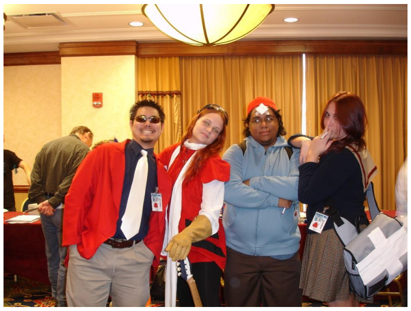

*predicted*

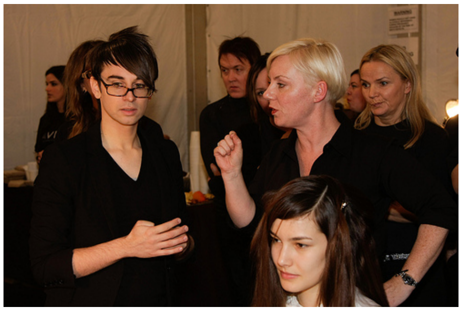

*random control*

**worth**

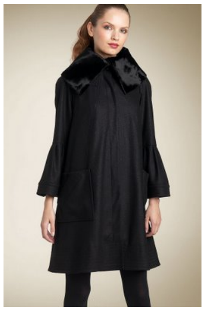

*predicted*

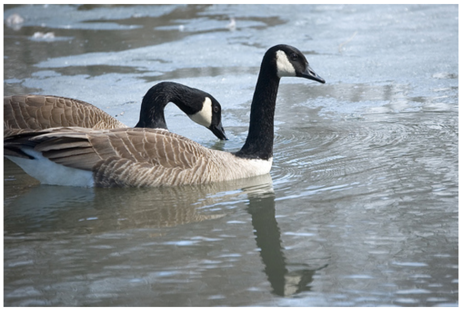

*random control*

**principle**

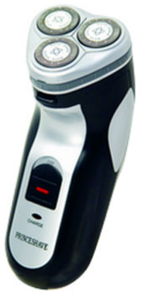

*predicted*

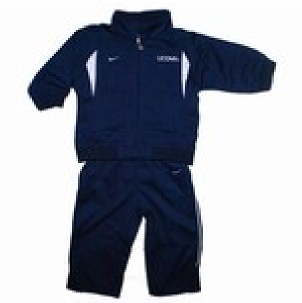

*random control*
