# Supplementary material for: Images of the unseen: extrapolating visual representations for abstract and concrete words in a data-driven computational model
Source: Psychol Res. 2020 Nov 12;86(8):2512–32. doi: 10.1007/s00426-020-01429-7 (PMC9674750; doi:10.1007/s00426-020-01429-7)
Supplement: Supplementary file 2 — Supplementary material 2 (pdf 24305 KB) [file 426_2020_1429_MOESM2_ESM.pdf]

Experiment 2 (exemplar model)

freighter

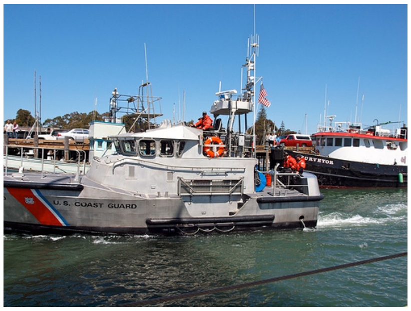

predicted

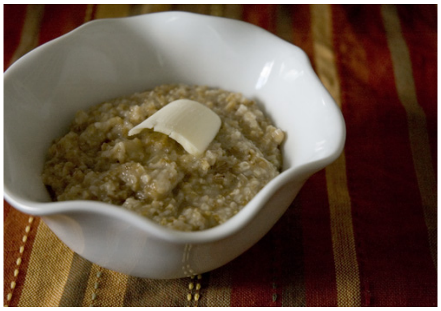

random control

pocket

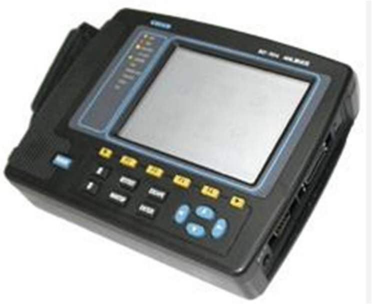

predicted

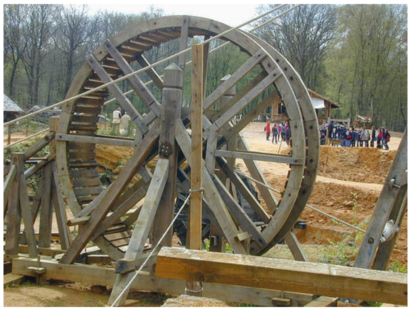

random control

uncle

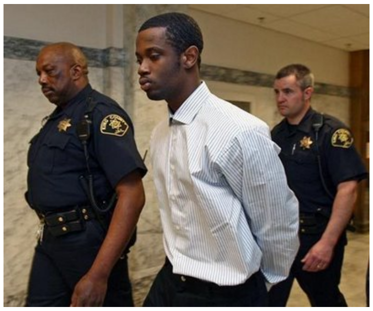

predicted

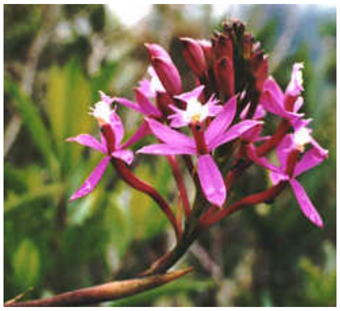

random control

performer

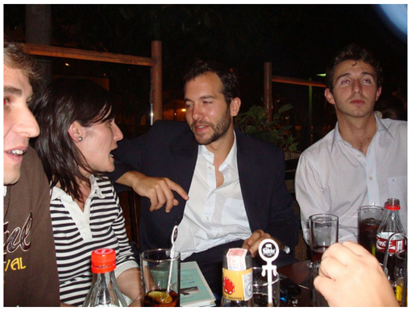

predicted

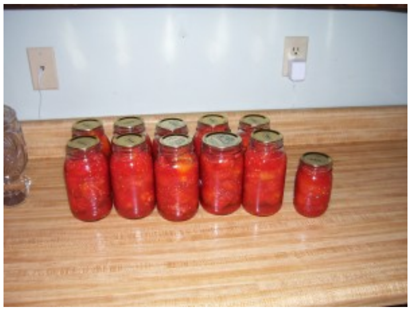

random control

whiskey

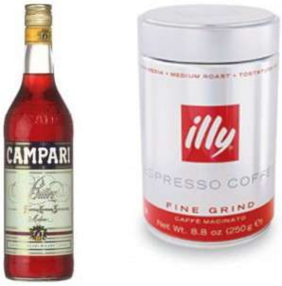

predicted

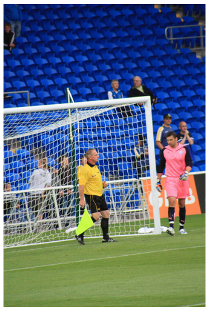

random control

hamburger

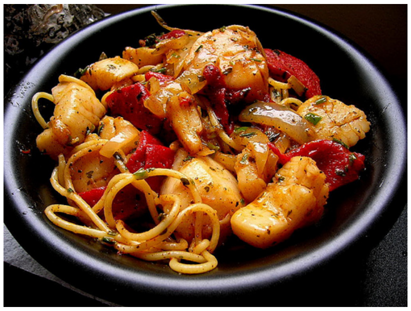

predicted

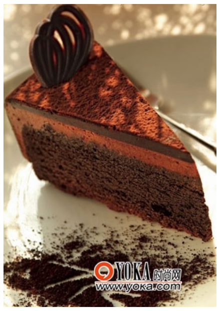

random control

barb

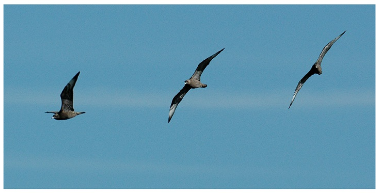

predicted

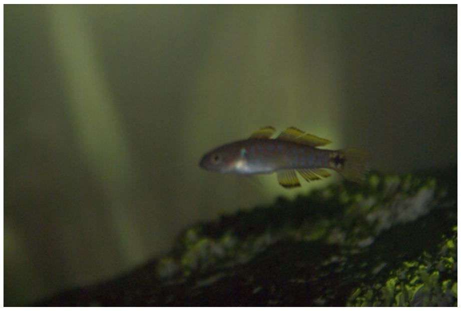

random control

cushion

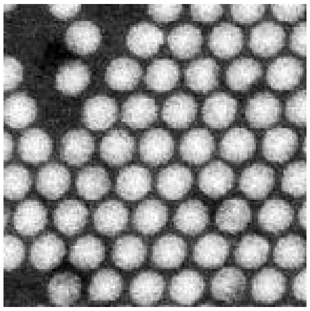

predicted

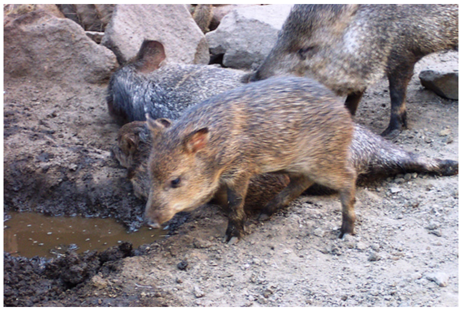

random control

spear

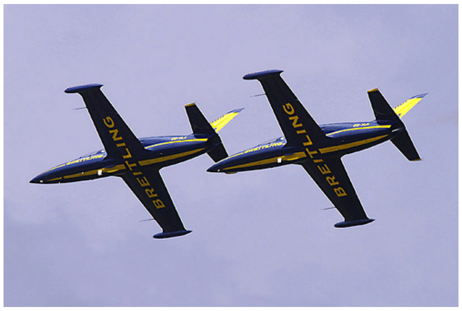

predicted

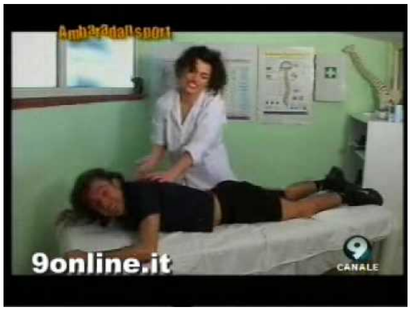

random control

engineer

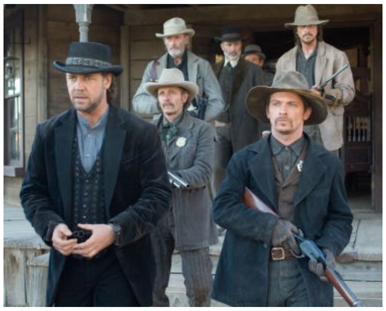

predicted

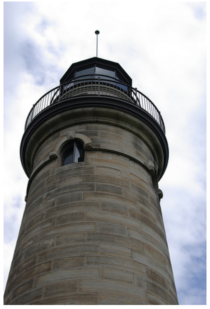

random control

cheesecake

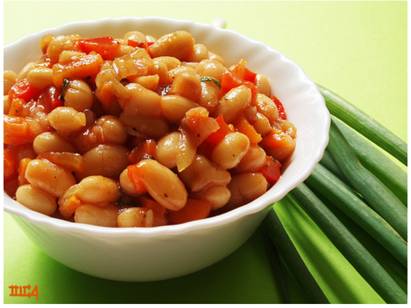

predicted

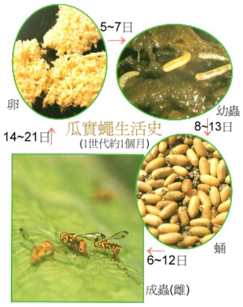

random control

ribbon

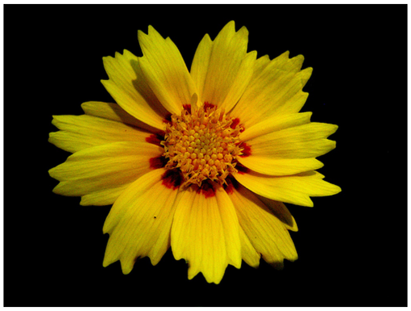

predicted

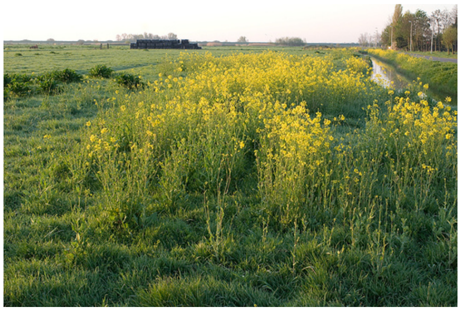

random control

cockroach

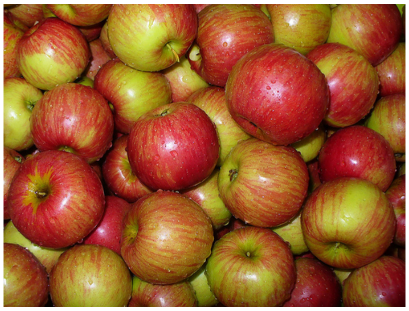

predicted

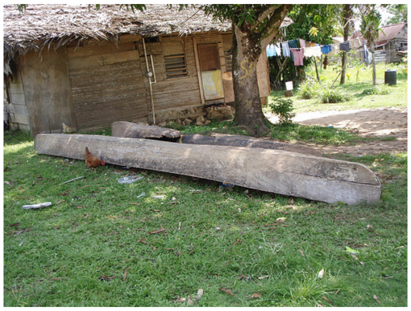

random control

stallion

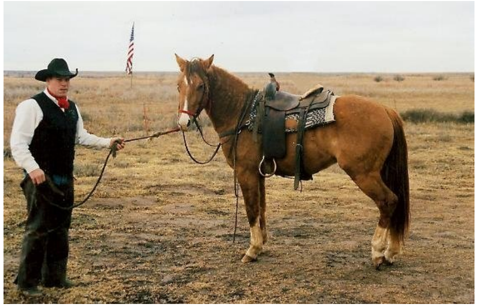

predicted

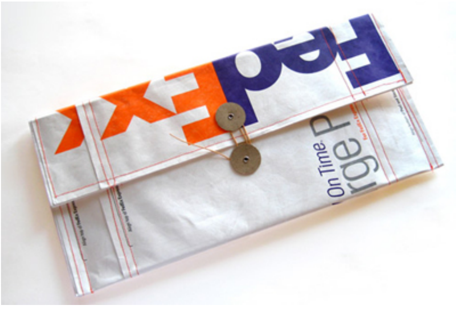

random control

crank

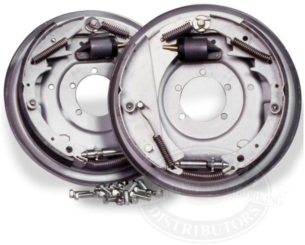

predicted

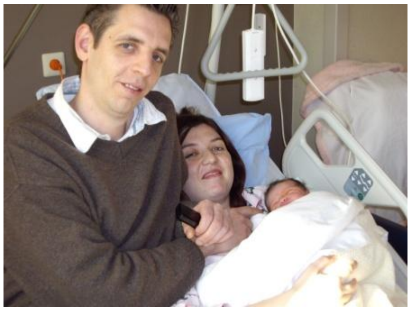

random control

cloth

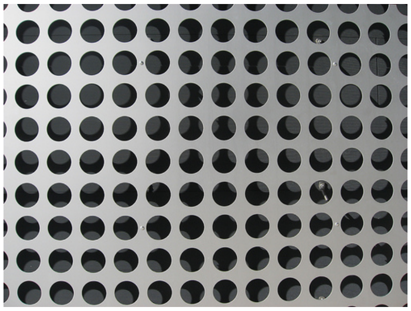

predicted

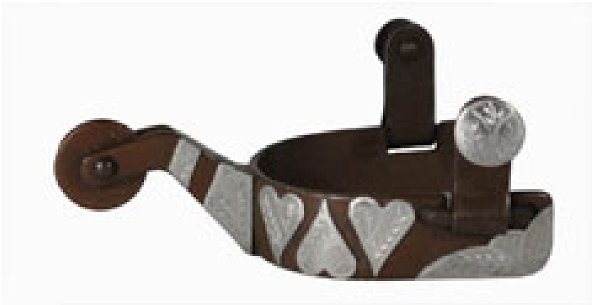

random control

housewife

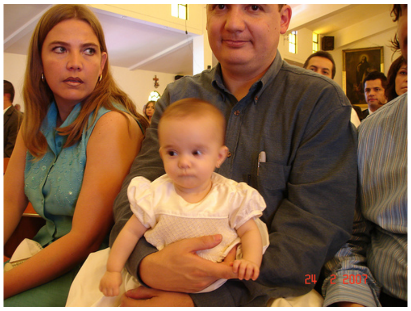

predicted

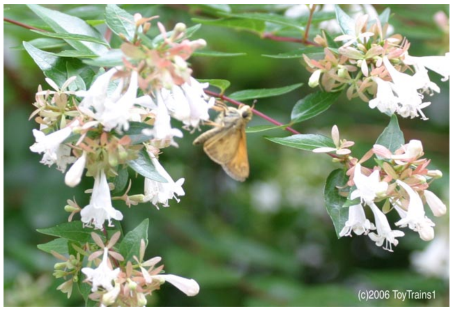

random control

ivy

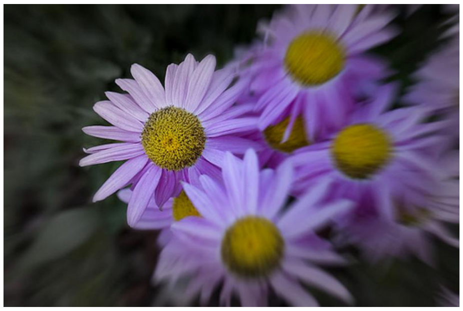

predicted

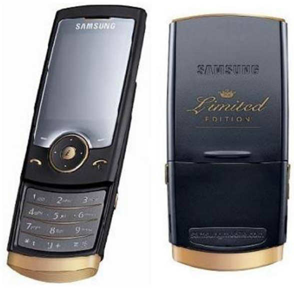

random control

tomb

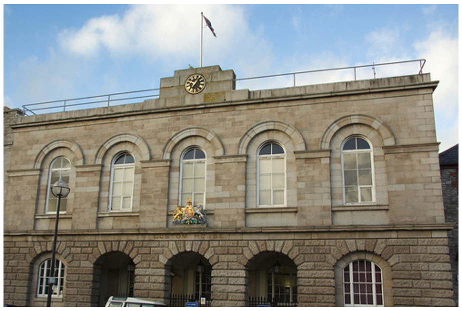

predicted

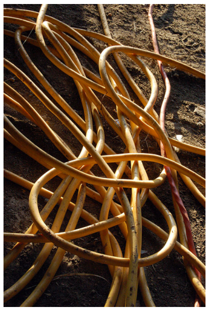

random control

leash

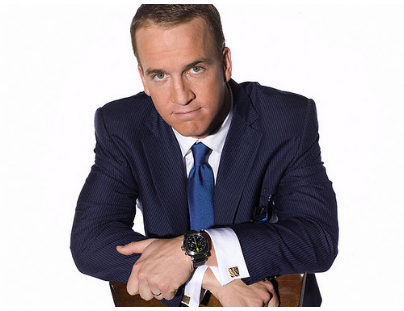

predicted

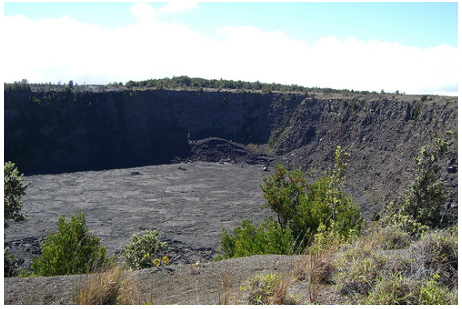

random control

cactus

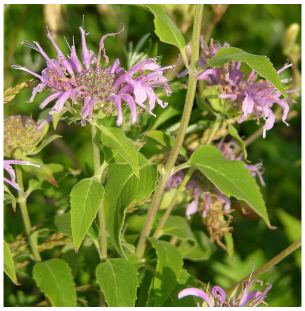

predicted

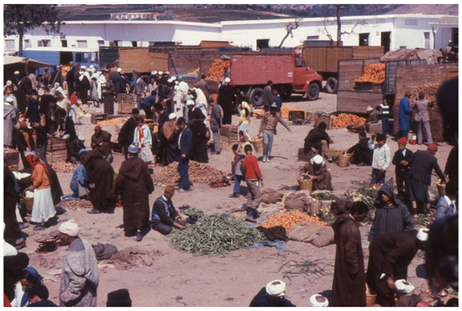

random control

rug

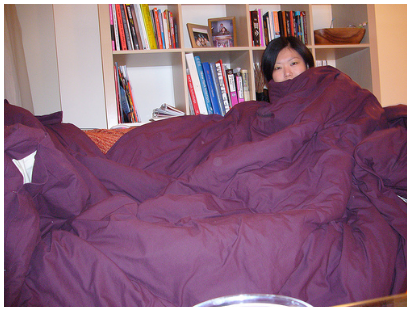

predicted

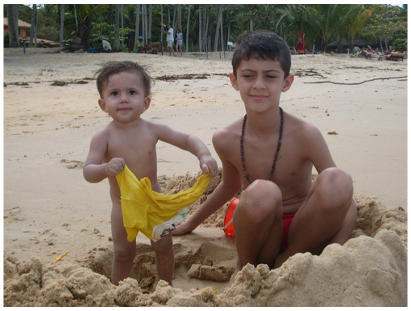

random control

chimp

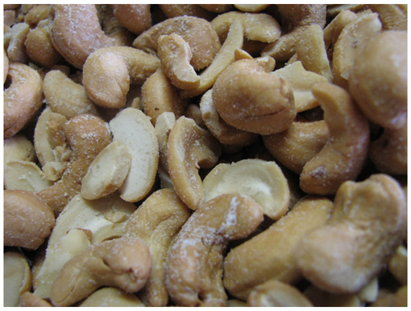

predicted

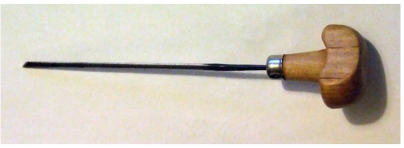

random control

scenery

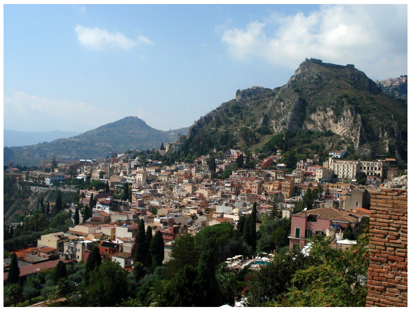

predicted

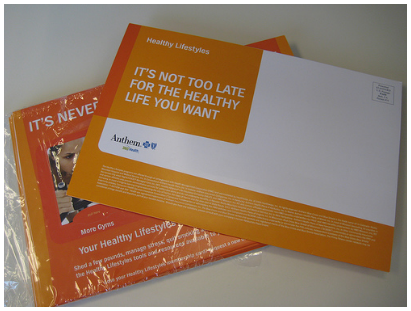

random control

writing

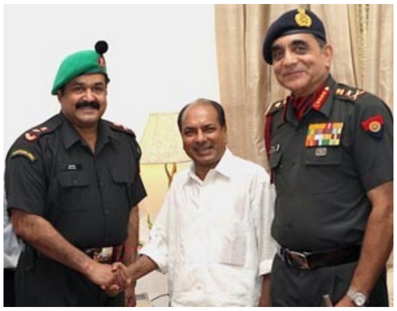

predicted

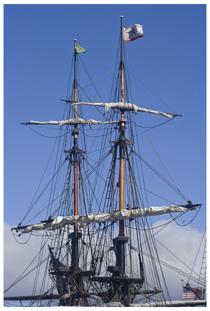

random control

address

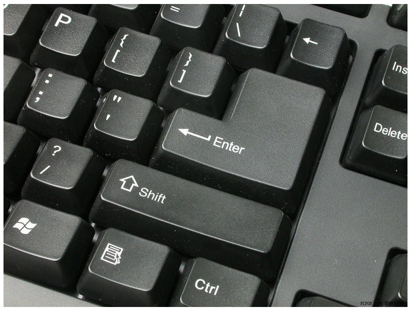

predicted

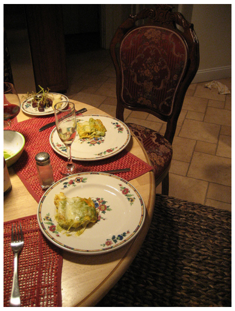

random control

killing

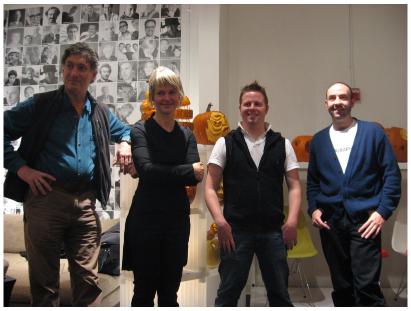

predicted

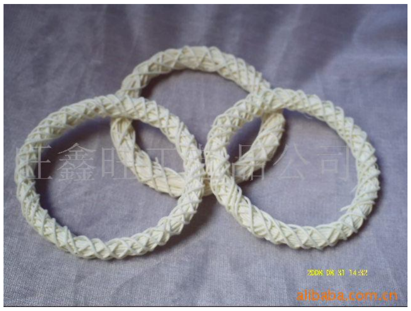

random control

greeting

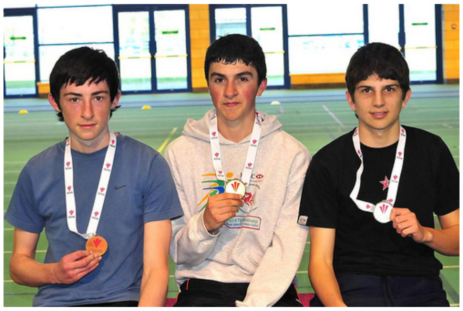

predicted

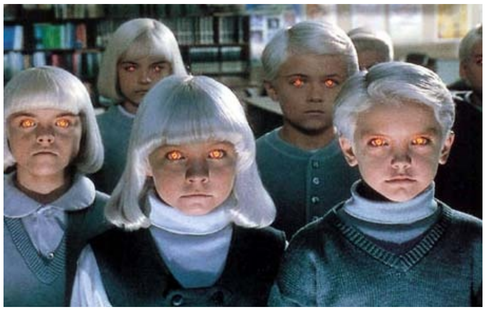

random control

paragraph

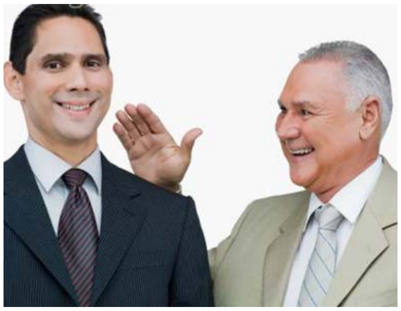

predicted

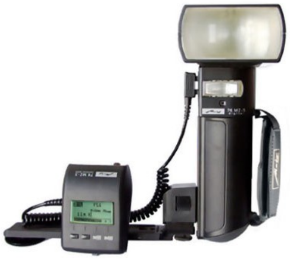

random control

bent

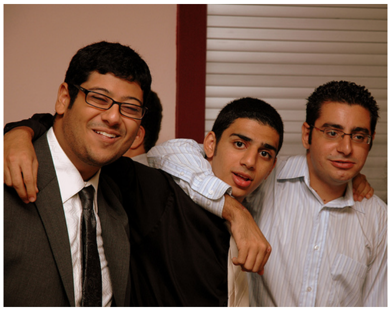

predicted

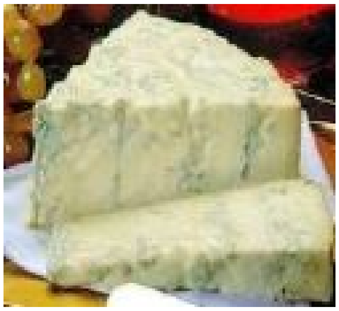

random control

dime

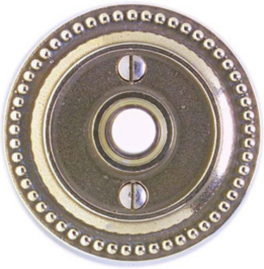

predicted

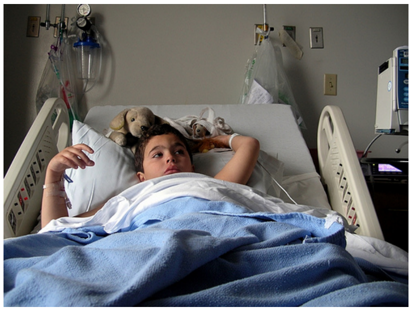

random control

filth

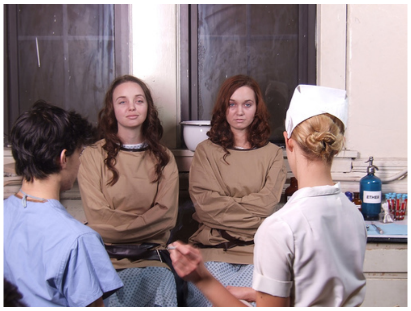

predicted

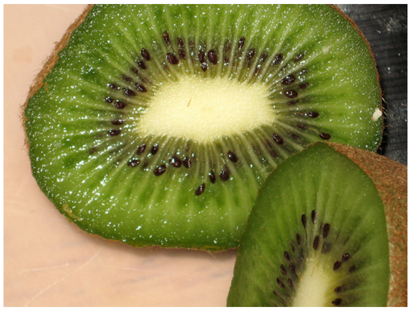

random control

mob

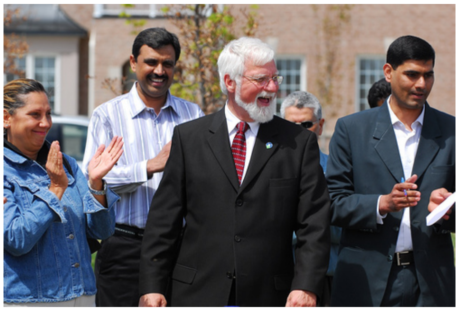

predicted

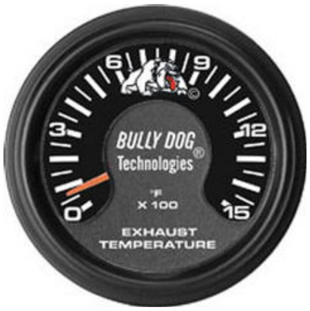

random control

sedative

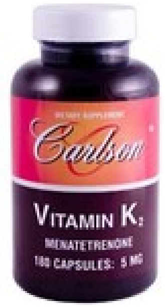

predicted

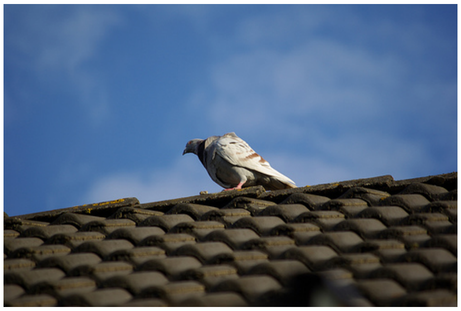

random control

plague

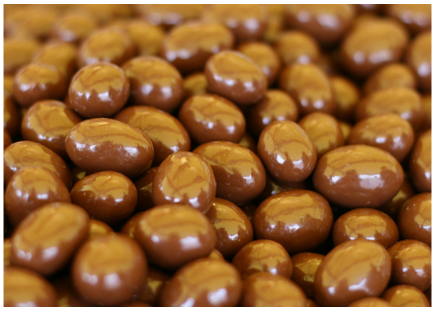

predicted

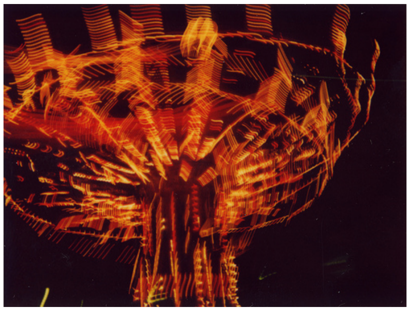

random control

petition

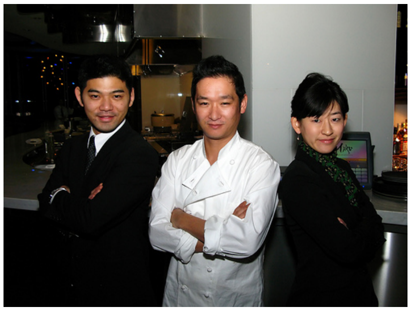

predicted

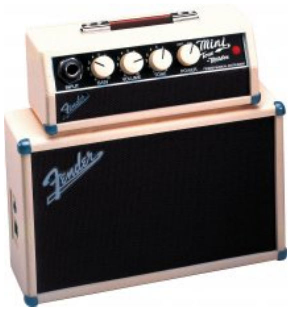

random control

asthma

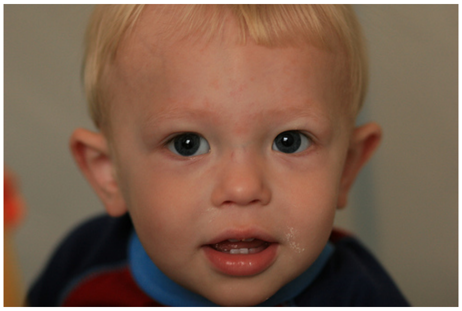

predicted

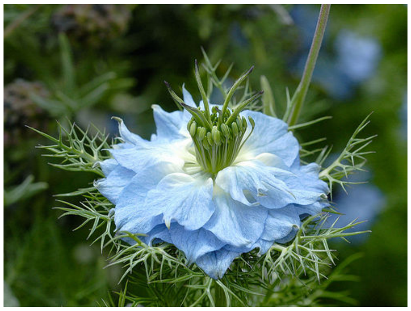

random control

scrap

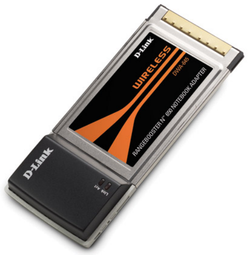

predicted

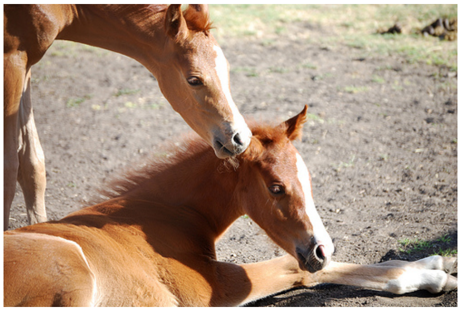

random control

gram

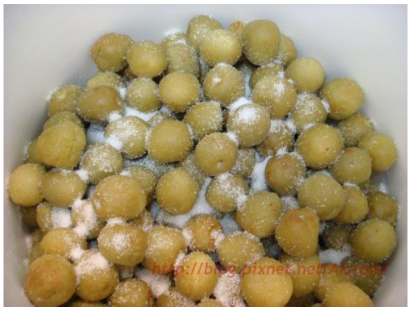

predicted

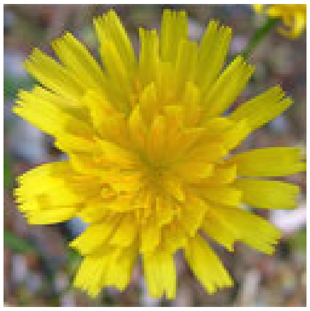

random control

biology

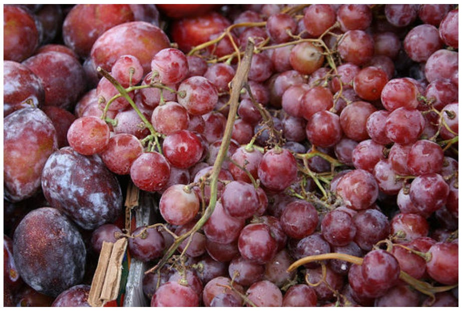

predicted

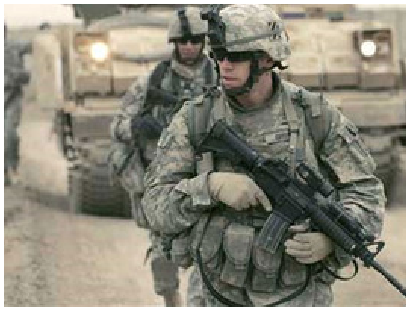

random control

operation

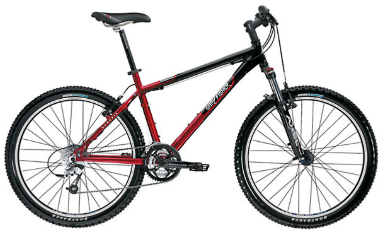

predicted

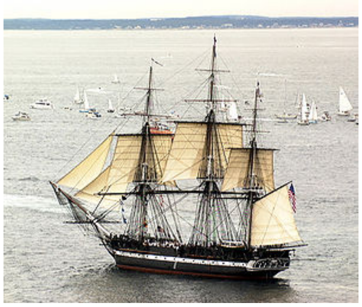

random control

bruise

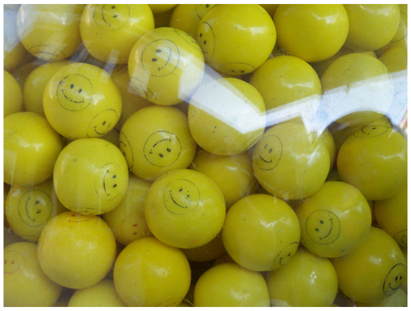

predicted

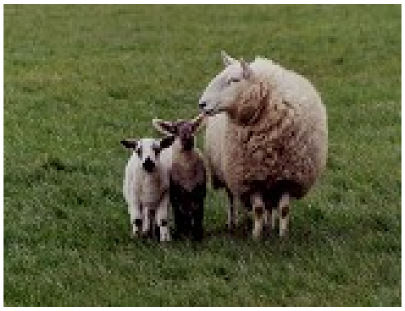

random control

symphony

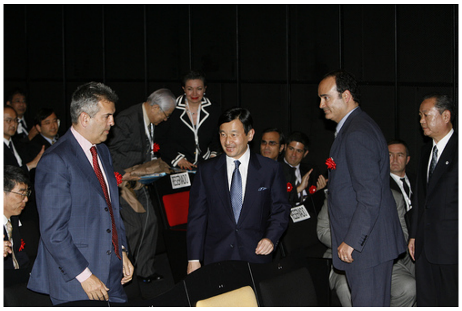

predicted

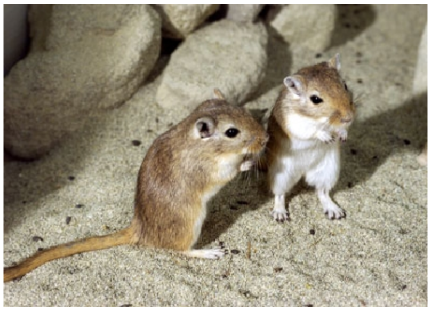

random control

aspirin

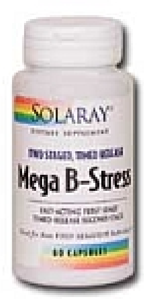

predicted

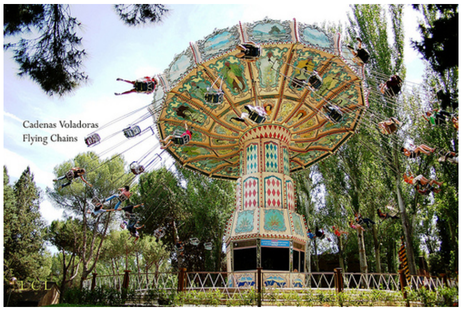

random control

strike

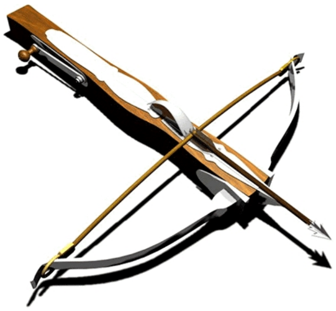

predicted

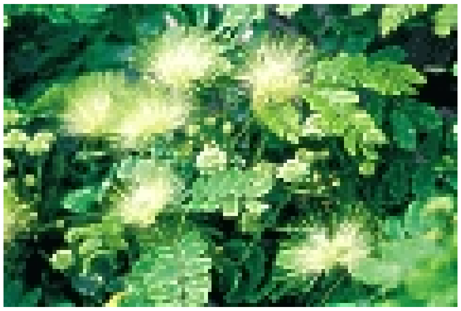

random control

bible

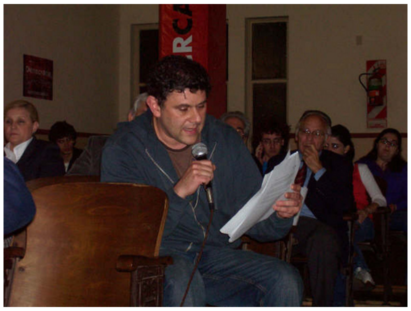

predicted

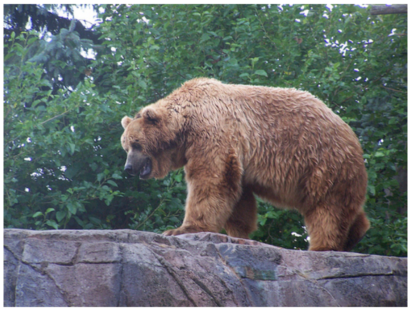

random control

land

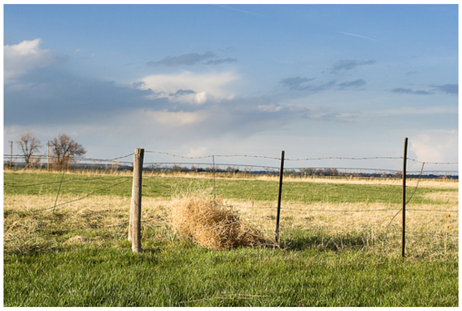

predicted

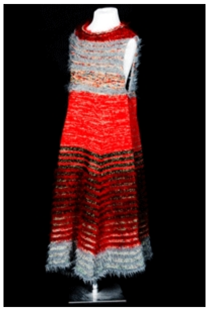

random control

poison

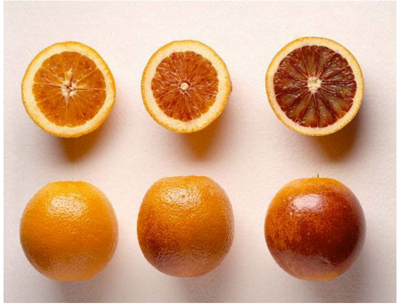

predicted

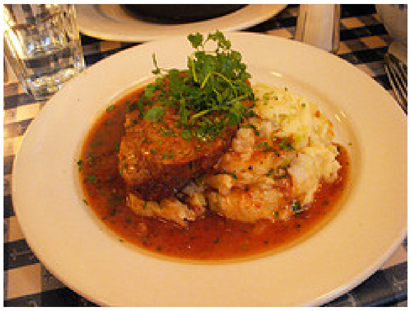

random control

flame

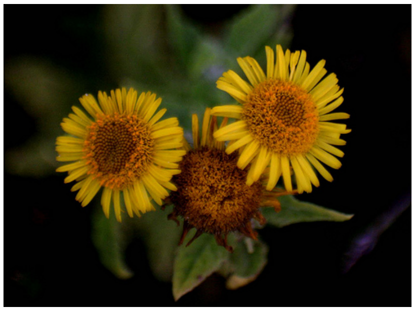

predicted

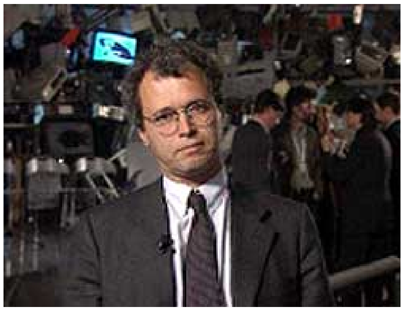

random control

playboy

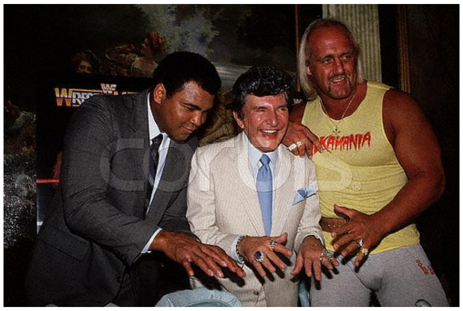

predicted

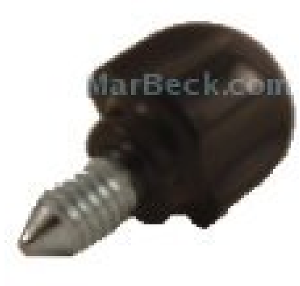

random control

traps

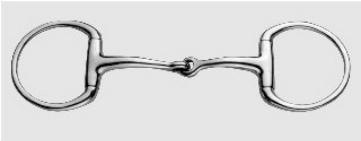

predicted

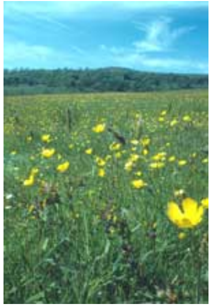

random control

scout

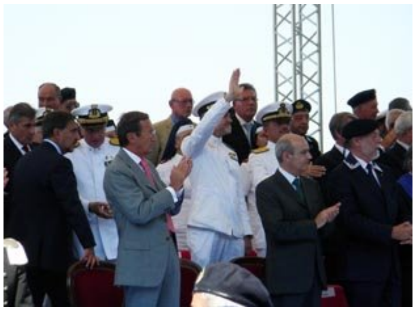

predicted

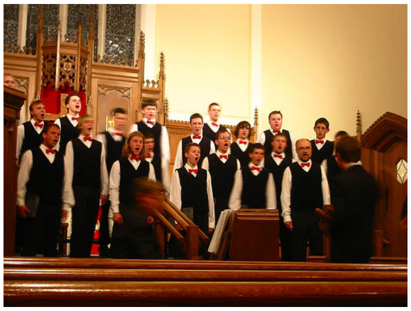

random control

cardboard

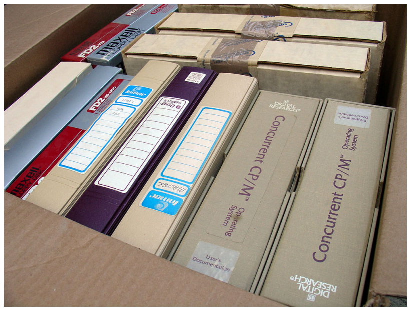

predicted

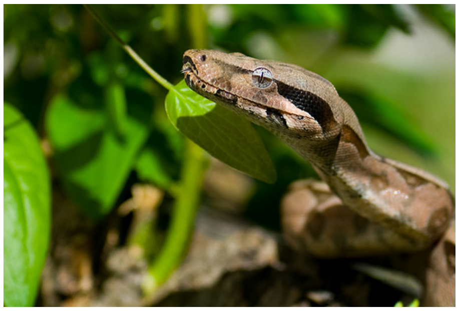

random control

archives

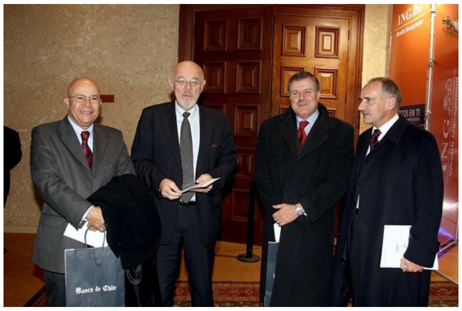

predicted

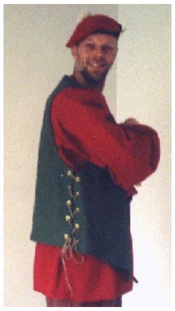

random control

cutting

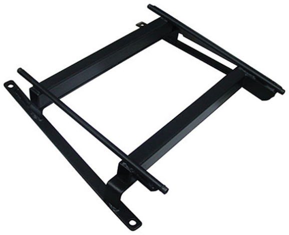

predicted

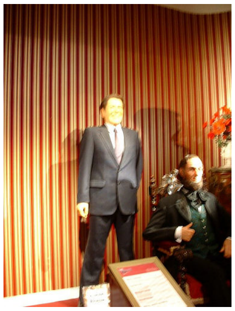

random control

snack

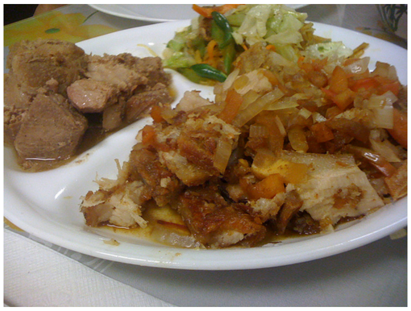

predicted

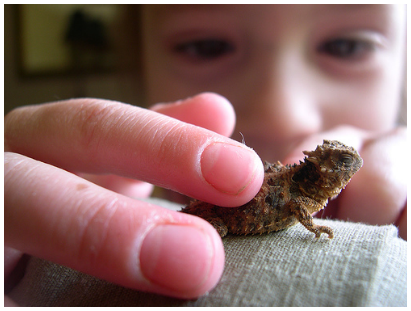

random control

piglet

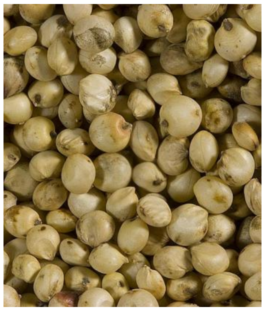

predicted

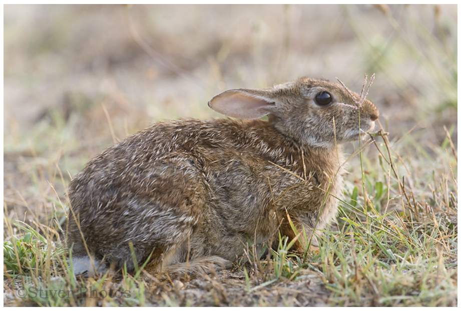

random control

warden

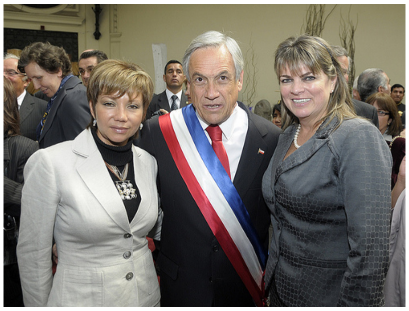

predicted

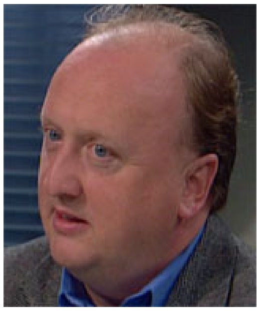

random control

chop

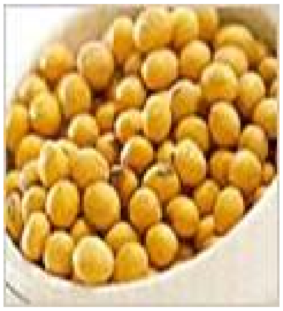

predicted

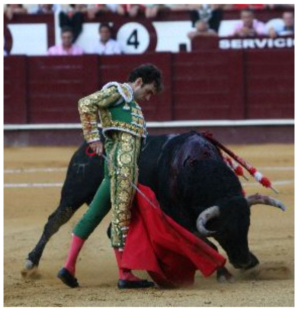

random control

critic

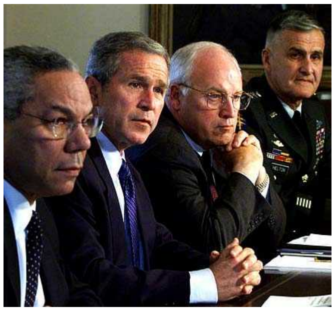

predicted

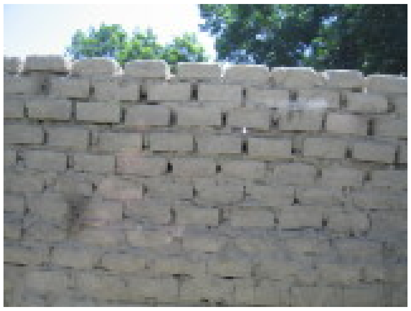

random control

muffin

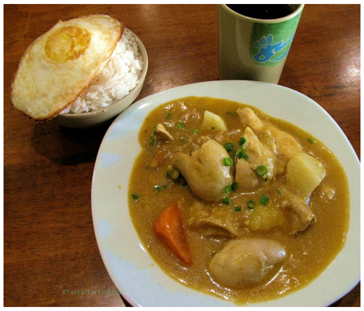

predicted

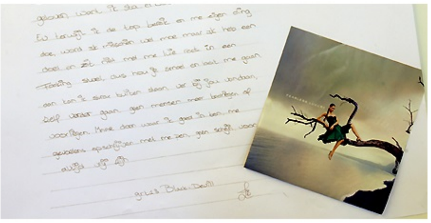

random control

assistant

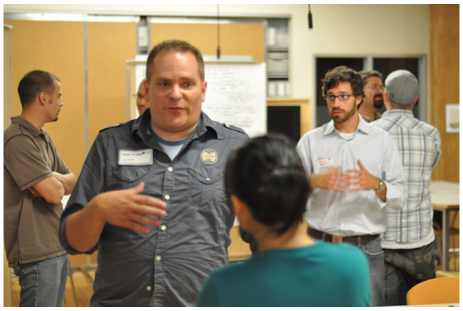

predicted

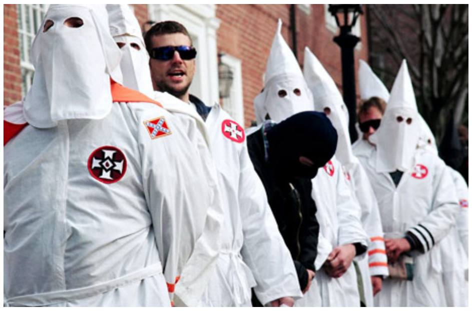

random control

paramedic

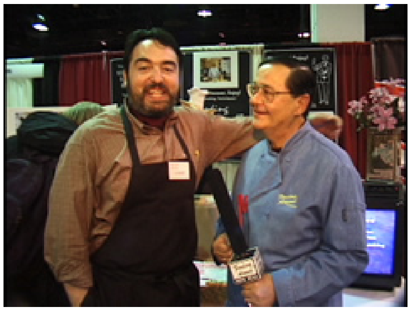

predicted

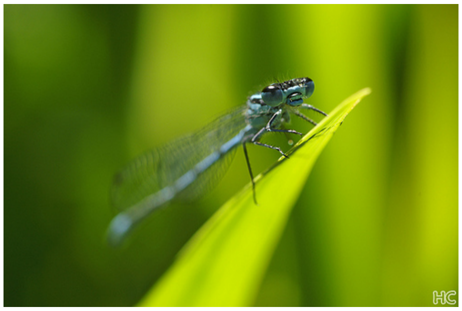

random control

auntie

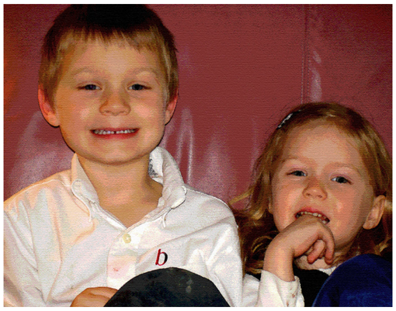

predicted

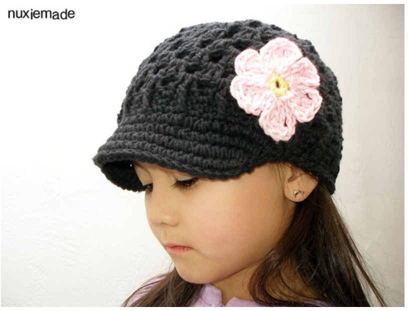

random control

preacher

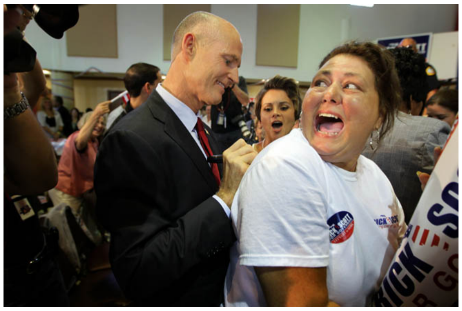

predicted

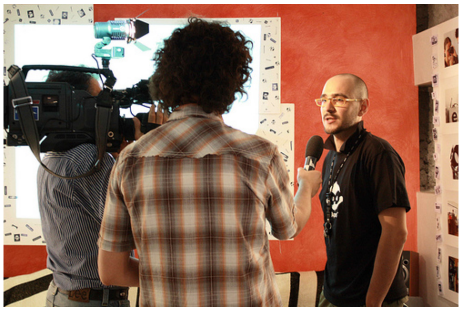

random control

skeleton

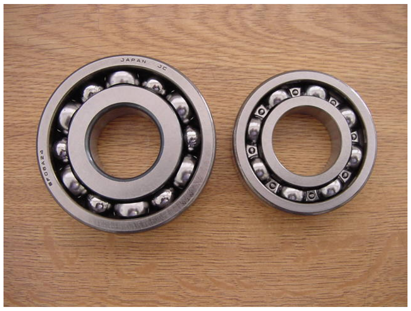

predicted

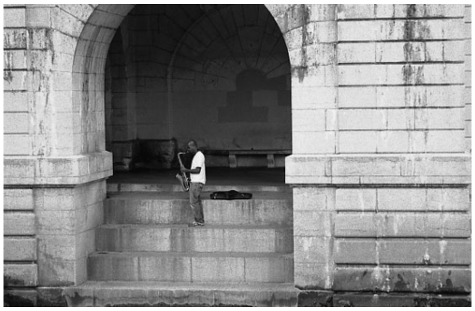

random control

lily

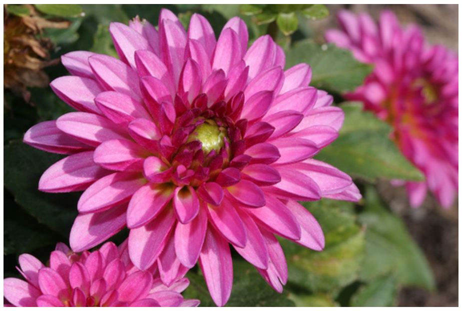

predicted

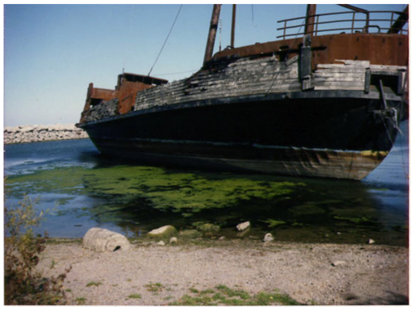

random control

leather

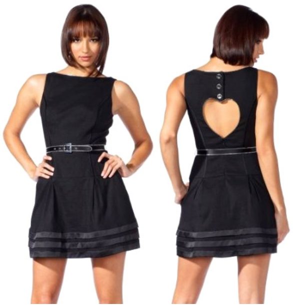

predicted

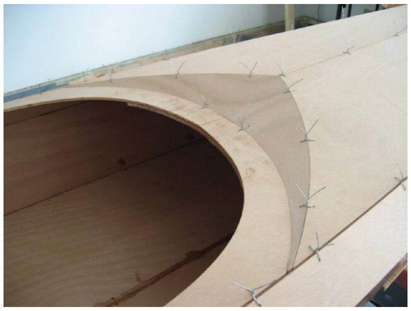

random control

ally

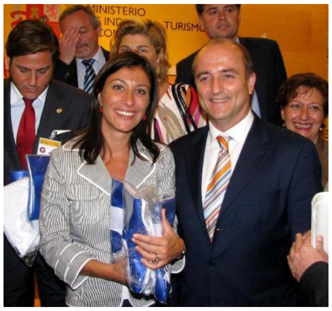

predicted

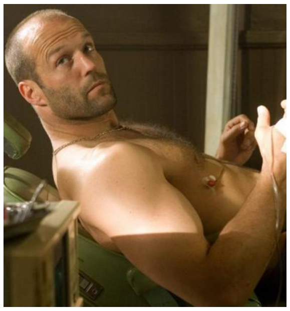

random control

bout

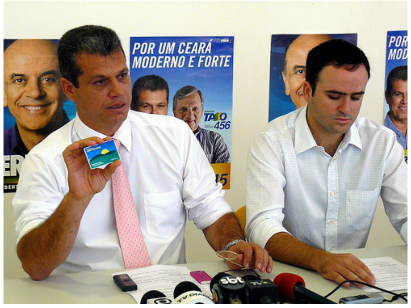

predicted

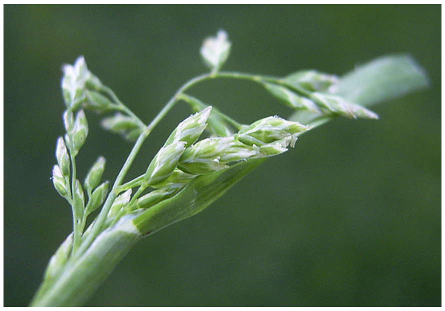

random control

breed

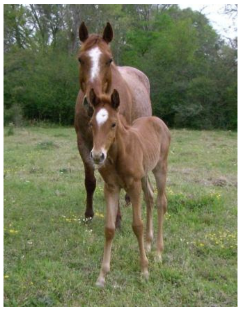

predicted

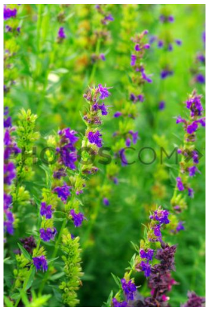

random control

childhood

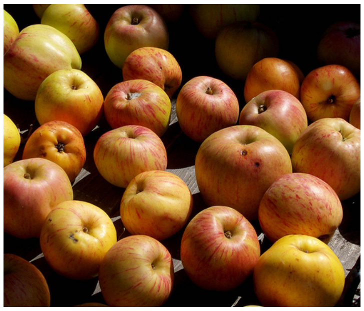

predicted

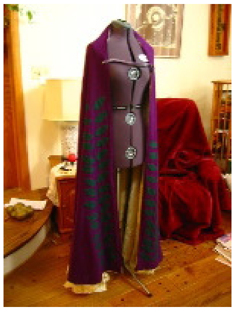

random control

corporal

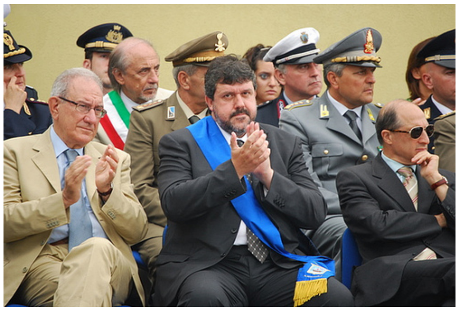

predicted

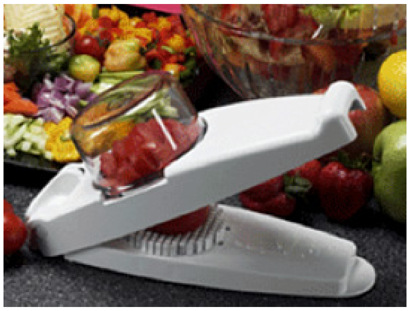

random control

defensive

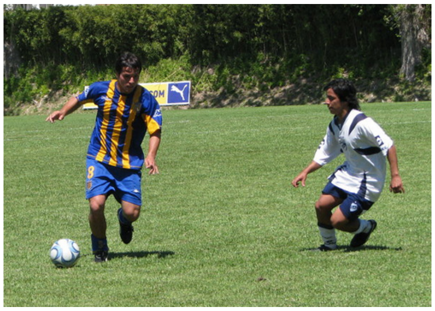

predicted

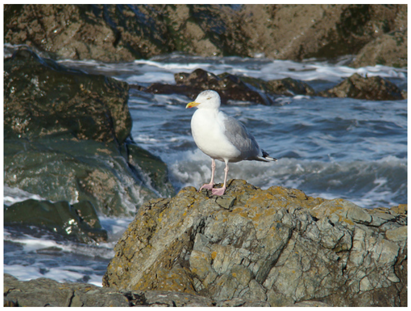

random control

department

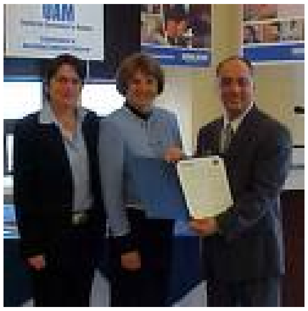

predicted

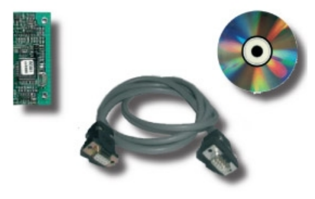

random control

fit

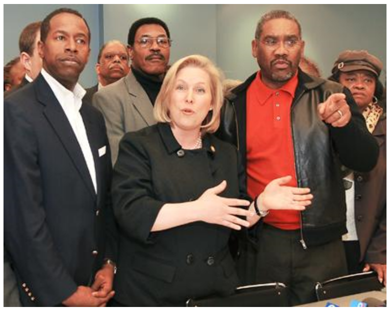

predicted

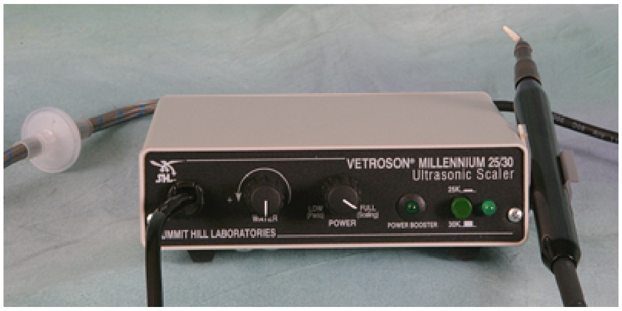

random control

fore

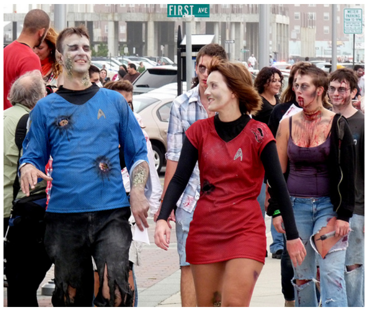

predicted

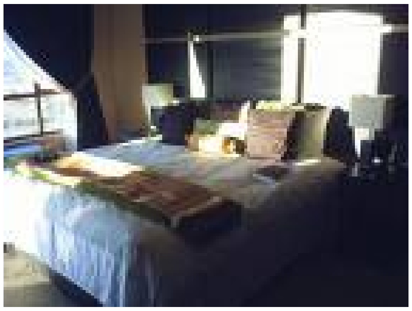

random control

gall

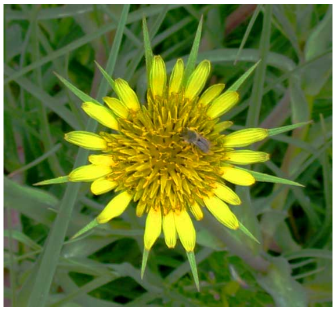

predicted

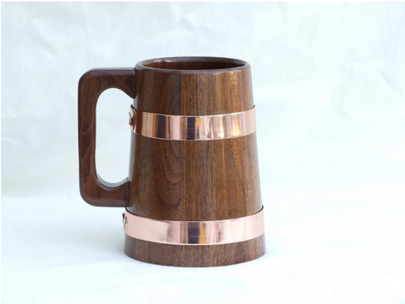

random control

graduating

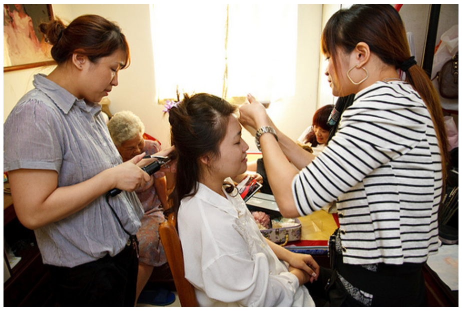

predicted

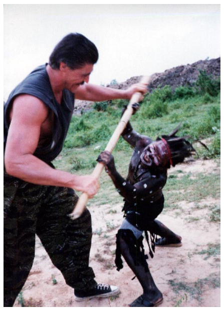

random control

journalism

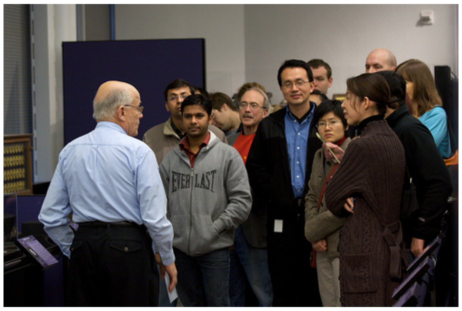

predicted

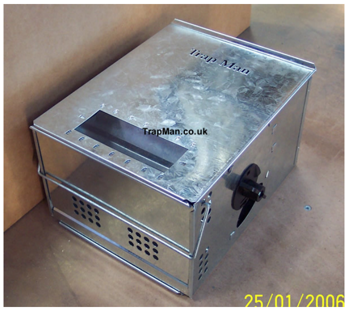

random control

main

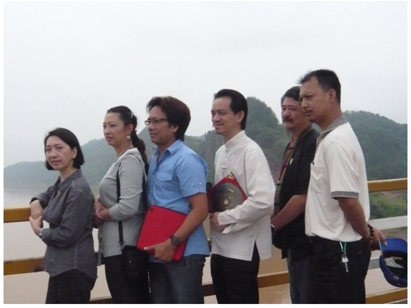

predicted

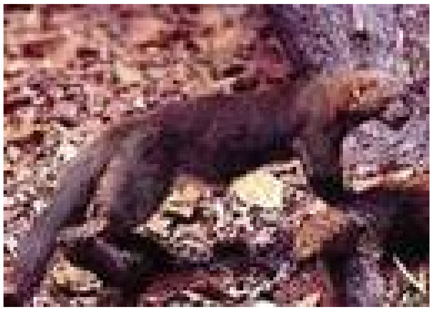

random control

member

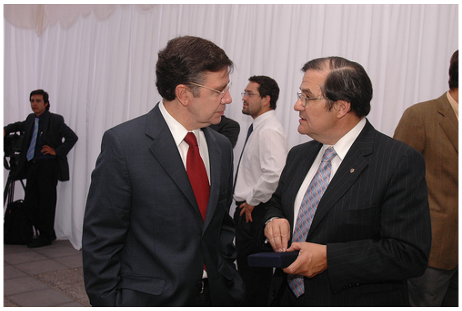

predicted

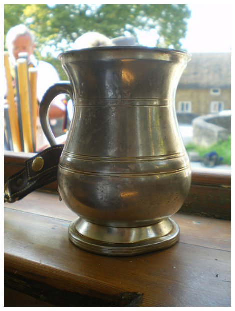

random control

outsider

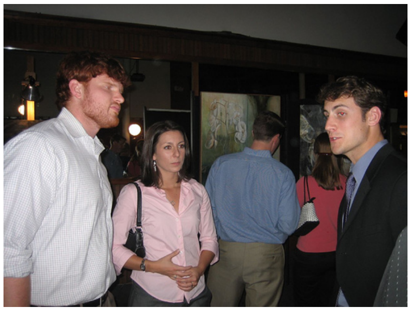

predicted

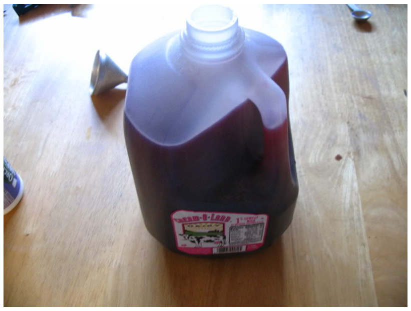

random control

position

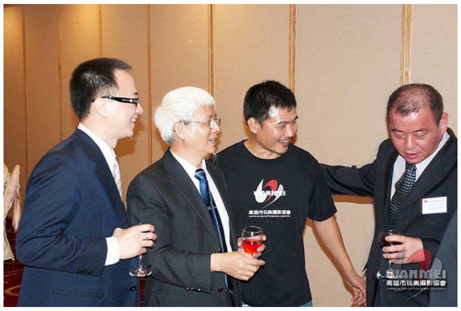

predicted

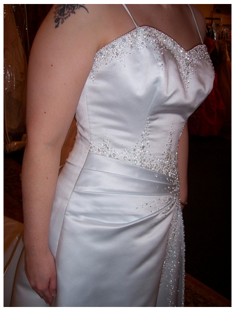

random control

salvage

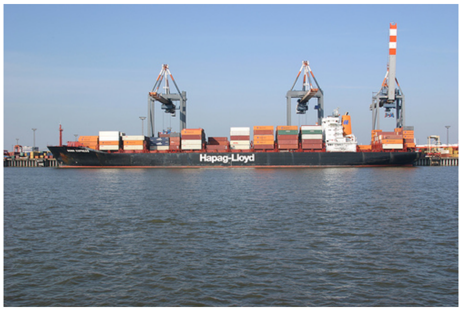

predicted

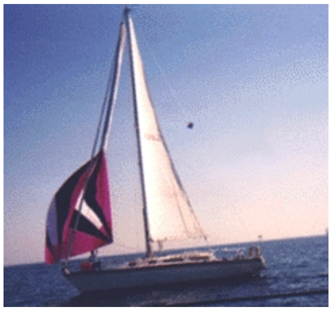

random control

scoundrel

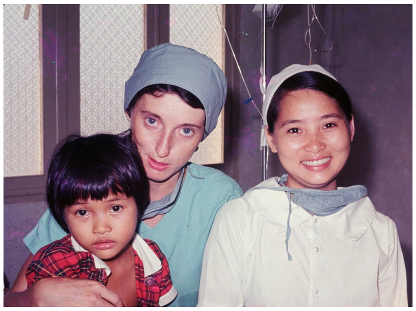

predicted

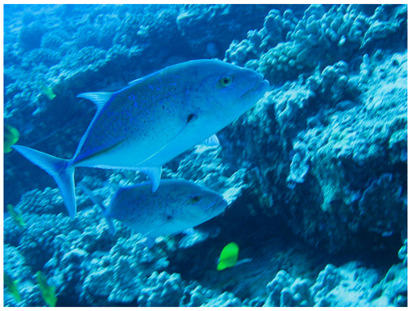

random control

sight

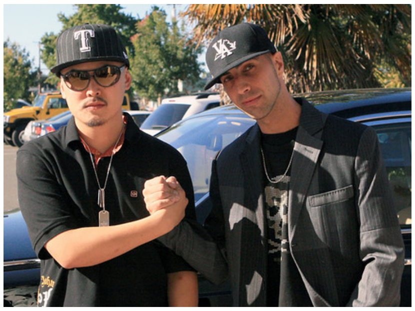

predicted

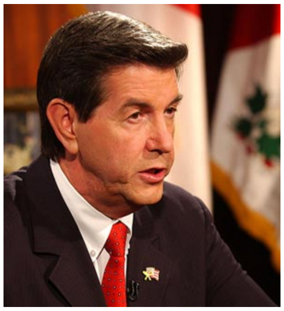

random control

starboard

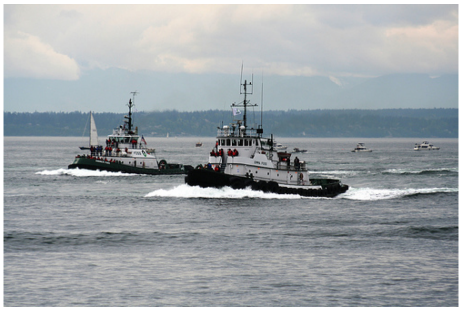

predicted

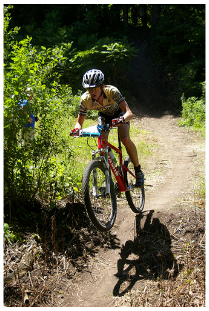

random control

vice

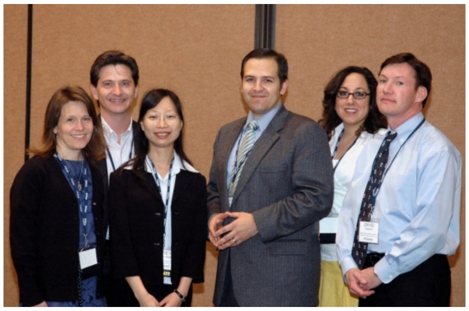

predicted

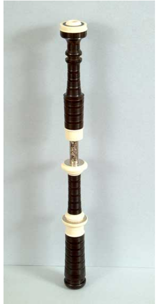

random control

vogue

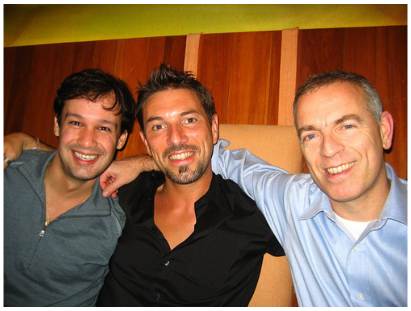

predicted

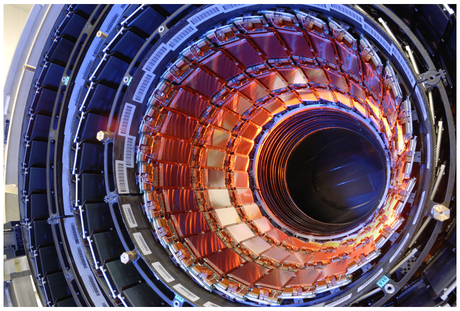

random control

wild

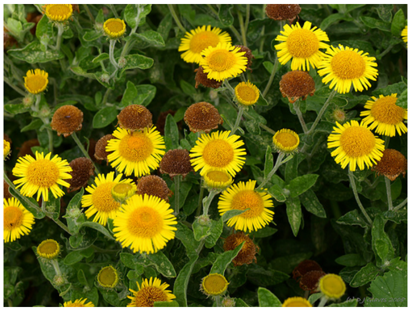

predicted

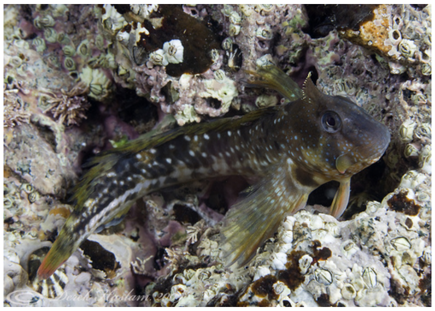

random control

virtue

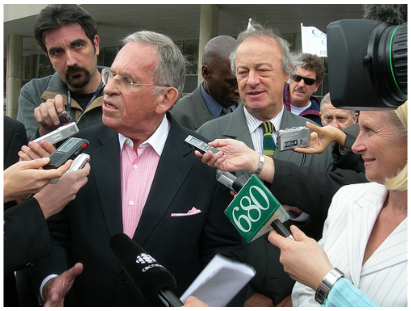

predicted

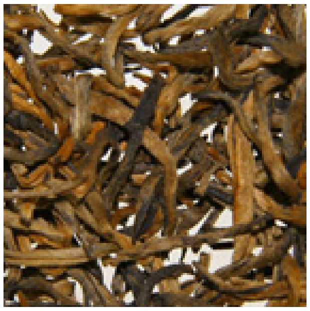

random control

perfection

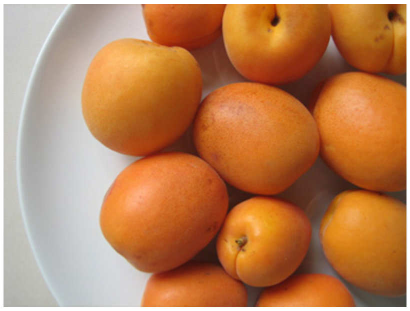

predicted

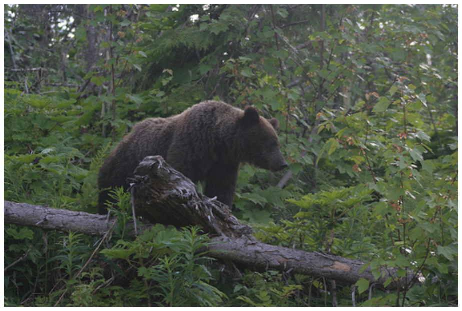

random control

awareness

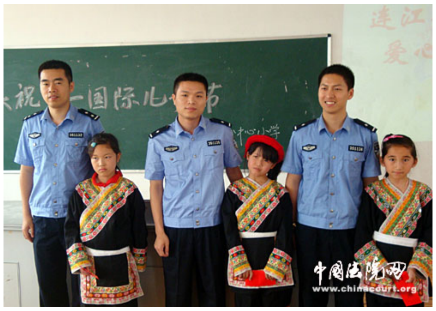

predicted

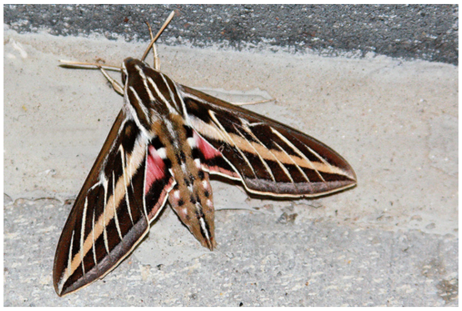

random control

madness

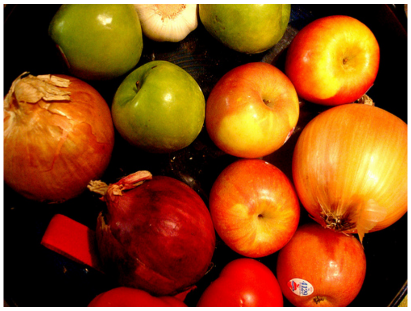

predicted

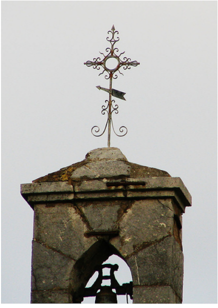

random control

determination

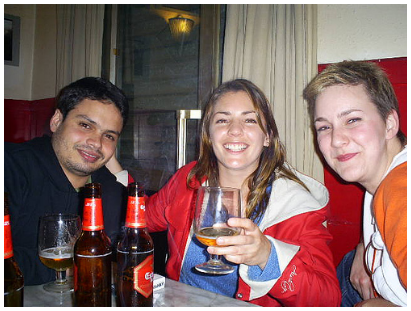

predicted

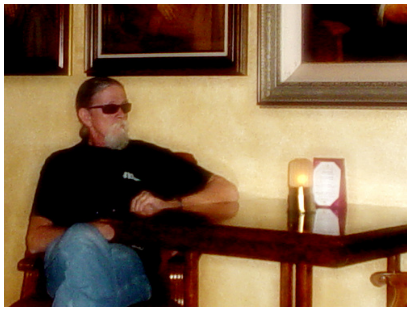

random control

metaphor

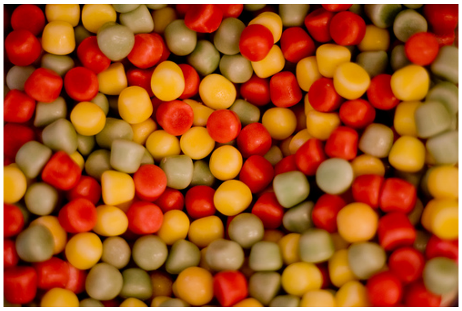

predicted

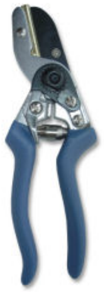

random control

curiosity

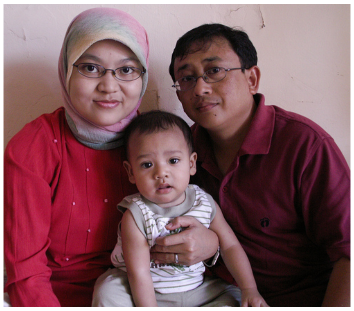

predicted

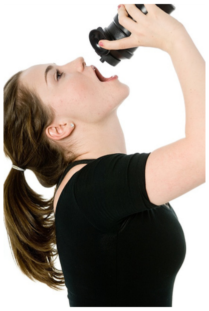

random control

destiny

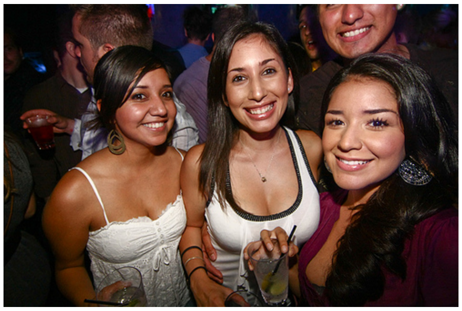

predicted

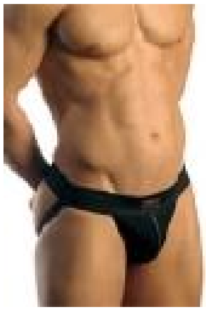

random control

opportunity

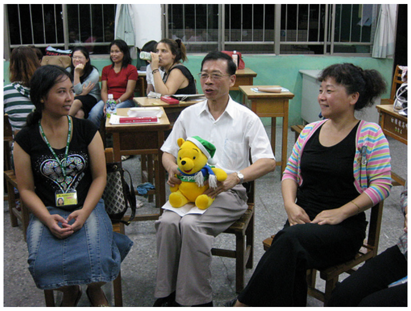

predicted

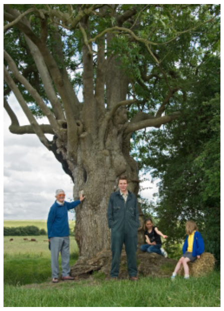

random control

aspect

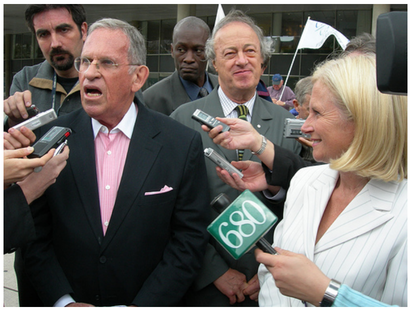

predicted

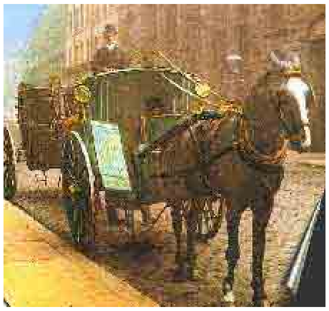

random control

experience

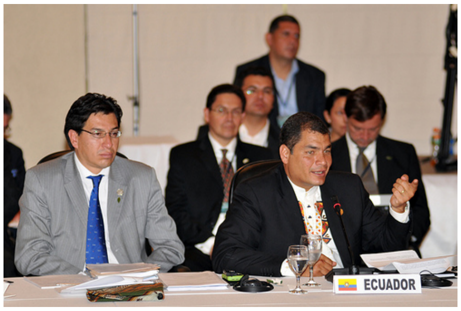

predicted

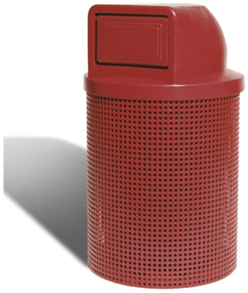

random control

jealousy

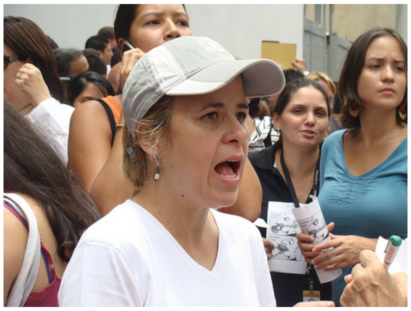

predicted

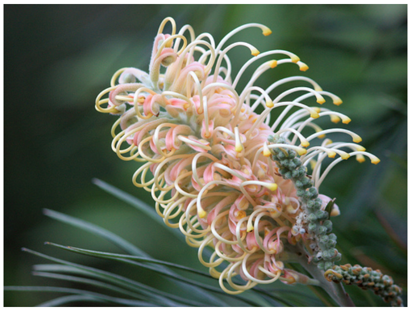

random control

funk

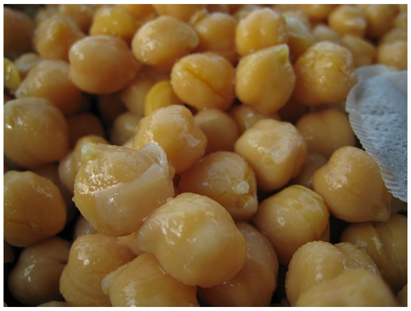

predicted

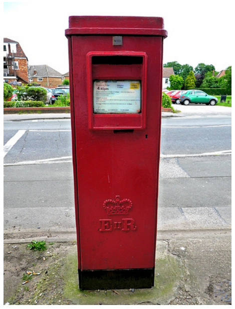

random control

meaning

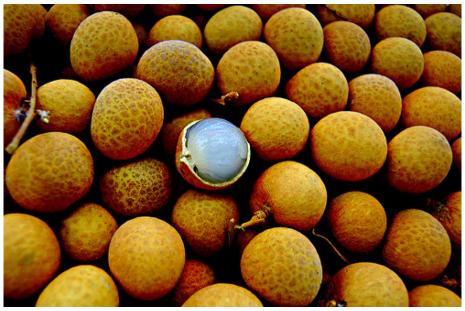

predicted

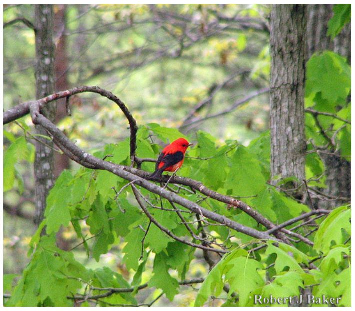

random control

luck

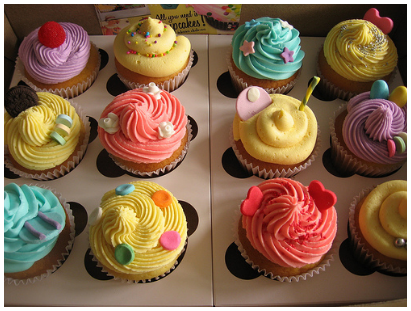

predicted

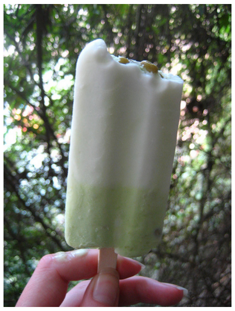

random control

contrary

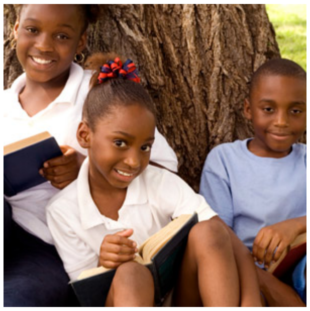

predicted

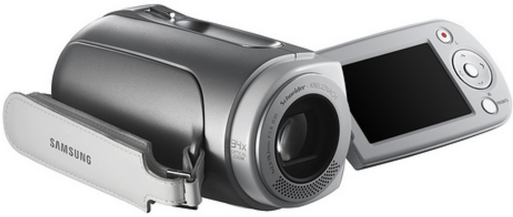

random control

sadness

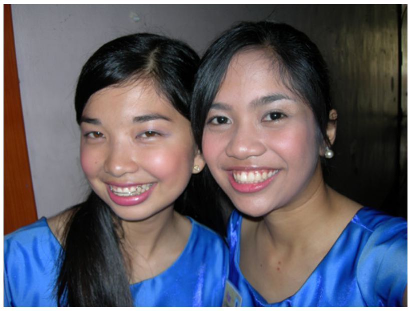

predicted

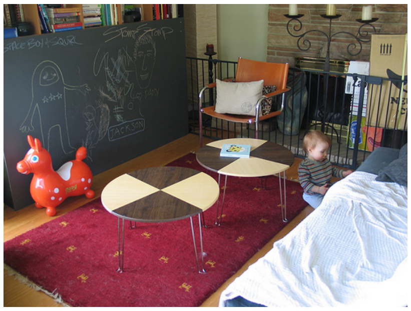

random control

conscience

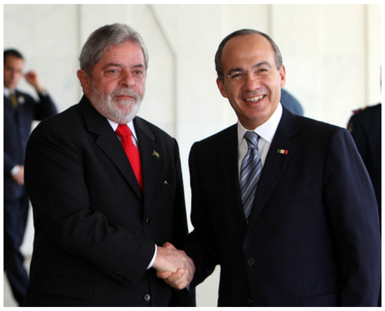

predicted

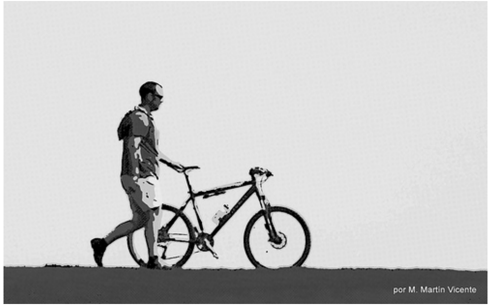

random control

inconvenience

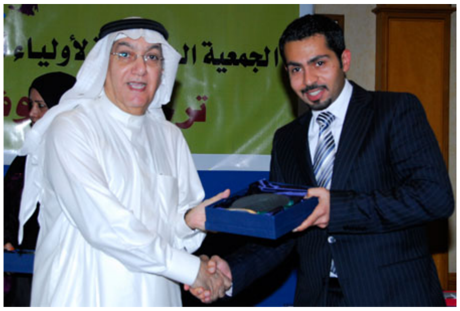

predicted

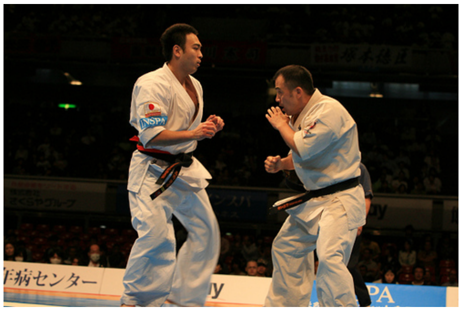

random control

boredom

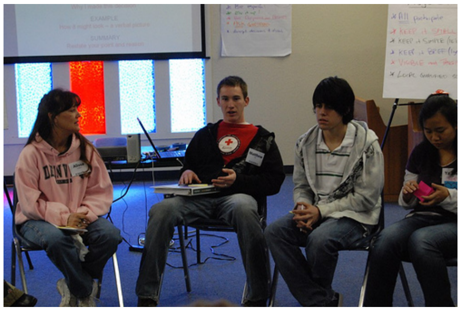

predicted

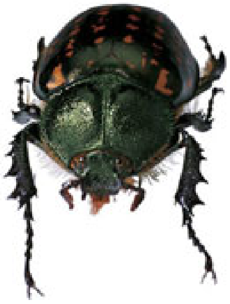

random control

debut

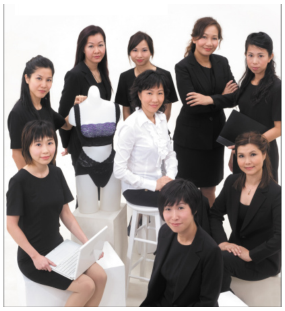

predicted

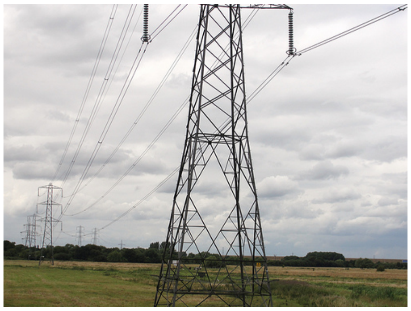

random control

worth

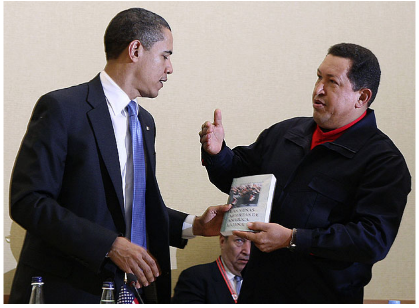

predicted

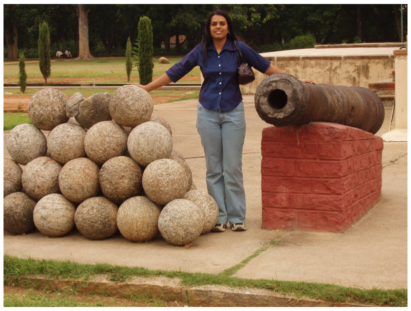

random control

principle

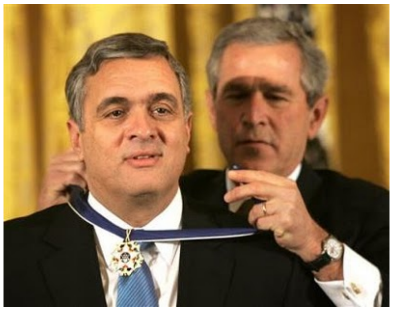

predicted

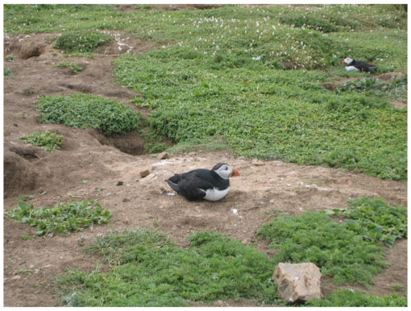

random control
