# Supplementary material for: Images of the unseen: extrapolating visual representations for abstract and concrete words in a data-driven computational model
Source: Psychol Res. 2020 Nov 12;86(8):2512–32. doi: 10.1007/s00426-020-01429-7 (PMC9674750; doi:10.1007/s00426-020-01429-7)
Supplement: Supplementary file 3 — Supplementary material 3 (pdf 79133 KB) [file 426_2020_1429_MOESM3_ESM.pdf]

Experiment 3 (prototype model)

nausea

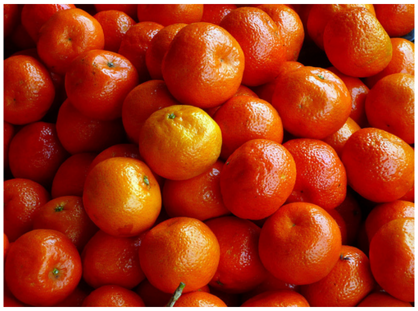

predicted

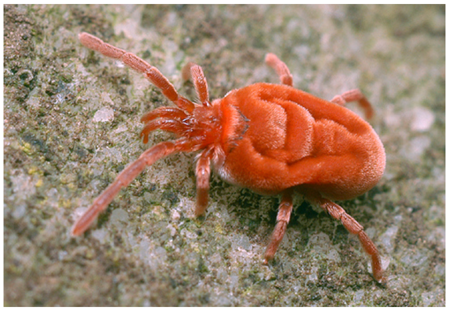

random control

appendix

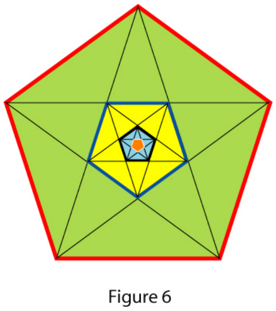

predicted

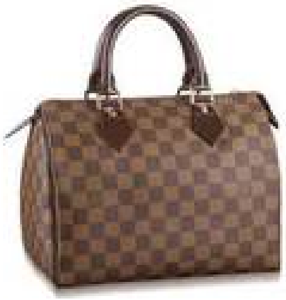

random control

moron

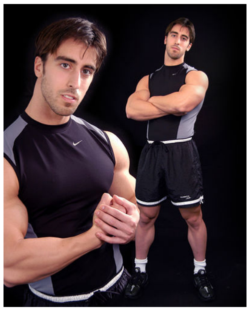

predicted

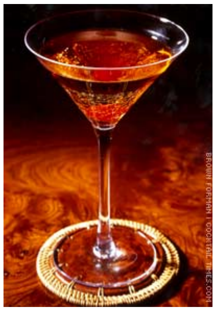

random control

question

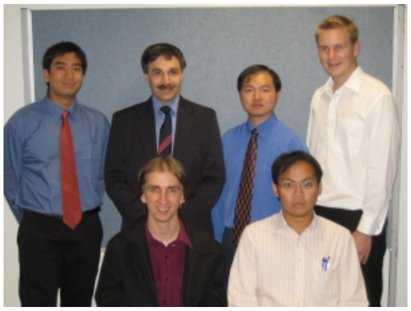

predicted

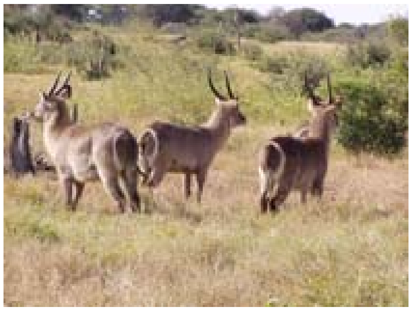

random control

breed

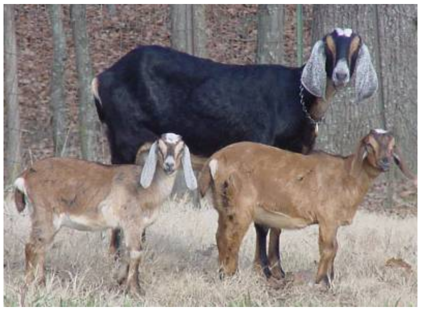

predicted

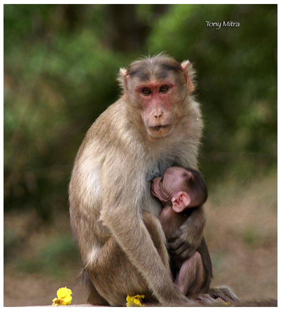

random control

dispatch

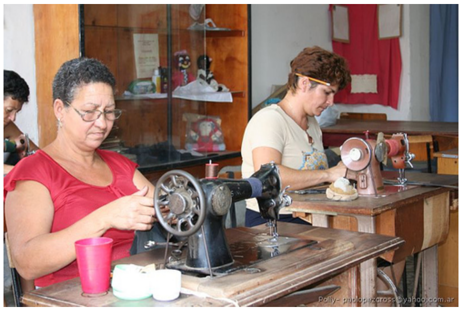

predicted

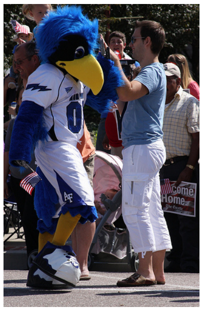

random control

behalf

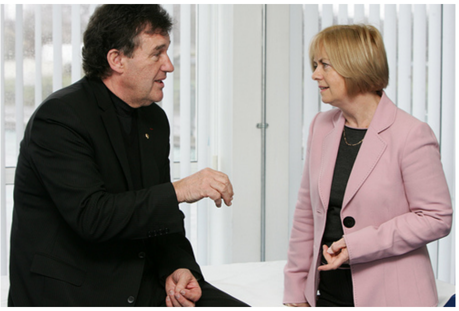

predicted

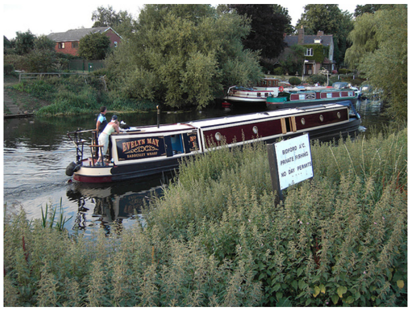

random control

guardian

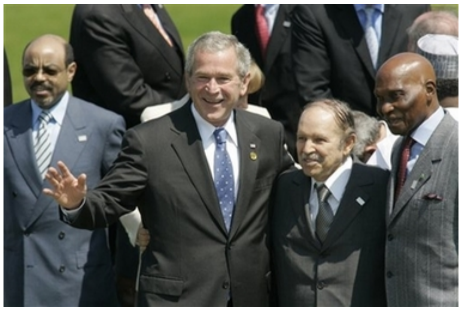

predicted

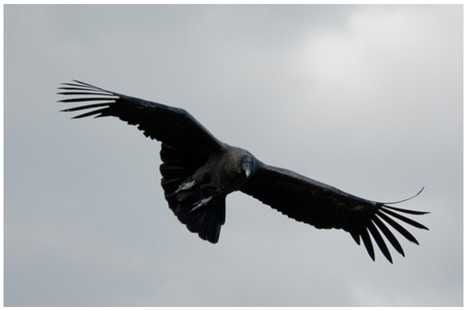

random control

temp

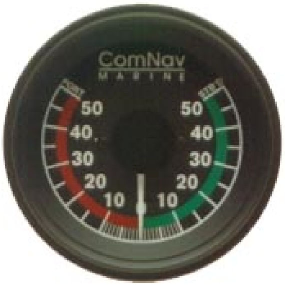

predicted

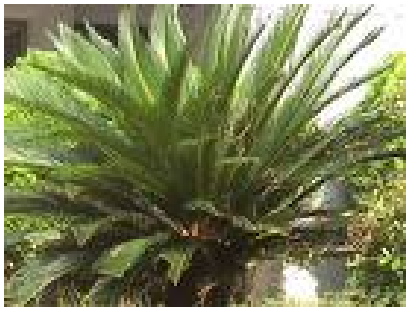

random control

initiative

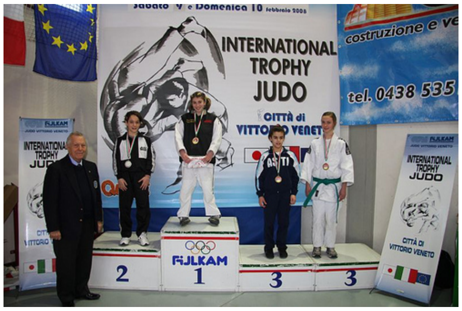

predicted

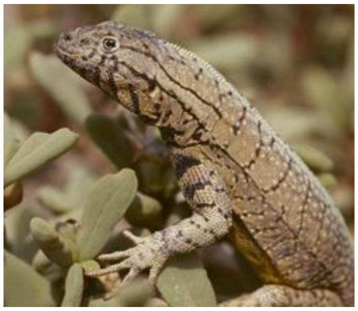

random control

communion

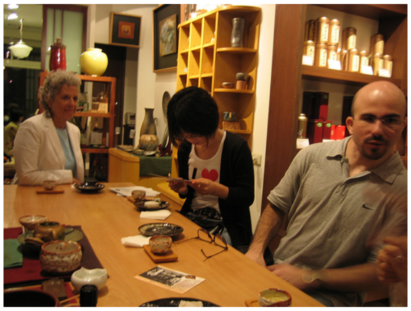

predicted

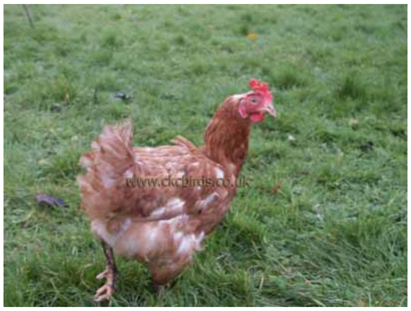

random control

inning

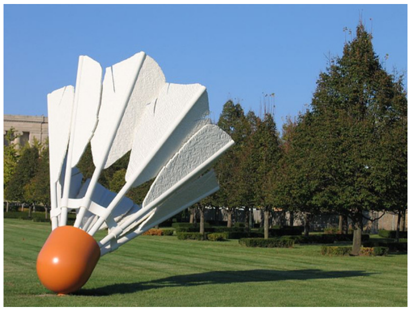

predicted

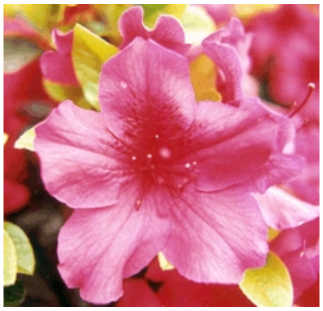

random control

jealousy

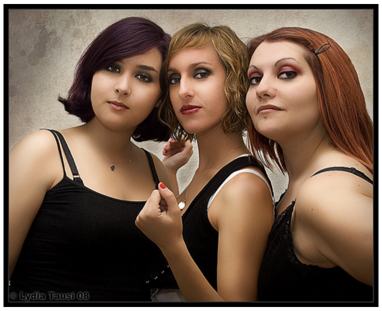

predicted

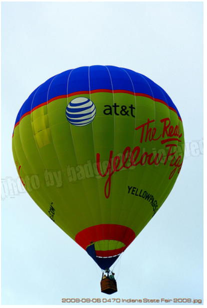

random control

conspiracy

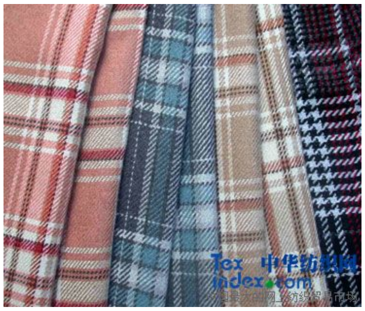

predicted

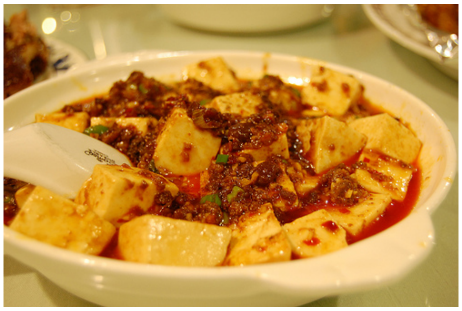

random control

suspense

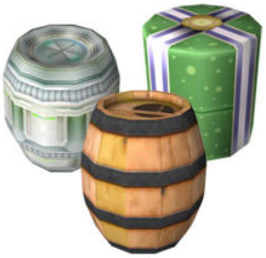

predicted

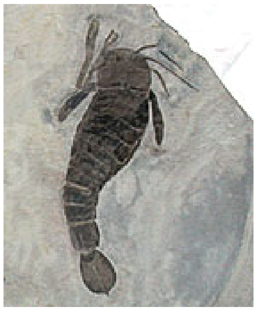

random control

thousand

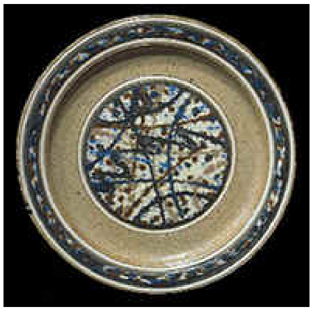

predicted

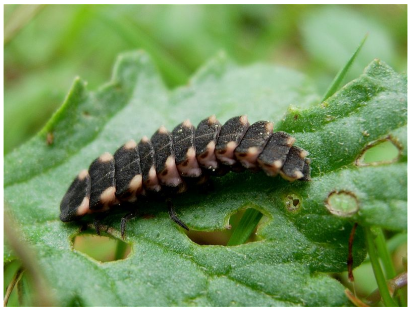

random control

classic

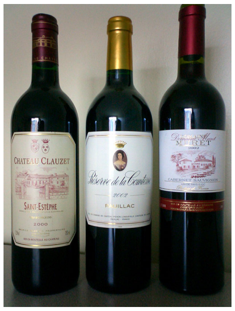

predicted

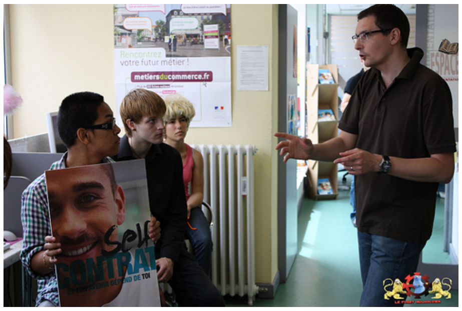

random control

ticket

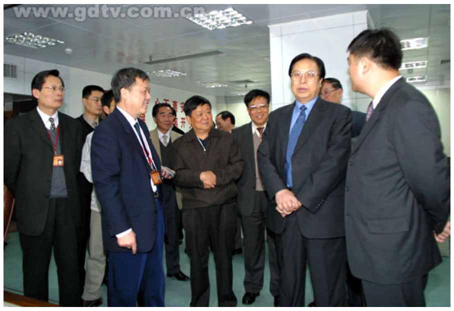

predicted

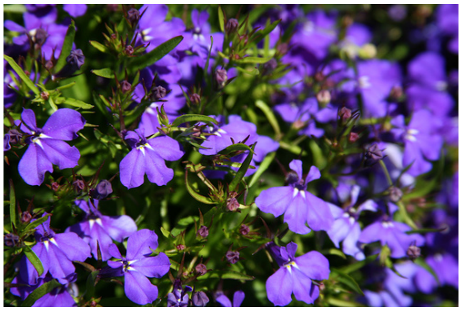

random control

diversion

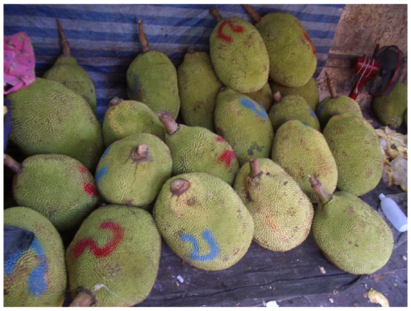

predicted

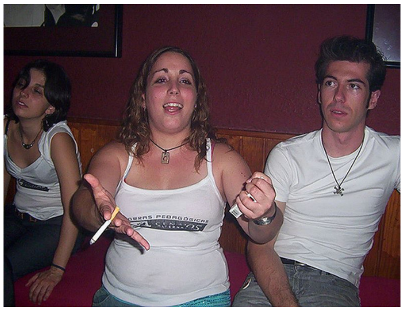

random control

southwest

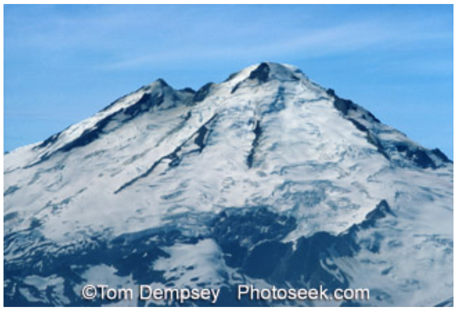

predicted

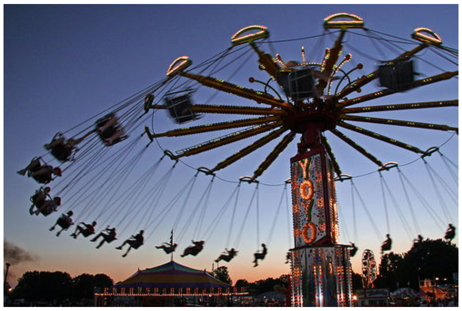

random control

research

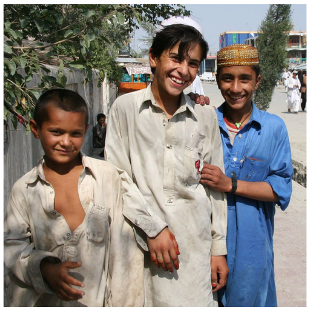

predicted

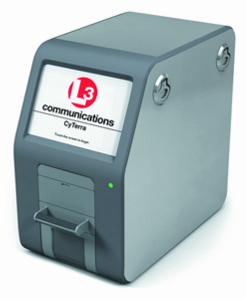

random control

horror

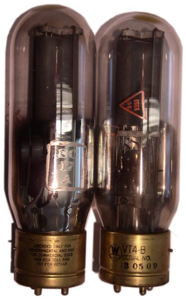

predicted

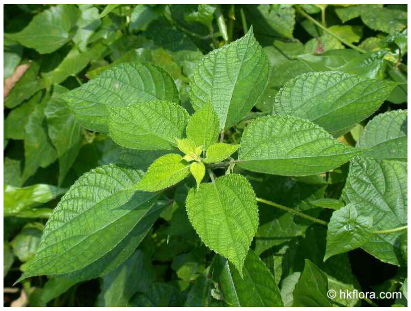

random control

corporation

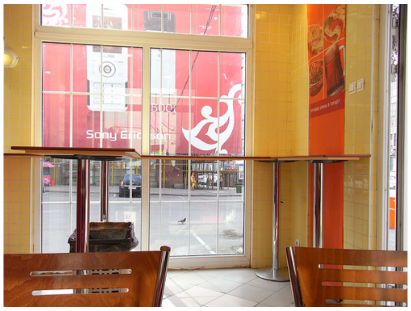

predicted

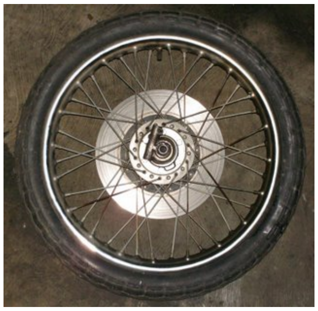

random control

rascal

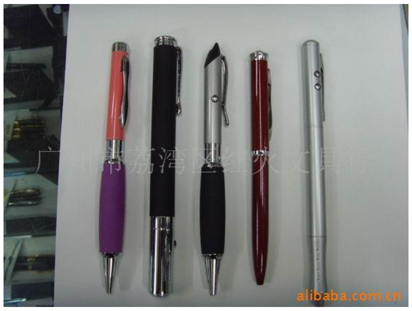

predicted

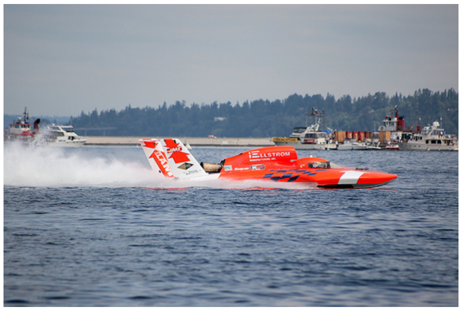

random control

parasite

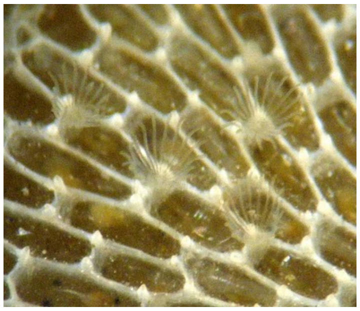

predicted

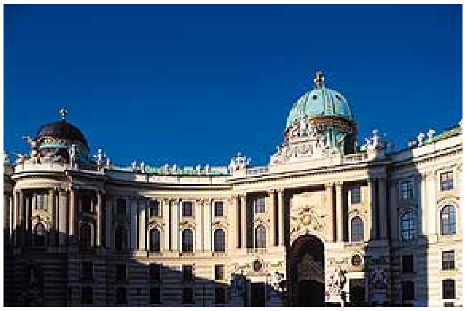

random control

direction

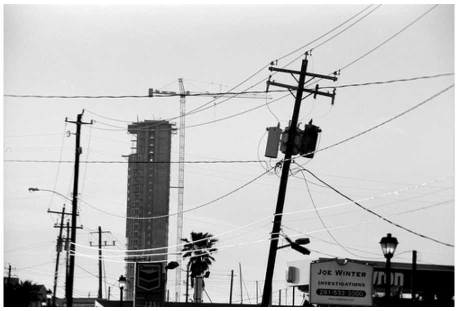

predicted

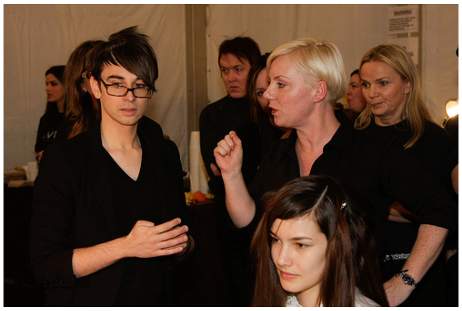

random control

speaking

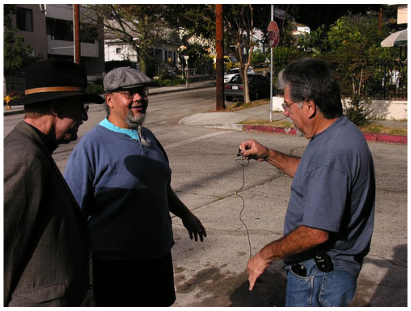

predicted

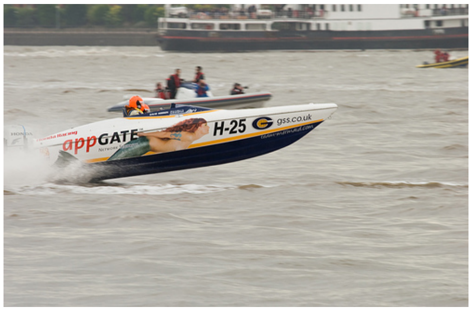

random control

thrill

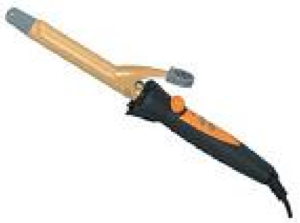

predicted

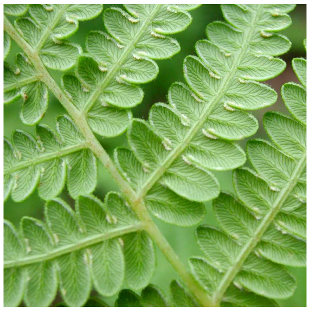

random control

executive

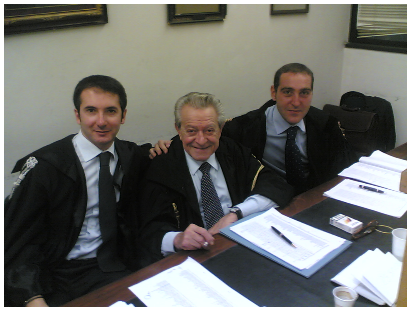

predicted

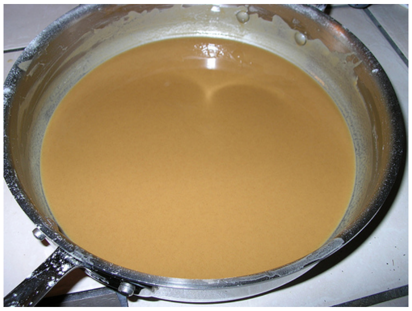

random control

freeman

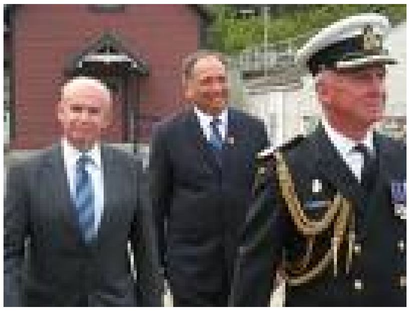

predicted

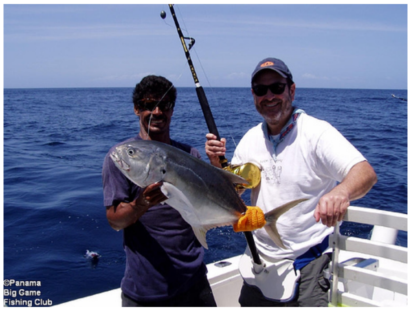

random control

religion

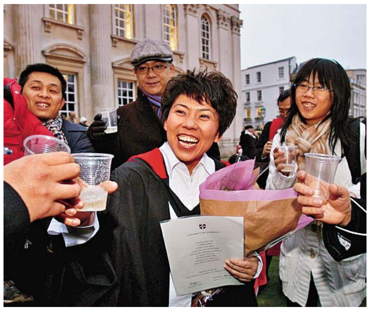

predicted

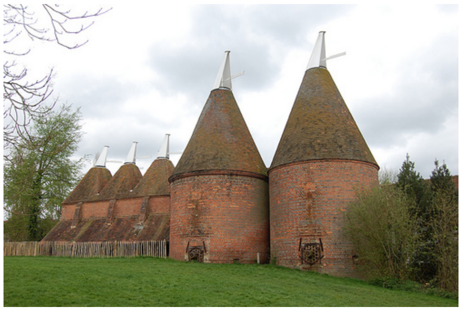

random control

tale

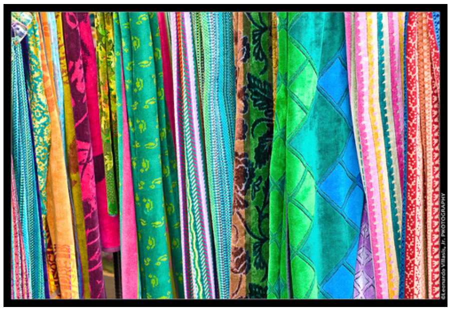

predicted

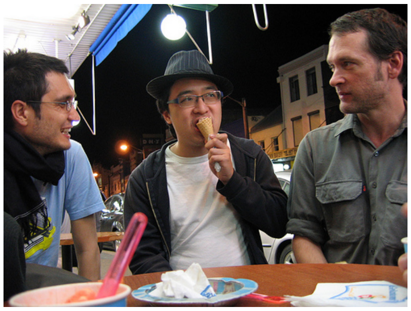

random control

energy

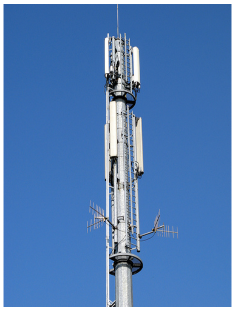

predicted

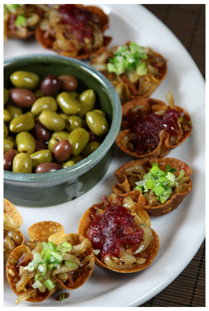

random control

army

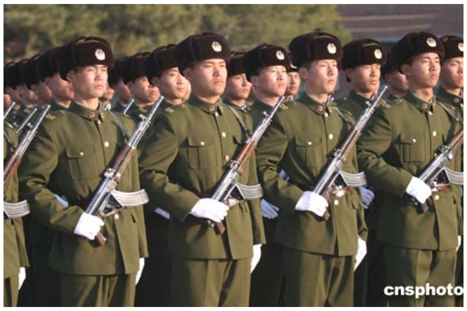

predicted

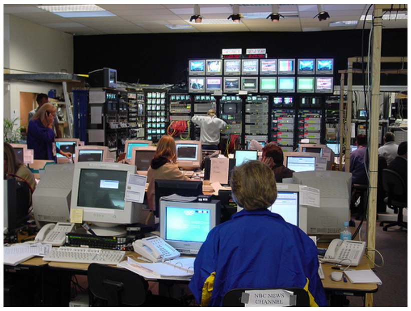

random control

paradise

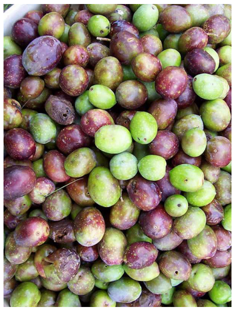

predicted

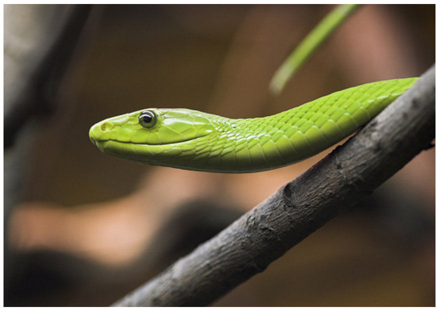

random control

shooting

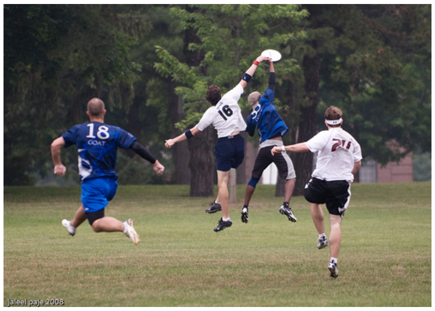

predicted

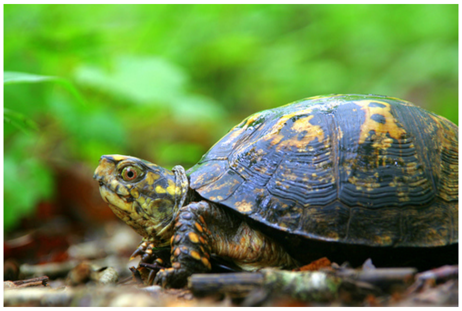

random control

choosing

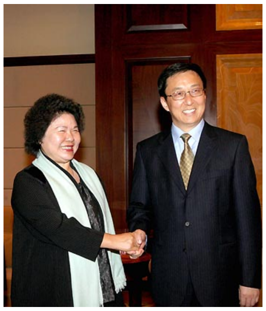

predicted

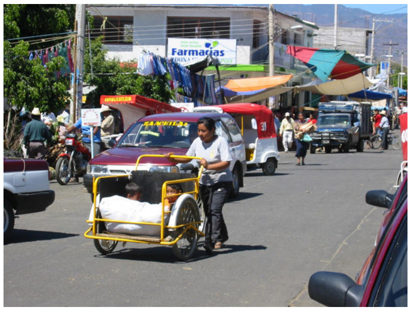

random control

tow

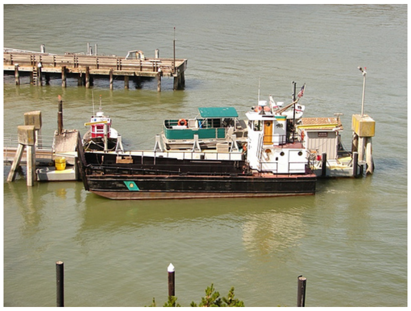

predicted

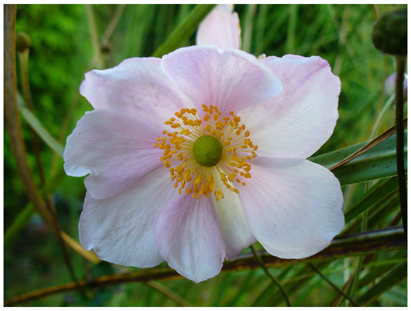

random control

scenery

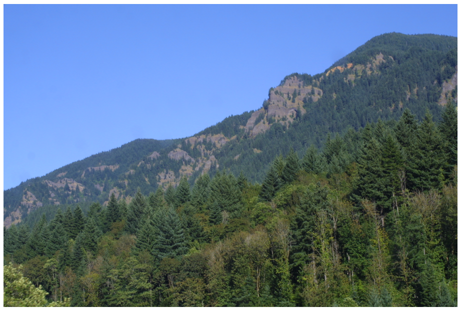

predicted

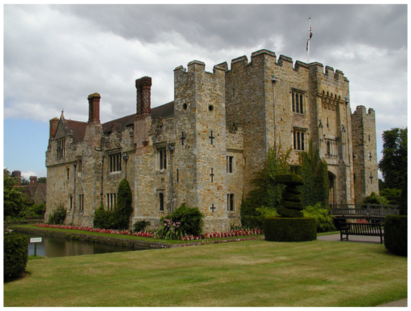

random control

resume

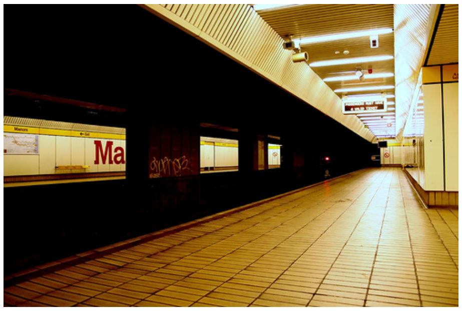

predicted

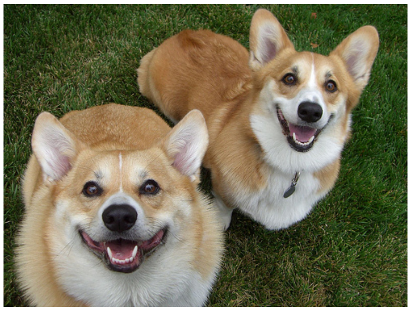

random control

semen

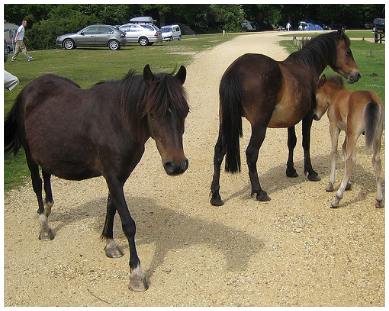

predicted

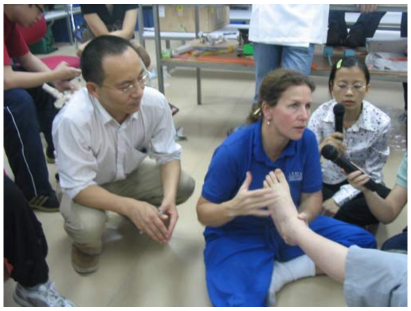

random control

engagement

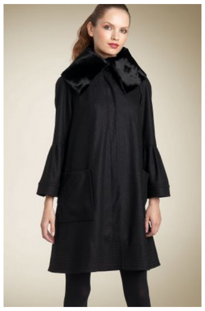

predicted

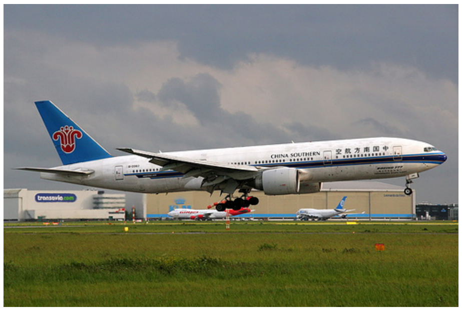

random control

heavy

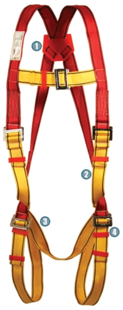

predicted

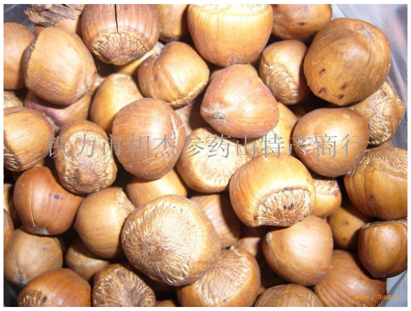

random control

workshop

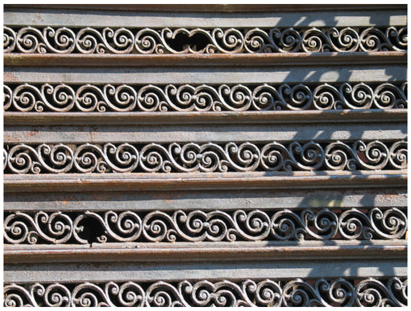

predicted

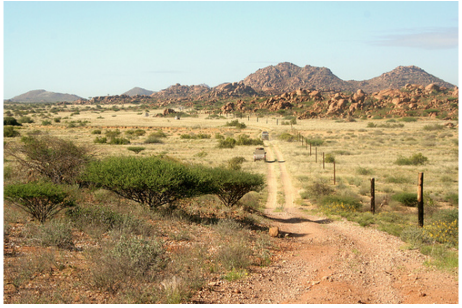

random control

voting

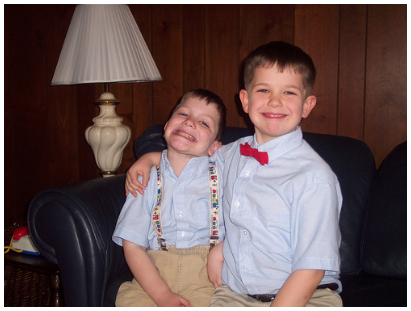

predicted

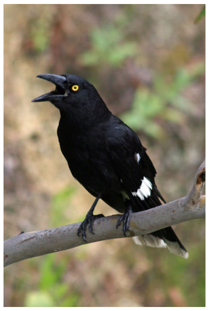

random control

fingerprint

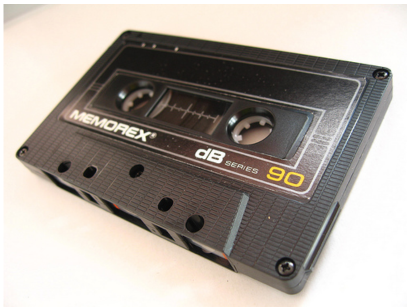

predicted

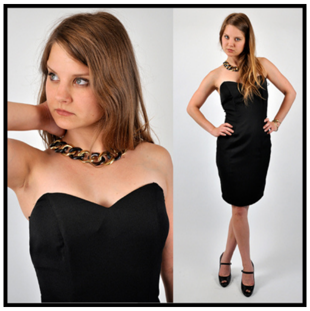

random control

hooray

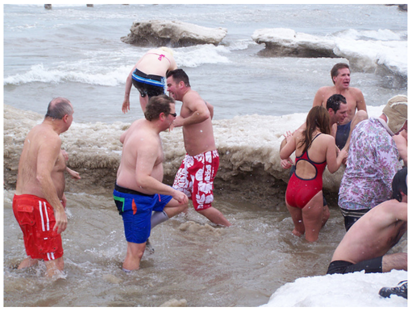

predicted

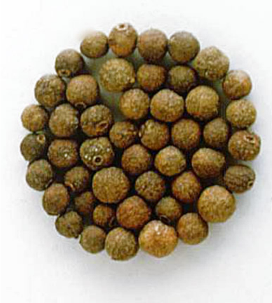

random control

carry

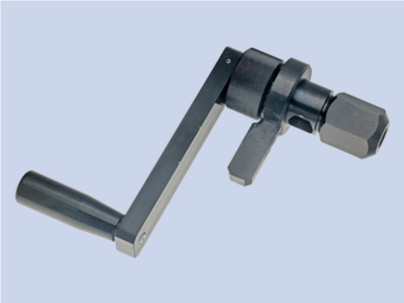

predicted

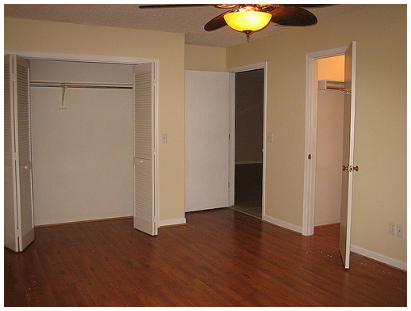

random control

lunatic

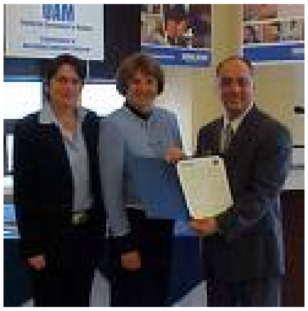

predicted

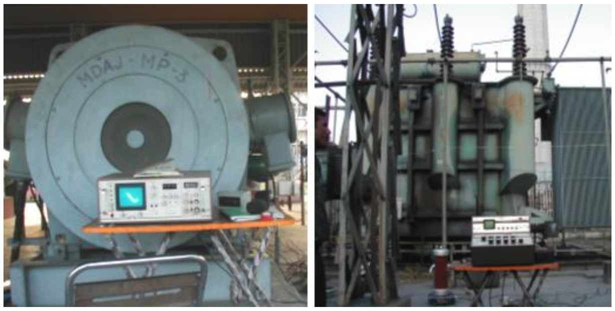

random control

gangster

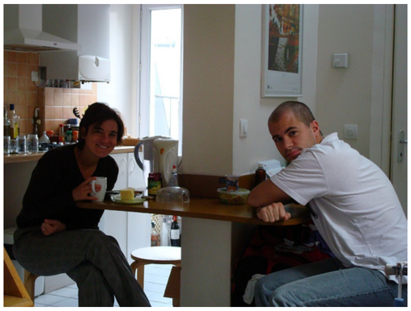

predicted

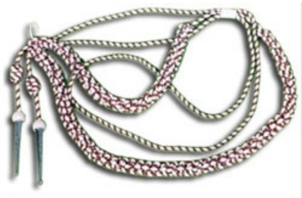

random control

evidence

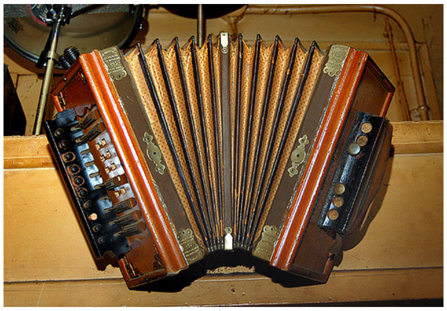

predicted

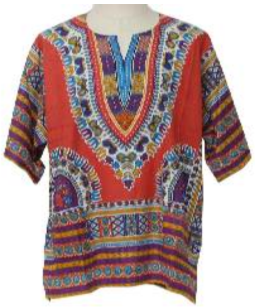

random control

plan

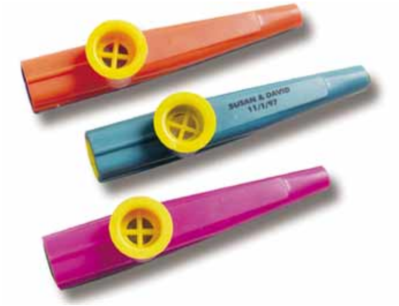

predicted

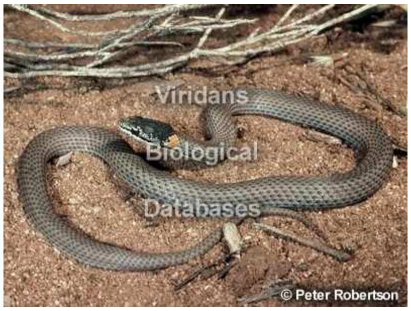

random control

economics

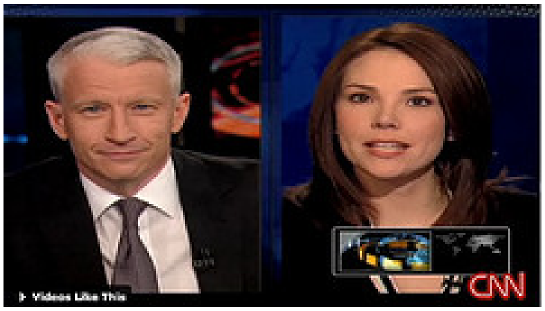

predicted

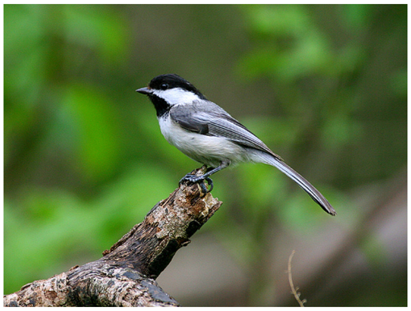

random control

coverage

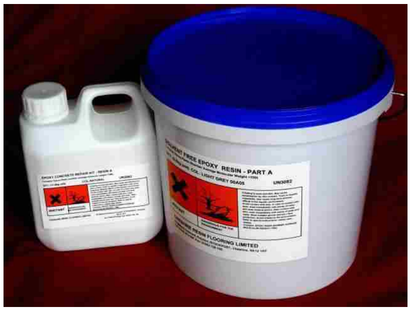

predicted

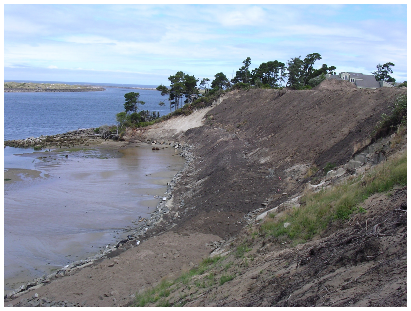

random control

parent

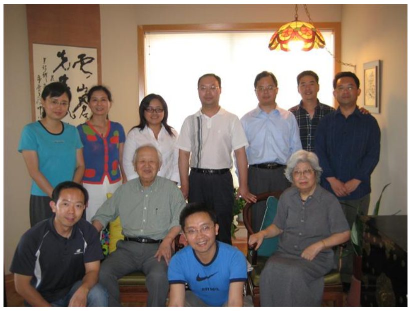

predicted

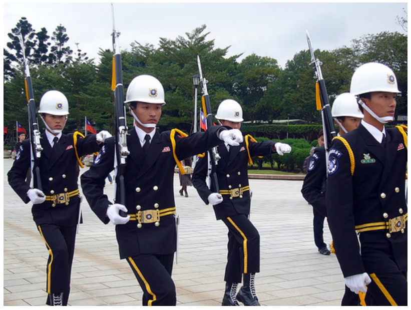

random control

pipeline

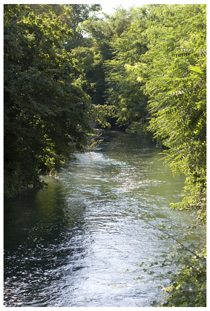

predicted

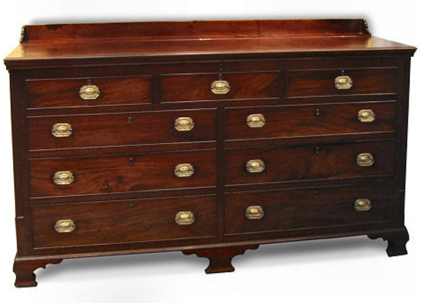

random control

fiend

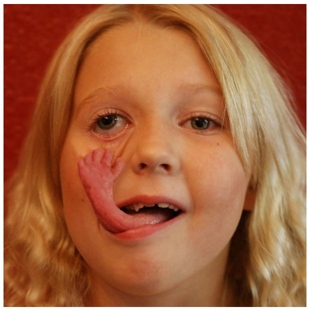

predicted

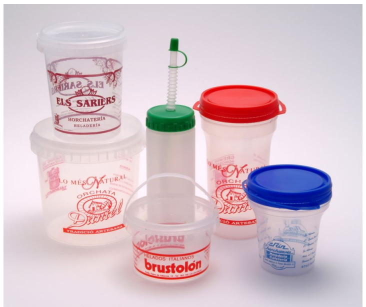

random control

debate

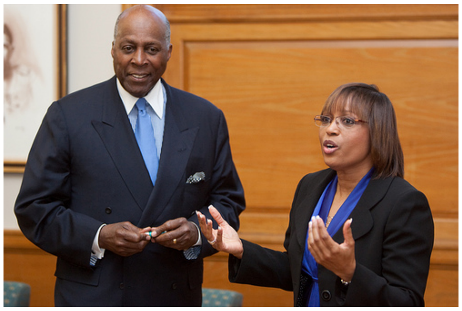

predicted

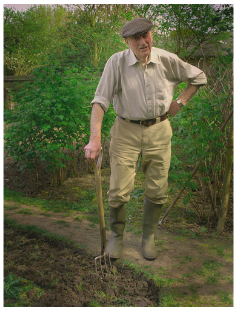

random control

sarcasm

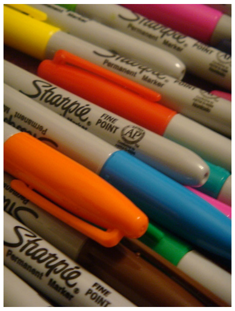

predicted

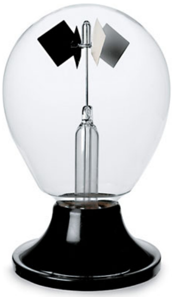

random control

flush

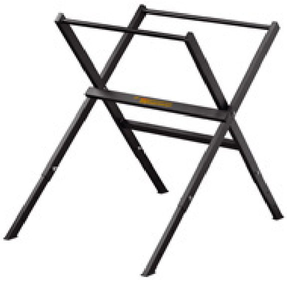

predicted

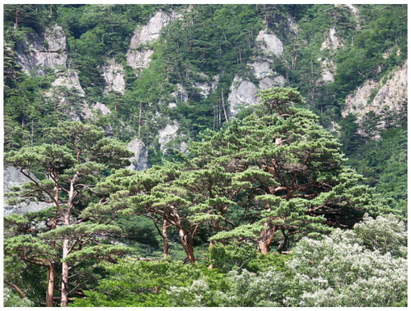

random control

importance

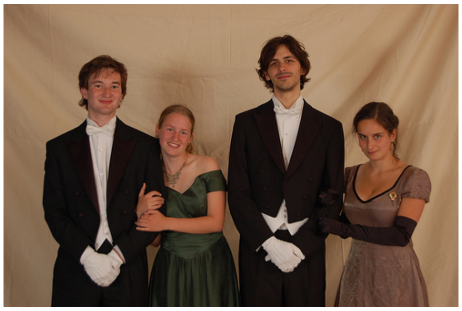

predicted

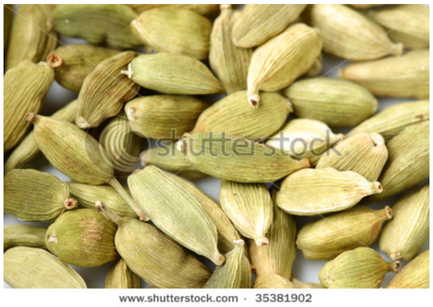

random control

share

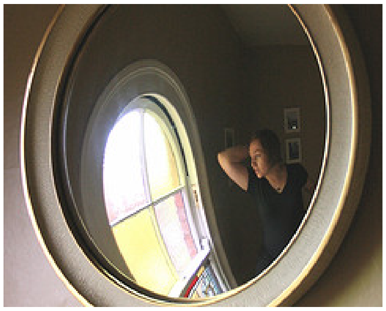

predicted

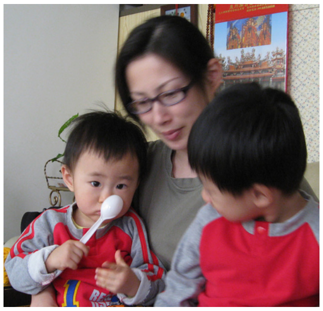

random control

supreme

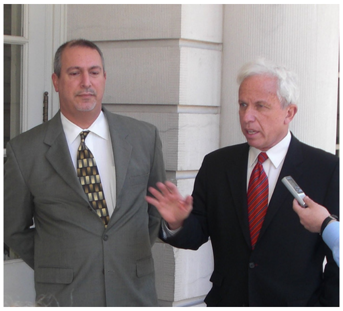

predicted

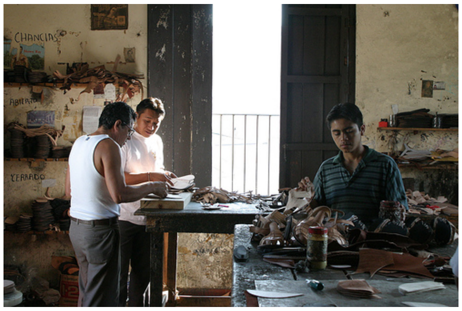

random control

avoid

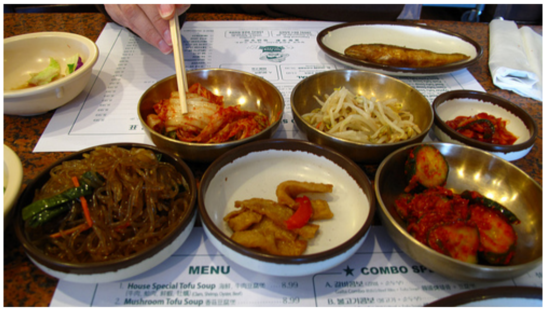

predicted

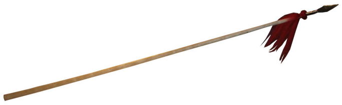

random control

capital

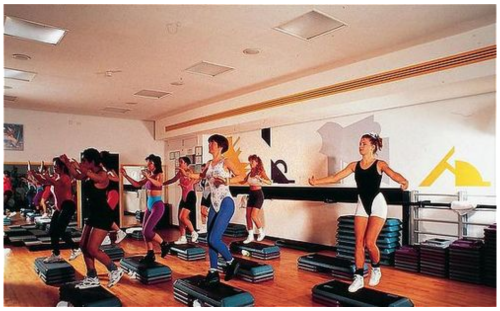

predicted

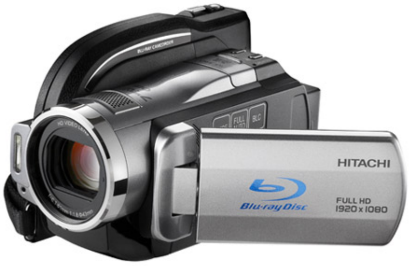

random control

rating

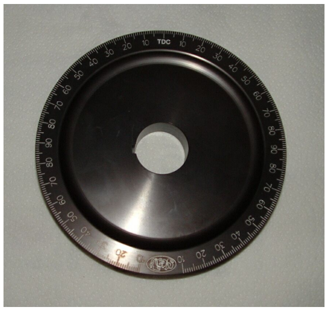

predicted

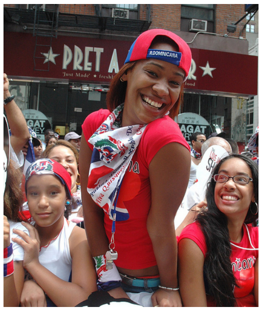

random control

manner

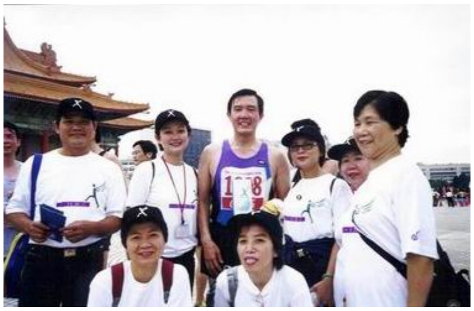

predicted

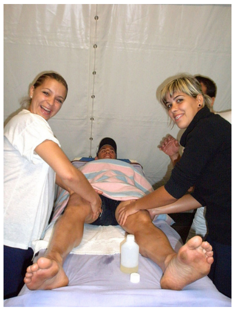

random control

fuss

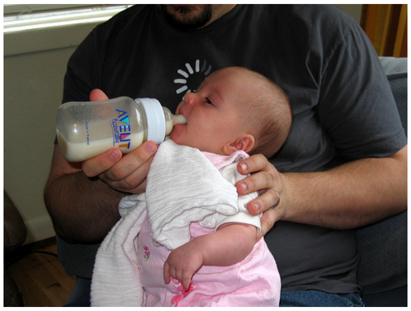

predicted

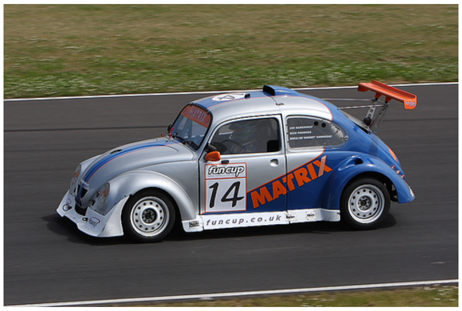

random control

prairie

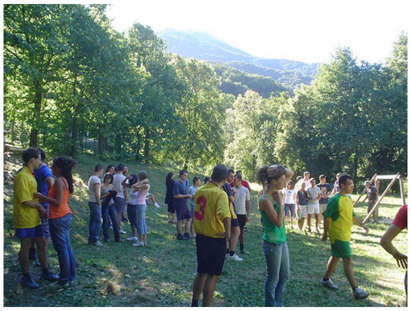

predicted

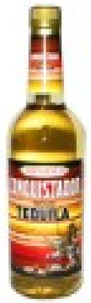

random control

affair

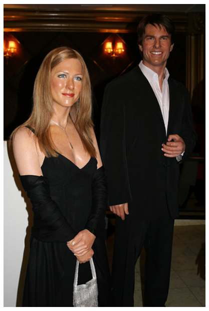

predicted

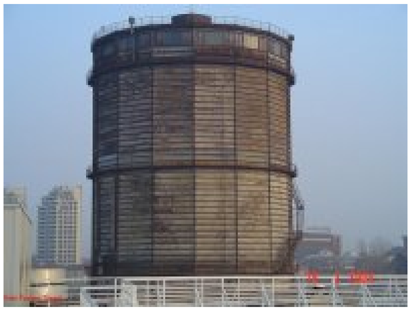

random control

forum

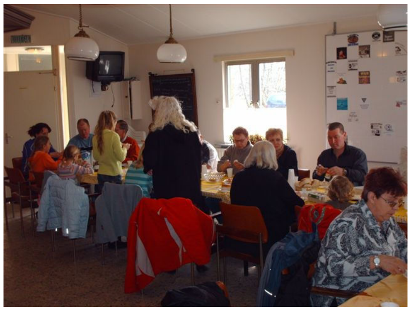

predicted

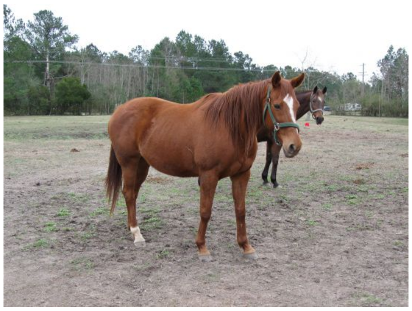

random control

sale

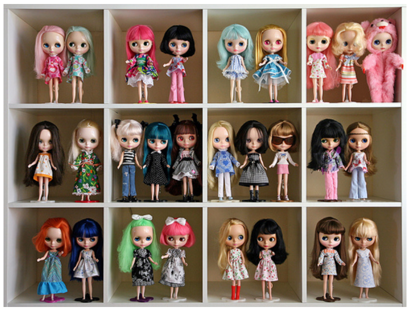

predicted

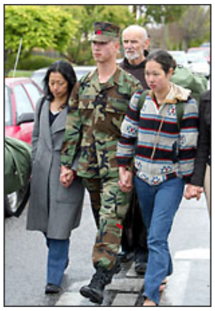

random control

hurry

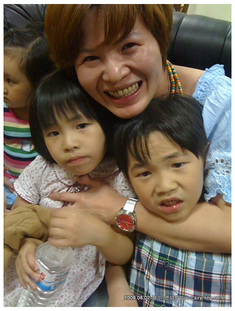

predicted

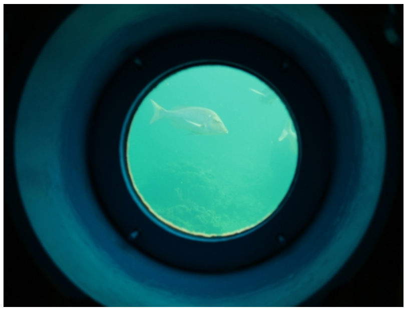

random control

faith

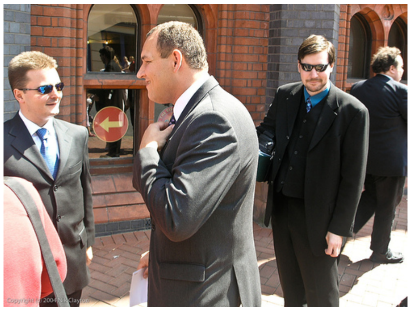

predicted

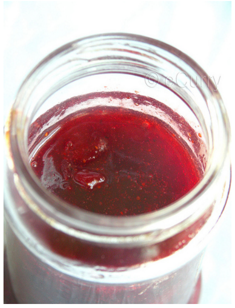

random control

elf

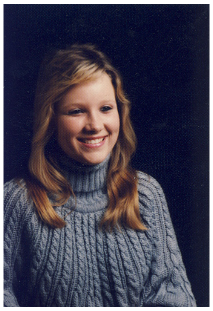

predicted

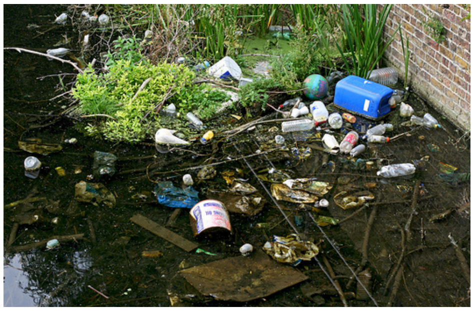

random control

rebound

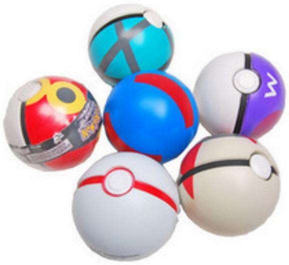

predicted

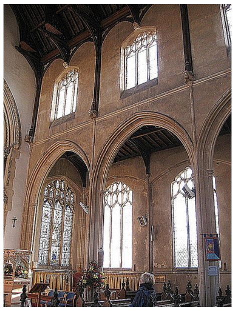

random control

beginning

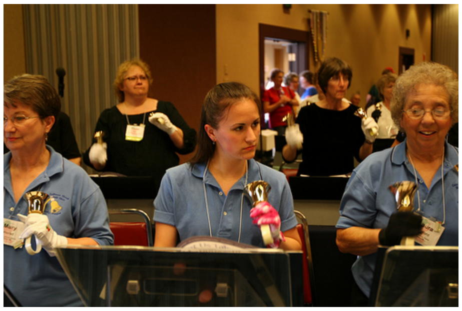

predicted

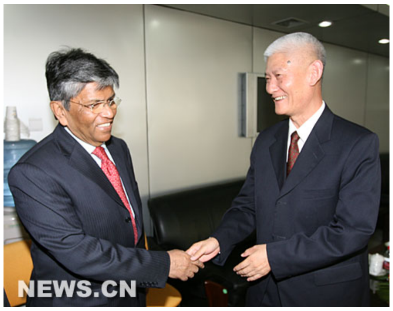

random control

pageant

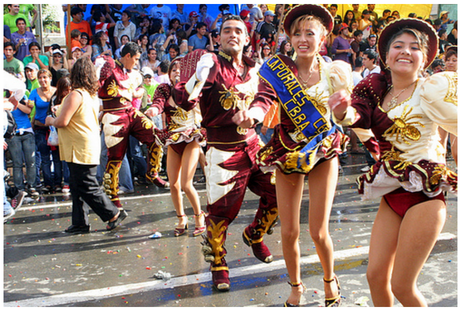

predicted

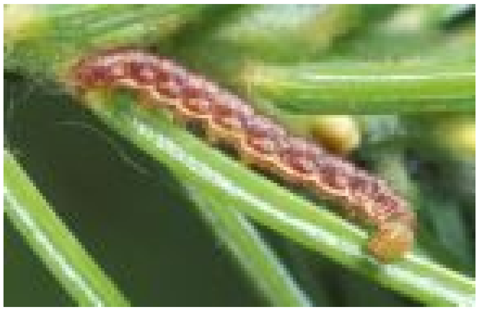

random control

subconscious

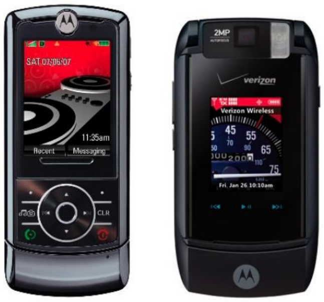

predicted

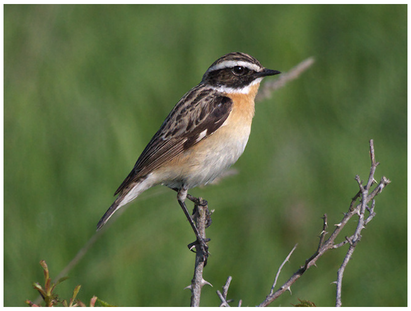

random control

demonstration

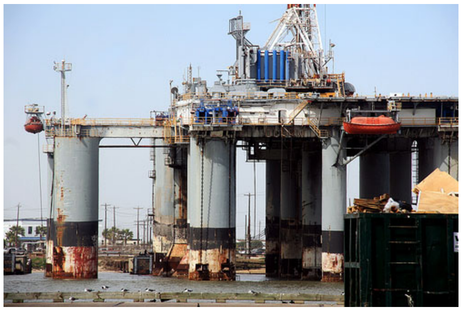

predicted

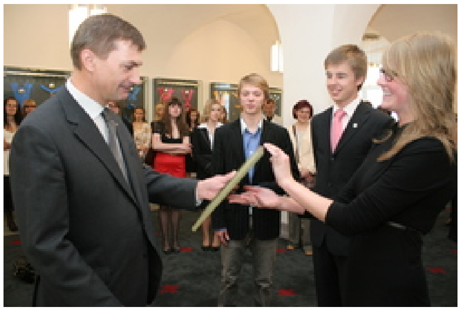

random control

relation

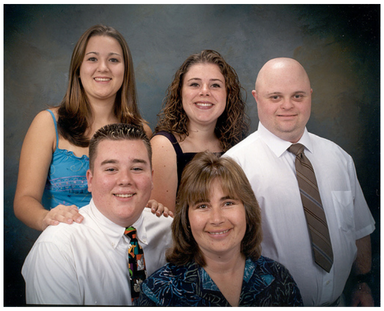

predicted

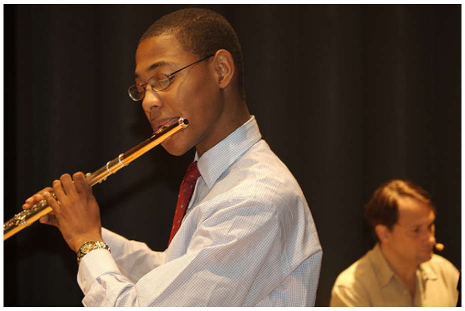

random control

psychologist

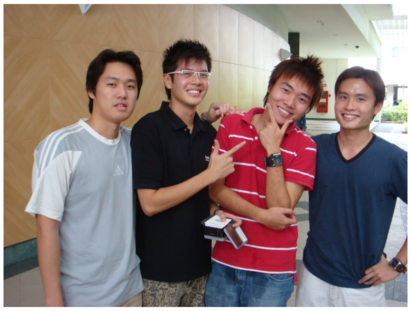

predicted

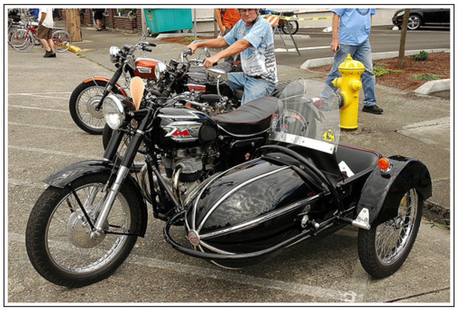

random control

generation

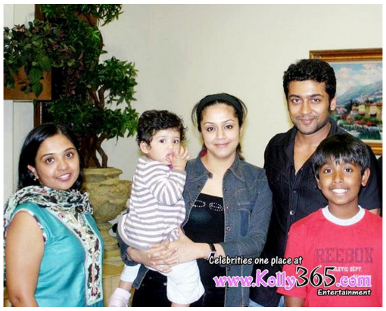

predicted

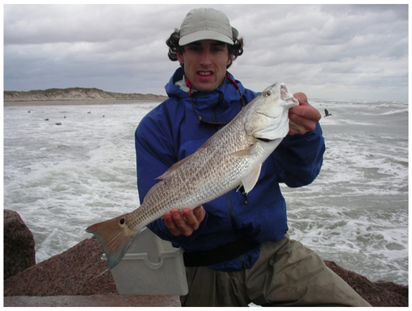

random control

term

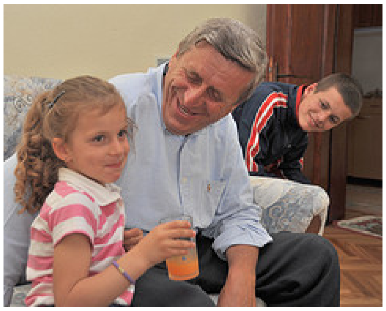

predicted

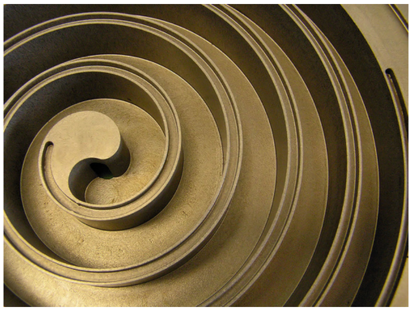

random control

automobile

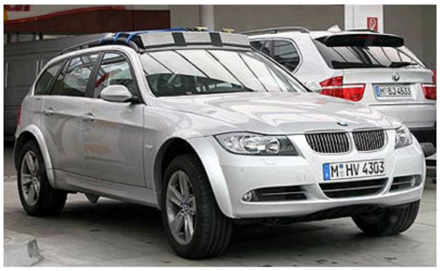

predicted

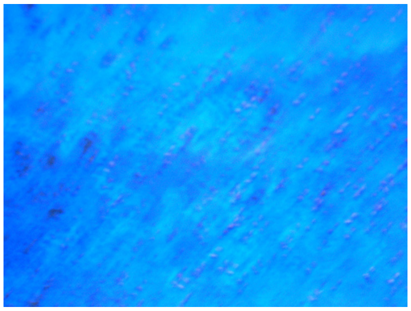

random control

millennium

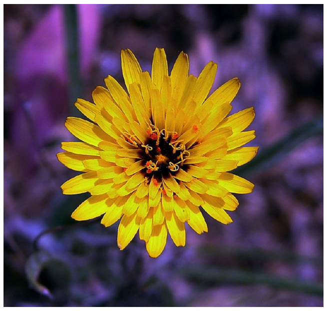

predicted

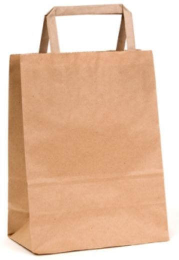

random control

low

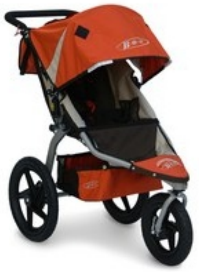

predicted

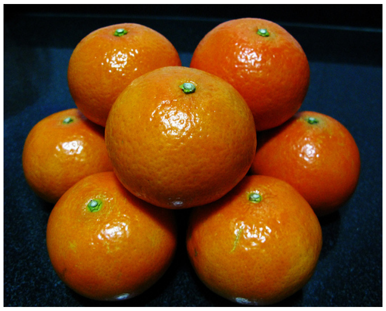

random control

virus

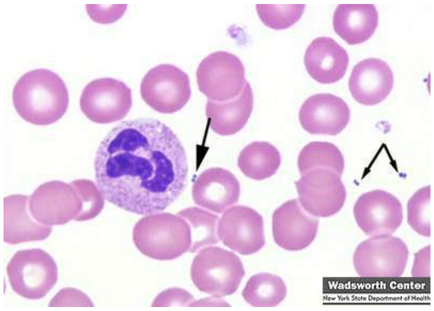

predicted

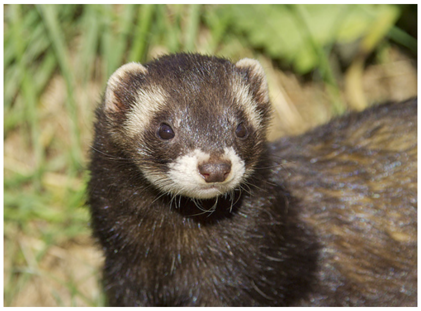

random control

ache

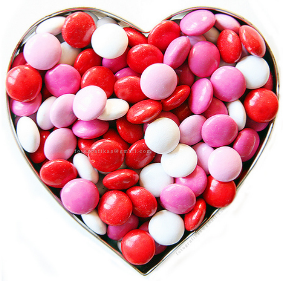

predicted

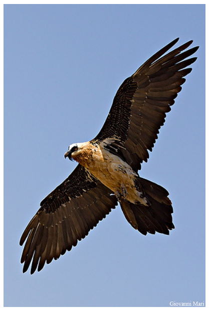

random control

fed

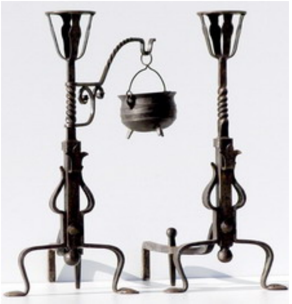

predicted

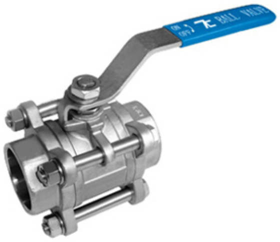

random control

list

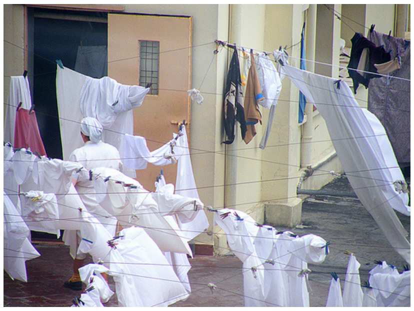

predicted

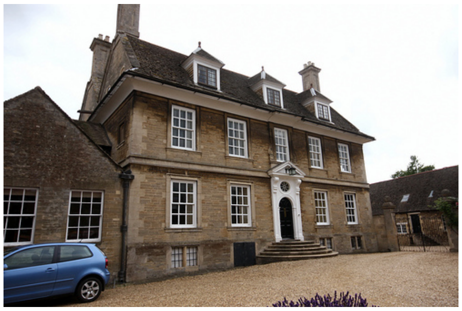

random control

cripple

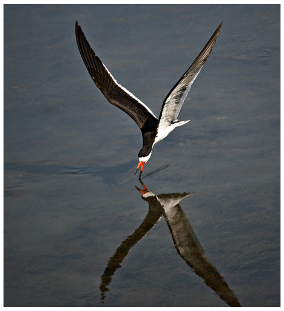

predicted

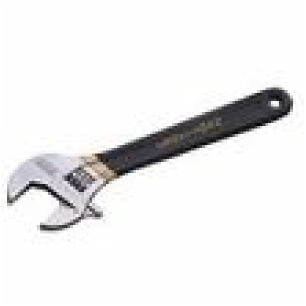

random control

sore

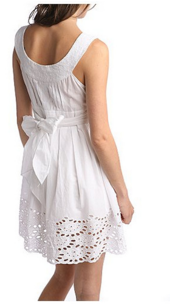

predicted

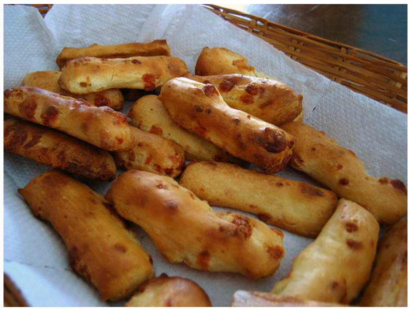

random control

entertainment

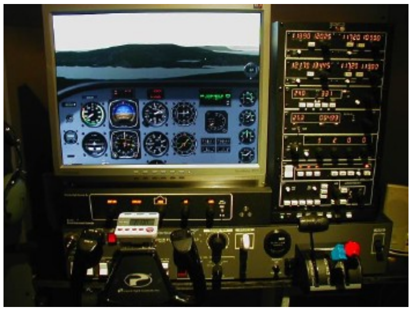

predicted

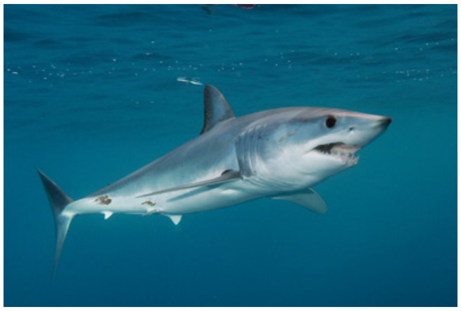

random control

fusion

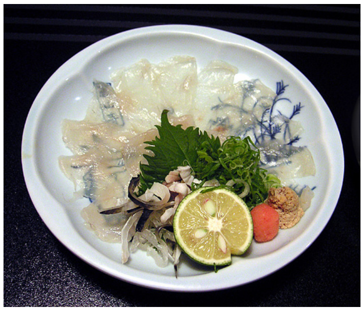

predicted

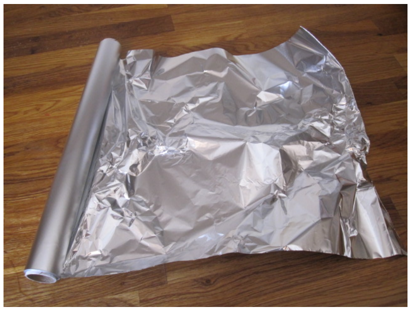

random control

proposal

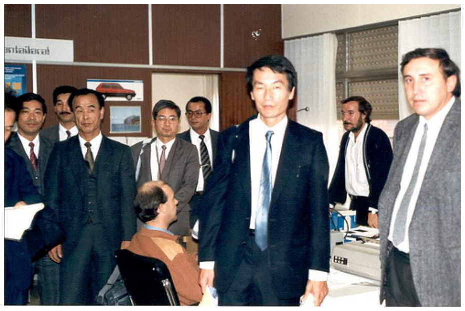

predicted

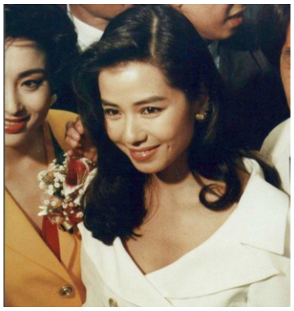

random control

driving

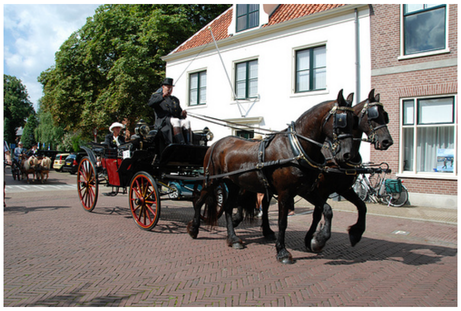

predicted

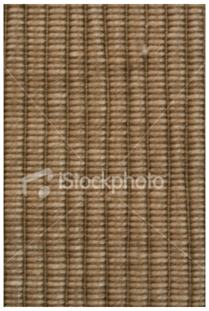

random control

piss

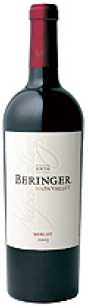

predicted

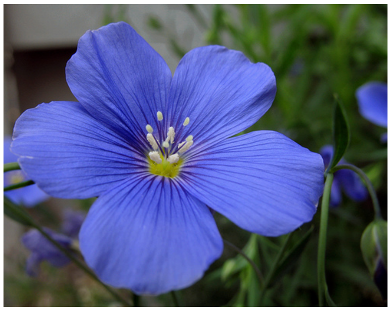

random control

message

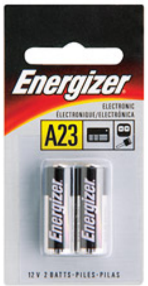

predicted

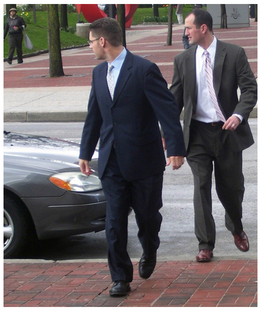

random control

address

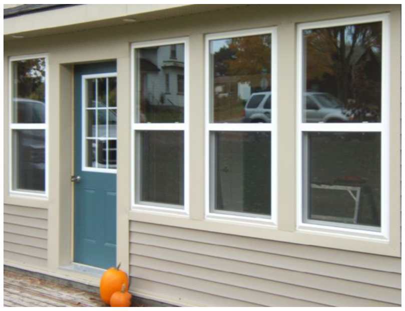

predicted

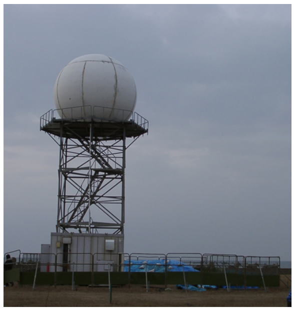

random control

oil

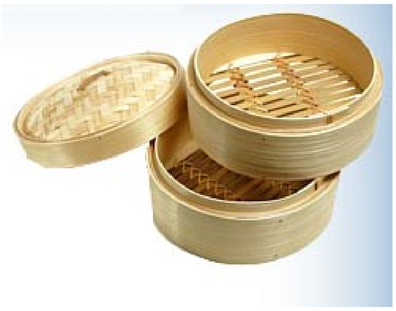

predicted

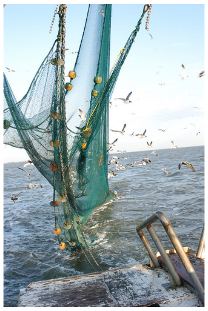

random control

lifetime

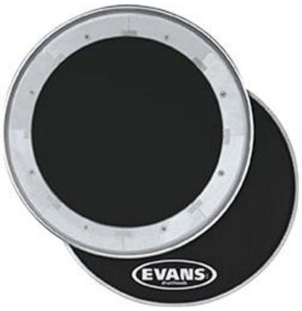

predicted

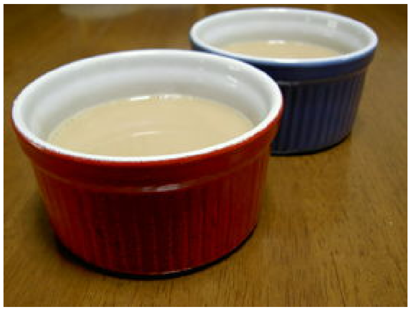

random control

slayer

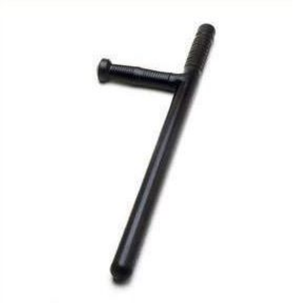

predicted

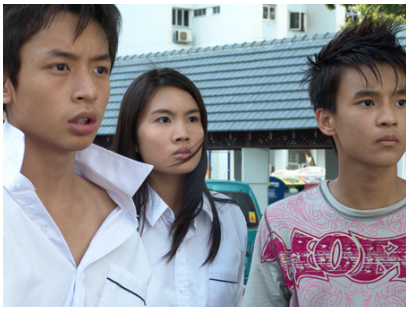

random control

explanation

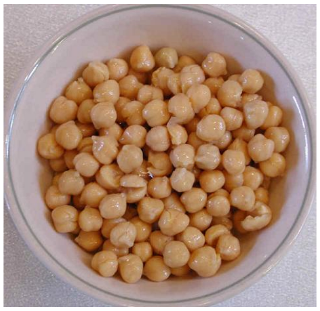

predicted

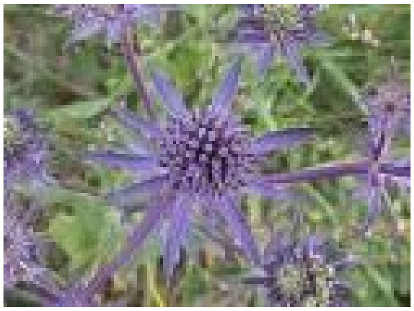

random control

spotlight

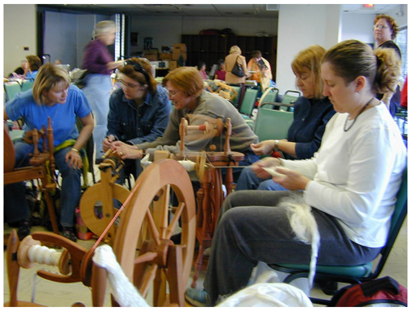

predicted

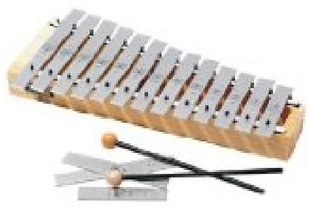

random control

article

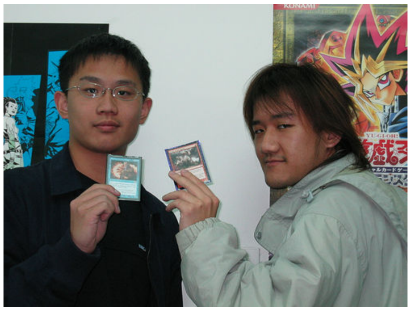

predicted

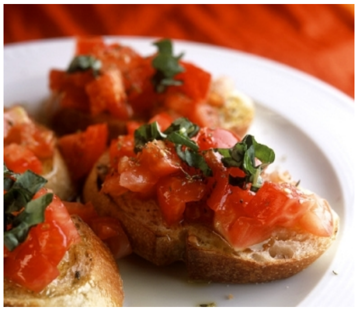

random control

series

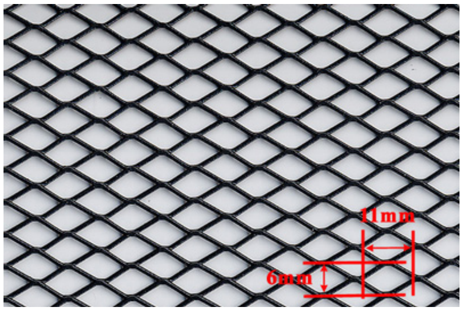

predicted

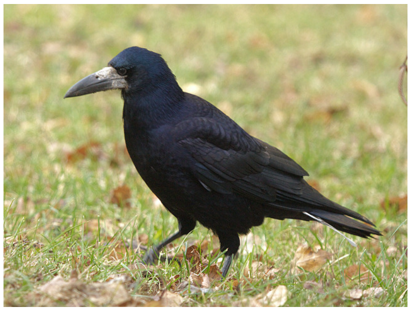

random control

exam

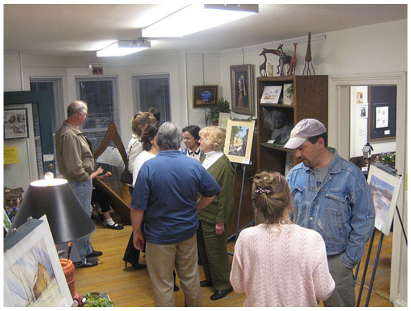

predicted

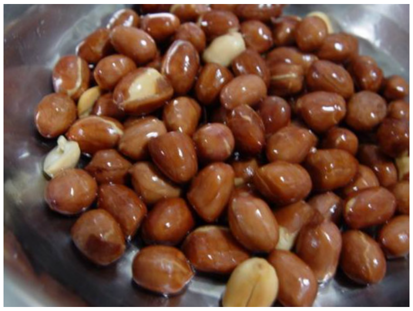

random control

disaster

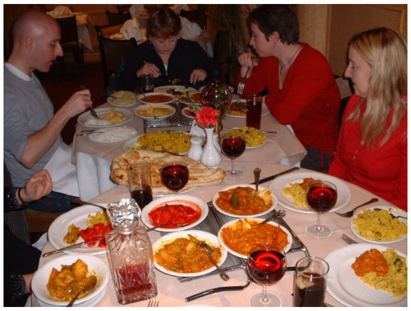

predicted

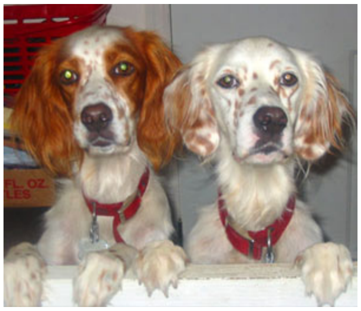

random control

river

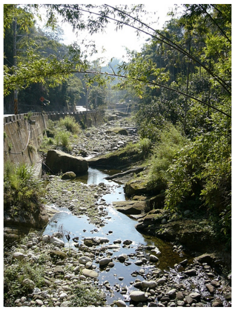

predicted

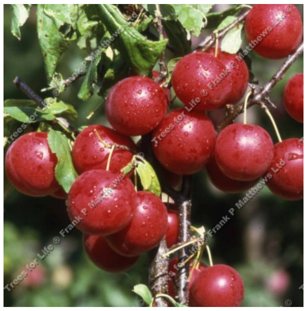

random control

ecstasy

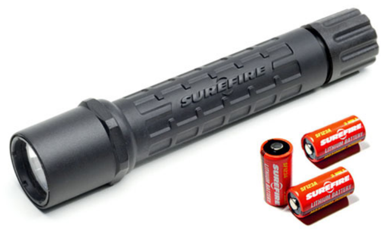

predicted

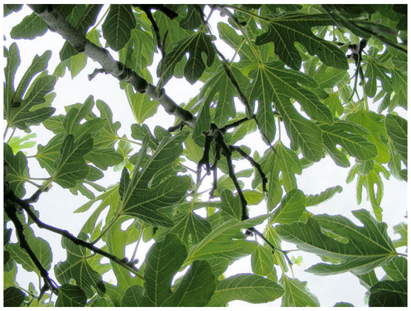

random control

shape

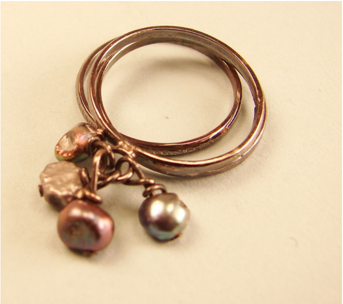

predicted

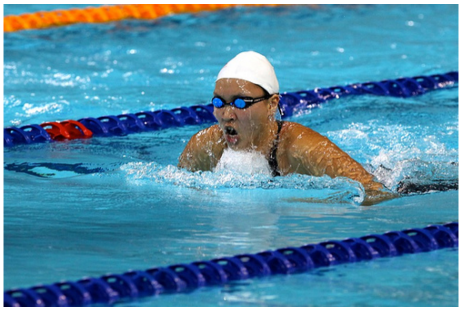

random control

ulcer

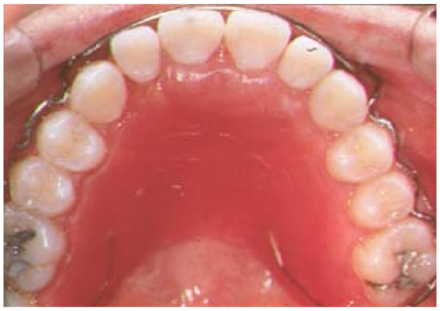

predicted

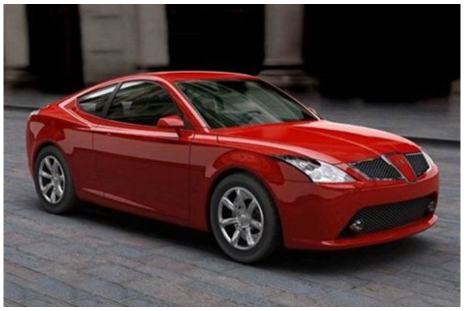

random control

test

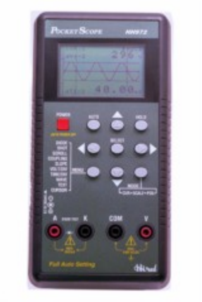

predicted

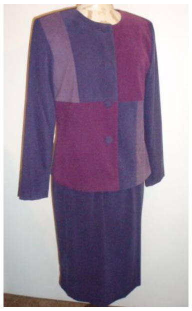

random control

population

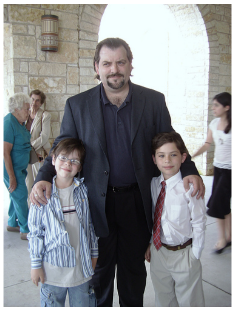

predicted

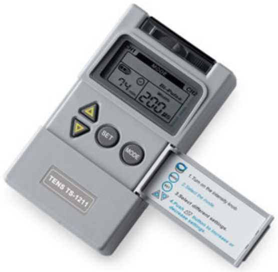

random control

getaway

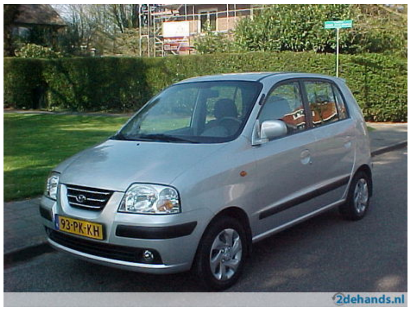

predicted

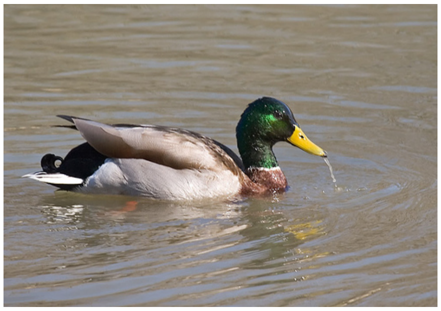

random control

goddess

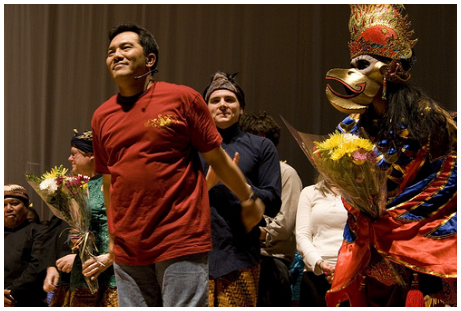

predicted

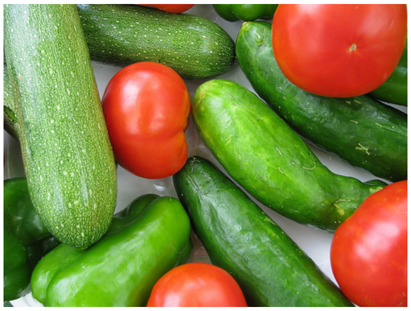

random control

consultant

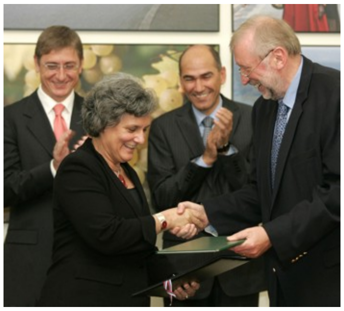

predicted

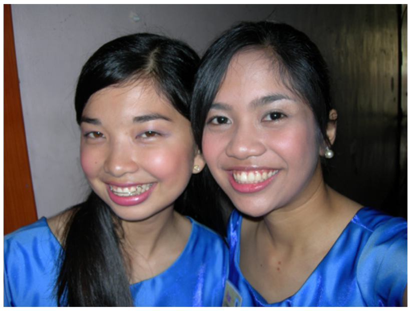

random control

psychiatry

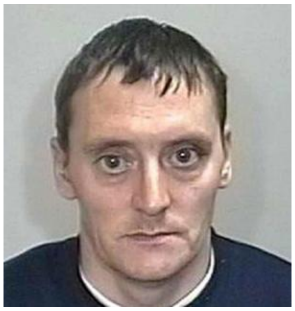

predicted

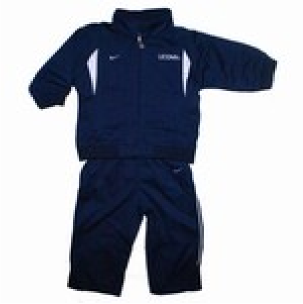

random control

feed

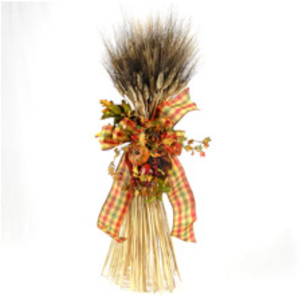

predicted

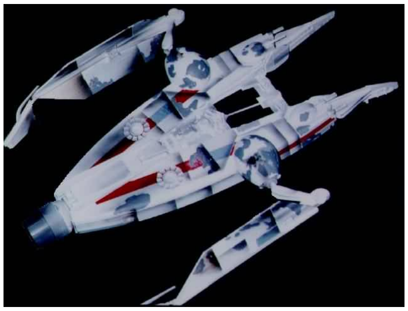

random control

administration

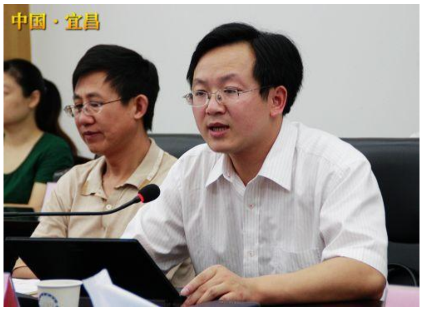

predicted

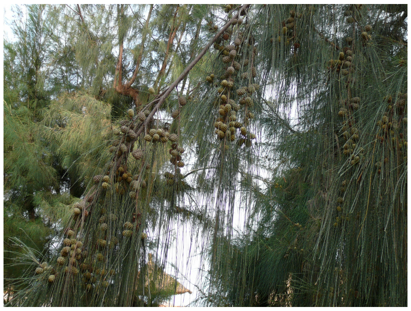

random control

manufacture

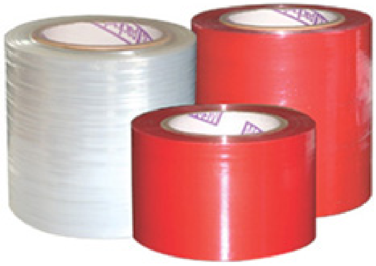

predicted

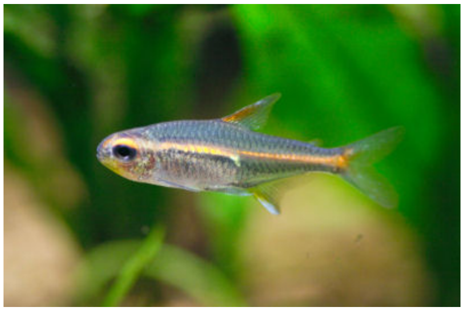

random control

thesis

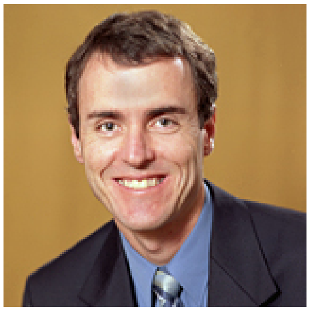

predicted

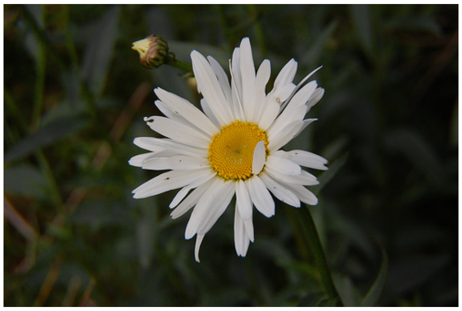

random control

bulletin

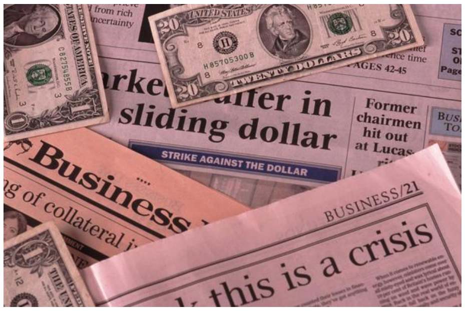

predicted

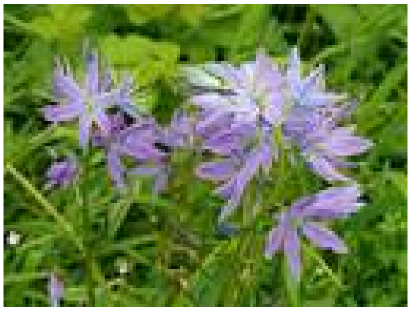

random control

tenth

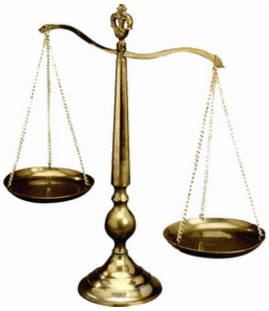

predicted

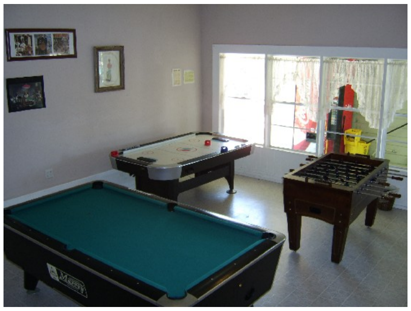

random control

final

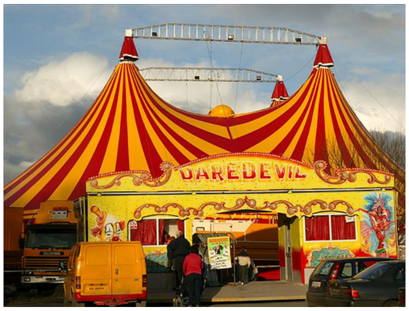

predicted

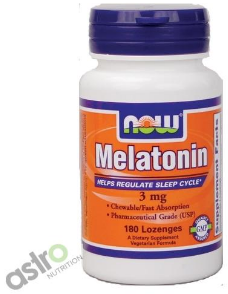

random control

postman

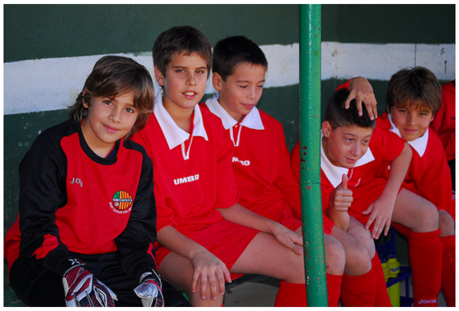

predicted

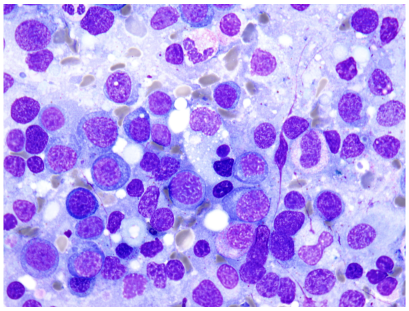

random control

payment

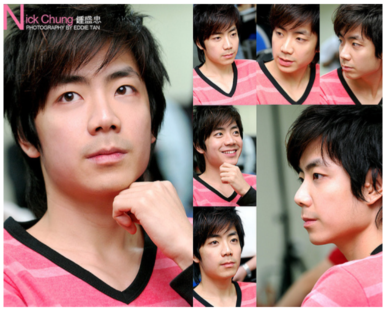

predicted

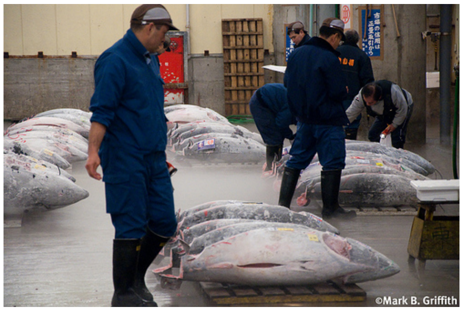

random control

posse

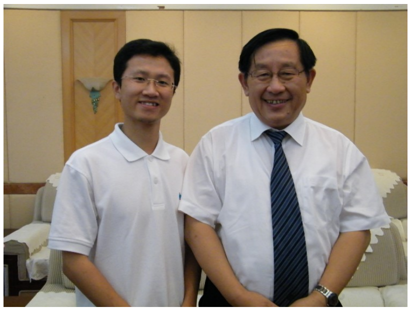

predicted

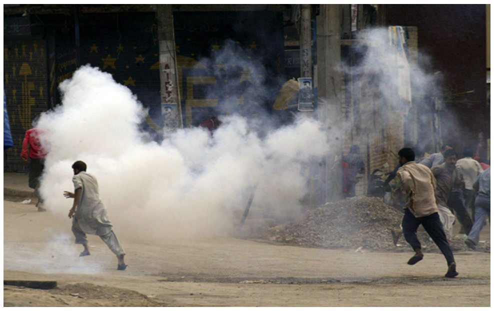

random control

situation

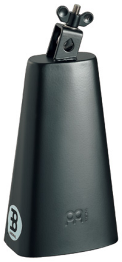

predicted

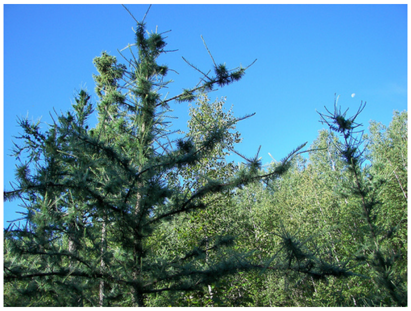

random control

accusation

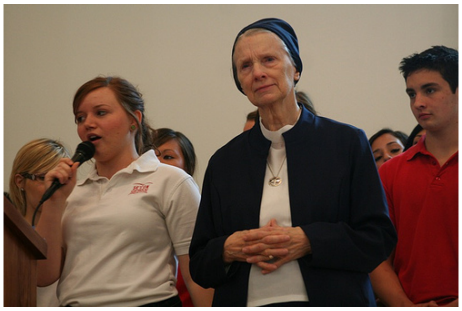

predicted

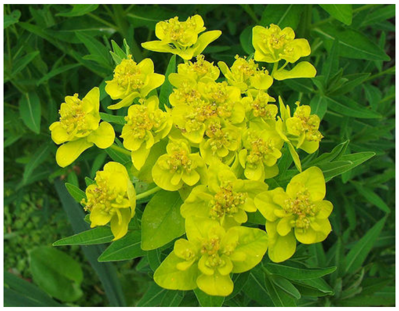

random control

discussion

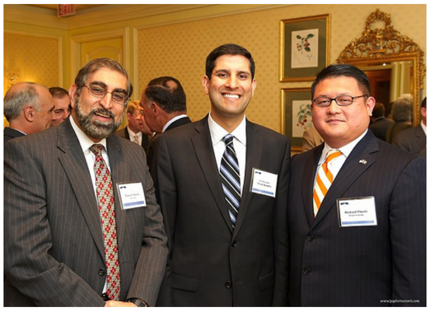

predicted

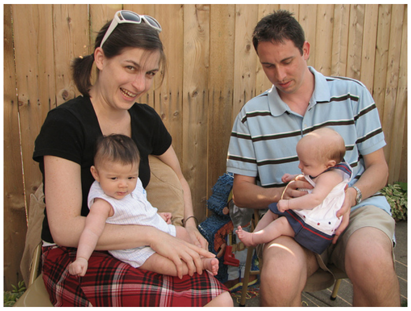

random control

antenna

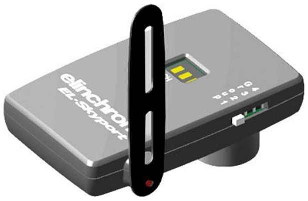

predicted

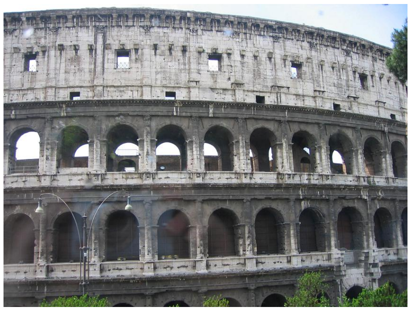

random control

messenger

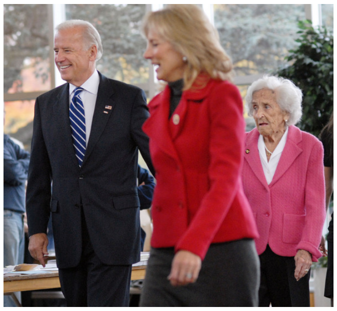

predicted

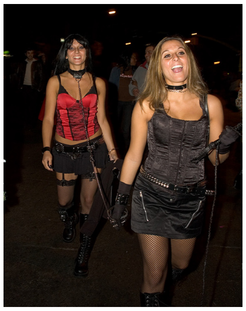

random control

humiliation

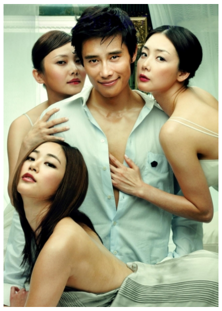

predicted

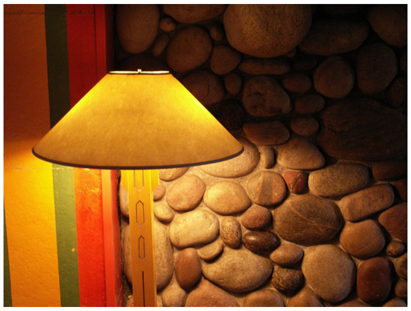

random control

appreciation

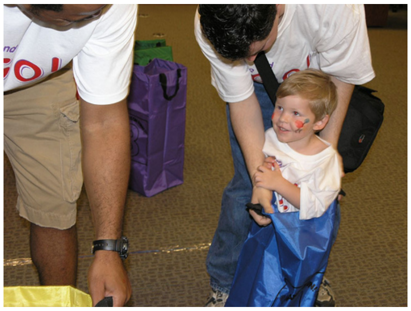

predicted

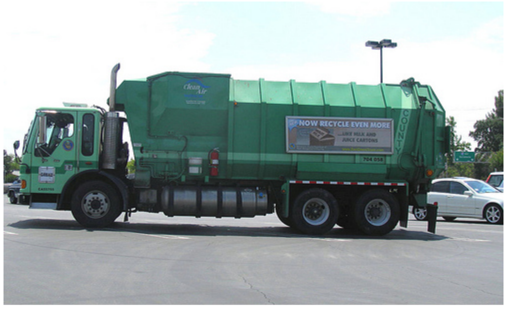

random control

note

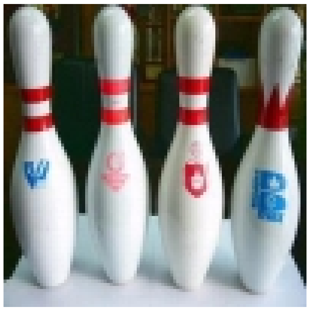

predicted

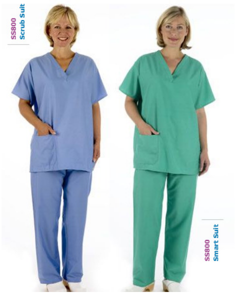

random control

nomination

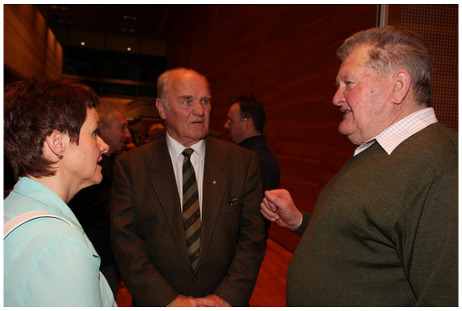

predicted

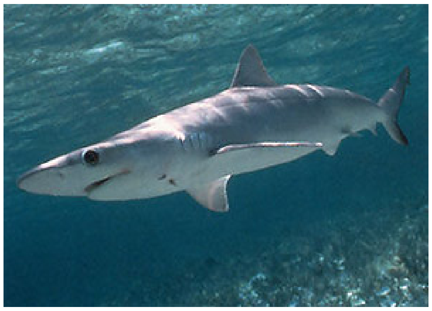

random control

grab

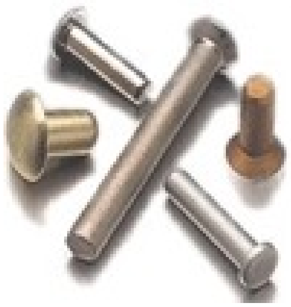

predicted

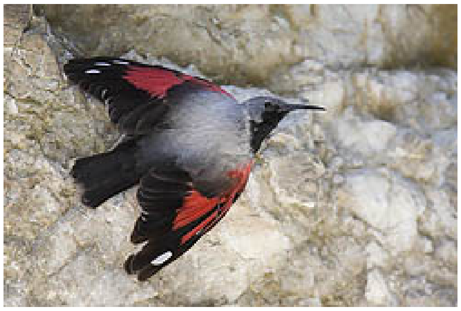

random control

chaplain

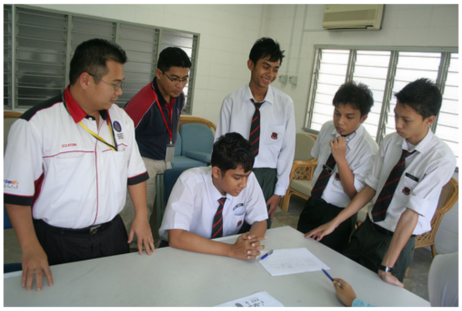

predicted

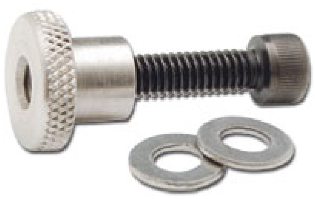

random control

cruise

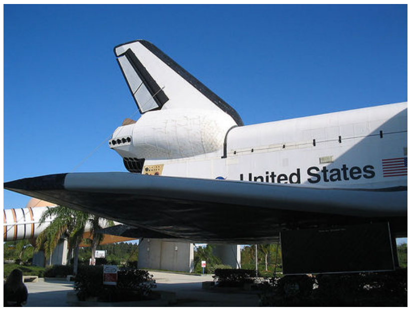

predicted

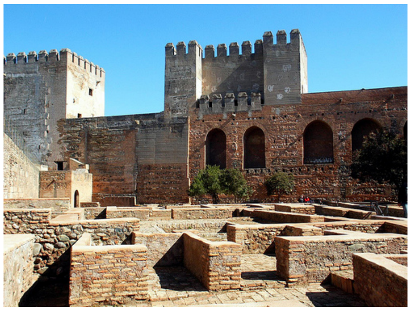

random control

burning

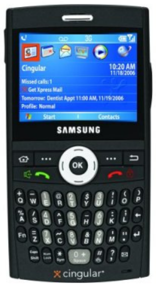

predicted

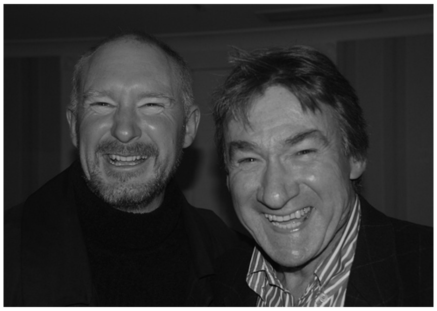

random control

brain

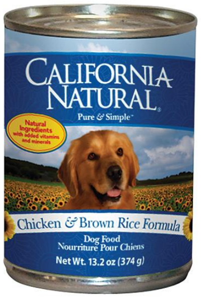

predicted

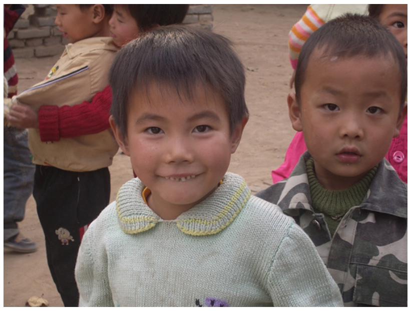

random control

sinner

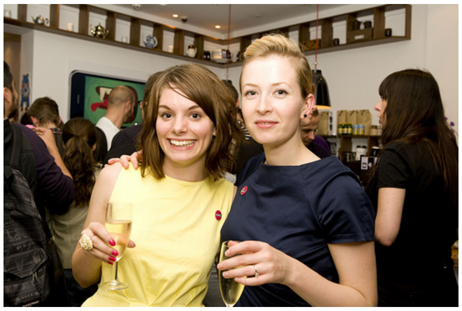

predicted

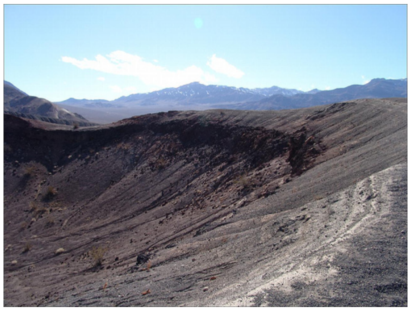

random control

goody

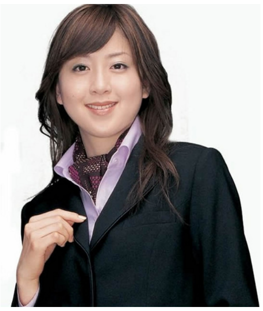

predicted

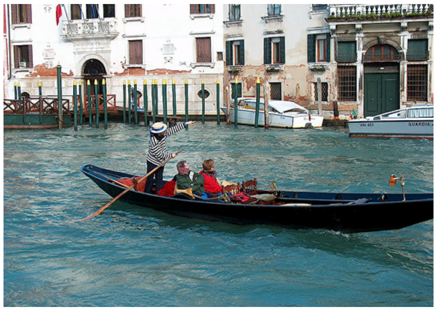

random control

barber

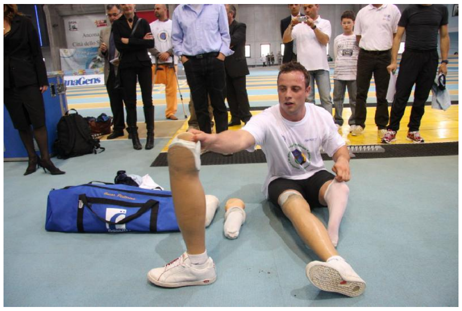

predicted

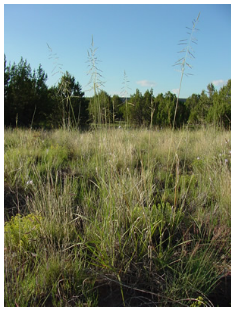

random control

republic

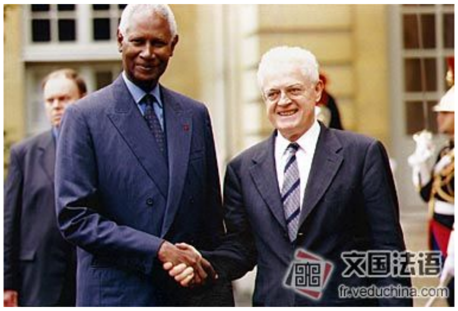

predicted

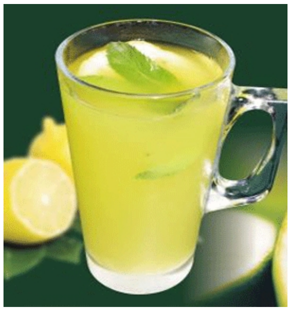

random control

tenderness

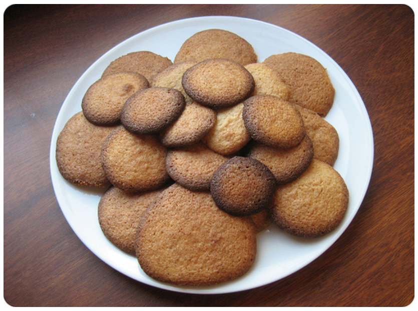

predicted

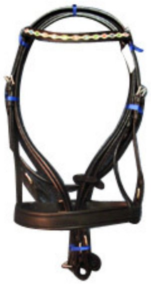

random control

bake

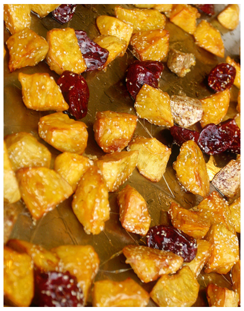

predicted

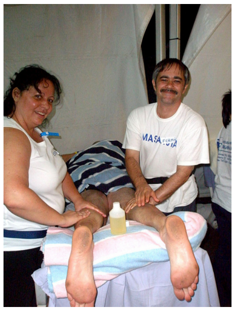

random control

dynamite

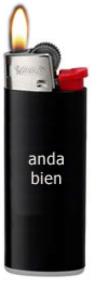

predicted

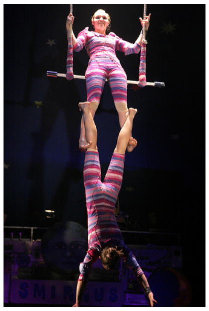

random control

impostor

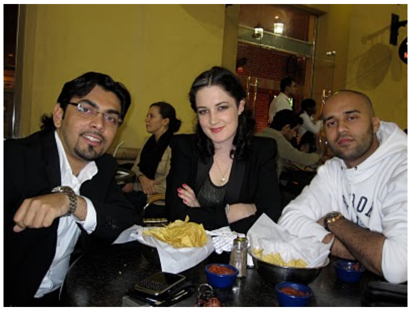

predicted

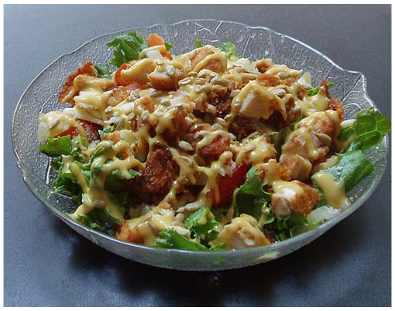

random control

former

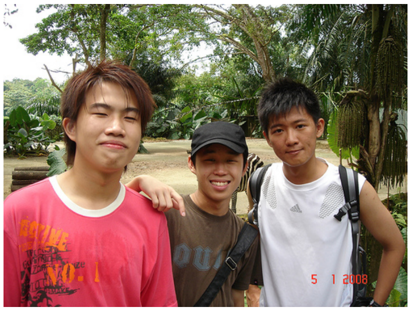

predicted

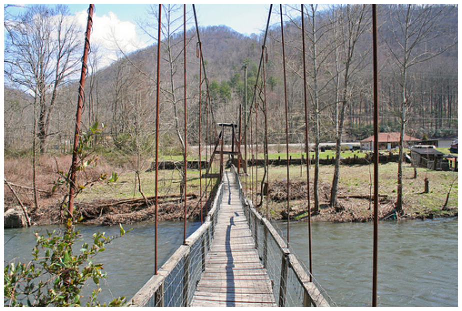

random control

conference

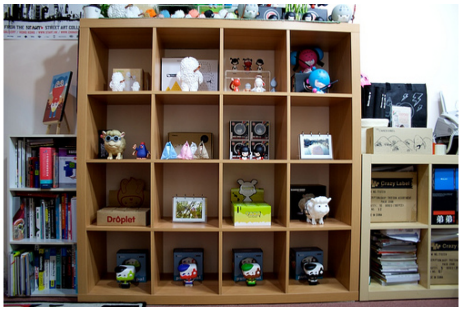

predicted

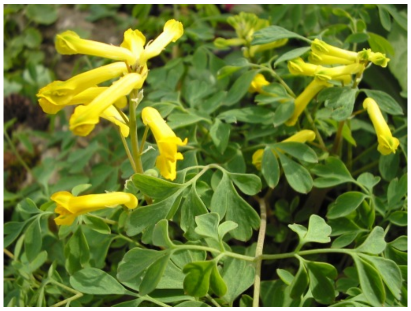

random control

textbook

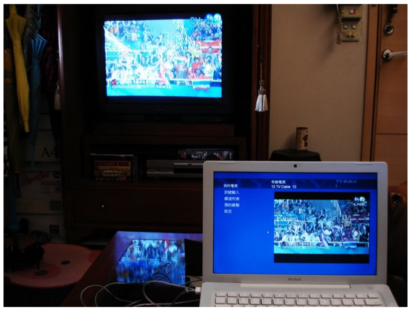

predicted

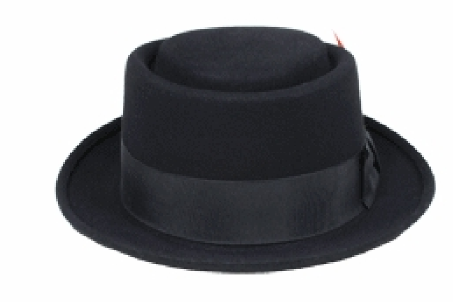

random control

curse

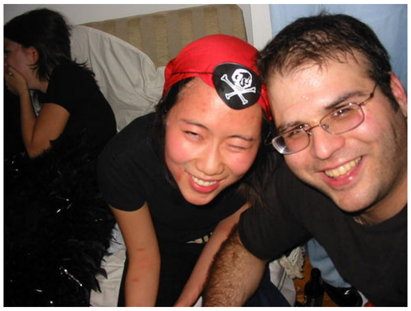

predicted

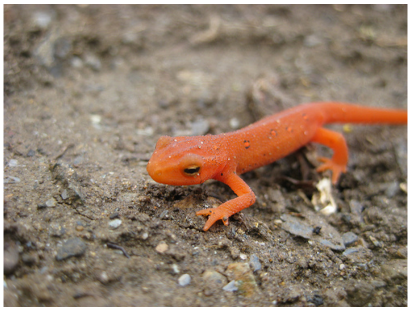

random control

navy

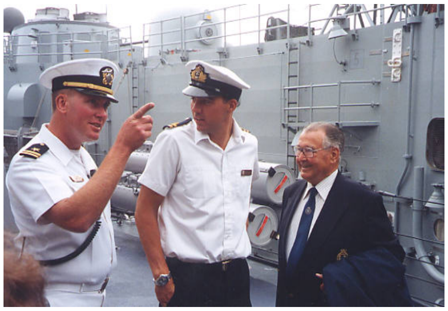

predicted

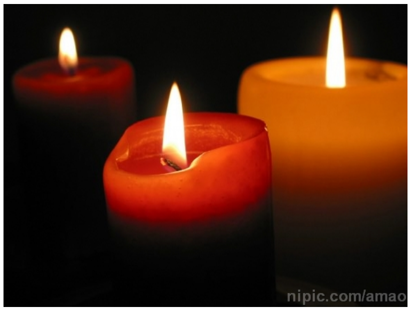

random control

loner

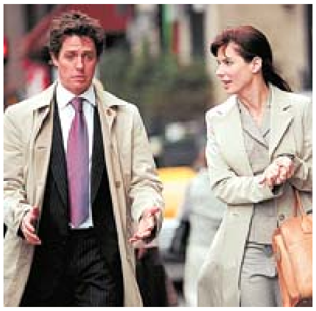

predicted

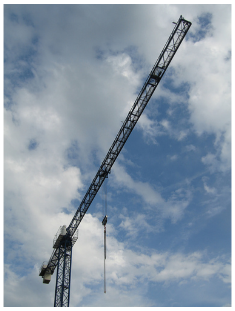

random control

sincerity

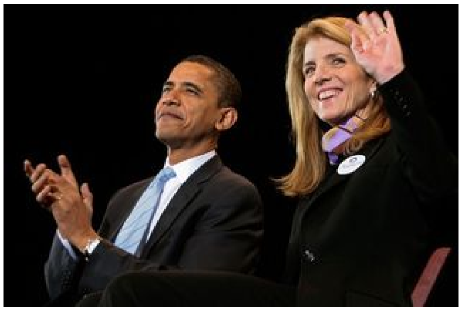

predicted

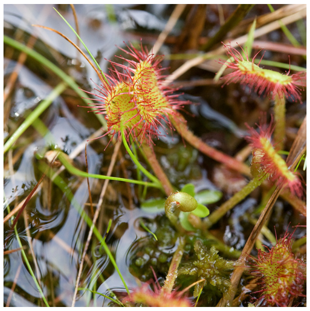

random control

poker

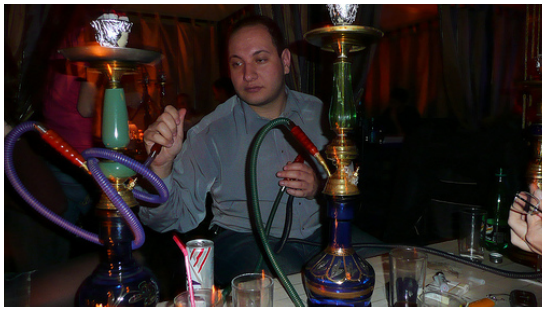

predicted

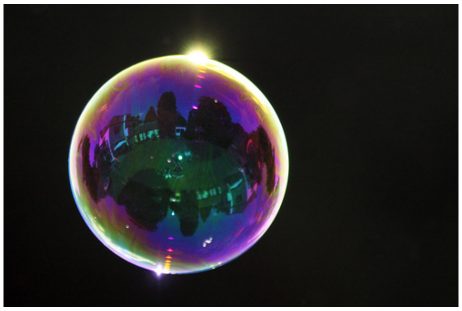

random control

clearing

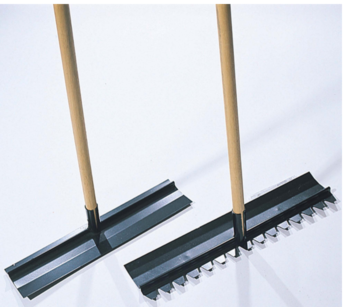

predicted

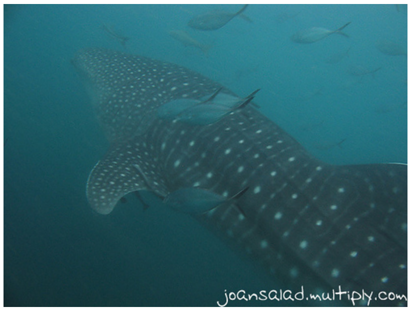

random control

revenge

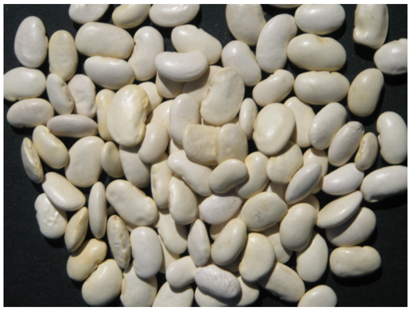

predicted

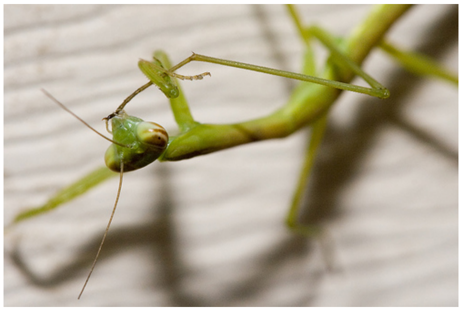

random control

ego

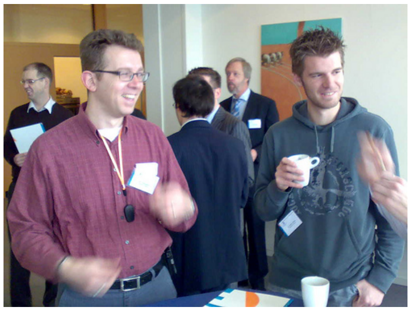

predicted

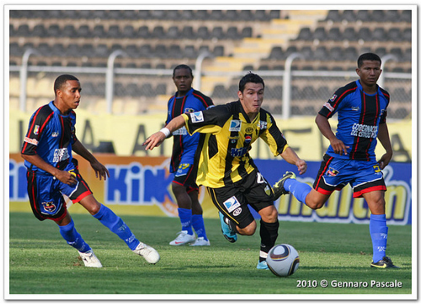

random control

triumph

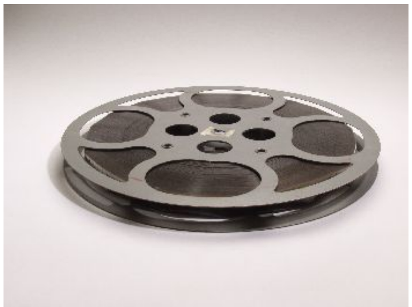

predicted

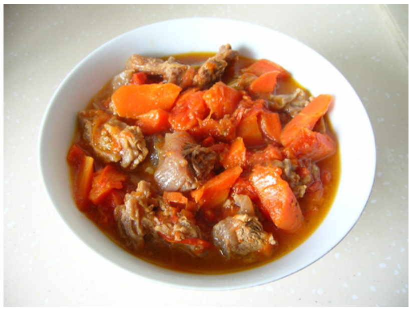

random control

hippie

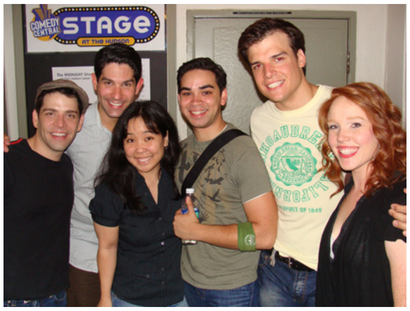

predicted

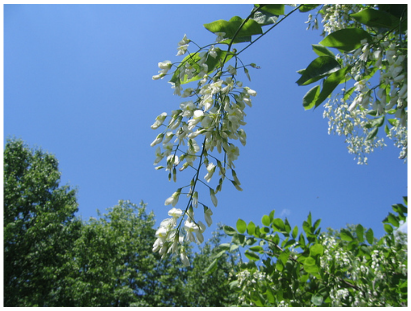

random control

charm

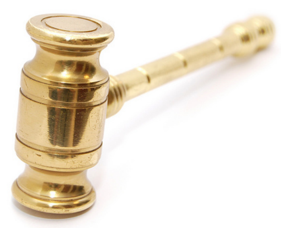

predicted

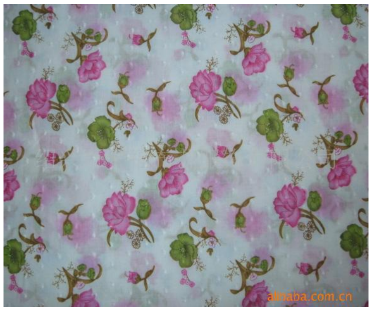

random control

bend

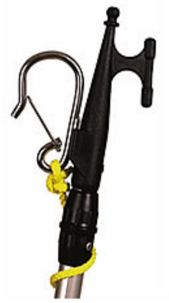

predicted

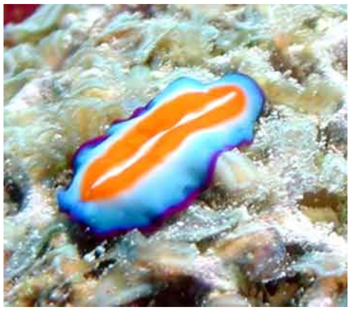

random control

negotiation

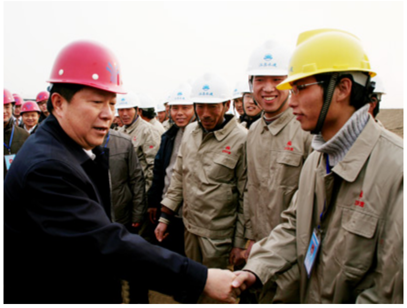

predicted

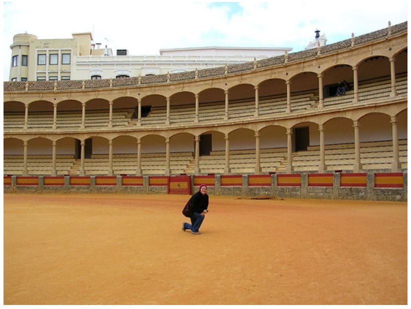

random control

liver

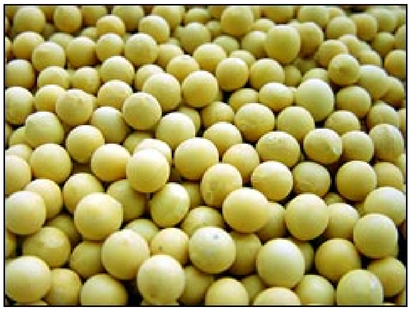

predicted

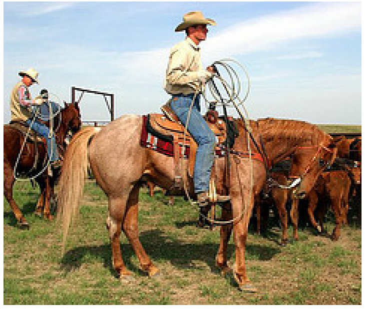

random control

angle

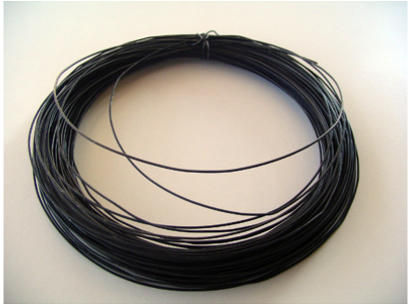

predicted

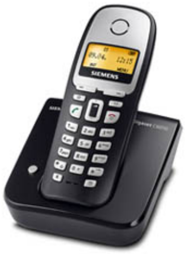

random control

cartoon

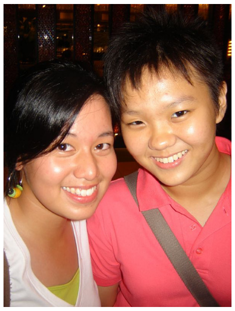

predicted

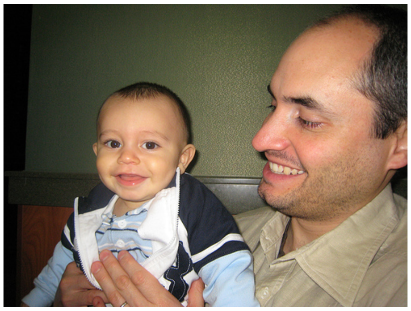

random control

shuffle

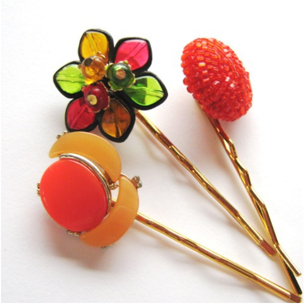

predicted

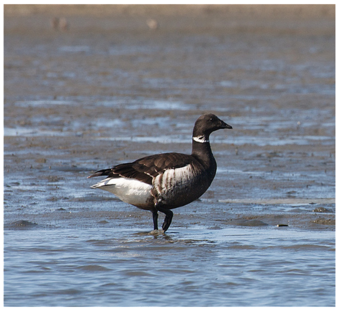

random control

goodnight

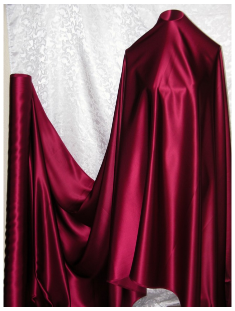

predicted

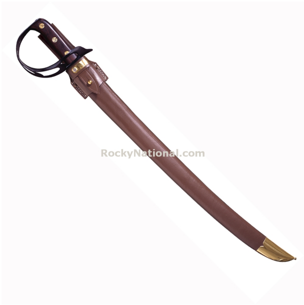

random control

hypocrite

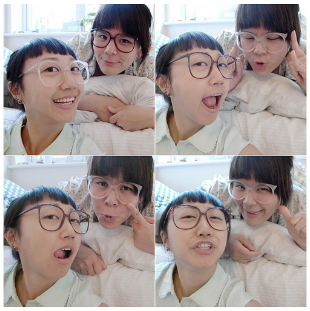

predicted

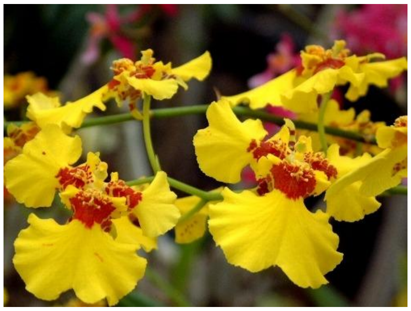

random control

fraud

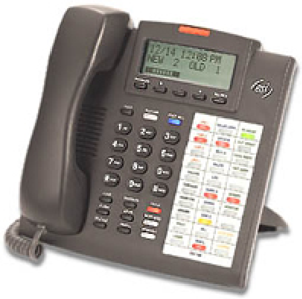

predicted

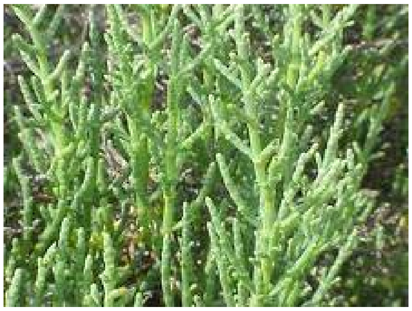

random control

temptation

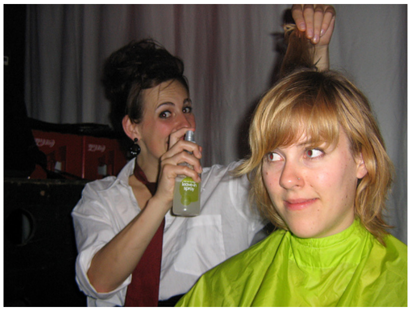

predicted

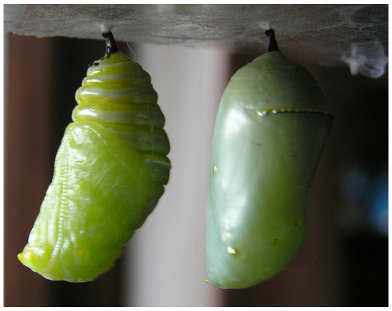

random control

haircut

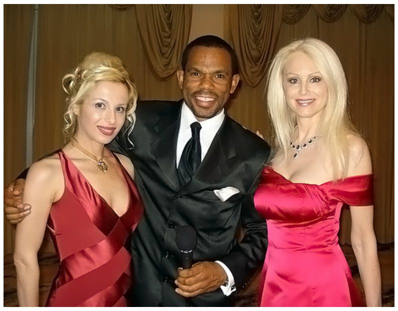

predicted

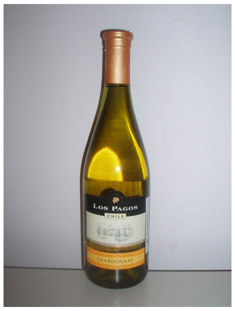

random control

grid

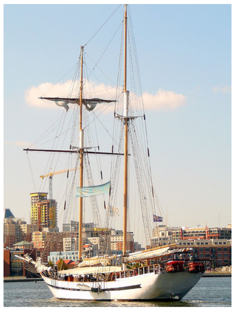

predicted

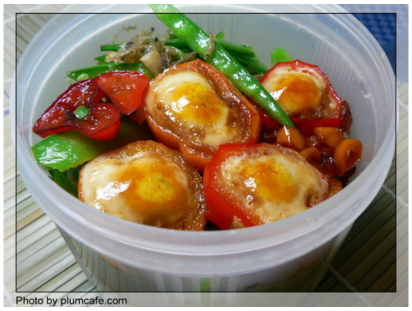

random control

cool

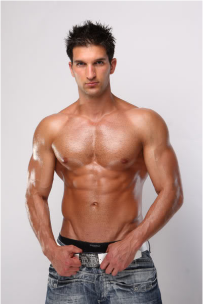

predicted

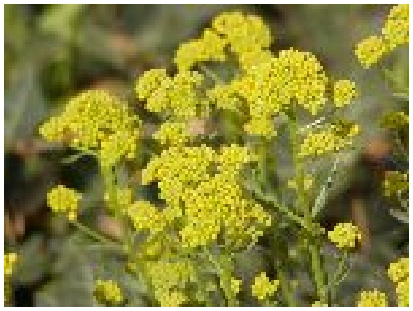

random control

autograph

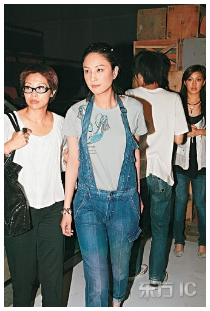

predicted

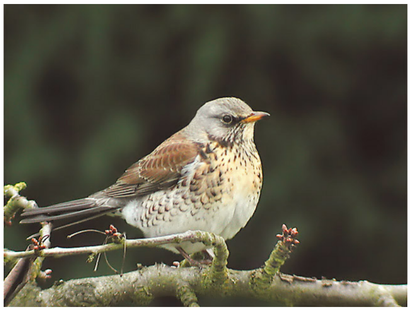

random control

affect

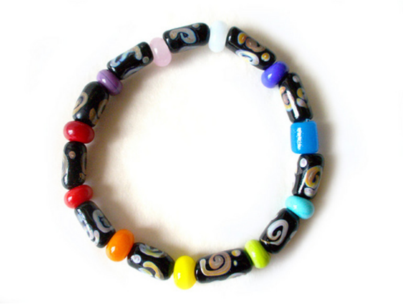

predicted

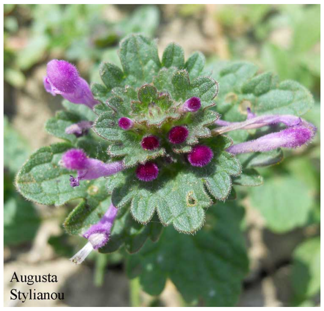

random control

quarter

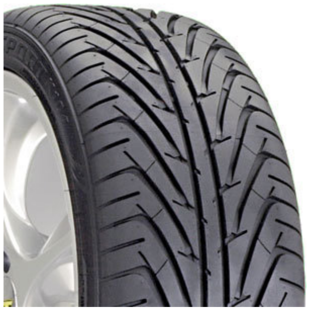

predicted

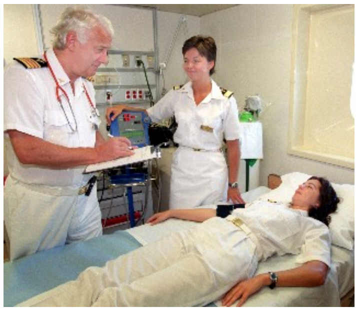

random control

patent

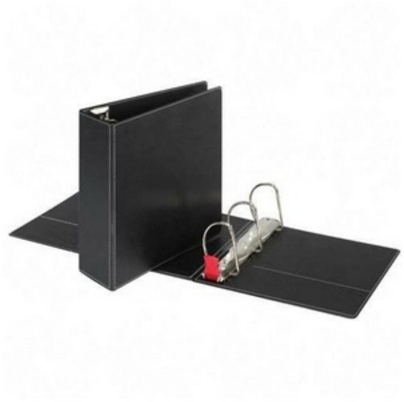

predicted

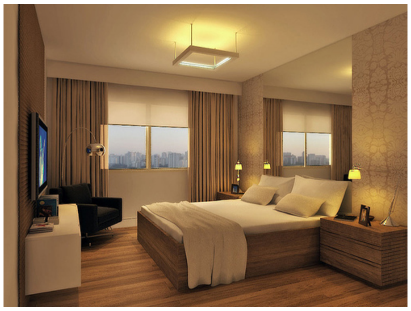

random control

shaman

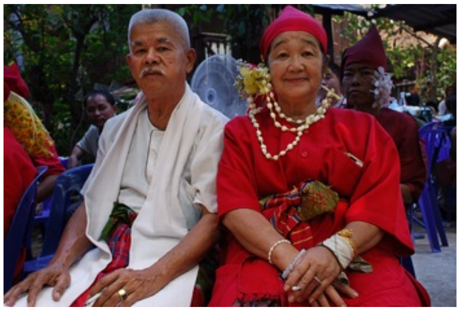

predicted

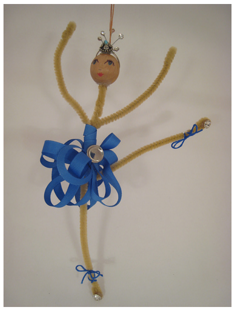

random control

program

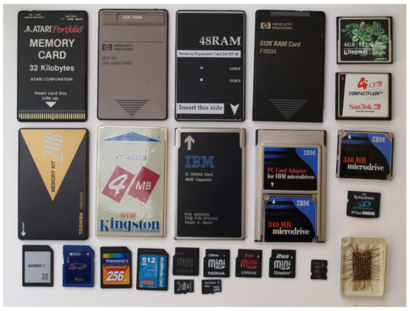

predicted

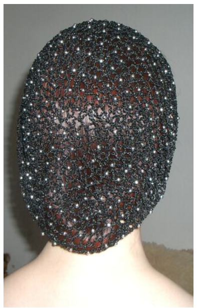

random control

ensure

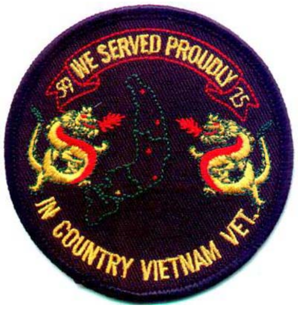

predicted

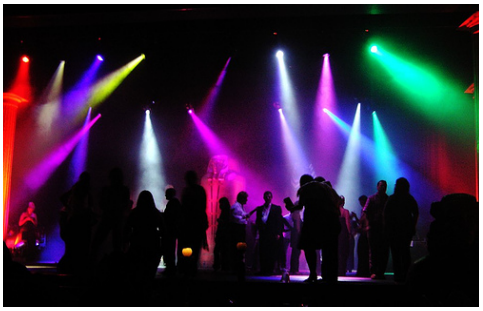

random control

consent

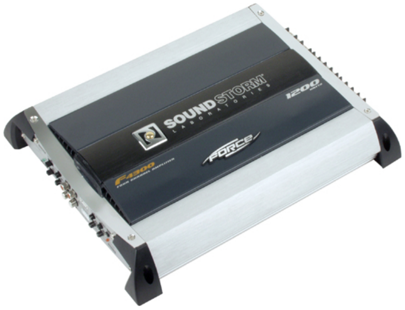

predicted

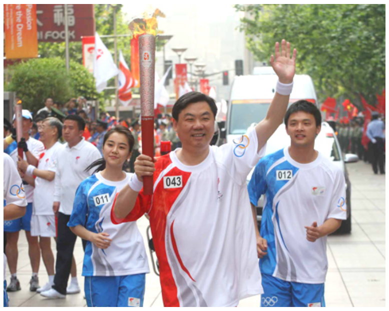

random control

hers

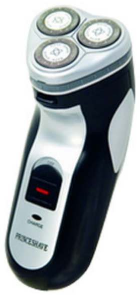

predicted

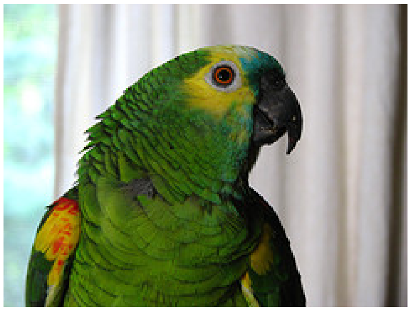

random control

werewolf

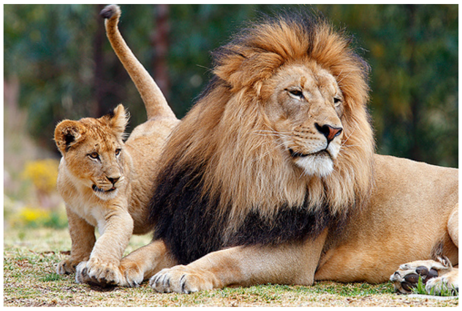

predicted

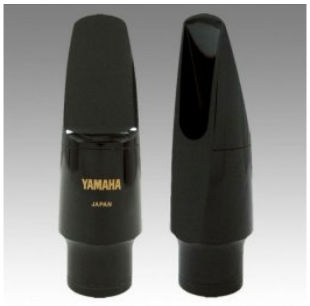

random control

ad

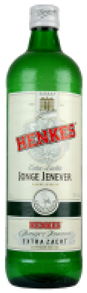

predicted

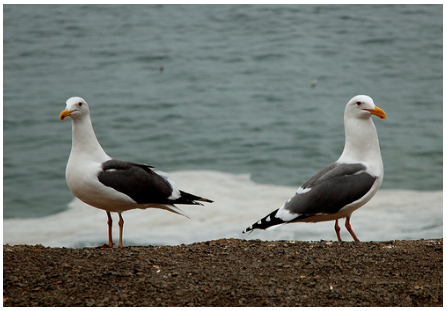

random control

size

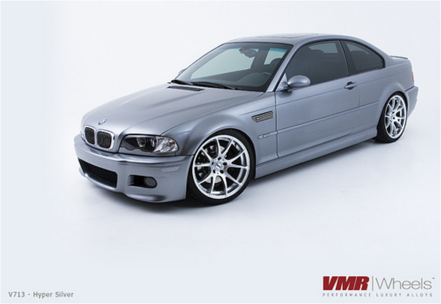

predicted

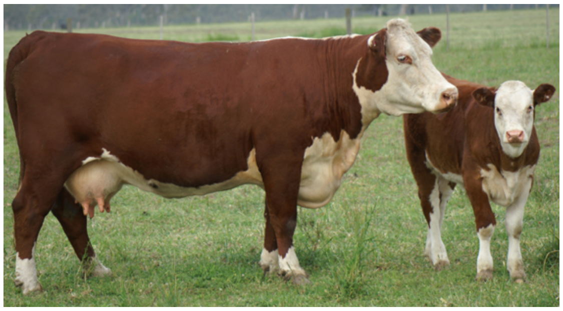

random control

zen

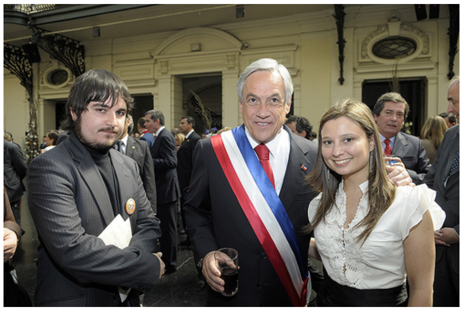

predicted

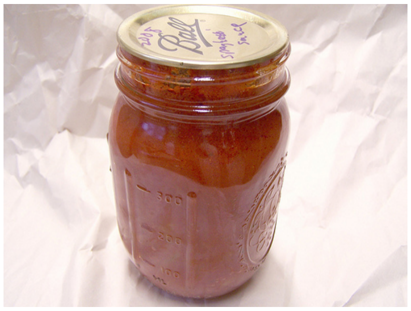

random control

currency

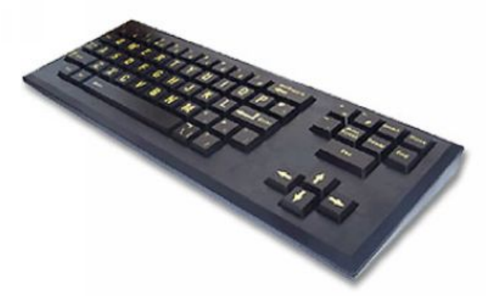

predicted

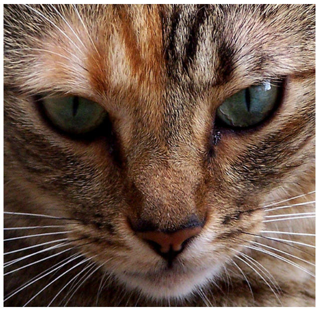

random control

sheriff

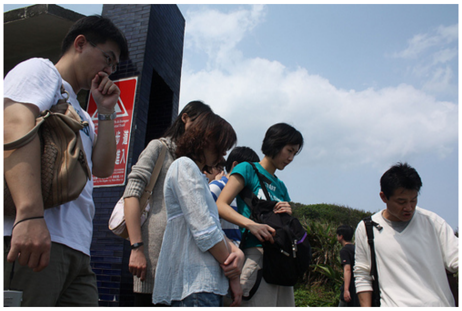

predicted

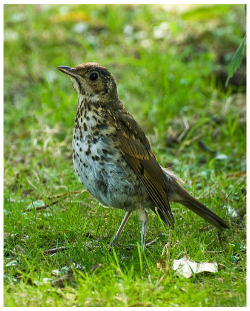

random control

playboy

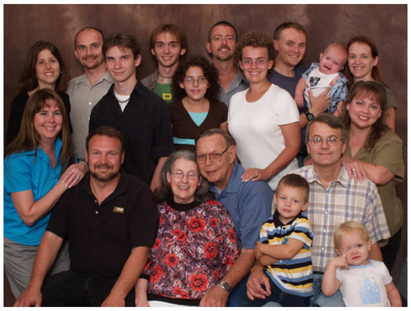

predicted

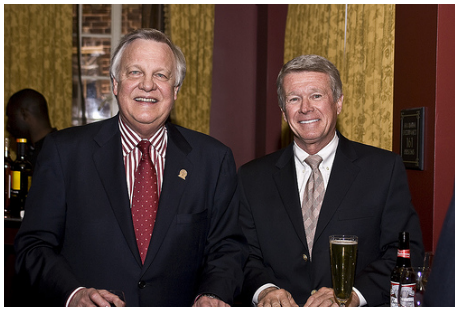

random control

calendar

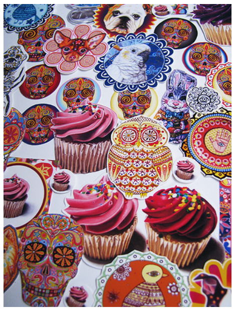

predicted

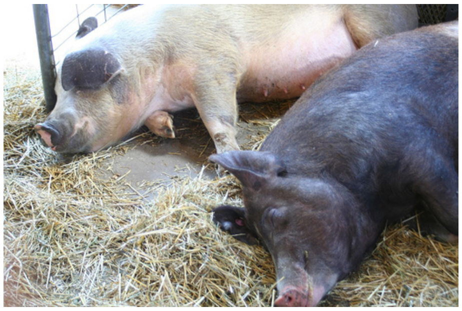

random control

yours

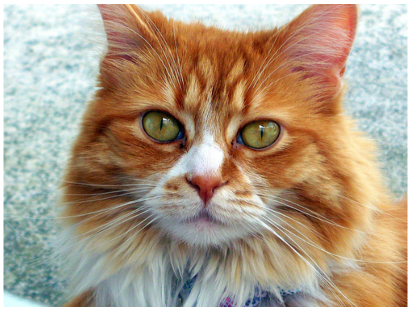

predicted

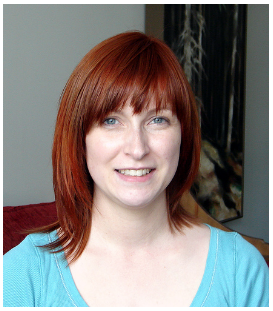

random control

role

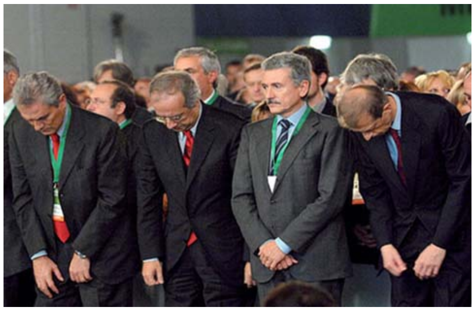

predicted

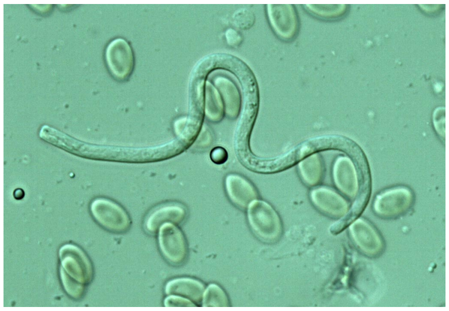

random control

letter

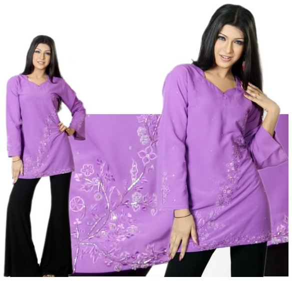

predicted

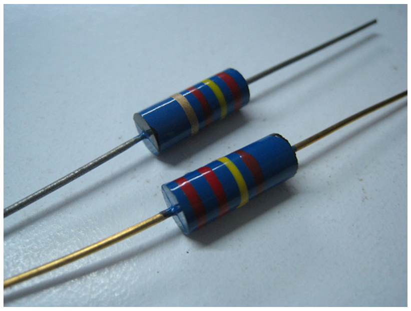

random control

countryside

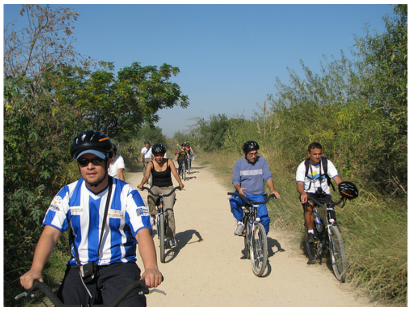

predicted

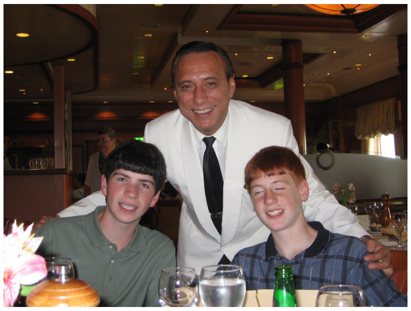

random control

pressure

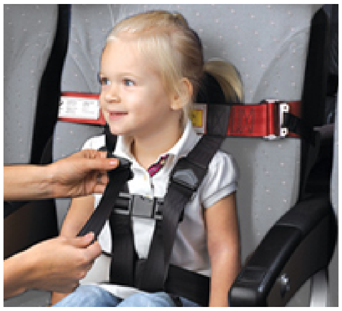

predicted

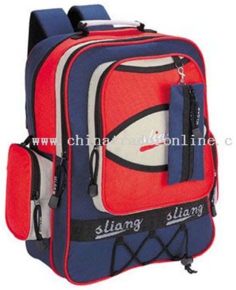

random control

overdose

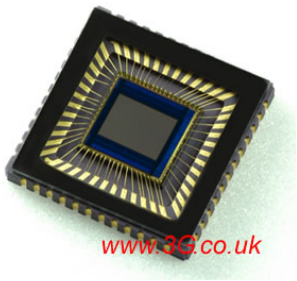

predicted

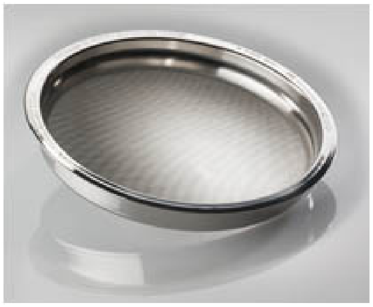

random control

archives

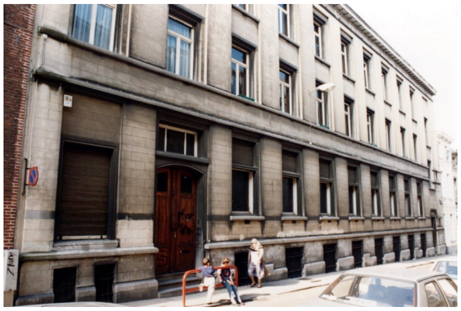

predicted

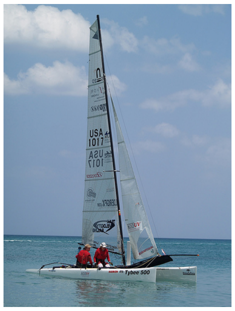

random control

muslim

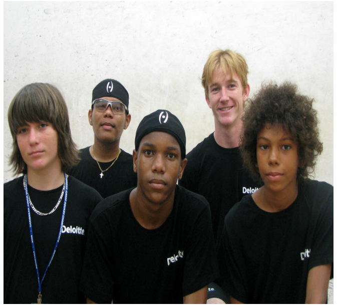

predicted

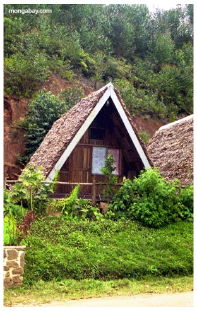

random control

backup

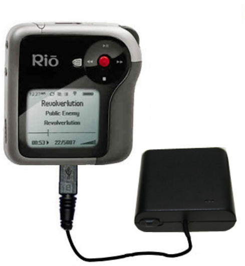

predicted

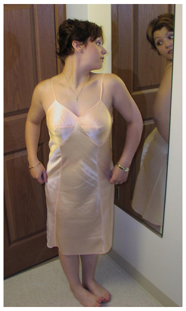

random control

lab

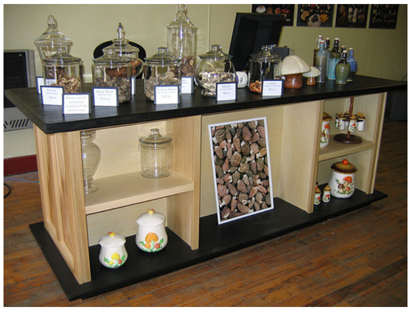
predicted

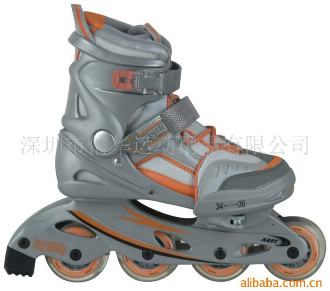

random control

nasal

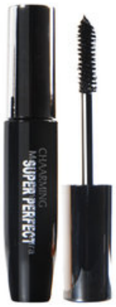

predicted

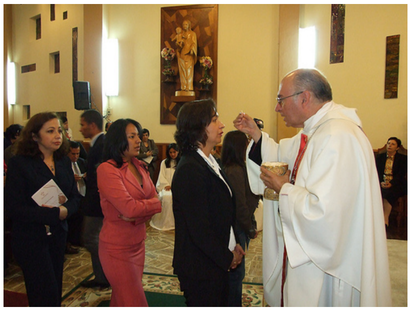
random control

specialty

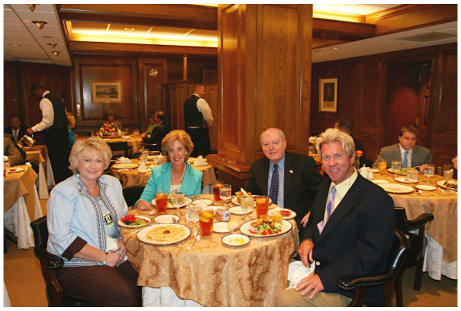
predicted

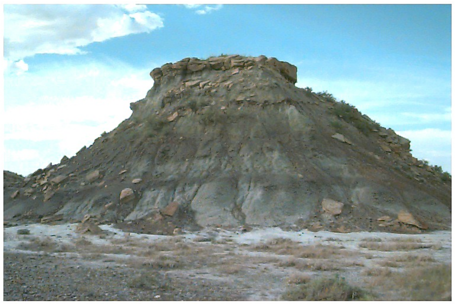
random control

laughter

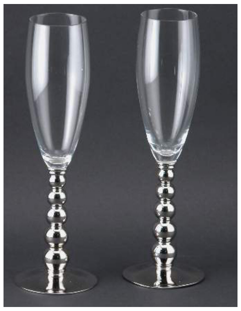

predicted

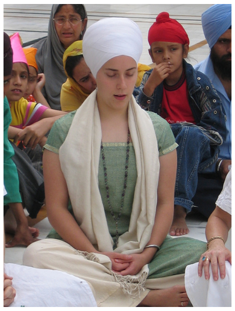

random control

pitching

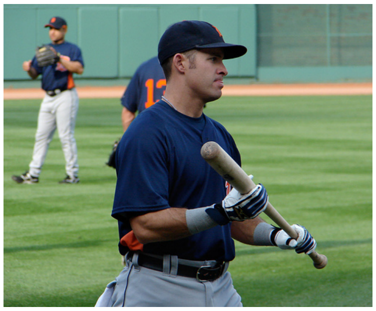

predicted

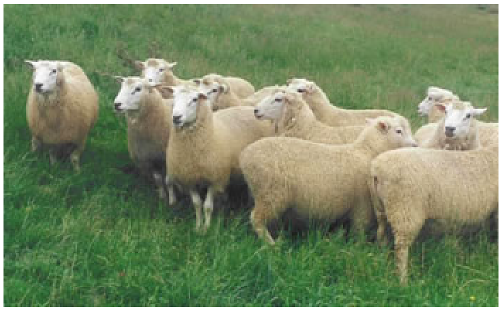

random control

graduating

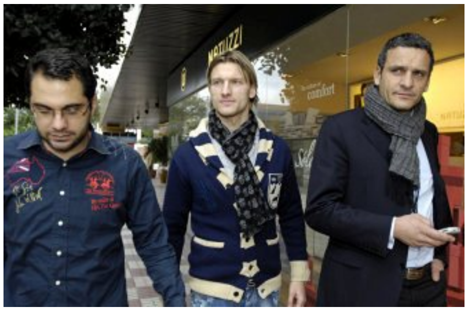

predicted

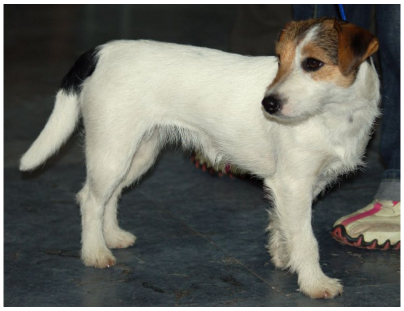

random control

debut

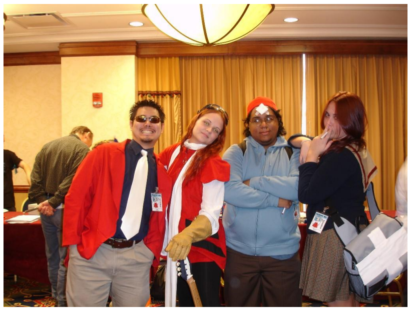

predicted

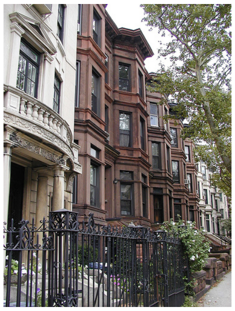

random control

faithful

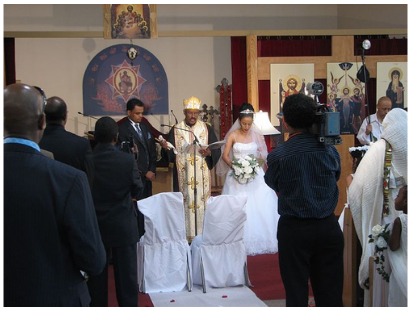

predicted

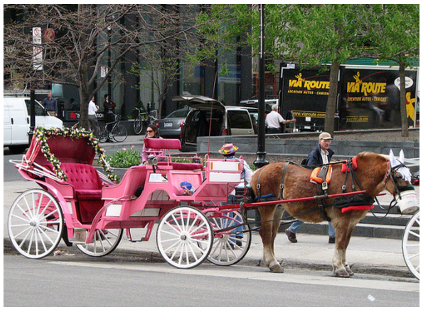

random control

herd

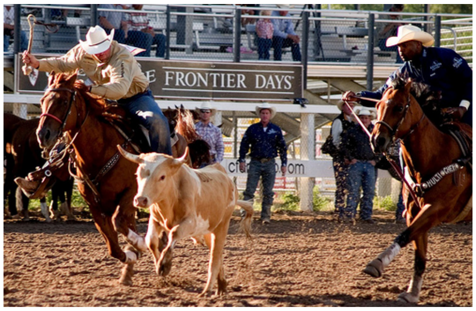

predicted

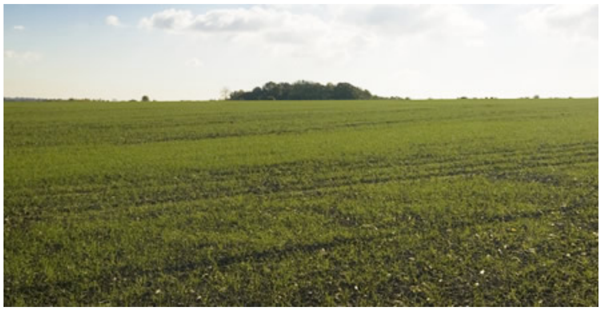

random control

eligible

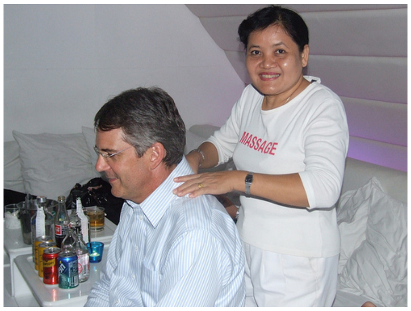

predicted

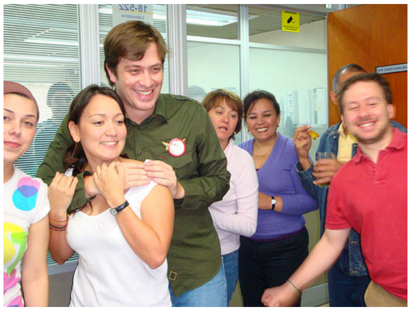

random control

following

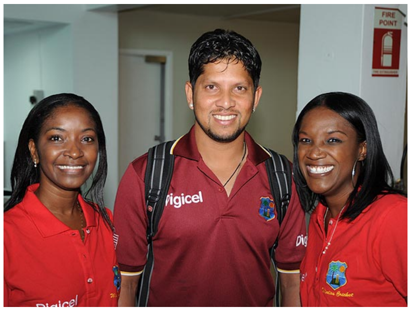

predicted

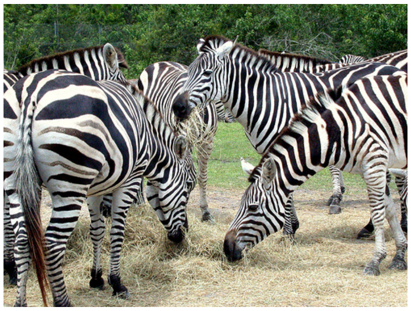

random control

consumer

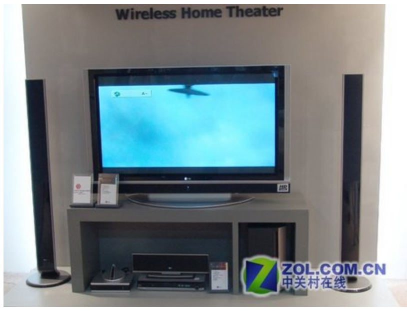

predicted

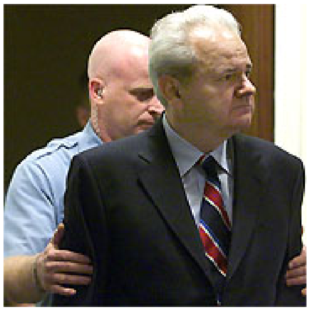

random control

receipt

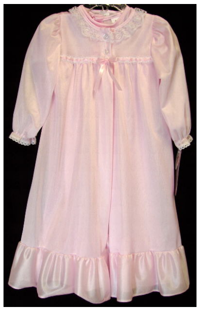

predicted

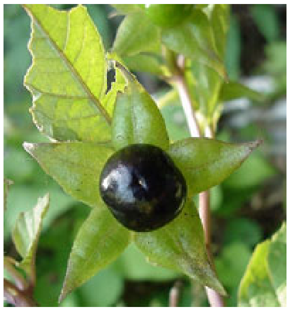

random control

analyst

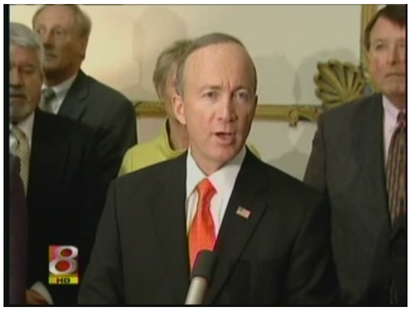

predicted

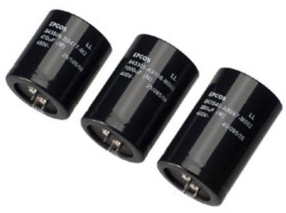

random control

soap

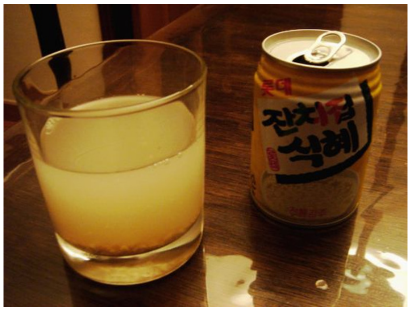

predicted

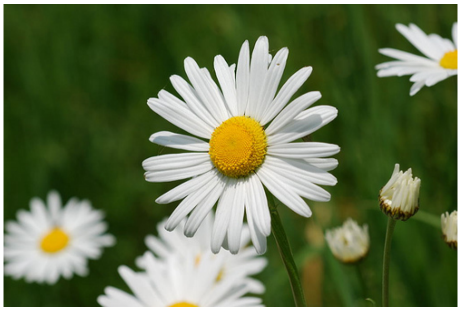

random control

solo

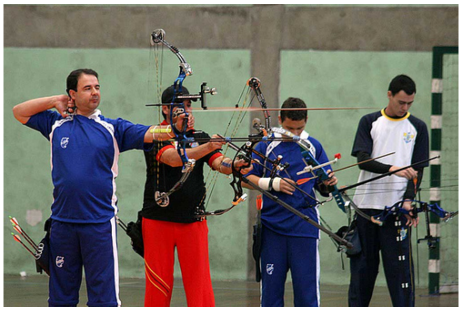

predicted

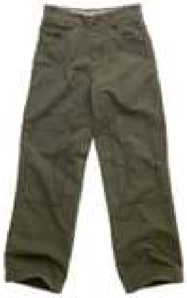

random control

favorite

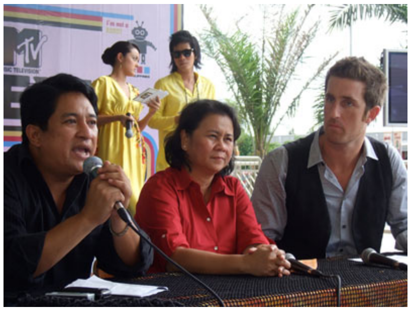

predicted

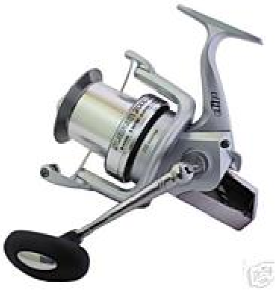

random control

starboard

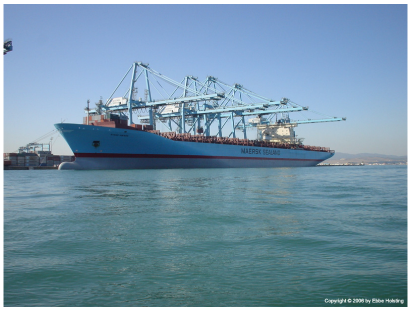

predicted

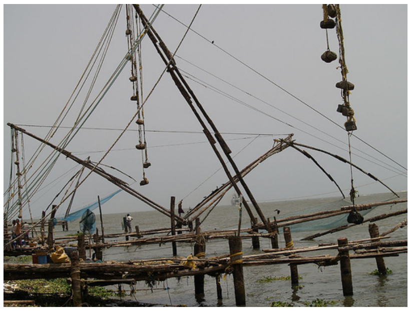

random control

echo

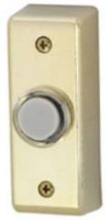

predicted

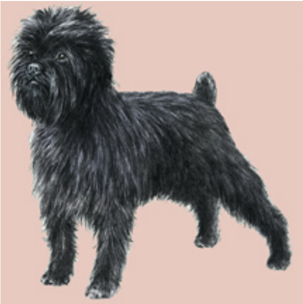

random control

extortion

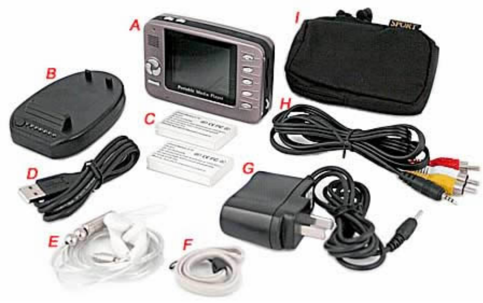

predicted

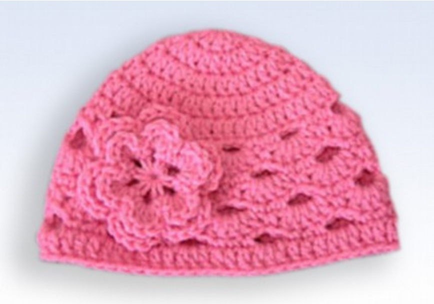

random control

microphone

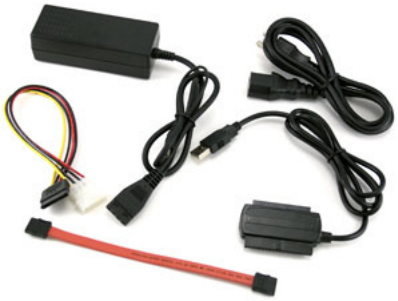

predicted

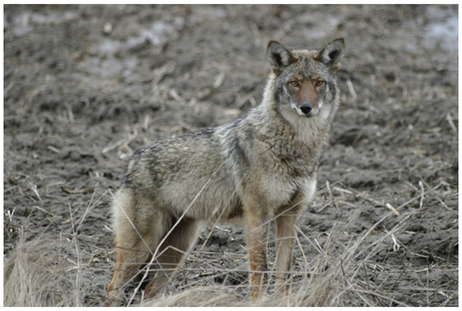

random control

sympathy

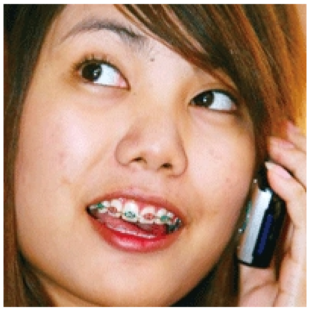

predicted

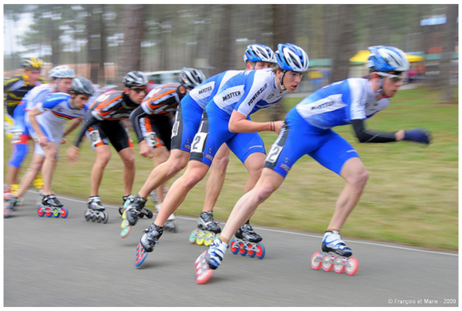

random control

visual

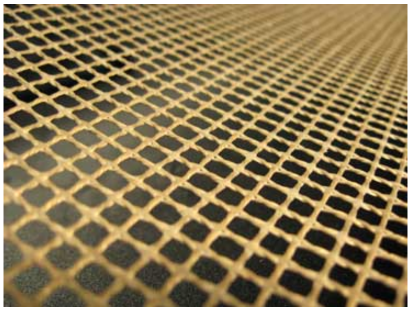

predicted

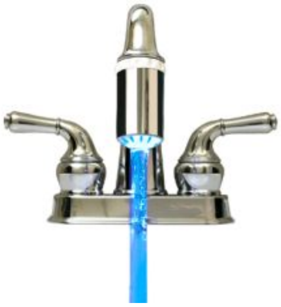

random control

design

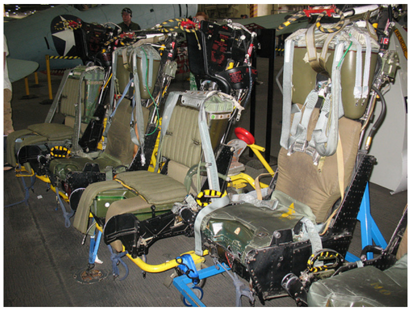

predicted

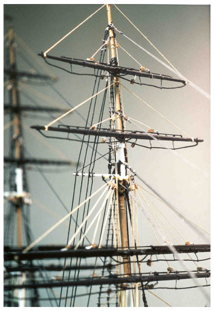

random control

concern

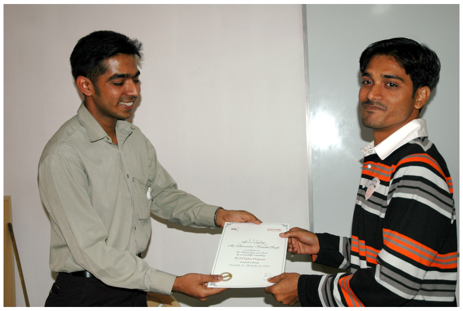

predicted

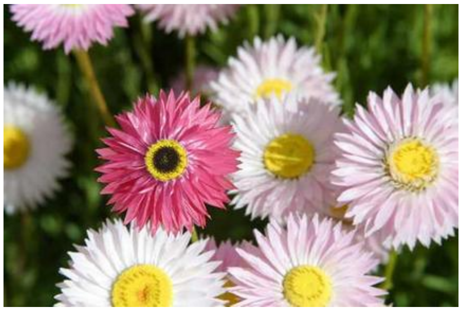

random control

universe

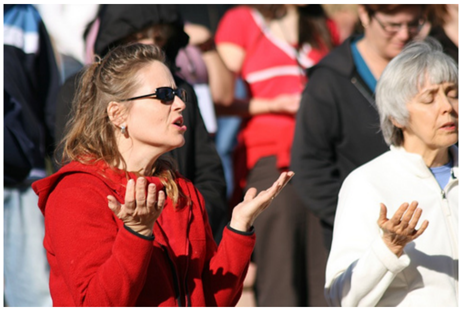

predicted

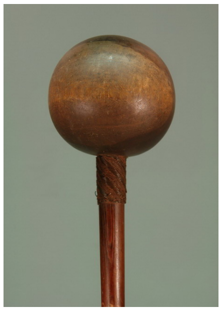

random control

lust

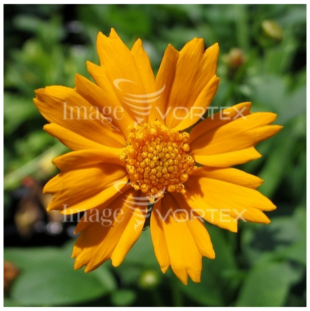

predicted

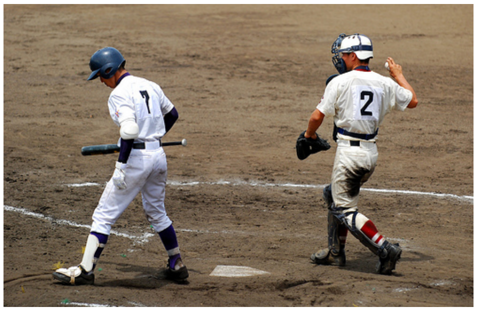

random control

par

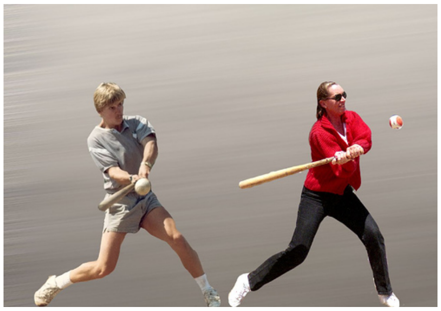

predicted

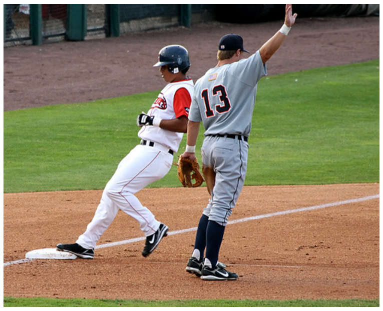

random control

mood

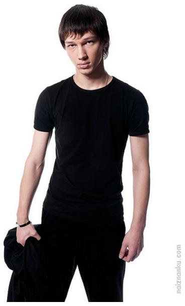

predicted

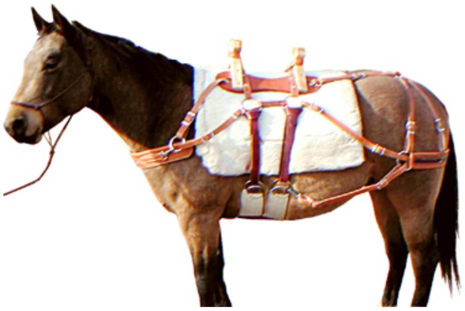

random control

terminal

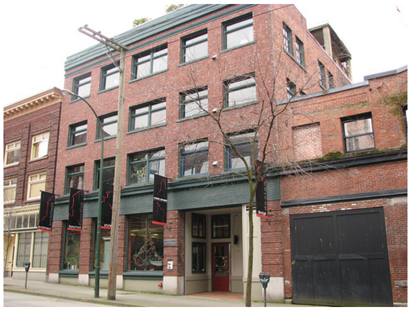

predicted

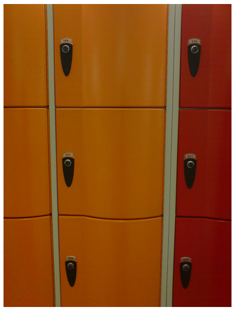

random control

diploma

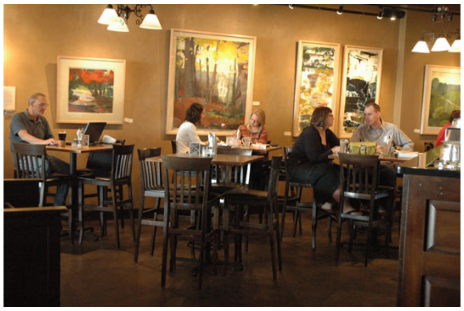

predicted

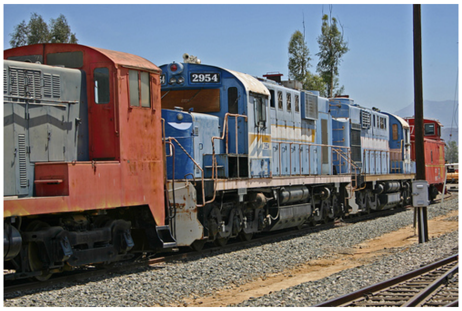

random control

performance

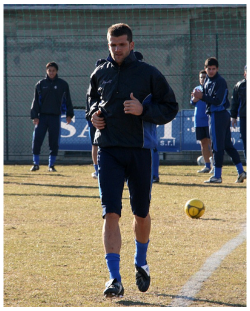

predicted

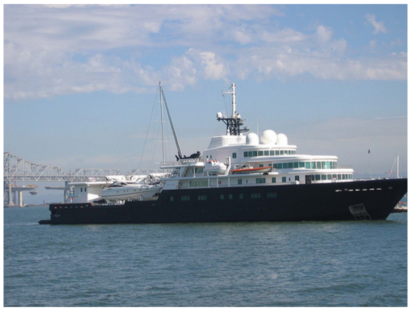

random control

chief

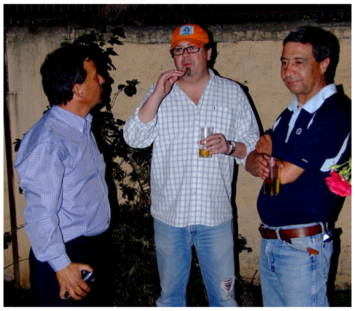

predicted

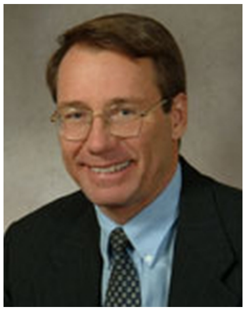

random control

meeting

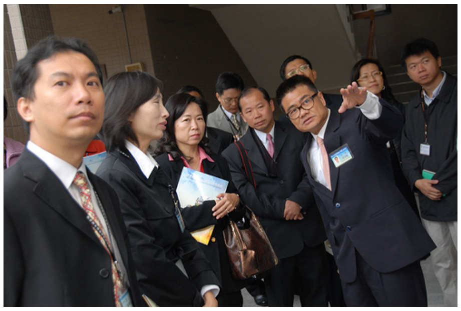

predicted

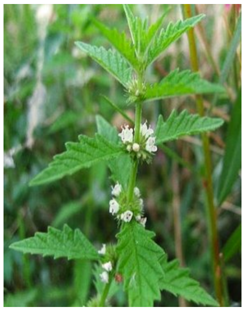

random control

commercial

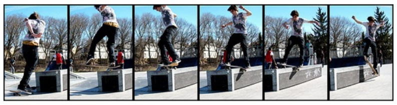

predicted

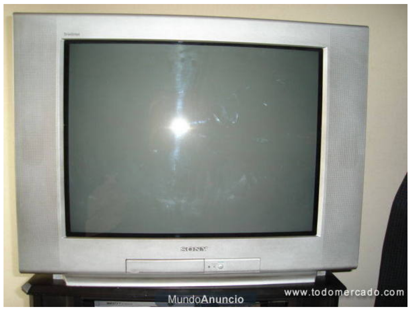

random control

teen

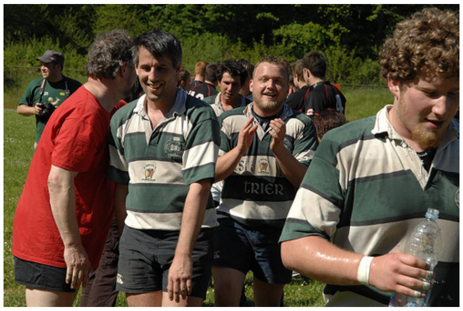

predicted

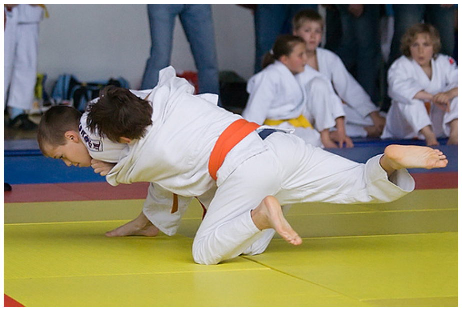

random control

sorrow

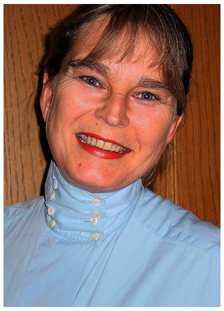

predicted

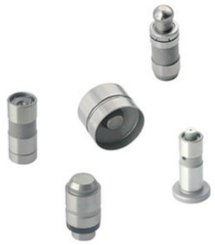

random control

headache

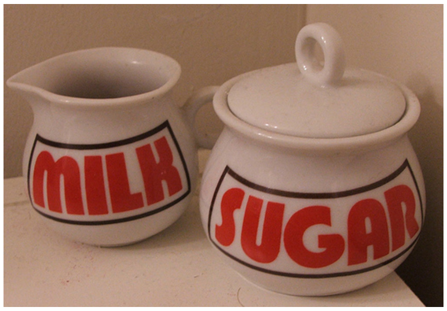

predicted

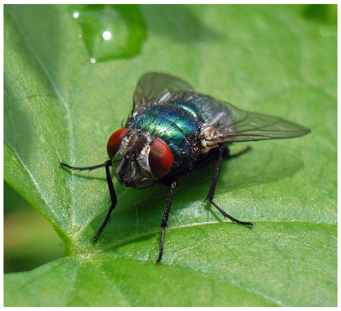

random control

capacity

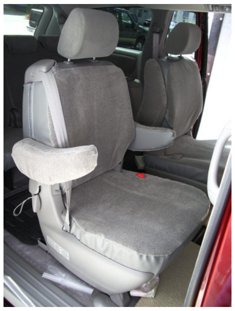

predicted

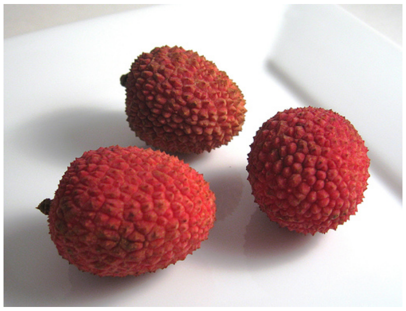

random control

mining

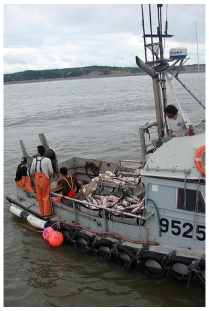

predicted

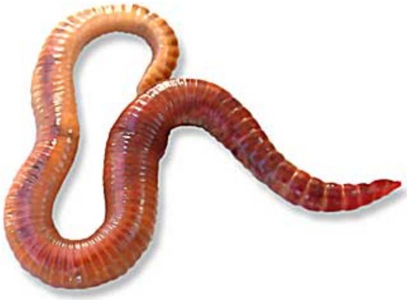

random control

belle

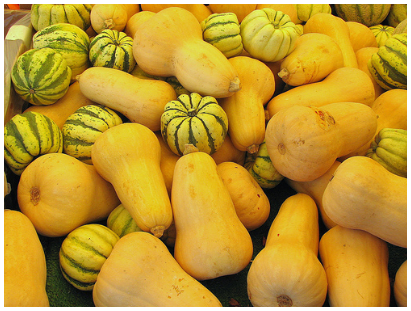

predicted

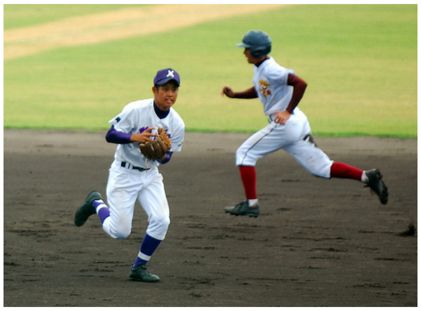

random control

immortal

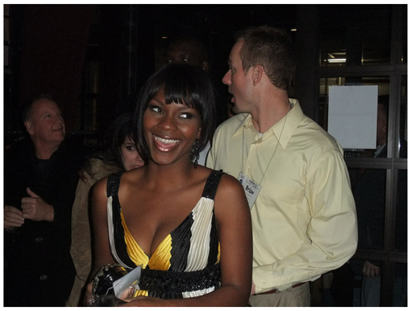

predicted

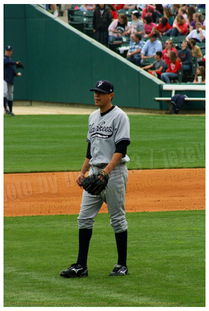

random control

goodwill

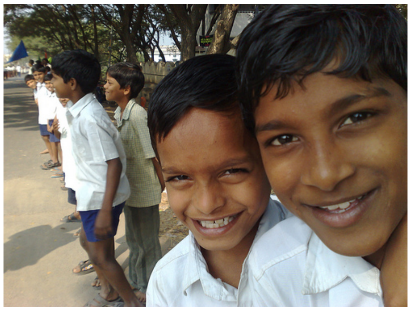

predicted

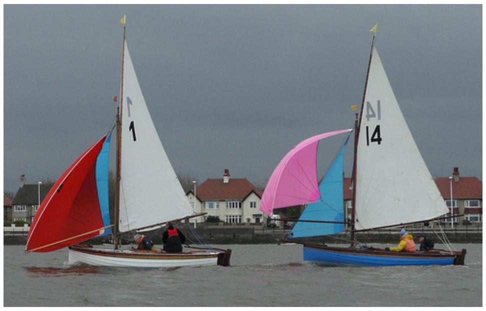

random control

organic

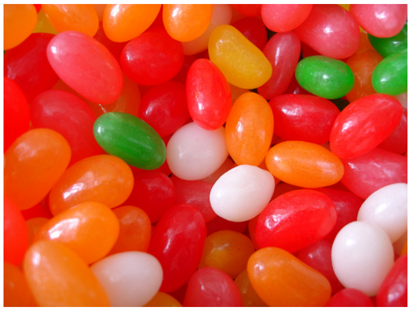

predicted

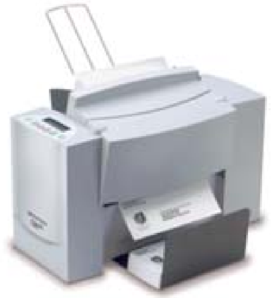

random control

slam

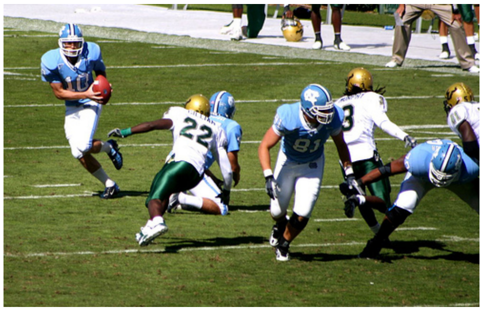

predicted

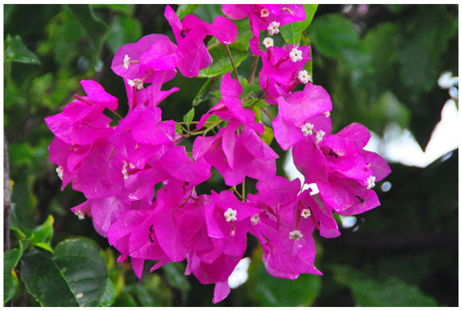

random control

dwarf

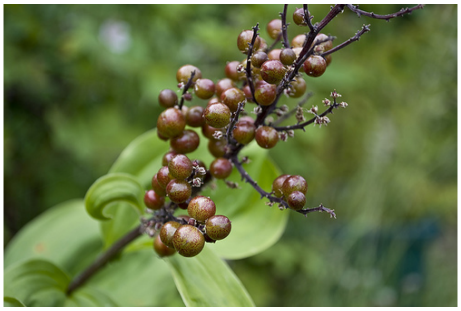

predicted

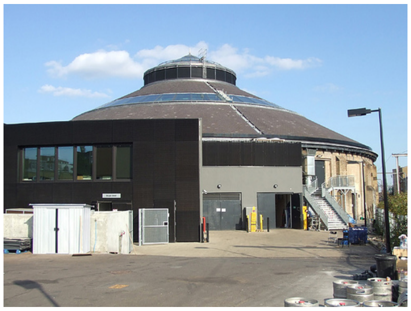

random control

pint

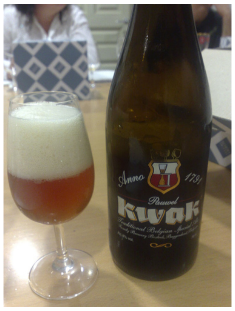

predicted

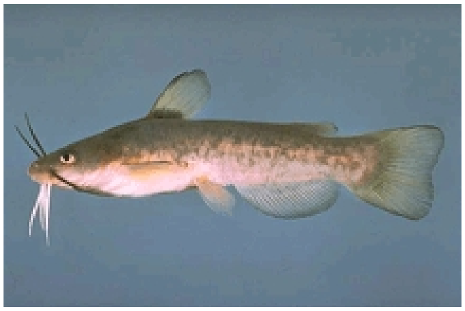

random control

rub

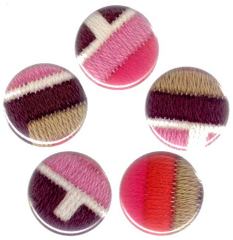

predicted

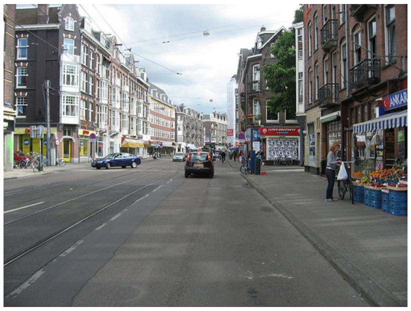

random control

chauffeur

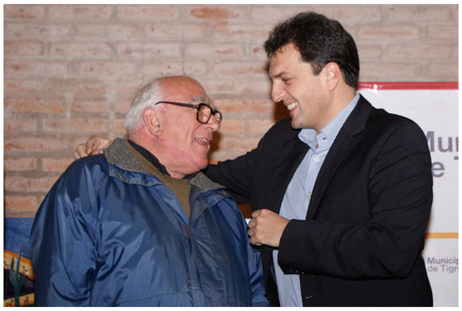

predicted

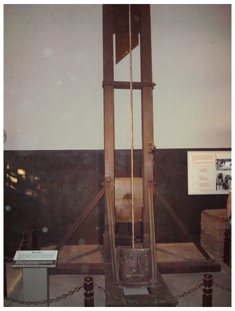

random control

original

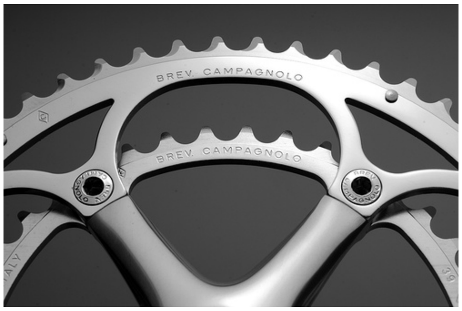

predicted

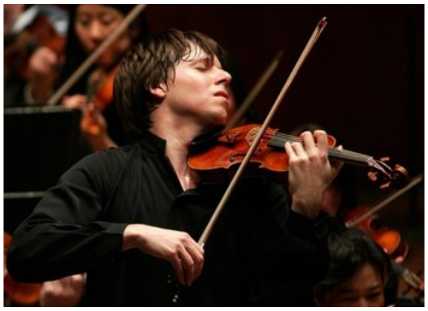

random control

nickname

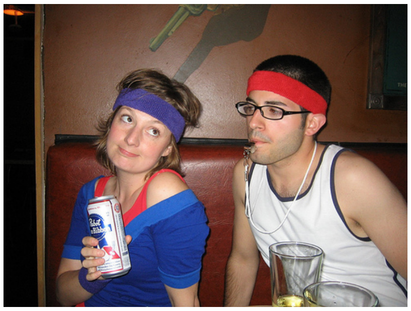

predicted

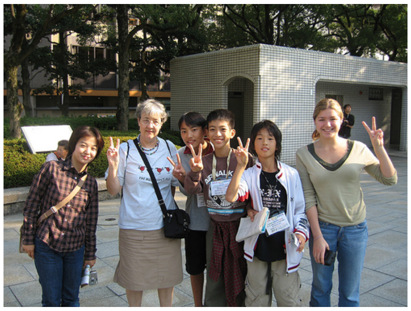

random control

equation

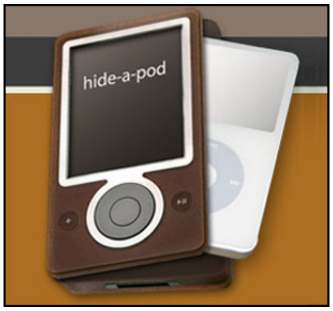

predicted

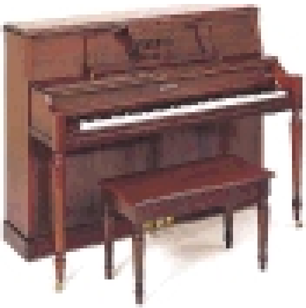

random control

report

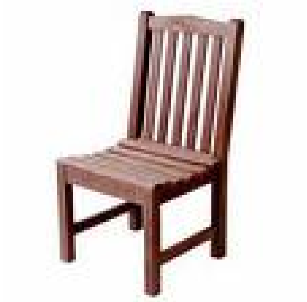

predicted

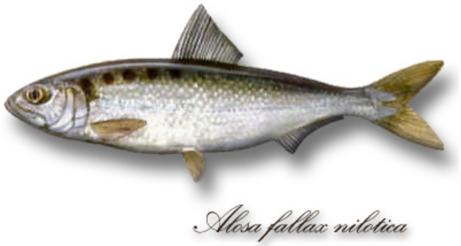

random control

loser

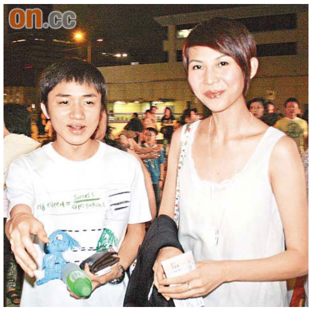

predicted

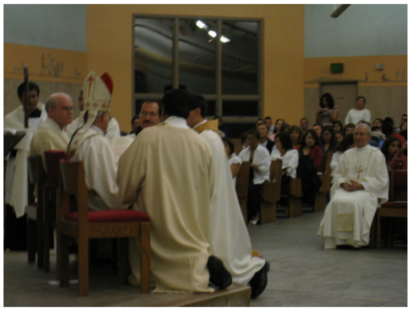

random control

funk

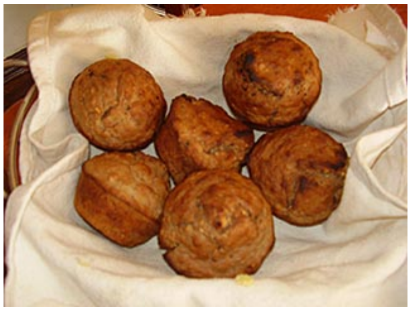

predicted

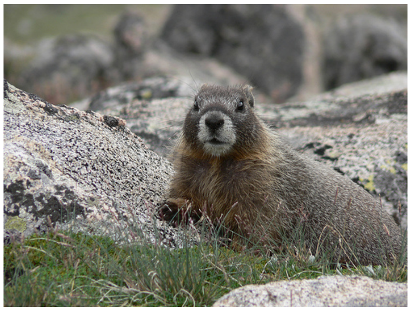

random control

version

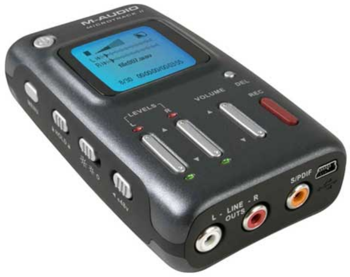

predicted

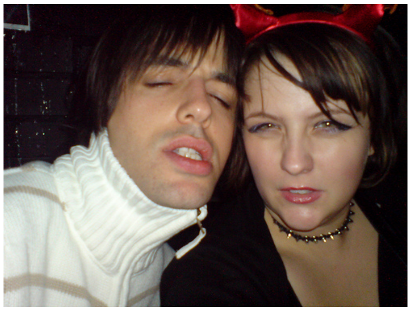

random control

commerce

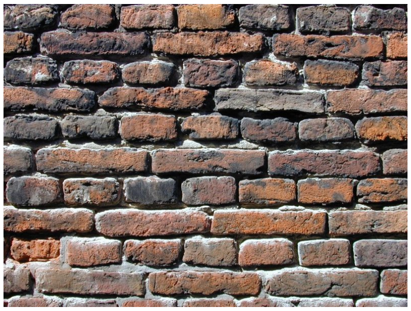

predicted

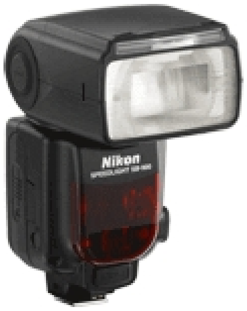

random control

mayhem

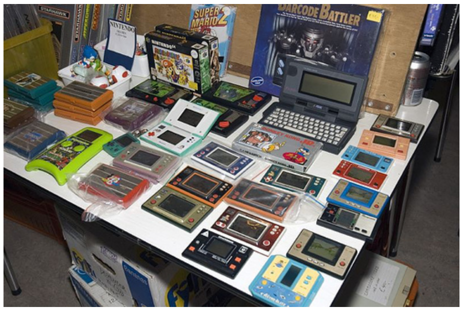

predicted

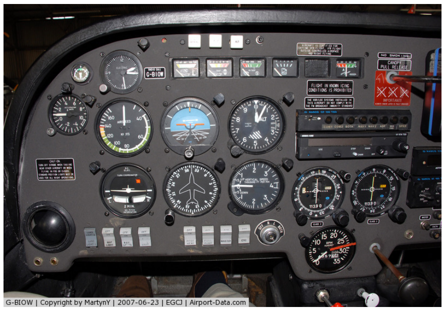

random control

technique

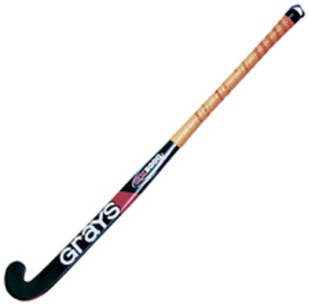

predicted

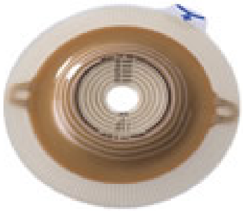

random control

yourselves

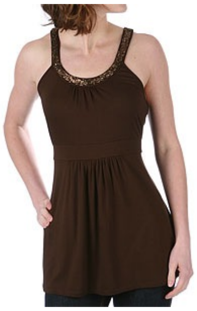

predicted

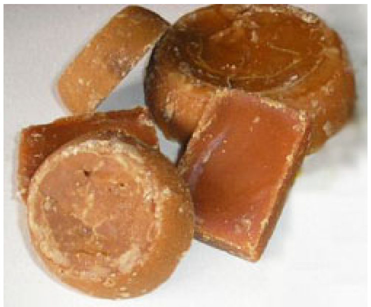

random control

slash

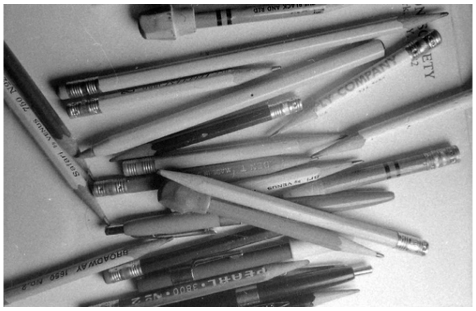

predicted

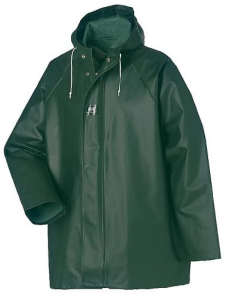

random control

correspondence

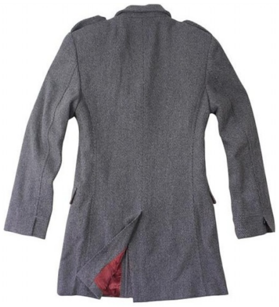

predicted

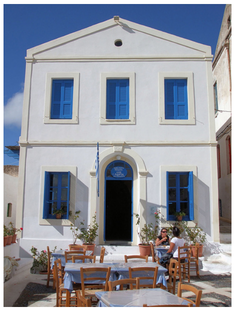

random control

behavior

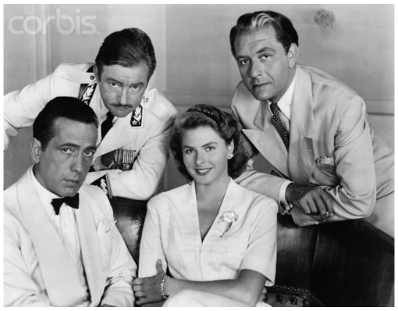

predicted

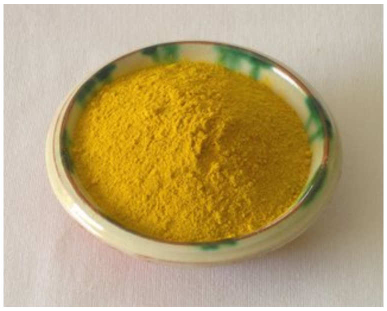

random control

flock

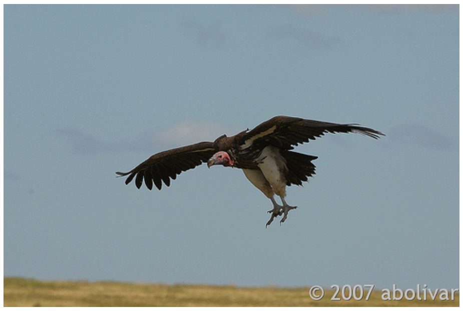

predicted

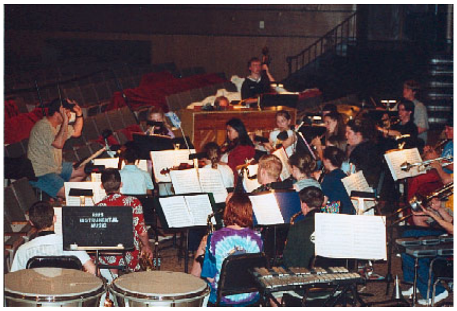

random control

mischief

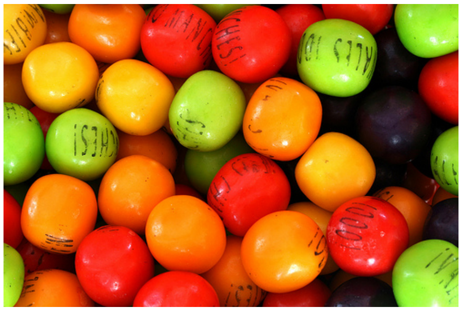

predicted

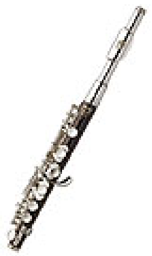

random control

basis

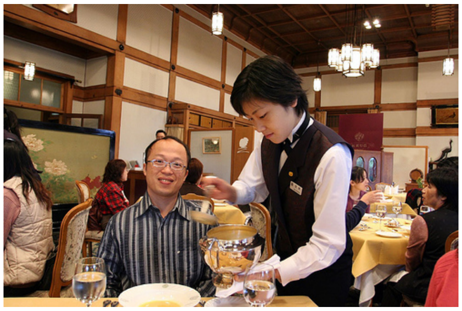

predicted

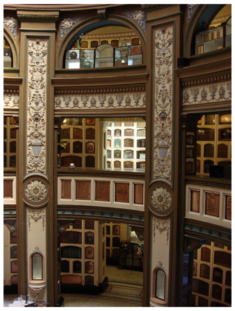

random control

blossom

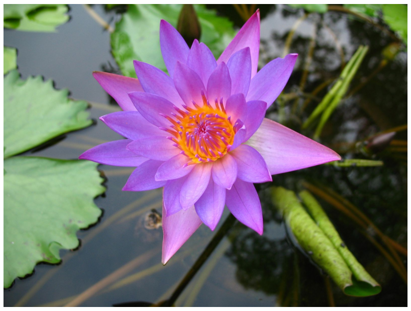

predicted

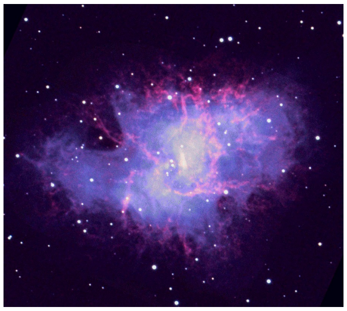

random control

cannot

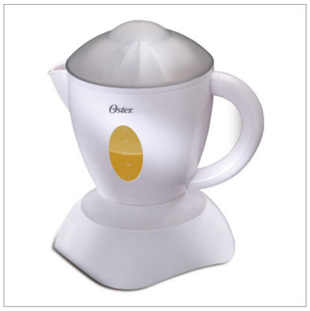

predicted

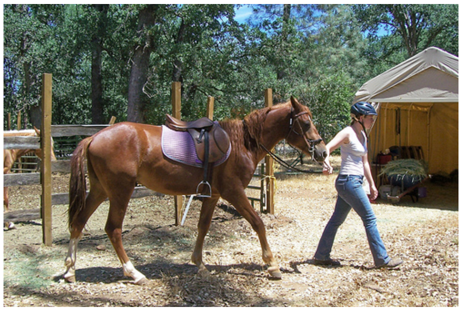

random control

nod

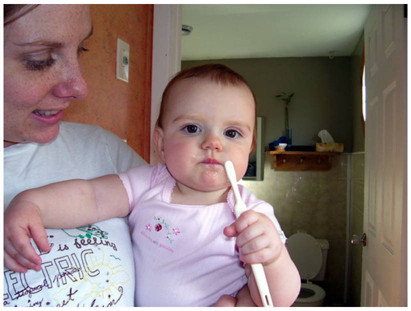

predicted

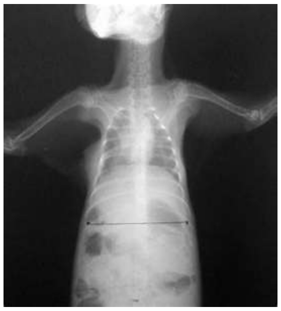

random control

fright

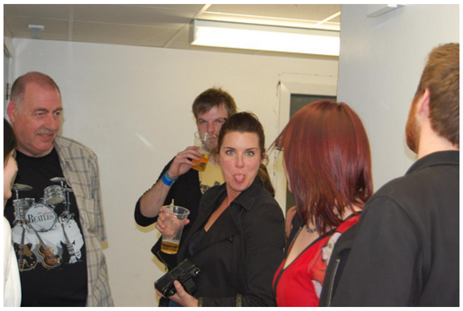

predicted

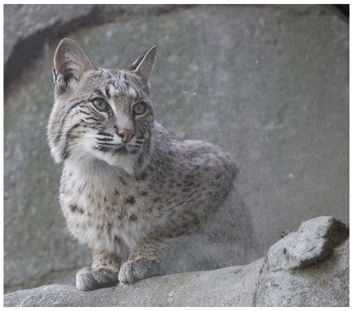

random control

ugh

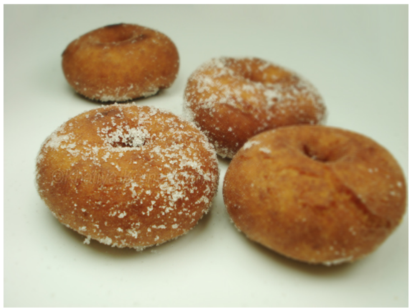

predicted

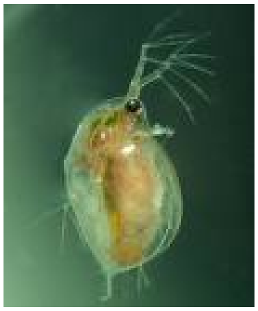

random control

accidental

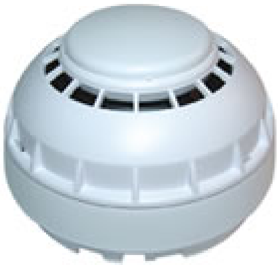

predicted

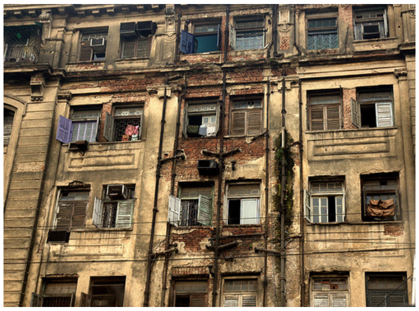

random control

employment

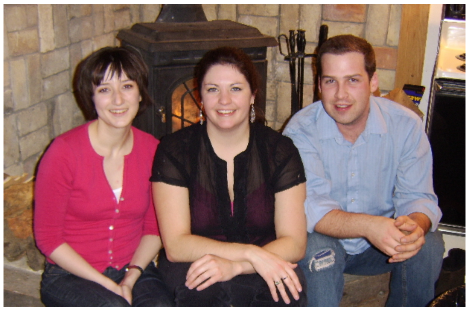

predicted

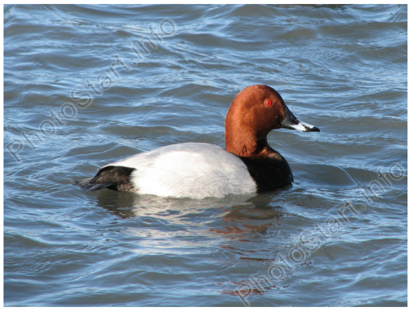

random control

petition

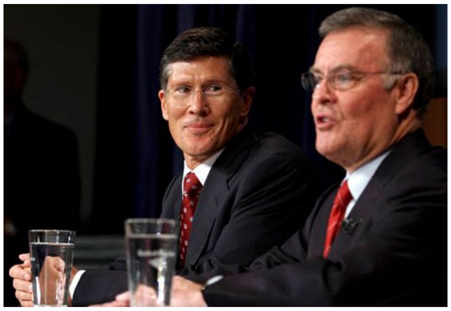

predicted

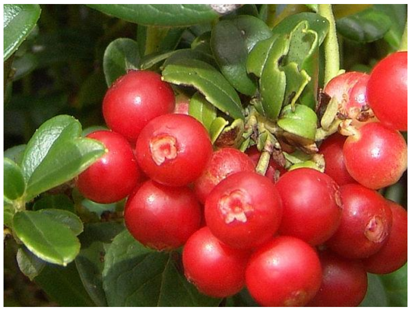

random control

history

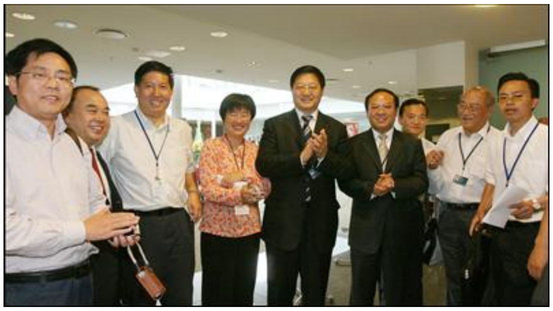

predicted

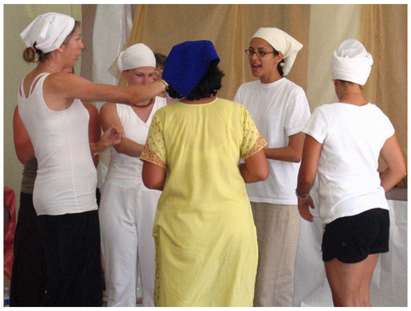

random control

simple

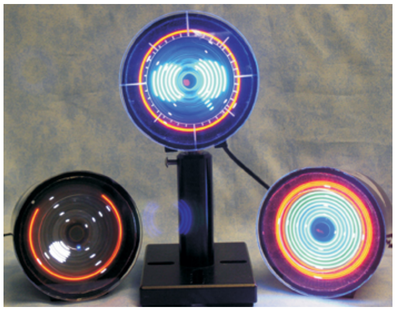

predicted

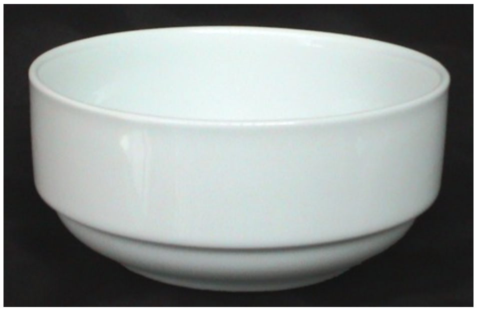

random control

mythology

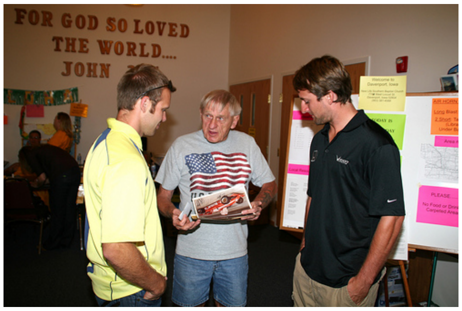

predicted

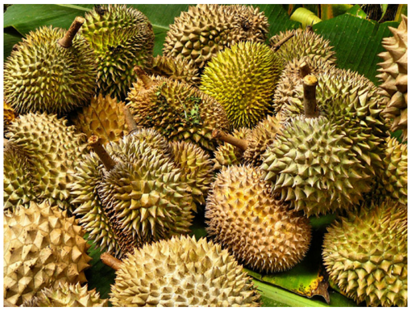

random control

percentage

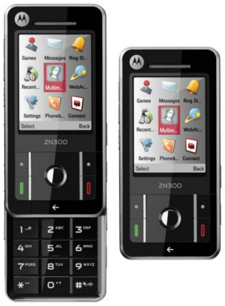

predicted

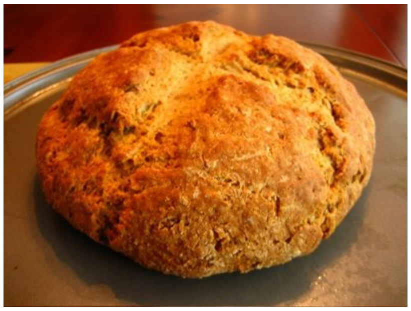

random control

undress

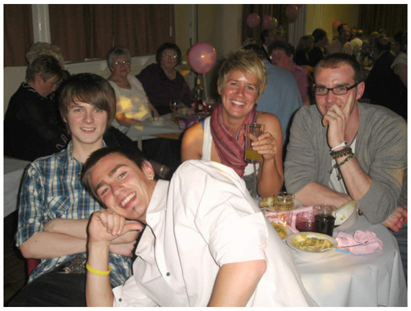

predicted

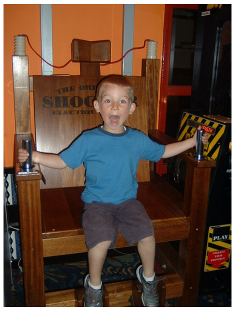

random control

weekend

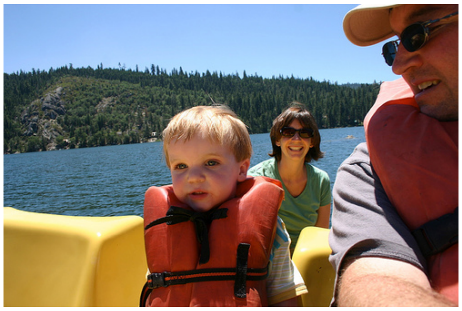

predicted

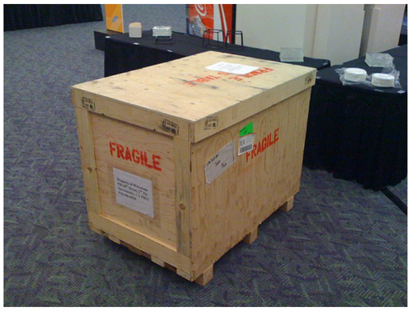

random control

morgue

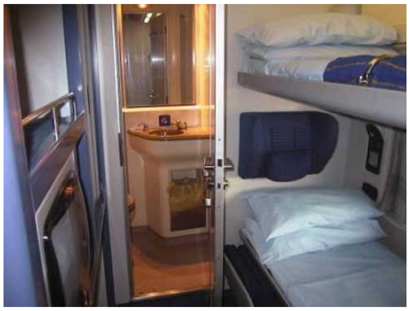

predicted

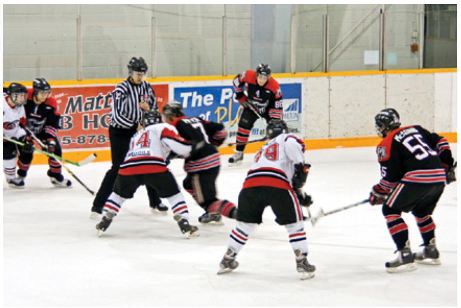

random control

darkness

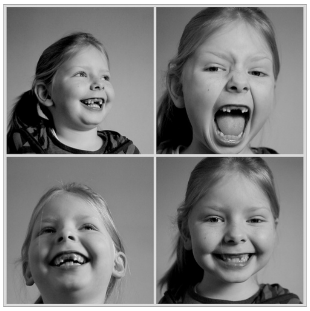

predicted

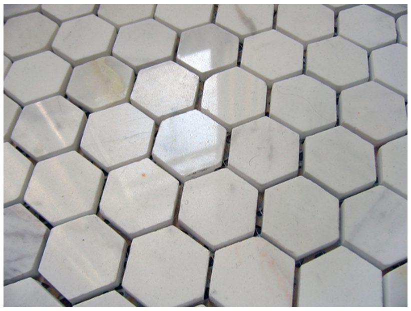

random control

bravery

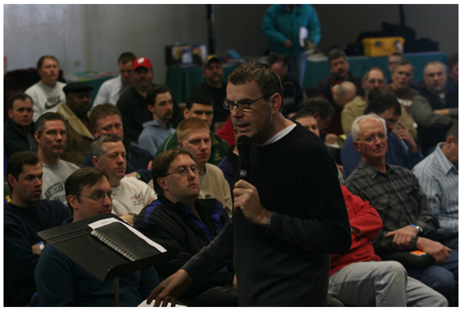

predicted

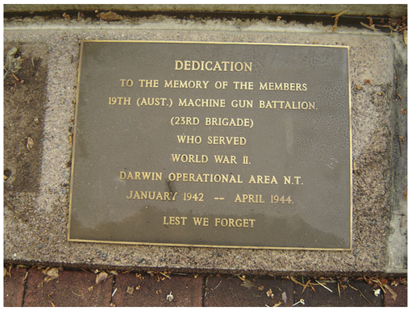

random control

folk

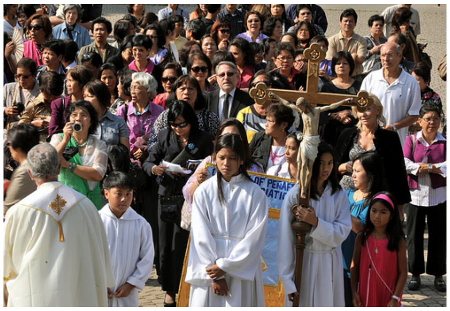

predicted

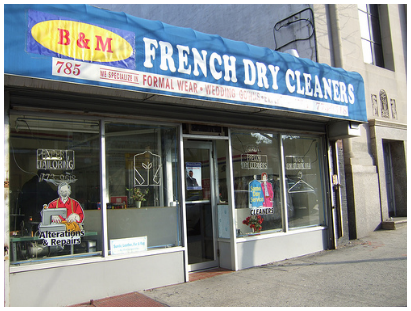

random control

viewing

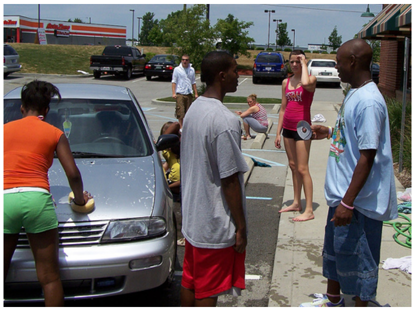

predicted

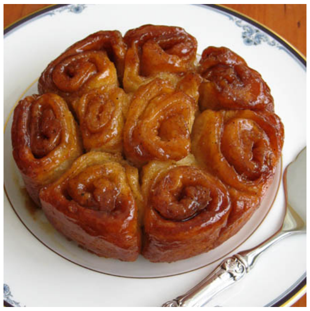

random control

distraction

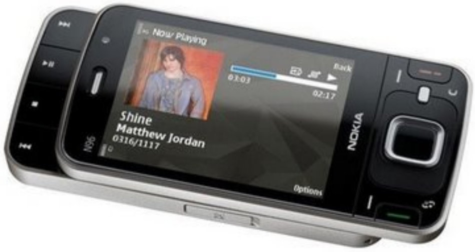

predicted

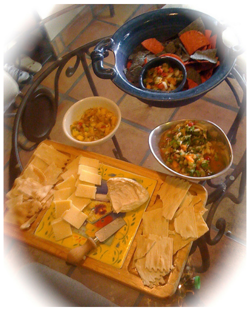

random control

loss

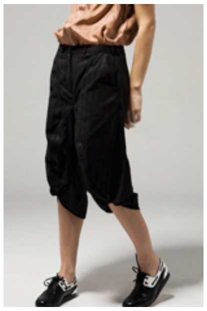

predicted

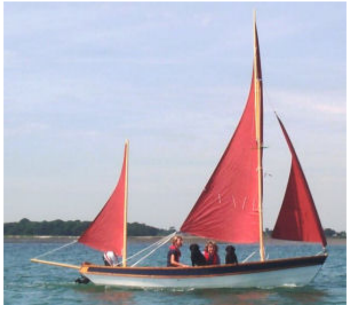

random control

dependent

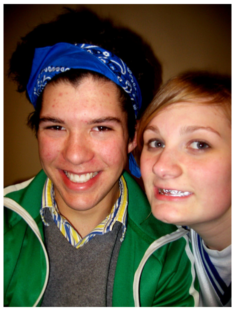

predicted

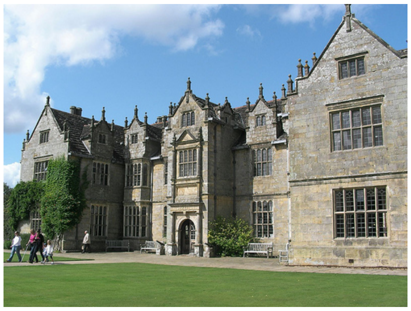

random control

rookie

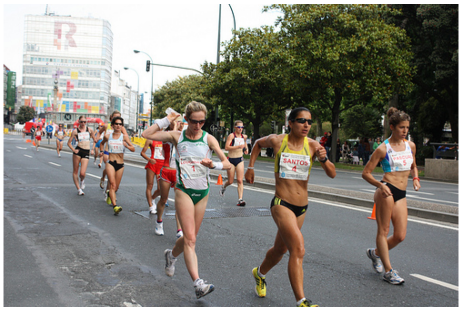

predicted

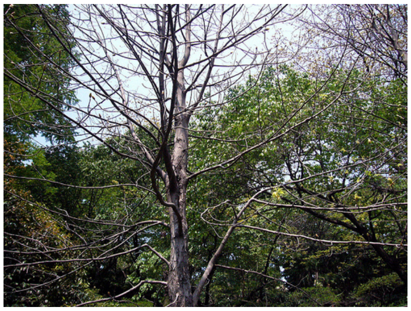

random control

hoax

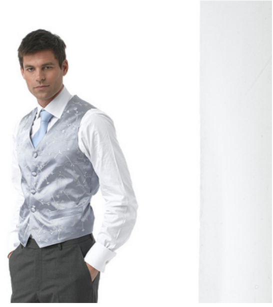

predicted

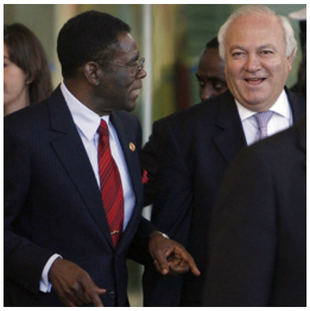

random control

handshake

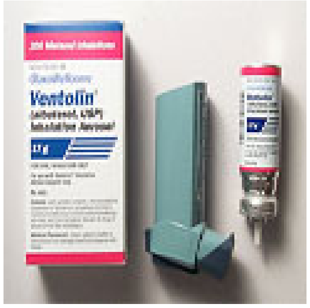

predicted

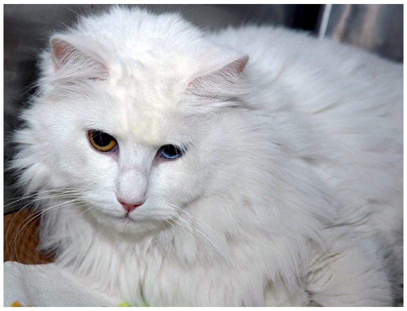

random control

auxiliary

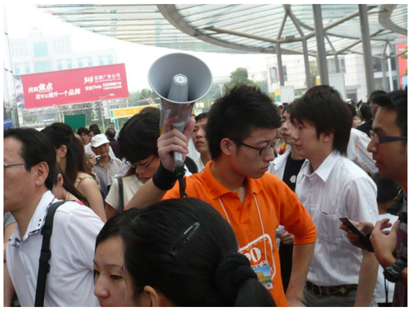

predicted

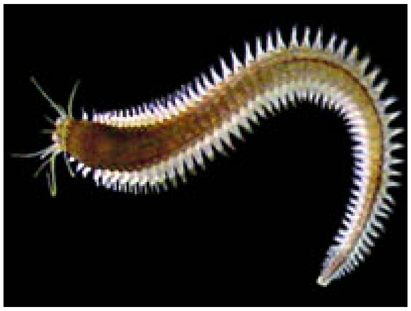

random control

airline

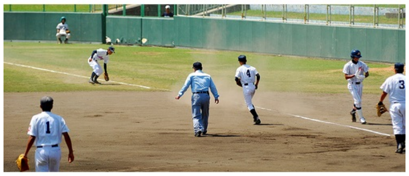

predicted

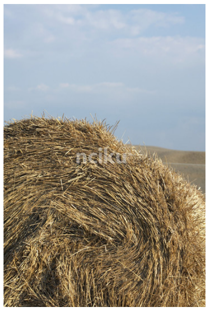

random control

keeper

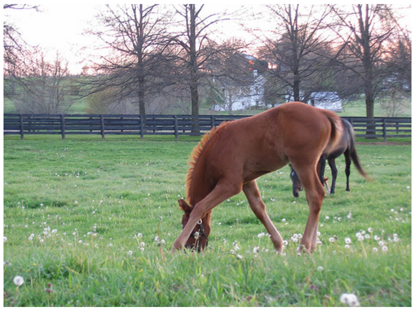

predicted

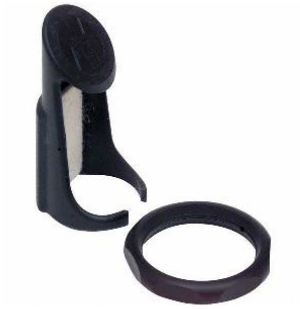

random control

troop

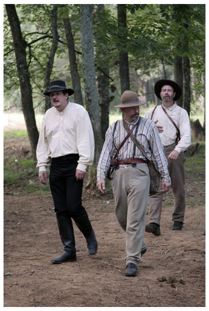

predicted

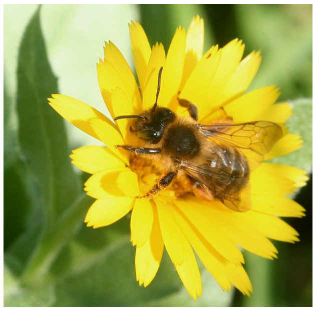

random control

cake

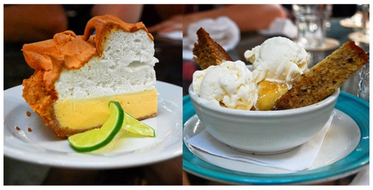

predicted

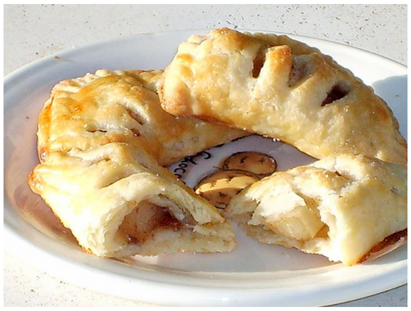

random control

schedule

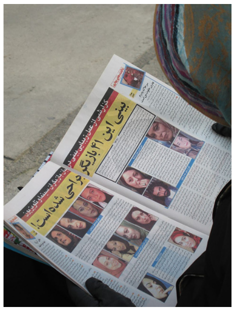

predicted

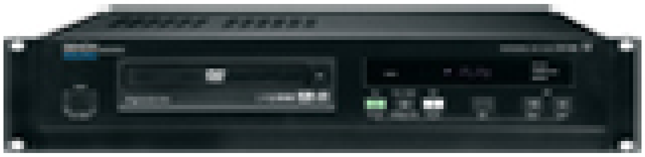

random control

bookie

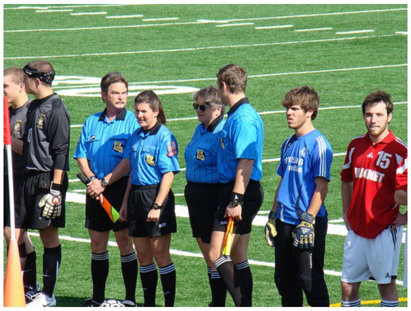

predicted

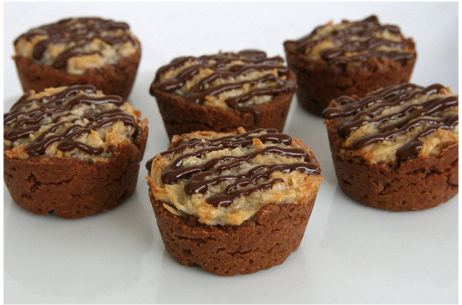

random control

cola

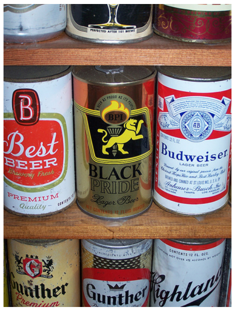

predicted

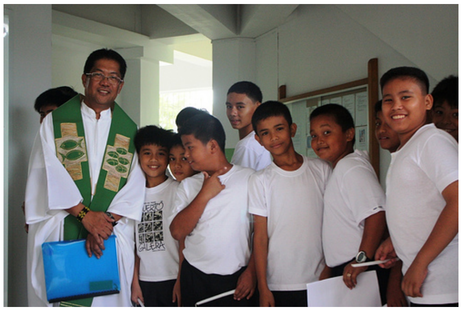

random control

overseas

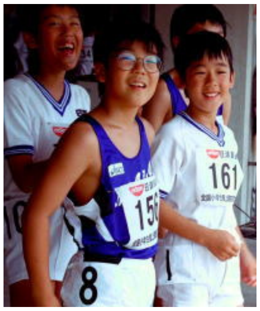

predicted

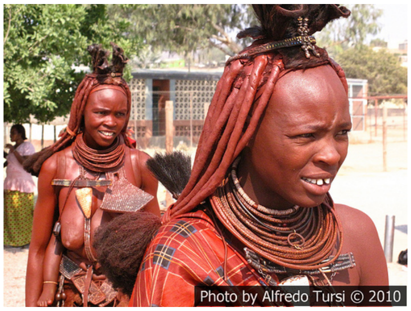

random control

saline

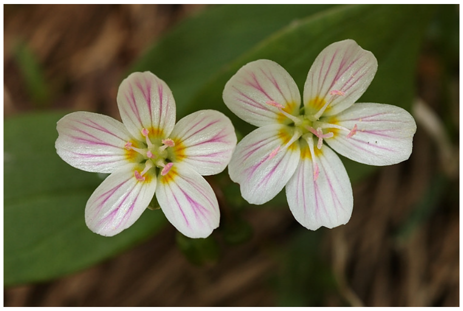

predicted

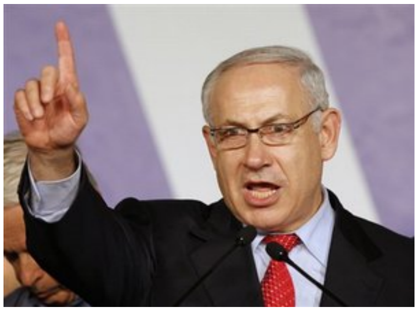

random control

joke

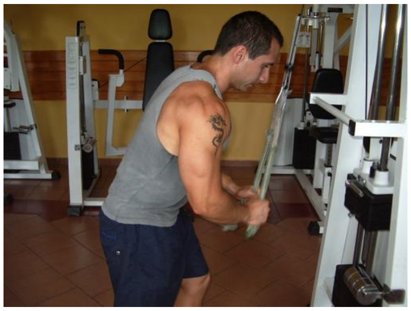

predicted

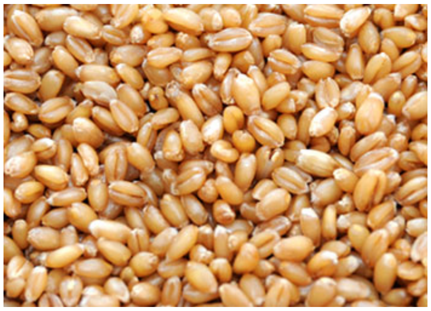

random control

product

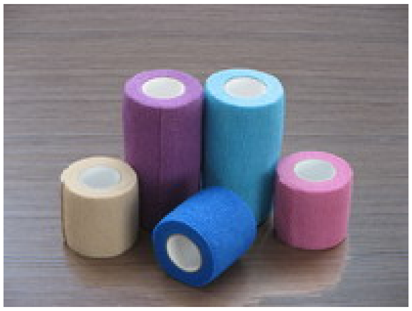

predicted

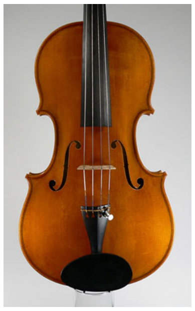

random control

romance

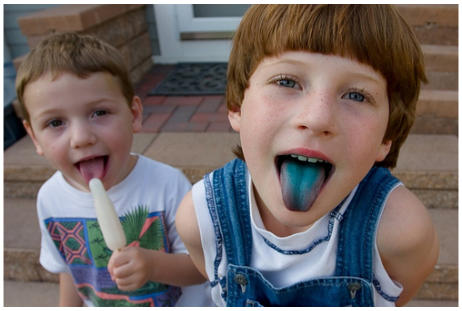

predicted

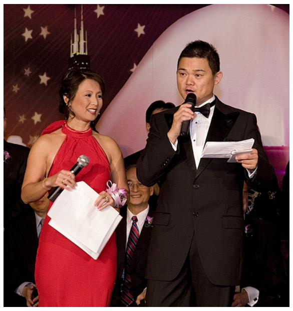

random control

platoon

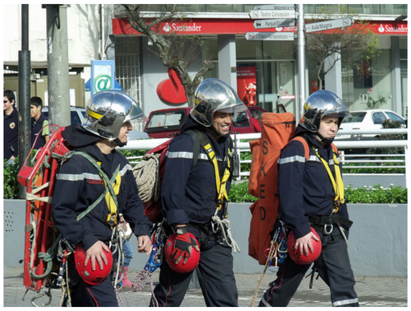

predicted

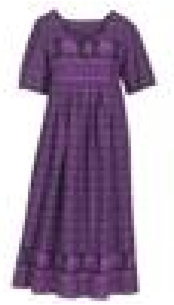

random control

arena

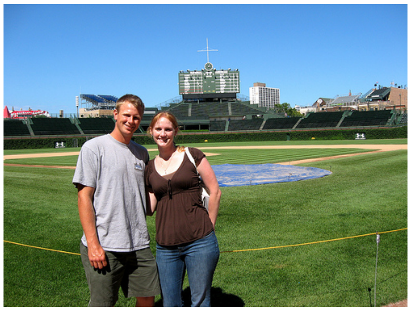

predicted

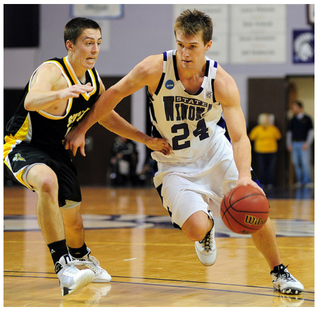

random control

invitation

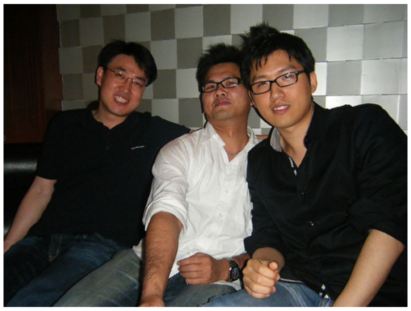

predicted

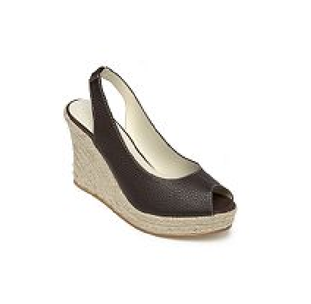

random control

bandit

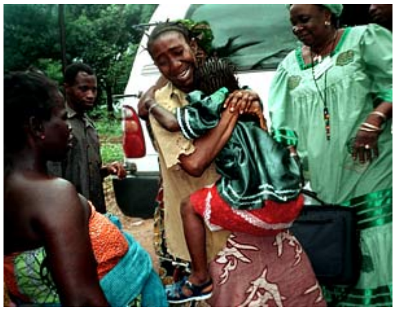

predicted

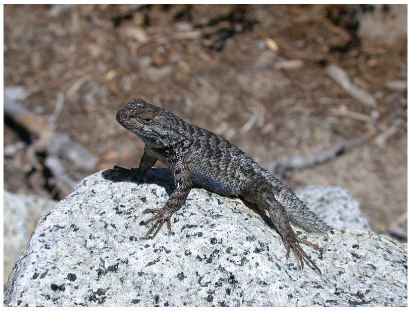

random control

collateral

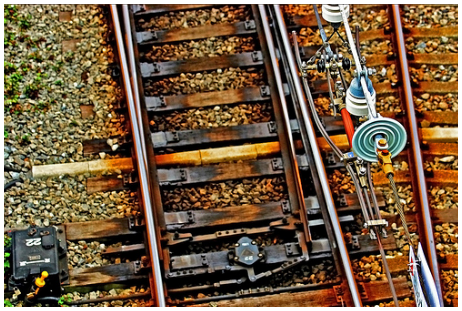

predicted

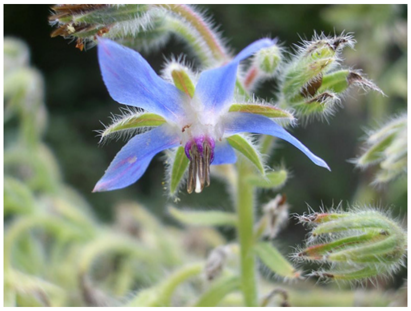

random control

slit

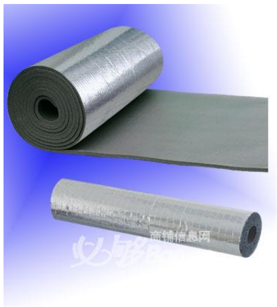

predicted

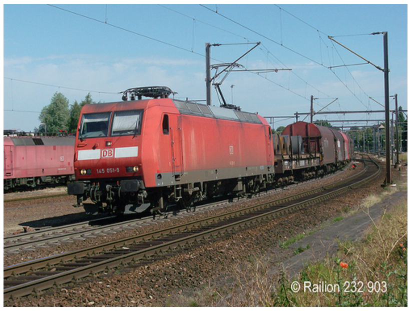

random control

hint

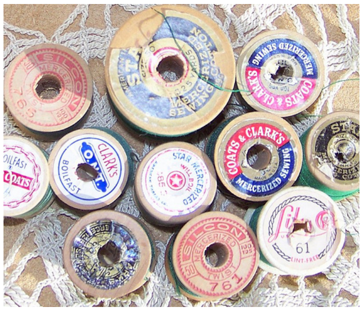

predicted

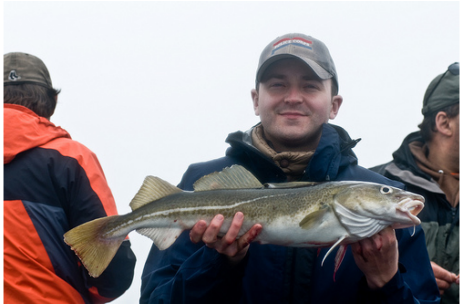

random control

recruiting

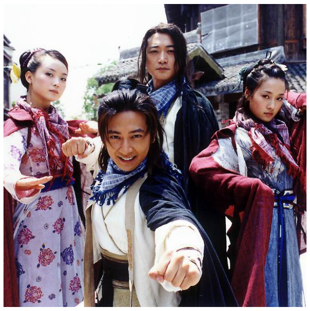

predicted

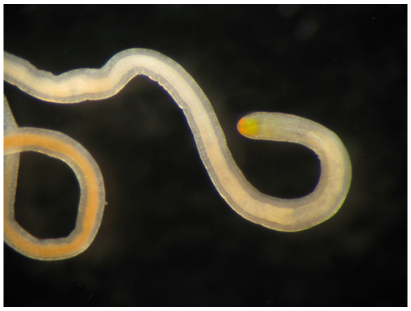

random control

expertise

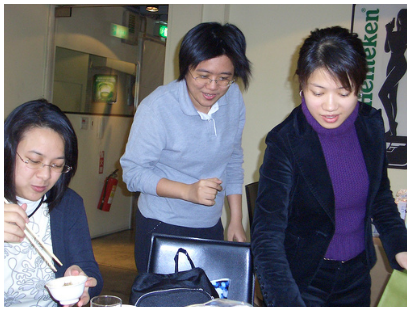

predicted

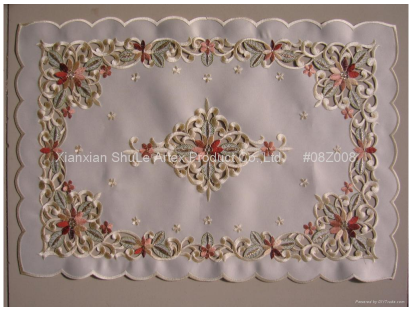

random control

wallpaper

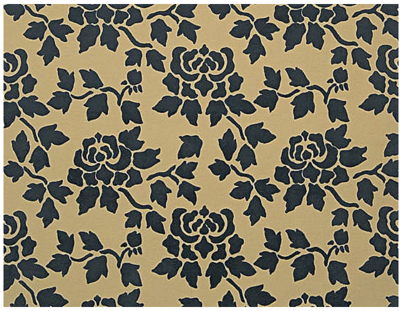

predicted

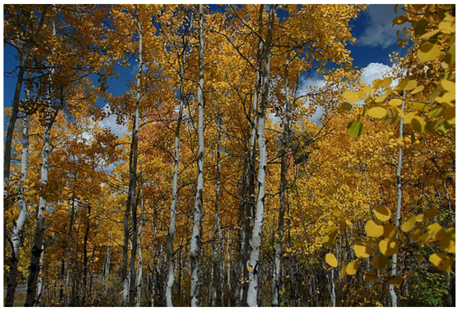

random control

ninety

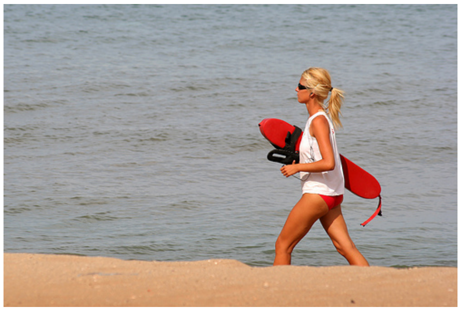

predicted

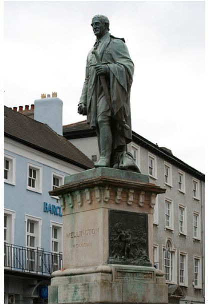

random control

funding

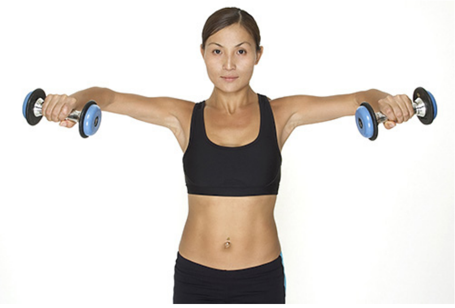

predicted

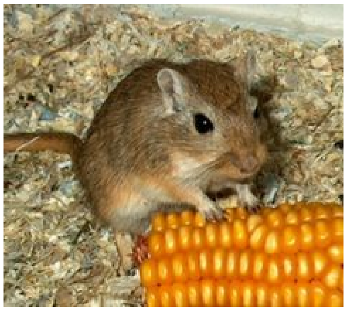

random control

gateway

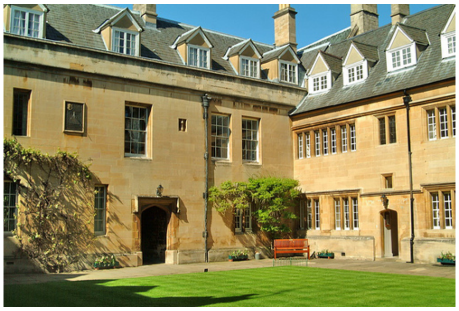

predicted

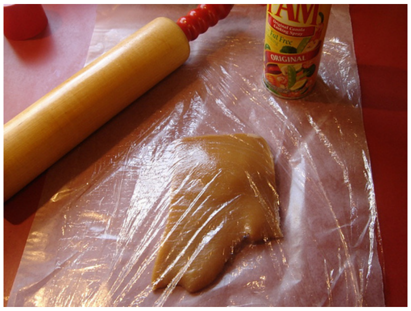

random control

demon

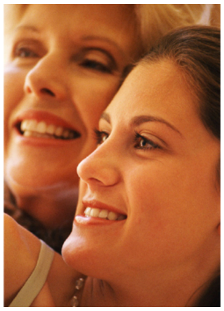

predicted

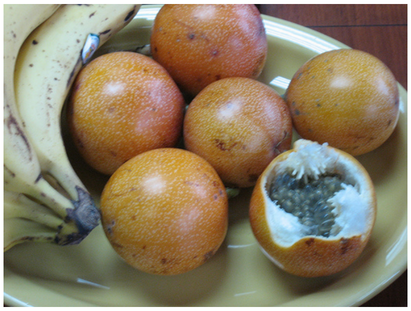

random control

watering

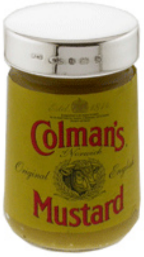

predicted

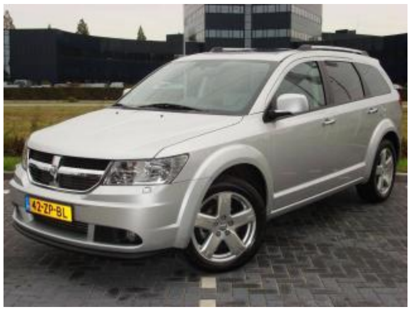

random control

freeze

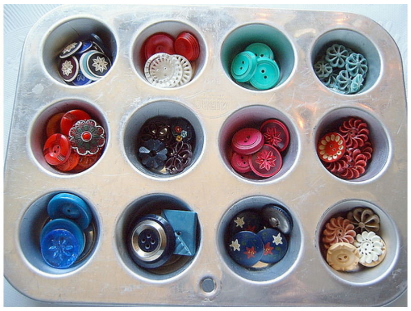

predicted

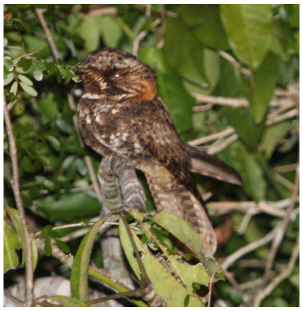

random control

brook

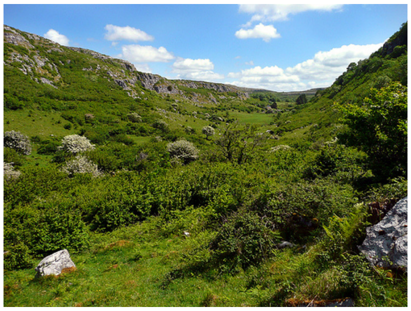

predicted

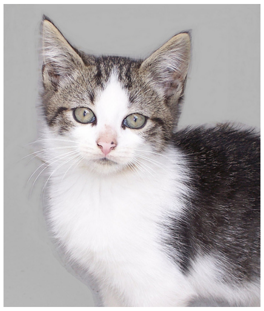

random control

congratulations

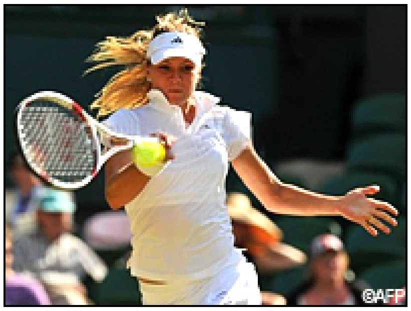

predicted

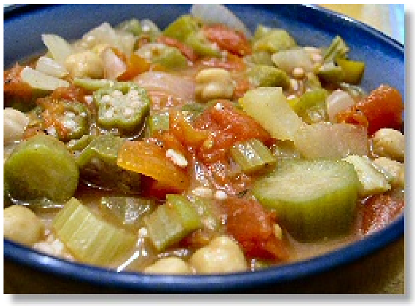

random control

apology

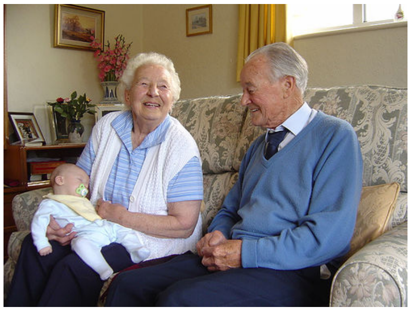

predicted

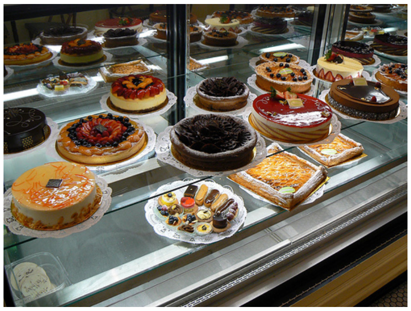

random control

audition

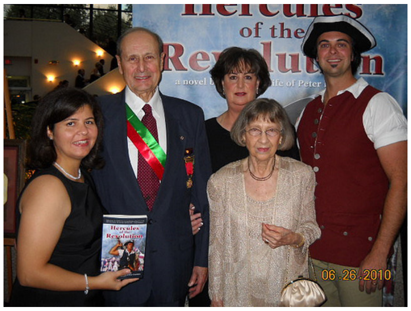

predicted

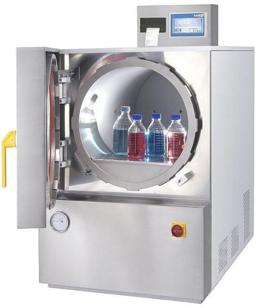

random control

humility

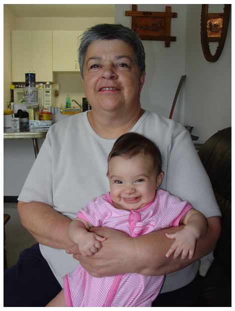

predicted

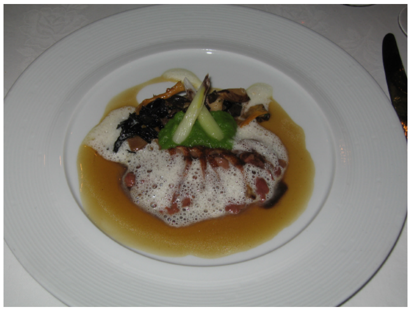

random control

objection

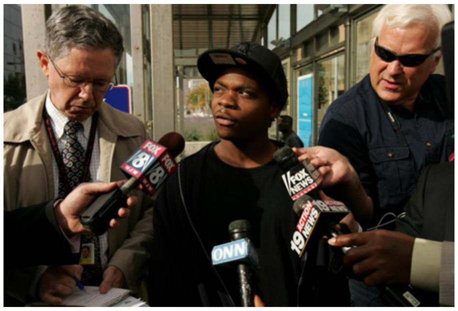

predicted

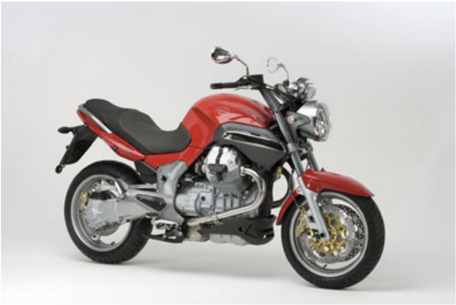

random control

government

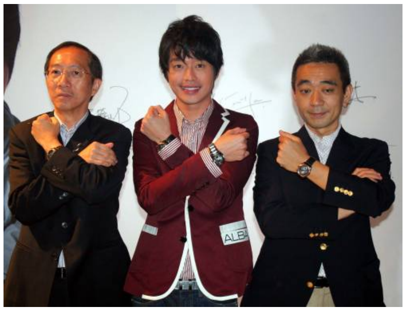

predicted

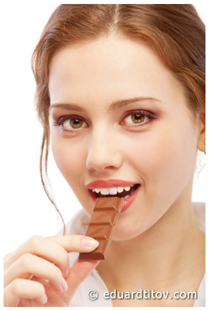

random control

blame

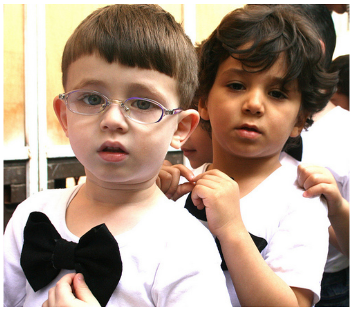

predicted

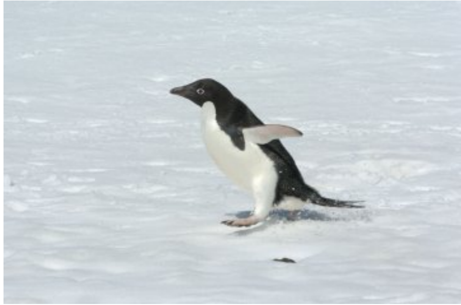

random control

ware

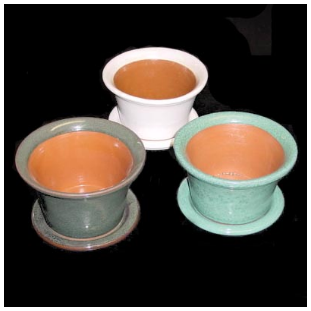

predicted

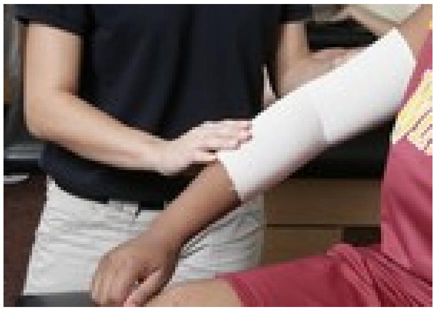

random control

poem

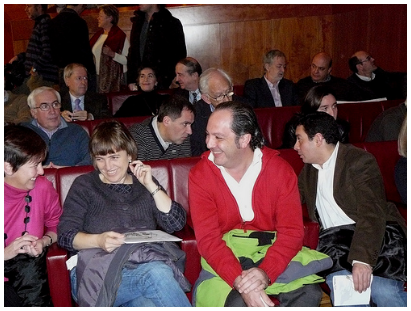

predicted

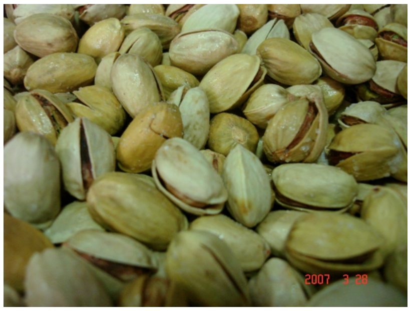

random control

telegram

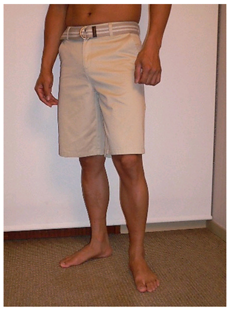

predicted

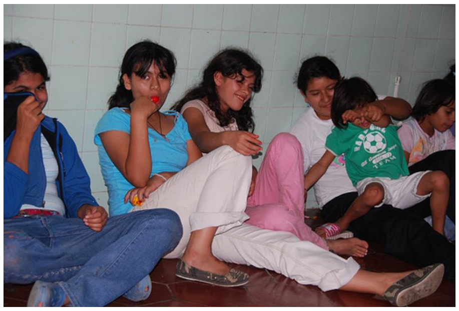

random control

gale

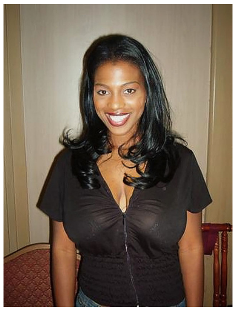

predicted

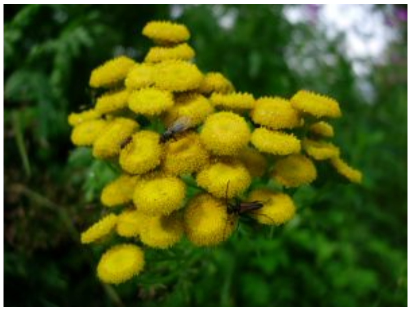

random control

conflict

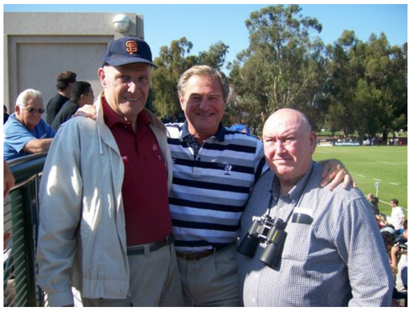

predicted

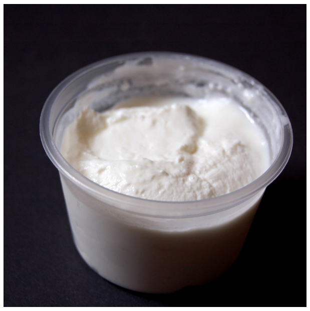

random control

hate

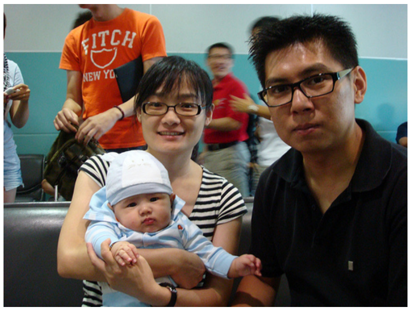

predicted

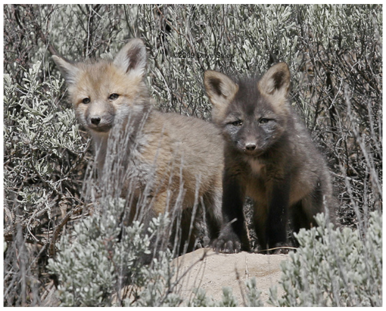

random control

crank

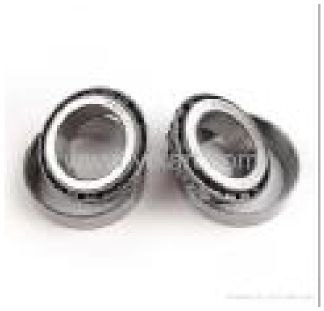

predicted

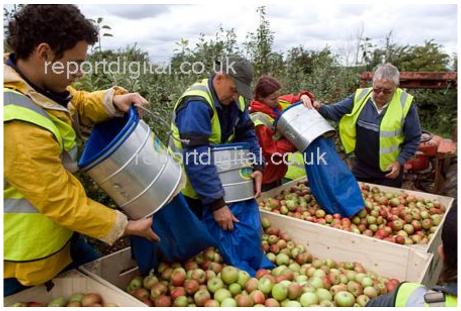

random control

fate

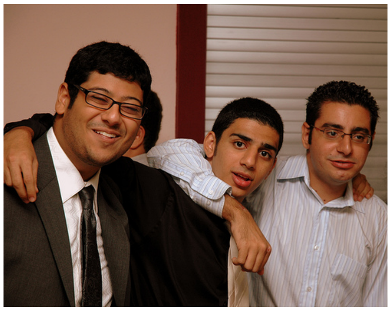

predicted

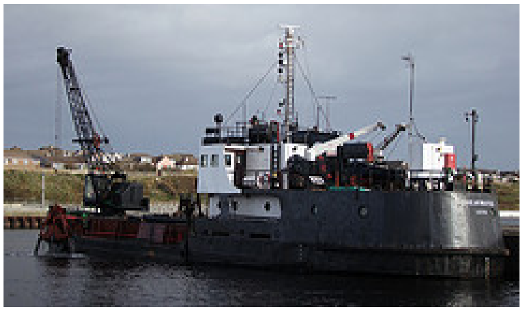

random control

bogey

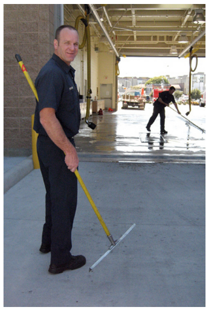

predicted

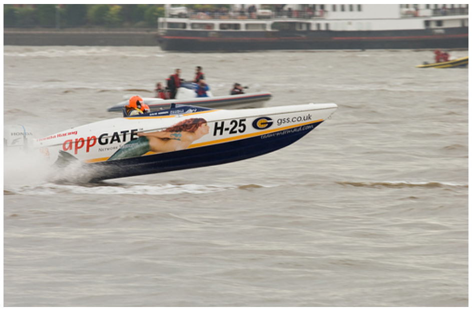

random control

inventory

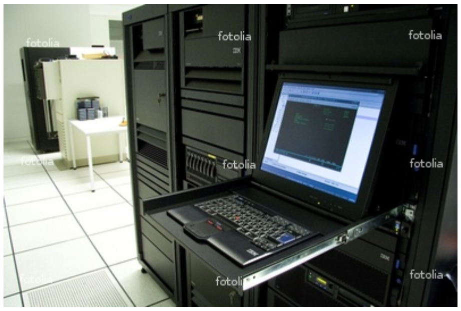

predicted

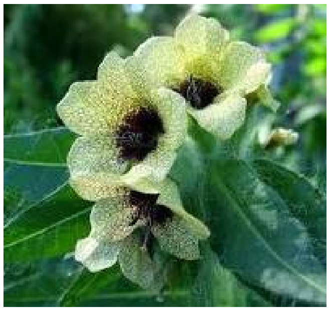

random control

testimony

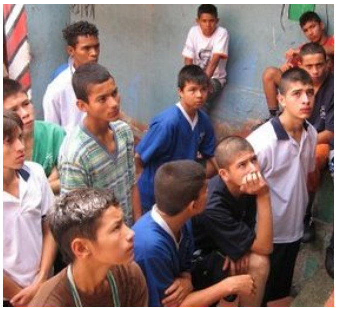

predicted

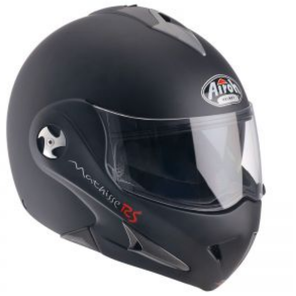

random control

discretion

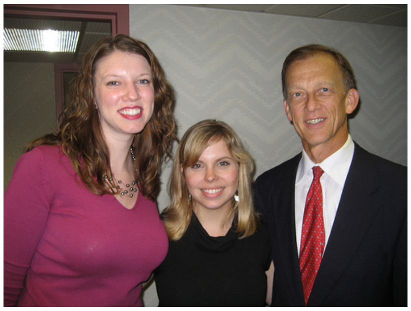

predicted

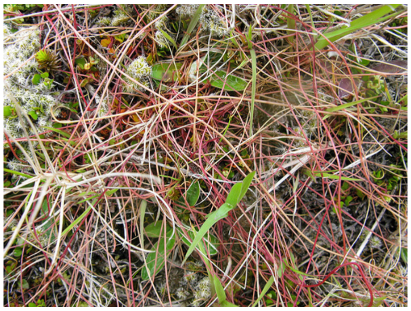

random control

rewrite

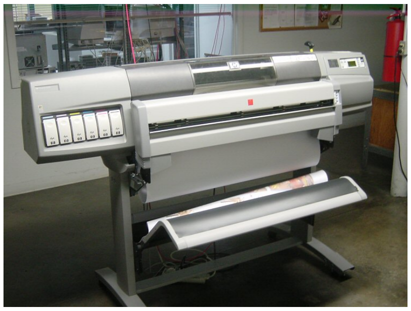

predicted

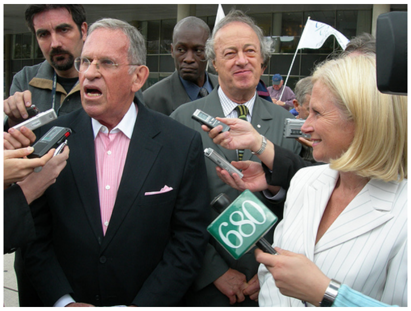

random control

plenty

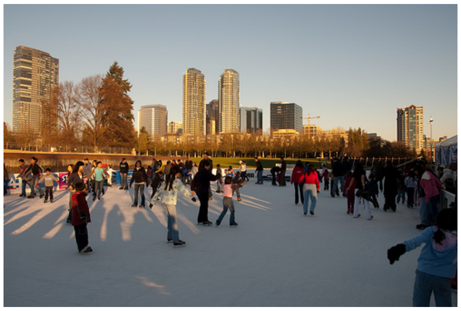

predicted

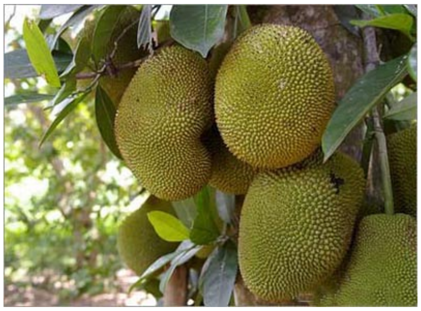

random control

fag

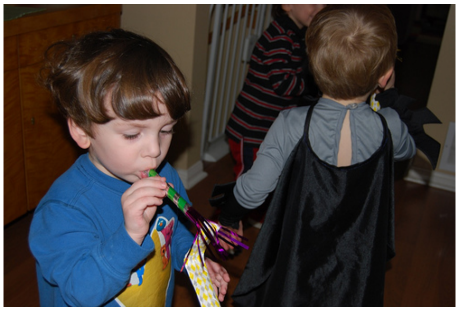

predicted

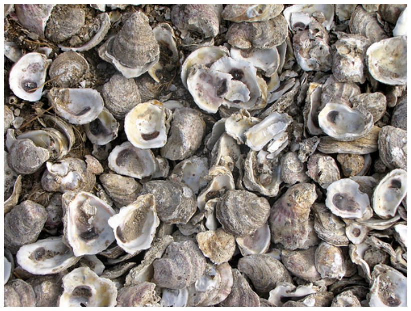

random control

freight

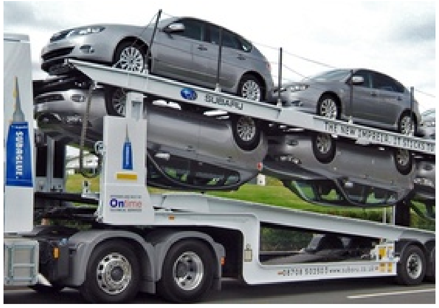

predicted

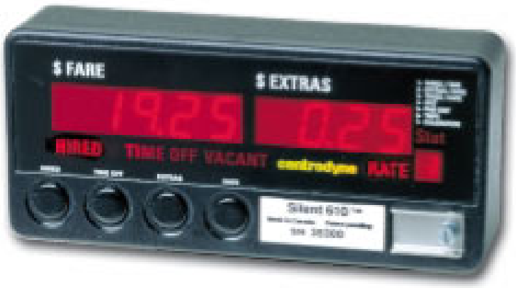

random control

superhero

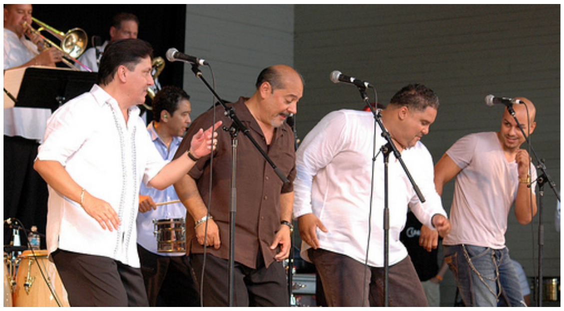

predicted

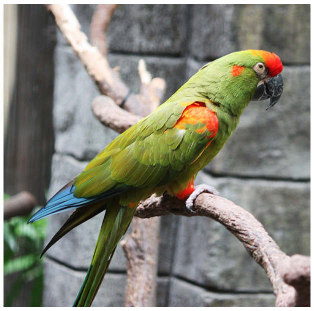

random control

happening

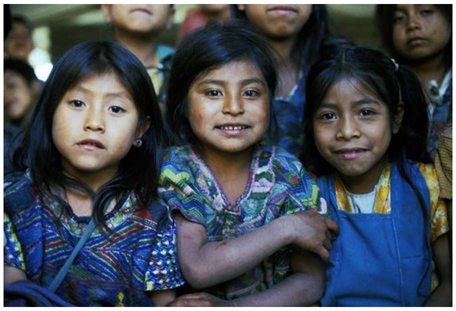

predicted

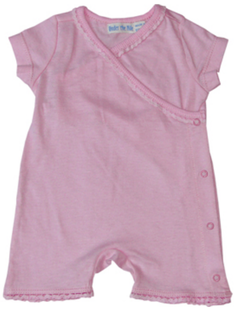

random control

bookstore

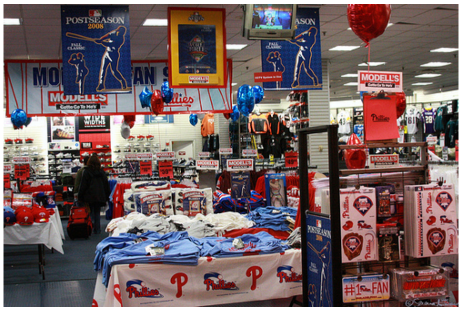

predicted

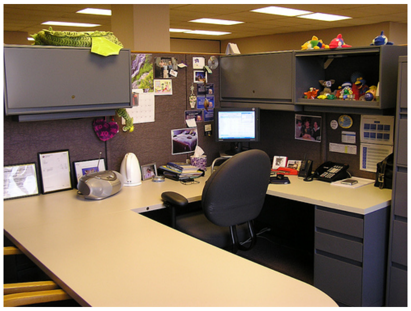

random control

tong

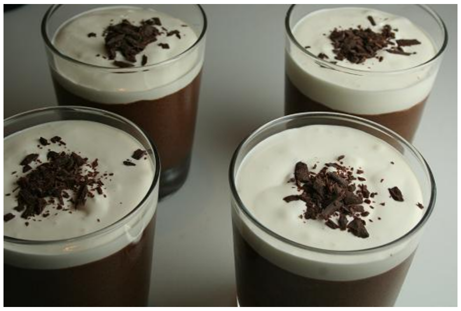

predicted

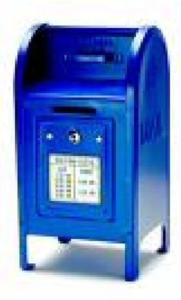

random control

maneuver

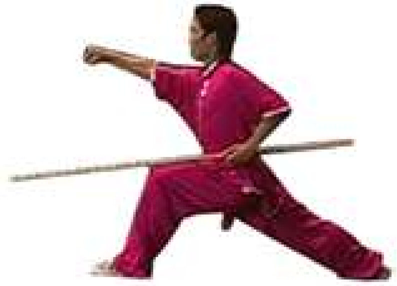

predicted

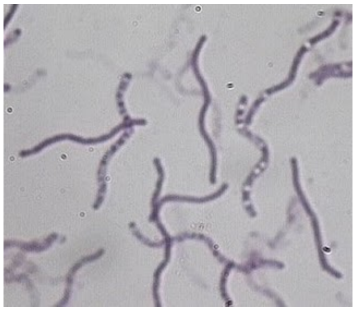

random control

maturity

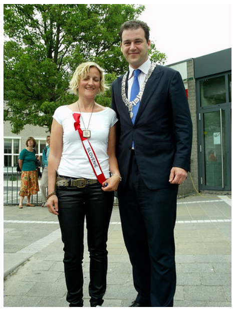

predicted

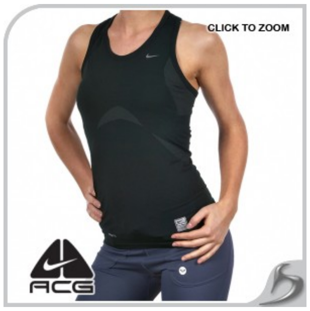

random control

serial

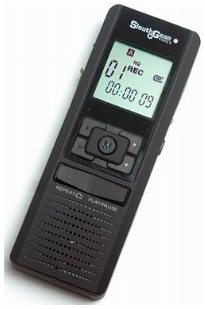

predicted

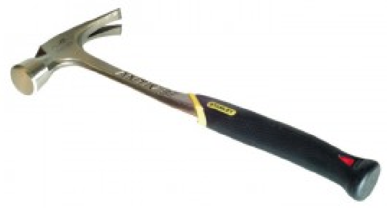

random control

mating

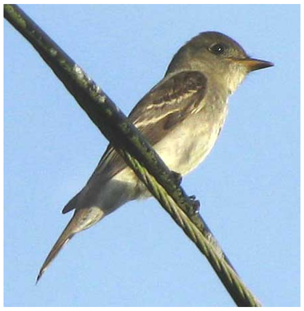

predicted

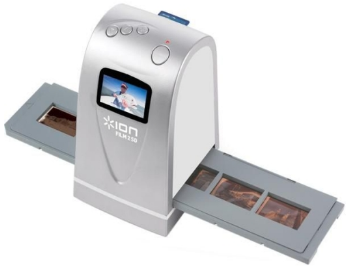

random control

impulse

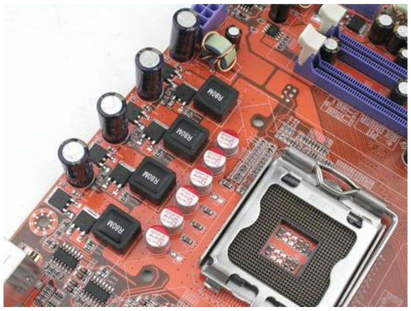

predicted

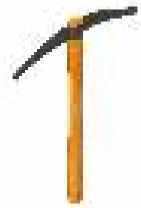

random control

risk

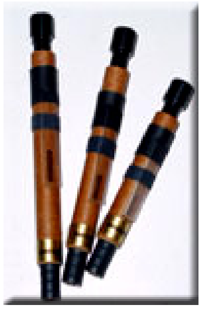

predicted

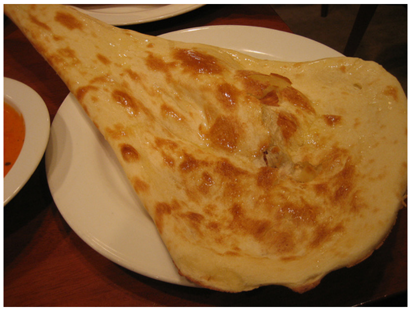

random control

survey

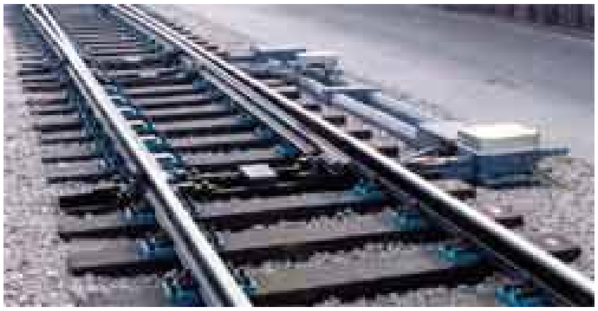

predicted

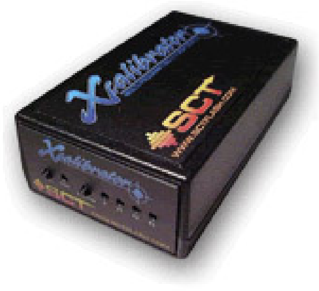

random control

page

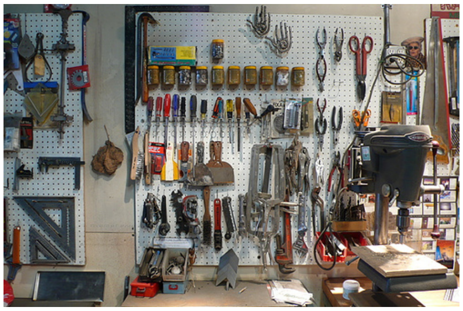

predicted

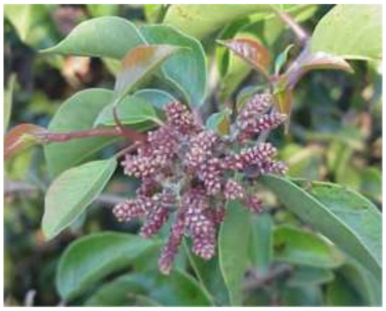

random control

pregnancy

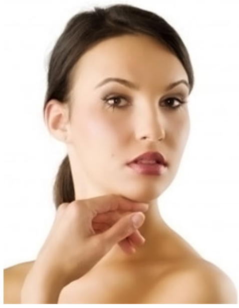

predicted

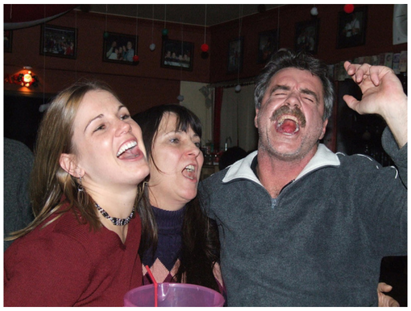

random control

success

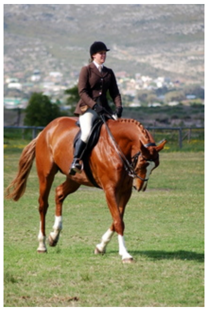

predicted

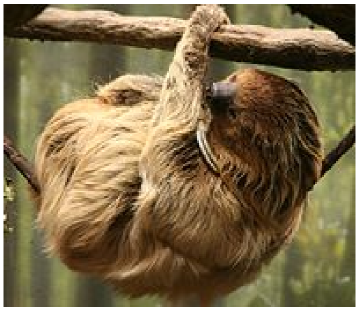

random control

ambition

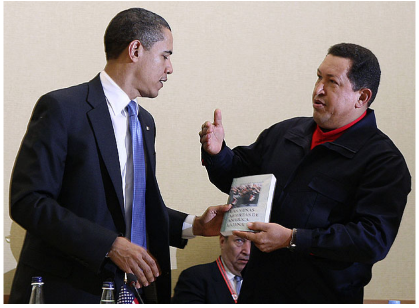

predicted

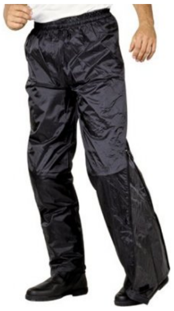

random control

dice

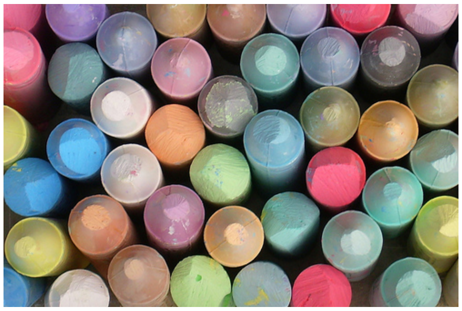

predicted

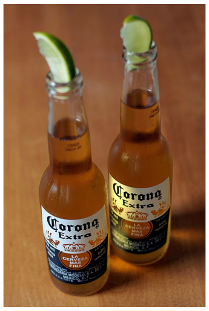

random control

aye

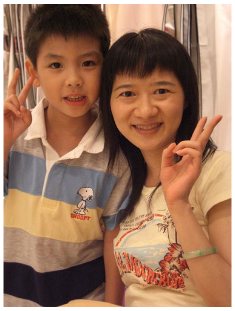

predicted

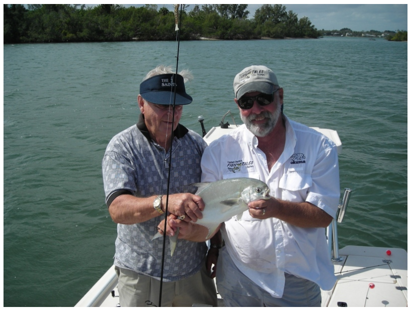

random control

envy

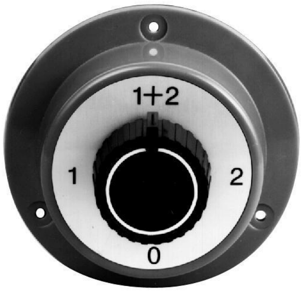

predicted

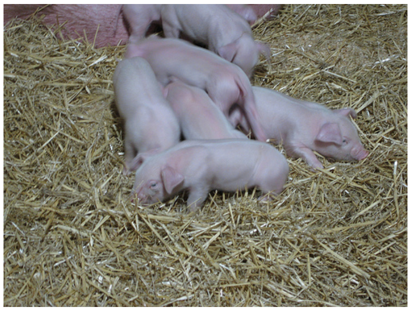

random control

pornography

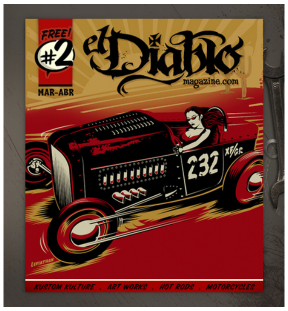

predicted

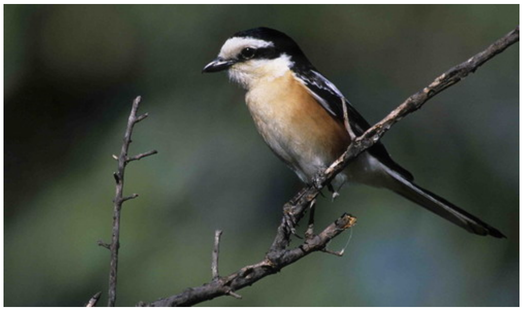

random control

whiskey

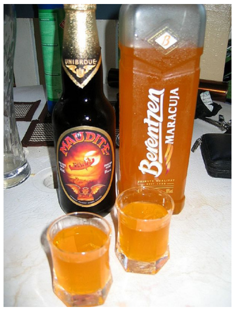

predicted

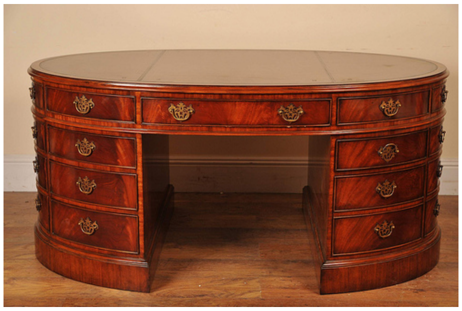

random control

disturbance

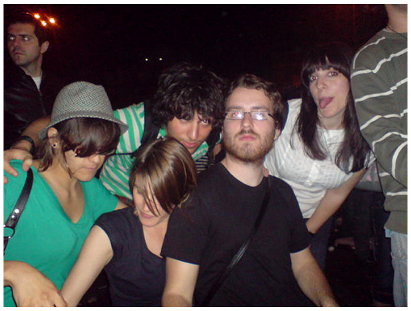

predicted

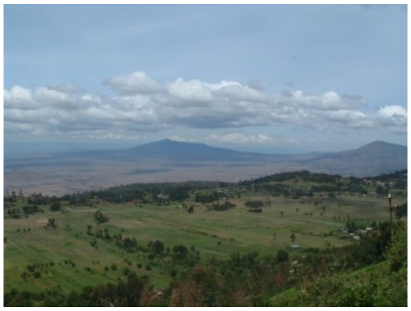

random control

manhood

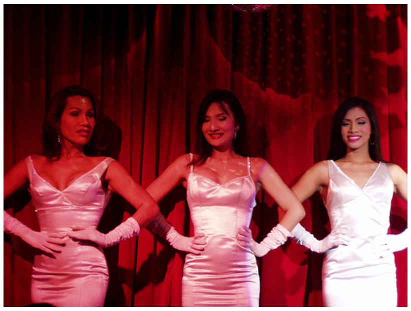

predicted

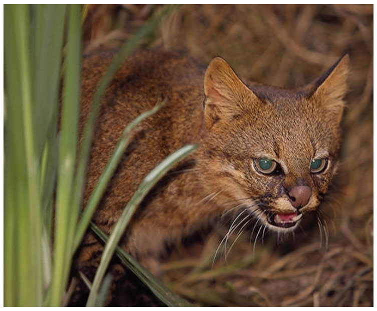

random control

premiere

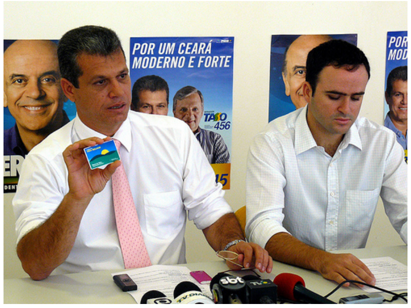

predicted

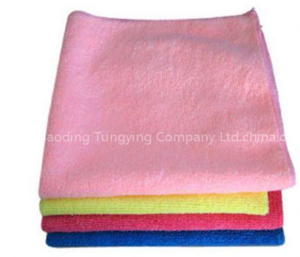

random control
